# miR-1 inhibits progression of high-risk papillomavirus-associated human cervical cancer by targeting G6PD

**DOI:** 10.18632/oncotarget.13344

**Published:** 2016-11-03

**Authors:** Tao Hu, Ye-Fei Chang, zhangang Xiao, Rui Mao, Jun Tong, Bo Chen, Guang-Cai Liu, Ying Hong, Hong-Lan Chen, Shu-Yi Kong, Yan-Mei Huang, Yan-Bin Xiyang, Hua Jin

**Affiliations:** ^1^ Department of Laboratory Medicine, The Third People's Hospital of Yunnan Province, Kunming 650011, Yunnan, PR China; ^2^ Institute of Neuroscience, Kunming Medical University, Chenggong District, Kunming 650500, Yunnan, PR China; ^3^ Laboratory of Molecular Pharmacology, Department of Pharmacology, School of Pharmacy, Southwest Medical University, Luzhou 646000, Sichuan, PR China; ^4^ School of Biomedical Sciences, Faculty of Medicine, The Chinese University of Hong Kong, Shatin, NT. Hong Kong, PR China; ^5^ School of Stomatologya, Kunming Medicine University, Kunming 650500, Yunnan, PR China; ^6^ Physical Eduction Department, Kunming Medical University, Kunming 650500, Yunnan, PR China; ^7^ Experiment Center for Medical Science Research, Kunming Medical University, Chenggong District, Kunming 650500, Yunnan, PR China; ^8^ Department of Anesthesiology, The First People's Hospital of Yunnan Province, Kunming 650032, Yunnan, PR China

**Keywords:** glucose-6-phosphate dehydrogenase, miR-1, cervical cancer, high-risk human papillomaviruses, carcinogenic events

## Abstract

Ectopic glucose-6-phosphate dehydrogenase (G6PD) expression may contribute to tumorigenesis in cervical cancer associated with high-risk human papillomavirus (HR-HPV 16 and 18) infections. Here, we demonstrate that microRNA-1 (miR-1) in association with AGO proteins targets G6PD in HR-HPV-infected human cervical cancer cells. miR-1 inhibited expression of a reporter construct containing a putative G6PD 3′-UTR seed region and suppressed endogenous G6PD expression. Down-regulation of miR-1 increased G6PD expression in cervical cancer cells. Regression analysis revealed that miR-1 levels correlate negatively with the clinicopathologic features in HR-HPV 16/18-infected cervical cancer patients. miR-1 overexpression inhibited proliferation and promoted apoptosis in cervical cancer cells and reduced xenograft tumor growth in nude mice. Conversely, sponge-mediated miR-1 knockdown markedly increased viability and reduced apoptosis in cervical cancer cells and supported neoplasm growth. Restoration of G6PD expression partially reversed the effects of miR-1 overexpression both *in vitro* and *in vivo*. In addition, co-transfection of G6PD siRNA and miR-1 sponge partially reversed miR-1 sponge-induced reductions in cell viability and neoplasm growth. These results suggest that miR-1 suppresses the development and progression of HR-HPV 16/18-infected cervical cancer by targeting G6PD and may be a promising novel therapeutic candidate.

## INTRODUCTION

In 2008, cervical cancer was the third most common malignant cancer, accounting for 9% of new cases (529,800), and the fourth leading cause of cancer-related death, accounting for 8% of such deaths, among women worldwide [1]. Persistent high-risk human papillomaviruses (HR-HPV) are the most important etiologic agent in cervical cancer pathogenesis [2, 3]. HPV genotypes are generally classified into low, high, and intermediate oncogenesis risk types. At present, HPV16, 18, 31, 33, 35, 39, 45, 51, 52, 56, 58, and 59 are considered HR or oncogenic types due to their occurrence in high-grade squamous intraepithelial lesions or cervical cancer [4]. However, the mechanisms underlying HPV pathogenesis are not well understood.

Glucose-6-phosphate dehydrogenase (G6PD) is the rate limiting enzyme in the pentose phosphate pathway (PPP). Functionally, G6PD supplies ribose and NADPH that support biosynthesis and antioxidant defense, and is thus involved in the oxidative stress response [5]. G6PD levels are elevated in various tumors, including melanoma [6], leukemia [7], colon cancers [8], breast cancers [9], and endometrial carcinomas [10]. Our previous data indicated that elevated G6PD levels were also positively correlated with cervical carcinogenesis in 30 to 40-year-old women infected with HR-HPV-16/18. G6PD knockdown decreased proliferative capacity and increased apoptosis in both HPV16+ Siha and HPV18+ Hela cells [5]. These data suggest that G6PD overexpression might contribute to the development and growth of HR-HPV 16/18-associated cervical cancer. However, the sources and regulators of ectopic G6PD expression in carcinogenic events of HR-HPV-16/18-associated cervical cancer, remain unknown.

MicroRNAs (miRNAs) are small noncoding RNAs that bind via base pair interactions with the 3′-untranslated region (UTR) of target mRNA to either degrade the mRNA or repress its translation [11]. miRNAs interact with mRNAs within microribonucleoparticles (miRNPs) through highly evolutionarily conserved molecular mechanisms [12]. At the core of miRNPs, Argonaute (AGO) proteins bind directly to mature miRNAs [13]. Four paralogous human AGO proteins, AGOs 1–4, help orchestrate miRNA activities [12, 14]. Upon binding to AGO proteins, the RNA induced silencing complex (RISC) forms and post-transcriptionally silences gene expression [15]. A single miRNA species, in association with AGO proteins, may target hundreds or even thousands of different mRNAs [16, 17]. Because miRNA:mRNA interactions play a role in many human illnesses, identifying physiological miRNA targets may be particularly helpful [13]. For example, aberrant miRNA expression may contribute to the progression of neurodegenerative diseases [18, 19]. In addition, miRNAs play essential roles in biological processes in tumor cells, such as cell proliferation, differentiation, migration, and invasion [20, 21]. Recent studies have shown that some miRNAs are highly expressed, while others are down-regulated, in tumor tissues [22, 23]. However, whether miRNAs contribute to pathological progression in HR-HPV-associated cervical cancer by targeting G6PD remains unknown.

In this study, we demonstrate that miR-1 might suppress the development and progression of HR-HPV 16/18-infected cervical cancer by targeting G6PD.

## RESULTS

### Validation of miRNA transfection

**Figure 1:**
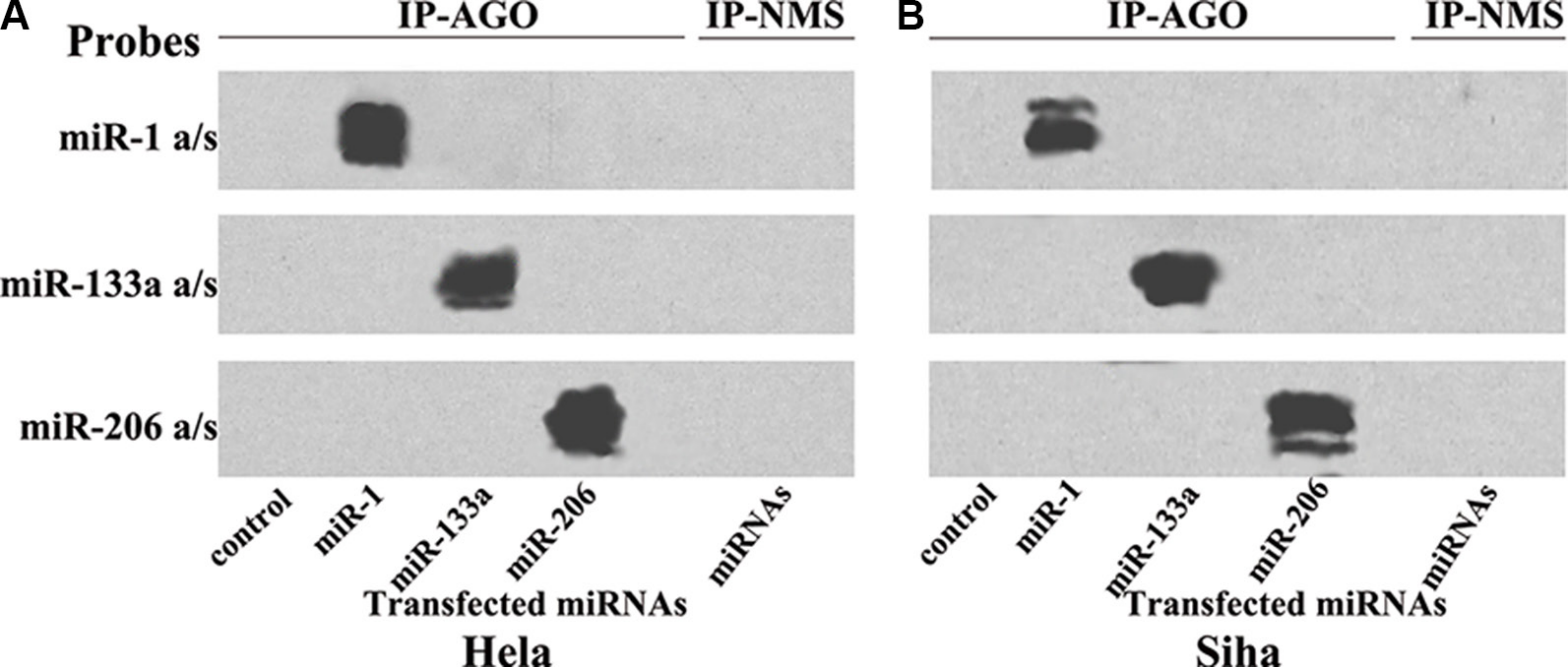
Transfected miRNAs are specifically recruited to miRNPs Northern blot analysis of miRNPs isolated after transfections with miR-1/133a/206 in Hela (**A**) and Siha (**B**) cells; these miRNAs were specifically recruited to miRNPs.

The effectiveness and specificity of miRNA transfections were evaluated using Northern blots (Figure 1). Co-immunoprecipitation (co-IP) revealed that transfected miR-1, miR-133a, and miR-206 were specifically incorporated into miRNPs in both Hela (Figure 1A) and Siha cells (Figure 1B).

### Ribonucleoprotein immunoprecipitation-gene chip (RIP-Chip) assay

RIP-Chip experiments were then performed in both Hela and Siha cervical cancer cells to validate computational predictions. Anti-AGO antibody was used to pull down endogenous AGO-containing miRNP complexes and associated mRNAs after miRNA transfection.

MiRNPs were co-IPed with anti-AGO antibodies bound to protein G agarose beads. RNA associated with AGO protein complexes was then isolated for microarray profiling to identify transcriptome-wide miR-1/133a/206 targets in cervical cancer cells. After confirming successful miR-1/133a/206 transfection, Affymetrix GeneChip microarrays were used to profile mRNAs associated with miRNPs following miR-1/133a/206 transfection.

**Figure 2:**
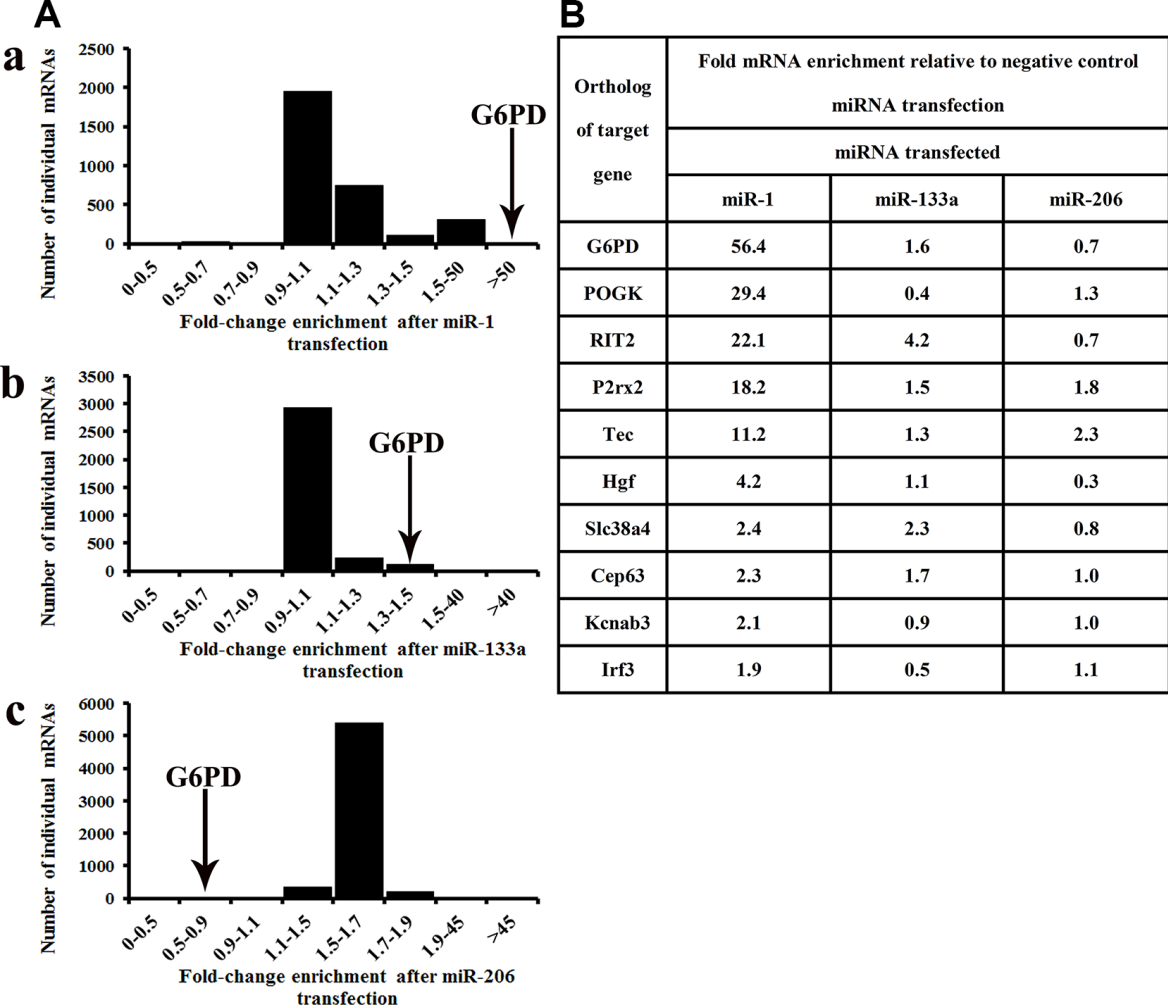
G6PD is a novel target of miR-1 RIP-Chip revealed that G6PD mRNA was recruited to the miRNPs to the greatest degree following transfection with miR-1. (**A-a**) Enrichment in AGO-miRNPs after miR-1 transfection, *n* = 3161; (**A-b**) Enrichment in AGO-miRNPs after miR-133a transfection, *n* = 3336; (**A-c**) Enrichment in AGO-miRNPs after miR-206 transfection, *n* = 5958. Relative G6PD enrichment in miRNPs consistently increased more than 50-fold following miR-1 transfection. The vast majority of mRNAs examined were not enriched in miRNPs following miR-1 transfection (A). (**B**) G6PD and the other top 10 enriched mRNAs following miR-1 transfection. Levels of these miR-1 targets in miRNPs are also shown following miR-133a/206 transfection.

RIP-Chip consistently indicated that G6PD mRNA was more strongly incorporated into miRNPs following miR-1 transfection than the other mRNAs examined (Figure 2A and Figure 2B). By contrast, G6PD mRNA was not enriched in miRNPs following transfection with either miR-133a or miR-206 (Figure 2A–2B and Figure 2A–2C). The top ten miR-1 targets identified by RIP-Chip are shown in Figure 2B; mRNAs enriched following miR-1 transfection are listed in the Supplemental data.

### G6PD is a potential target of miR-1

To further examine whether miR-1 directly targets G6PD mRNA in HR-HPV 16/18-infected (+) cervical cancer cells, G6PD expression was measured using qRT-PCR and Western blot in Hela and Siha cells transfected with miR-1 overexpression or control vectors. Databases were subsequently used to identify the potential target region of miR-1 in the G6PD mRNA 3′-UTR.

G6PD mRNA expression was down-regulated by 71% in Hela (Hela-plenti-miR-1, *P <* 0.01) and by 65% in Siha (Siha-plenti-miR-1, *P <* 0.01) cells overexpressing miR-1. Treatment with plenti-G6PD partially restored G6PD expression in both Hela-plenti-miR-1 and Siha-plenti-miR-1 cells.

**Figure 3:**
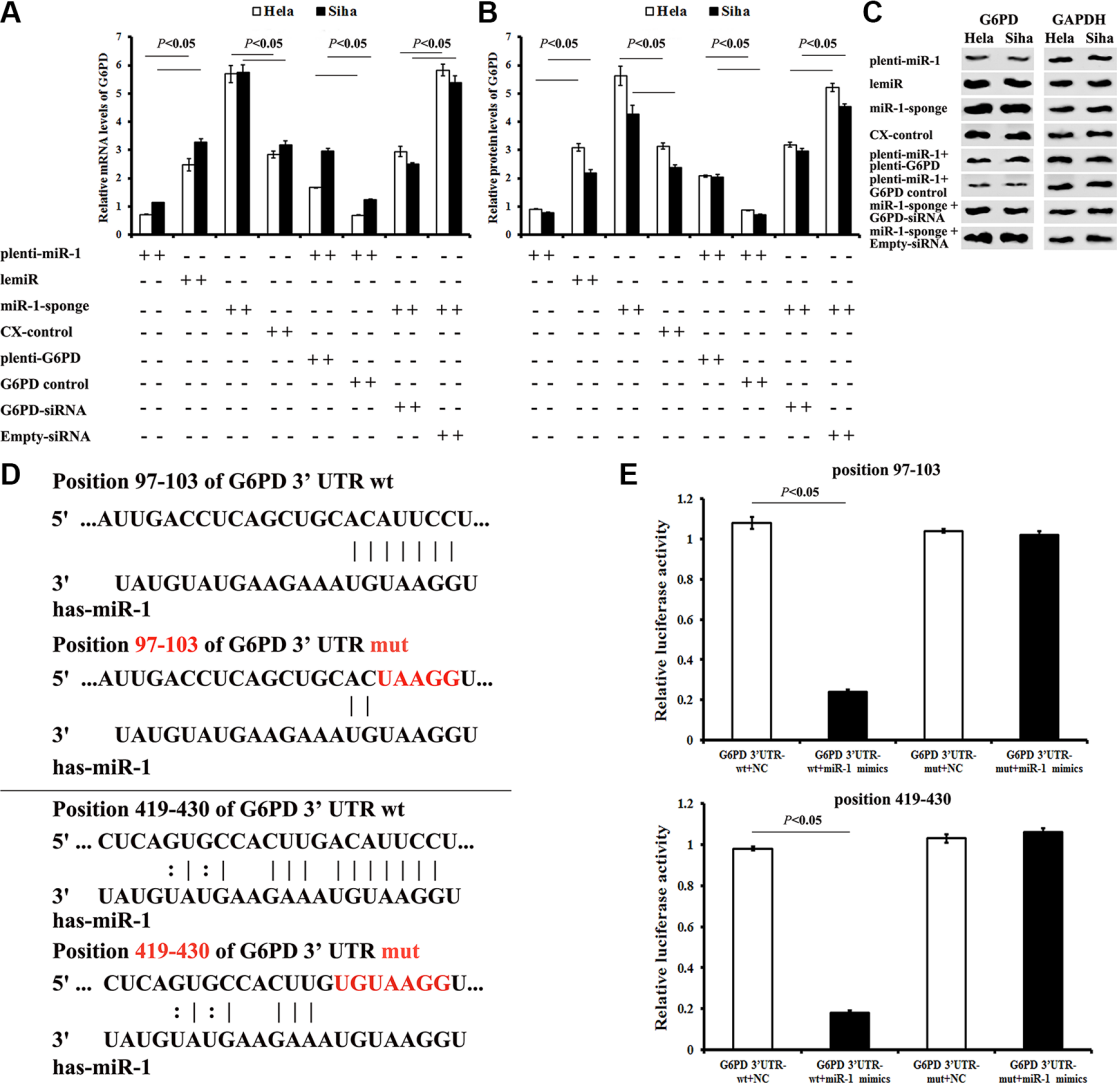
Identification of the G6PD mRNA 3′-UTR seed region directly regulated by miR-1 (**A**) G6PD mRNA expression in cervical cancer cells after different treatments. (**B**) G6PD protein levels in cervical cancer cells after different treatments. (**C**) Representative Western blots for G6PD protein expression. (**D**) Seed regions directly regulated by miR-1 were identified. To generate seed region mutations, both G6PD mRNA 3′-UTR “AUUCC” sites were mutated to “UAAGG”. (**E**) Relative luciferase activity of miR-1 mimics co-transfected with G6PD 3′-UTR-wt or G6PD 3′-UTR-mut was detected using a dual-luciferase reporter test. All data are representative of five independent experiments and are presented as means ± SE (*n* = 5).

In contrast, inhibition of miR-1 increased G6PD mRNA expression 2.3-fold in Hela cells and 1.8-fold in Siha cells (both *P <* 0.05) (Figure 3A). G6PD-siRNA treatment partially reversed these miR-1 inhibition-induced effects. Similar changes in G6PD protein levels were also observed in Siha and Hela cells after transfection with various chemicals (Figure 3B and 3C). These findings suggest that miR-1 targets G6PD.

All of the databases examined predicted two potential miR-1 target regions in the G6PD mRNA 3′-UTR (“seed regions”) (Figure 3D). To verify direct interactions between miR-1 and the seed regions, a wild-type G6PD 3′-UTR (G6PD 3′-UTR-wt) and a chemically synthesized G6PD 3′-UTR with two seed region mutations(G6PD 3′-UTR-mut) were cloned into dual-luciferase reporter plasmids. The plasmids were then co-transfected with miR-1 mimics or miRNA negative control (NC). Luciferase activity decreased by approximately 77% when miR-1 mimics were co-transfected with the G6PD 3′-UTR-wt plasmid (*P* < 0.01), but not with the G6PD 3′-UTR-mut plasmid (*P* > 0.05) (Figure 3E). These data demonstrated that miR-1 down-regulated G6PD expression by binding to the predicted regions of the G6PD mRNA 3′-UTR.

### Decreased miR-1 expression is associated with pathological features in HR-HPV-infected cervical cancer patients

All 60 patients with pathologically diagnosed cervical cancer were HPV DNA-positive (identified by PCR), and 88.33% (53/60) of these patients were positive for HR-HPV 16/18. The age range for these patients was 38 to 71 years, with a median age of 48 years. 18.1% had multiple HPV infections, and HPV16 infection was the most prevalent type (38.8%), followed by HPV-18 (35.1%), HPV-31 (9.2%), HPV-52 (6.3%), HPV-39 (5.5%), and HPV-58 (5.1%).

Fifty-seven histopathologically-confirmed cervical cancer specimens were obtained from these 60 patients. The remaining three samples were necrotic and unsuitable for further analysis.

**Figure 4:**
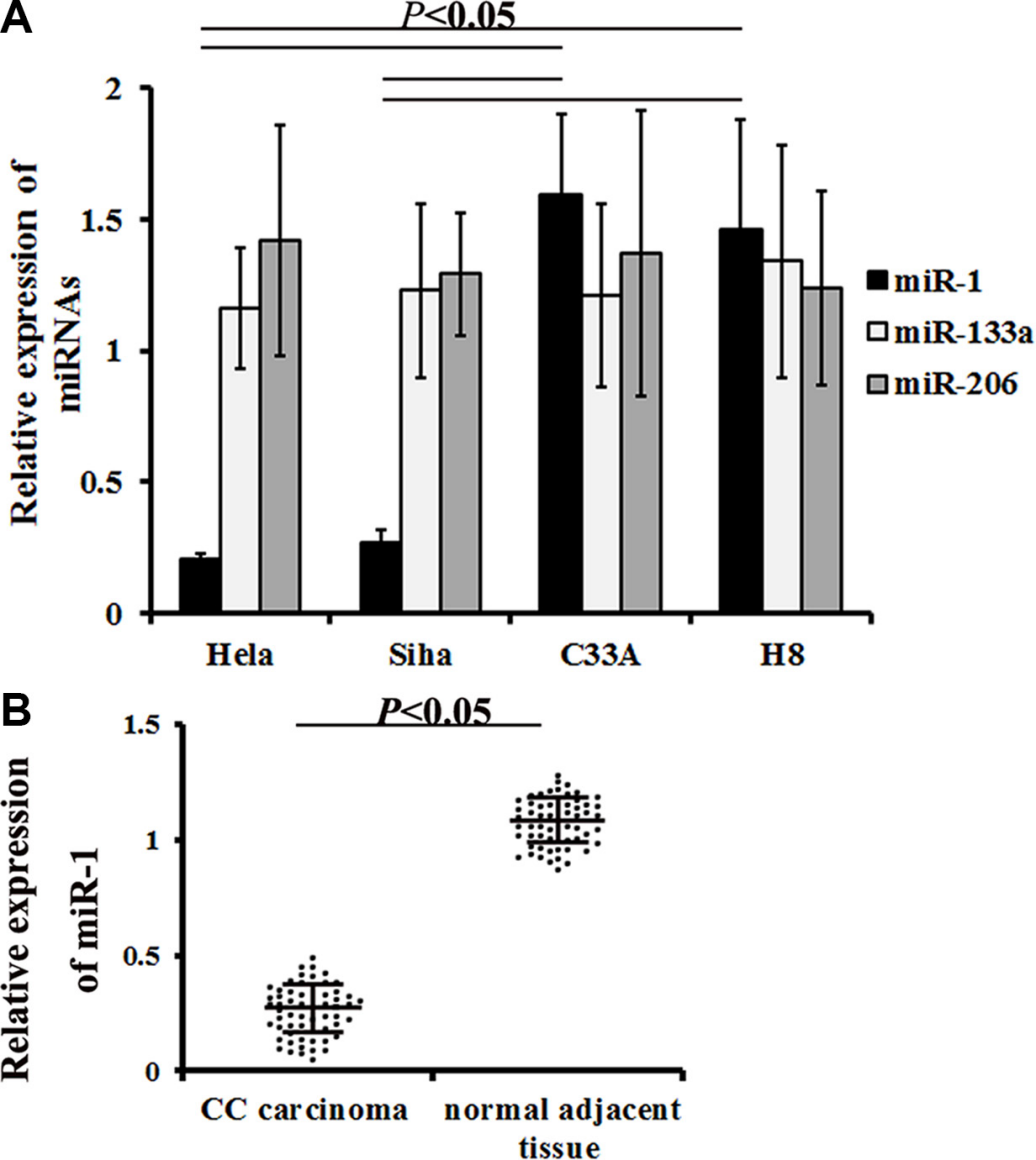
miR-1 expression in cervical cancer cells and samples qRT-PCR was used to measure miR-1/133a/206 expression in different cervical cancer cells and in carcinoma samples from cervical cancer patients. (**A**) Relative miR-1/133a/206 levels in different cells. Data are presented as means ± SE (*n* = 7). (**B**) Relative miR-1 levels detected in patient specimens. Data are presented as means ± SE (*n* = 57).

miR-1/133a/206 expression was evaluated in different cervical cancer cell lines using qRT-PCR. miR-1 expression decreased in Hela and Siha cells compared to C33A cells (0.21 ± 0.02 in Hela vs. 1.59 ± 0.31 in C33A, *P* = 0.000000; 0.27 ± 0.05 in Siha vs. 1.59 ± 0.31 in C33A, *P* = 0.000001) and H8 cells (0.31 ± 0.06 in Hela vs. 1.46 ± 0.42 in H8, *P* = 0.000000; 0.39 ± 0.08 in Siha vs. 1.46 ± 0.42 in H8, *P* = 0.000000). However, neither miR-133a nor miR-206 expression differed in HR-HPV+ cervical cancer cells compared to control cells (Figure 4A).

To determine whether increased miR-1 expression was associated with cervical cancer, surgical tissue samples from 57 HR-HPV+ cervical cancer patients and matched controls were examined. qRT-PCR revealed that miR-1 levels were lower in neoplasm tissues than in normal tissues (0.34 ± 0.04 vs. 1.28 ± 0.21, *P* = 0.000007, Figure 4B). All enrolled patients were then divided into two groups based on miR-1 levels; patients with miR-1 levels less than or equal to the median were assigned to the low level group, while those with miR-1 levels greater than the median were assigned to the high level group [33].

We then performed regression analysis to examine the association between miR-1 levels and clinicopathologic parameters. Low miR-1 expression was correlated with FIGO stages I–II (I, *P* = 0.000; II, *P* = 0.000), increased cell differentiation (well, *P* = 0.000; moderate, *P* = 0.001), and tumor diameter (≤ 4, *P* = 0.000; > 4, *P* = 0.03, Table 1). In addition, patients with low miR-1 levels in cervical cancer samples had higher HR-HPV 16 and HPV 18 infection rates (*P* < 0.05, Table 2).

**Table 1:** Multivariate analysis of HPV status and miR-1 levels in women diagnosed as CC (*N* = 57)

	HPV status (HR-HPV-16/18)						miR-1 expression (qRT-PCR detection)					
	HPV-16-/18-Negative		HPV-16+/18+Positive		Adjusted OR (95% CI)		miR-1 low level (≤ median)		miR-1 high level (> median)		Adjusted OR (95% CI)	
	n	(%)	n	(%)			n	(%)	n	(%)		
**FIGO stage**												
I	2	3.5	2	3.5	1.24	0.62–2.39	28	49.1	1	1.8	**29.71**	**14.64–32.98**
II	3	5.3	9	15.8	1.45	0.78–2.61	14	24.6	2	3.5	**14.02**	**10.48–30.13**
III	1	1.8	14	24.6	**14.72**	**11.04–23.61**	8	14.0	1	1.8	1.31	0.61–1.96
IV	1	1.8	25	43.9	**26.95**	**17.81–35.74**	2	3.5	1	1.8	1.12	0.39–1.35
**Differentiation**												
well	5	8.8	4	7.0	1.31	0.65–2.49	34	59.6	1	1.8	**35.79**	**20.34–45.77**
moderate	1	1.8	16	28.1	**10.74**	**6.71–14.21**	15	26.3	1	1.8	**10.11**	**5.12–19.63**
poor	1	1.8	30	52.6	**36.38**	**23.95–48.65**	1	1.8	3	5.3	1.12	0.39–1.35
**Tumor diameter (cm**)												
≤ 4	5	8.8	10	17.5	1.82	1.33–5.01	42	73.7	4	7.0	**51.23**	**32.03–64.49**
> 4	2	3.5	40	70.2	**49.03**	**29.92–58.12**	8	14.0	1	1.8	4.63	2.41–7.92

95% CI: confidence interval, OR: odds ratio, Values in bold: *p* < 0.05.

**Table 2:** The relationship between miR-1 expression and HPV 16/18 infected in CC patients (*N* = 57)

HPV status	miR-1 levels (cervical cancer vs normal adjacent tissue)					
	reduced by 10–20%		reduced by 30–40%	
	OR	95% CI	OR	95% CI
HPV 16+	9.62	4.36–15.68	23.24	19.61–34.38
HPV 18+	10.03	5.12–13.99	21.25	15.16–38.92

95% CI: confidence interval, OR: odds ratio,

Values in bold: *p* < 0.05.

### MiR-1 inhibits proliferation and promotes apoptosis in cervical cancer cells by down-regulating G6PD

**Figure 5:**
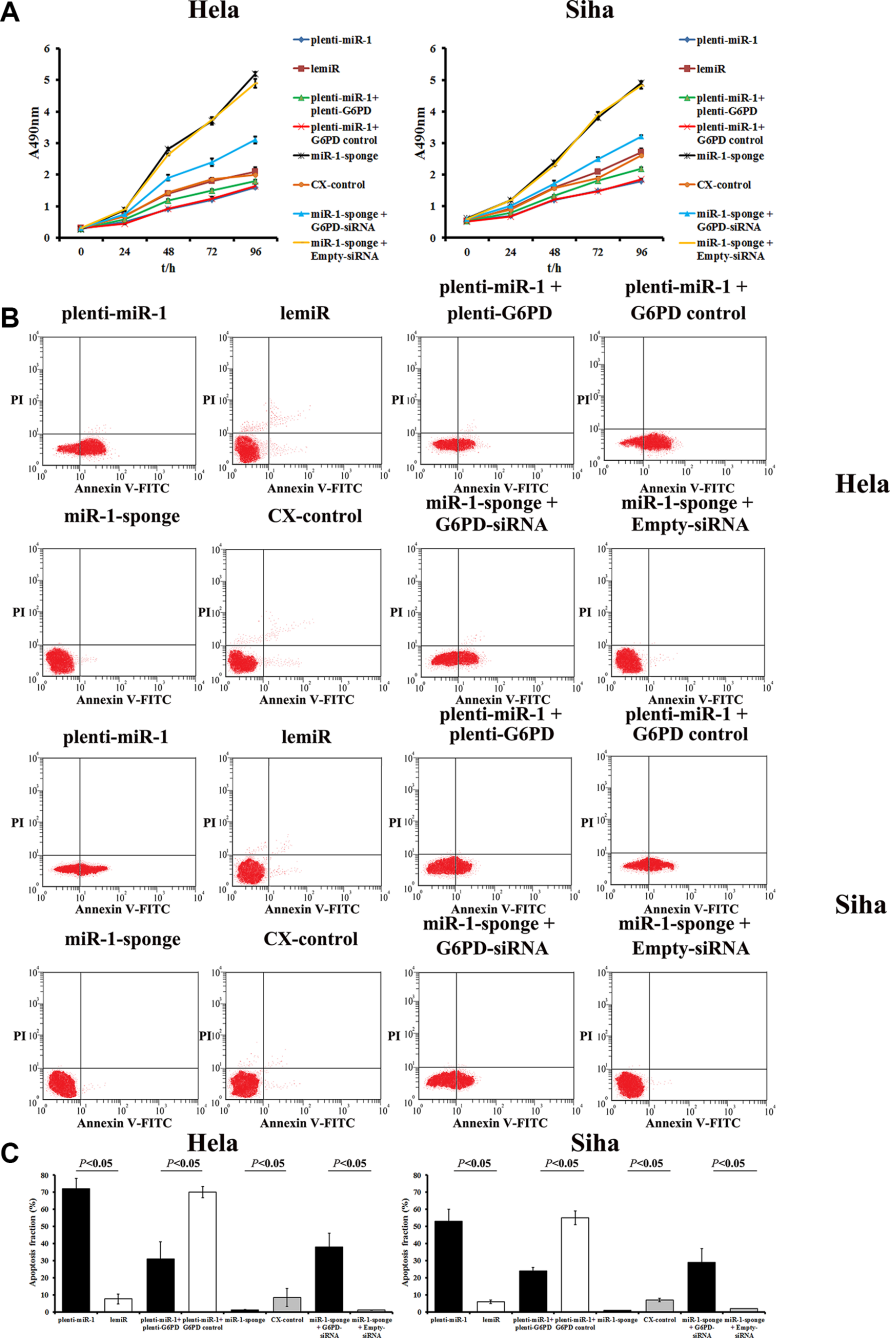
MiR-1 inhibits proliferation and promotes apoptosis in cervical cancer cells by down-regulating G6PD (**A**) MTT assay showing the time course of changes in viability in Hela and Siha cells transfected with plenti-miR-1, lemiR, plenti-miR-1+plenti-G6PD, plenti-miR-1+G6PD control, miR-1-sponge, CX-control, miR-1-sponge+G6PD-siRNA, or miR-1-sponge+Empty-siRNA. Data are presented as means ± SE (*n* = 5). (**B**) miR-1 increased apoptosis rates in cervical cancer cells by inhibiting G6PD. Hela and Siha cells were transfected with plenti-miR-1, lemiR, plenti-miR-1+plenti-G6PD, plenti-miR-1+G6PD control, miR-1-sponge, CX-control, miR-1-sponge+G6PD-siRNA, or miR-1-sponge+Empty-siRNA. (**C**) Percentages of apoptotic cells after different treatments. Data are presented as means ± SE (*n* = 5).

An MTT assay revealed that relative proliferative capacity decreased in miR-1-overexpresing Hela and Siha cells after 48, 72, and 96 h compared to matched controls (*P* < 0.05, Figure 5A). In contrast, proliferation increased in Hela and Siha cells after miR-1 sponge transduction compared to matched CX-controls (*P* < 0.05, Figure 5A).

Meanwhile, apoptosis rates markedly increased in miR-1-overexpressing cells compared to control lemiR-infected cells (Figure 5B). Conversely, apoptosis decreased in the miR-1-sponge transfected Hela and Siha cells compared to CX-control cells (*P* < 0.05, Figure 5B).

Plenti-G6PD treatment partially attenuated the inhibition of proliferation and increase in apoptosis caused by miR-1 overexpression in Hela-plenti-miR-1 and Siha-plenti-miR-1 cells (Figure 5). Similarly, co-transfection of miR-1-sponge and G6PD-siRNA neutralized the increase in proliferative capacity and inhibition of apoptosis induced by miR-1-sponge treatment alone.

### Effects of miR-1/G6PD on tumor formation in nude mice

**Figure 6:**
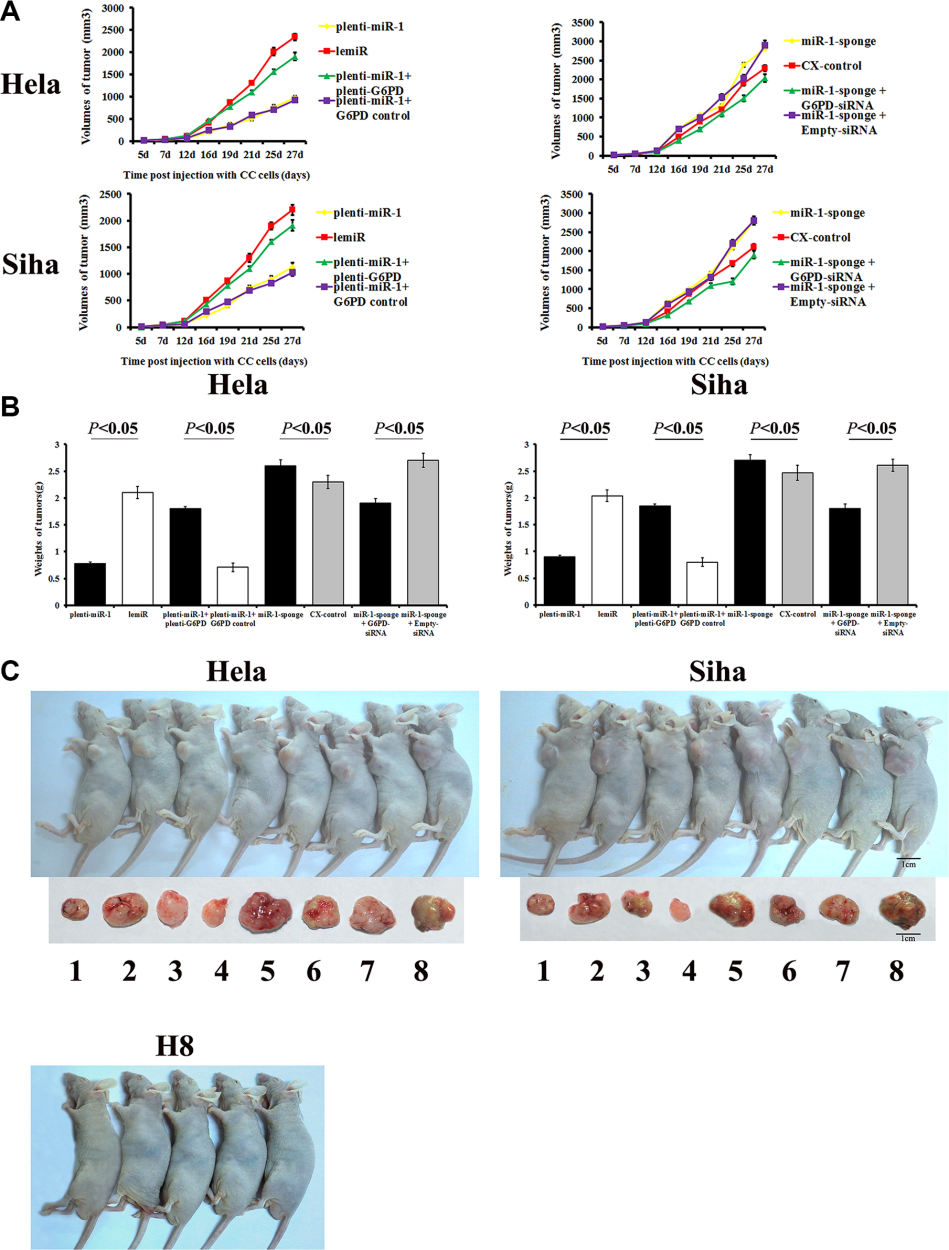
Tumor formation and growth in nude mice after cervical cancer cell xenografts H8 cells, Hela and Siha cells transfected with plenti-miR-1, lemiR, plenti-miR-1+plenti-G6PD, plenti-miR-1+G6PD control, miR-1-sponge, CX-control, miR-1-sponge+G6PD-siRNA, or miR-1-sponge+Empty-siRNA were injected into nude mice. Tumor size was measured on days 7, 12, 16, 19, 21, 25, and 27 post-injection, and a tumor volume growth curve (**A**) was plotted. Mice were photographed on day 27 (**C**) and then sacrificed. Tumors were isolated and tumor weights were measured (**B**). (C) From 1 to 8, Hela or Siha cells transfected with plenti-miR-1, lemiR, plenti-miR-1+plenti-G6PD, plenti-miR-1+G6PD control, miR-1-sponge, CX-control, miR-1-sponge+G6PD-siRNA, or miR-1-sponge+Empty-siRNA, respectively. Data are presented as means ± SE (*n* = 5).

Tumor formation was measured in mice after injections of different cervical cancer cells. Neoplasms formed last and grew slowest in mice treated with plenti-miR-1-transfected cells compared to the other treatment groups (plenti-miR-1 and plenti-miR-1 + G6PD control groups). Tumor growth was fastest in mice treated with miR-1 sponge-treated cervical cancer cells (miR-1-sponge and miR-1-sponge + Empty-siRNA groups, Figure 6). Tumor sizes were smaller in the miR-1 overexpression groups (plenti-miR-1 and plenti-miR-1 + G6PD control) than in the other groups between 16 and 27 days post-injection (*P* < 0.05).

Tumors were larger in miR-1 sponge-treated groups than in the other groups 16 days post-injection (*P* < 0.05). However, partial G6PD knockdown counteracted miR-1 overexpression-induced growth (miR-1-sponge + G6PD-siRNA vs. miR-1-sponge + Empty-siRNA, *P* < 0.05).

At 27 days post injection, tumors were smallest in the G6PD-deficient siRNA-treated group and the plenti-miR-1 treated group and largest in the miR-1 sponge-transfected groups. Plenti-G6PD-induced G6PD overexpression partially reversed the inhibition of xenograft growth resulting from plenti-miR-1 treatment alone. Tumor weights did not differ between lemiR and CX-control cells (*P* > 0.05, Figure 6).

These results indicate that miR-1 inhibited proliferation and promoted apoptosis and tumor formation in HR-HPV+ cervical cancer by down-regulating G6PD.

## DISCUSSION

We previously demonstrated that overexpression of G6PD in cervical cancer was positively correlated with cervical cancer development in patients infected with HR-HPV 16/18 [5]. However, the mechanisms by which ectopic G6PD expression contributes to these pathological changes are poorly understood. Here, we explored possible correlations between miRNA and G6PD levels and carcinogenic events in human cervical cancer patients with HR-HPV16/18 infections.

Our results demonstrate that miR-1 inhibited G6PD expression in human cervical cancer cells and tumors. Computational predictions, RIP-Chip assays, and dual-luciferase reporter assays revealed that G6PD mRNA was the most highly-expressed target of miR-1 in cultured HR-HPV+ Hela and Siha cells. Furthermore, regression analysis revealed that increased miR-1 levels in HR-HPV 16/18-infected cervical carcinoma were correlated with cancer inhibition. By reducing G6PD expression, miR-1 inhibited proliferation and promoted apoptosis in HR-HPV+ cervical cancer cells, and reduced the growth of tumor xenografts in nude mice. Together, these *in vitro* and *in vivo* results indicate that miR-1 might suppress the development and progression of HR-HPV-16/18+ cervical cancer by targeting G6PD. Therefore, miR-1 may serve as a novel therapeutic candidate in the treatment of HR-HPV 16/18-infected cervical cancer.

In combination with AGO proteins, single miRNA species have the potential to target thousands of different mRNAs, and single mRNAs may also be targeted by multiple miRNAs [13, 16, 34]. Complex principles govern the binding of metazoan miRNAs to mRNA targets, making it challenging to predict physiological interactions with particular mRNA targets [15]. Therefore, new high-throughput techniques are required to test current hypotheses and advance the understanding of the roles of miRNAs in cancer [15]. Recent reports have demonstrated that direct and rigorously-validated anti-AGO co-IP and downstream Affymetrix microarray analyses (RIP-Chip) can help guide computational algorithm development [13, 35]. Based on the TargetScan, miRanda, and Diana microT computational algorithms, we determined that miR-1, miR-133a, and miR-206 might target a combined site in the G6PD 3′-UTR gene sequence. We then used co-IP RIP-Chip to validate these predictions and found that miR-1 had the strongest interaction with G6PD. qRT-PCR revealed that decreased miR-1 expression and increased G6PD levels correlated with cancer development and malignant characteristics. miRNAs play a pivotal role in cancer progression and development, and may therefore serve as novel therapeutic tools for cancer therapy [31]. Dysregulation of miR-1 is involved in carcinogenic events in various cancers, including colon cancer [36, 37], hepatocellular carcinoma [38], and esophageal squamous cell carcinoma (ESCC) [39]. In this study, we demonstrated that G6PD is also a direct target of miR-1 in cervical cancer cells. miR-1 inhibited proliferation and promoted apoptosis in cervical cancer cells by down-regulating G6PD. These results indicate that the loss of miR-1-induced G6PD suppression may play a crucial role in pathogenic events in HR-HPV+ cervical cancer.

G6PD expression transforms NIH3T3 fibroblasts and induces tumor development in nude mice, indicating that G6PD acts as an oncogene [40]. Furthermore, G6PD expression is elevated in many kinds of tumors, including endometrial carcinomas and HR-HPV+ cervical cancer [5, 6, 7, 9, 10, 41]. In addition, silencing G6PD expression inhibited, and exogenous G6PD expression increased, proliferation in Rhabdomyosarcoma (RMS) cells, confirming that increased G6PD levels are associated with increases in cell growth [42]. In another pre-clinical study, treatment with adrenocortical steroid dehydroepiandrosterone (DHEA), a powerful inhibitor of G6PD, inhibited tumor development [25]. It is well known that cancer cells consume large quantities of glucose to support rapid growth and proliferation [43]. The reducing power of NADPH, which is produced primarily via the pentose phosphate pathway (PPP), is also required for biosynthesis in cancer cells [42]. G6PD catalyzes the rate-limiting step of the PPP, perhaps accounting for the increased G6PD levels found here and in many other cancers.

miRNAs modulate the expression of numerous metabolic factors and enzymes, such as SREBF2, AKT2, G6PD, CPS1, FADS1, and ETNK1, which alter tumor cell responses to chemotherapeutic treatments [44, 45]. Coda et al. showed that G6PD is strongly downregulated in RMS cells upon miR-206-induced differentiation, confirming that G6PD is a direct target of miR-206 [42]. These findings, together with our results, indicate that miR-1 inhibits proliferation and promotes apoptosis in cervical cancer both *in vitro* and *in vivo* by targeting G6PD, suggesting that G6PD-targeting treatments may provide a new strategy for cervical cancer therapy.

In conclusion, we demonstrated that: i) miR-1 bound to the 3′-UTR seed region of G6PD mRNA; ii) decreased miR-1 expression in HR-HPV 16/18-infected cervical carcinoma was correlated with carcinogenic development; iii) overexpression of miR-1 down-regulated G6PD, reduced proliferation, and promoted apoptosis in HR-HPV16/18+ cervical cancer cells; iv) co-transfection of both G6PD siRNA and miR-1 sponge partially reversed miR-1 sponge-induced promotions in cell viability and neoplasm growth. Taken together, these results indicate that miR-1 might suppress the development and progression of HR-HPV-16/18+ cervical cancer by directly targeting G6PD, and that miR-1 might therefore be a valuable novel therapeutic candidate.

## MATERIALS AND METHODS

### Ethical approval and informed consent

Animal use and care were in conducted in accordance with guidelines provided in the Guide for the Care and Use of Laboratory Animals published by the US National Institutes of Health (NIH Publication No. 85-23, revised 1996) and in the Care and Use Guidelines of Experimental Animals established by the Research Ethics Committee of Kunming University of China (Permit Number: kmu-eac-2015117). All surgical procedures were performed under chloral hydrate anaesthesia, and all efforts were made to minimize suffering.

This study also complied with the Ethical Declaration and was approved by the Human Ethics Committee and the Research Ethics Committee of Kunming University of China (Permit Number: kmu-hec-2014036). According to these guidelines, patients were informed that resected specimens would be kept by the Third People's Hospital of Yunnan Province and might be used for scientific research and that their privacy would be maintained. All of the patients who participated in this study provided informed consent.

### Preparation of cell lines

HPV-negative C33A (HPV-C33A), HPV18-positive Hela (HPV18+ Hela), and HPV16-positive Siha (HPV16+ Siha) human cervical carcinoma cells lines, as well as normal human cervical epithelial H8 cells, were used in this study. All cell lines were purchased from the Institute of Biochemistry and Cell Biology, Shanghai, China. Cells were maintained in Dulbecco's modified Eagle medium (DMEM, Gibco, Life Technologies, Carlsbad, CA) supplemented with 10% fetal calf serum (Invitrogen, USA) at 37°C in a humidified incubator with 5% CO2. All cells were cultured at 70–80% confluence.

### Cell treatments

To determine whether miR-1 contributed to carcinogenic events in HR-HPV-infected cervical cancer by targeting G6PD, miR-1 overexpression and/or miR-1 inhibition was established in cultured cervical cancer cells with or without G6PD overexpression. Proliferation and apoptosis were then examined in these cells using 3- (4, 5-Dimethylthiazol-2-yl)-2, 5- diphenyltetrazolium bromide (MTT) and flow cytometry (FCM) assays, respectively.

A lentiviral system was used for miR-1 overexpression. Hela and Siha cells were co-transfected with plenti-miR-1 along with the packaging plasmids psPAX2 and pMD2.G (Addgene), respectively (named Hela-plenti-miR-1 and Siha-plenti-miR-1). In control cells, plenti-miR-1 was replaced by control lemiR (named Hela-lemiR and Siha-lemiR). We simultaneously used miRNA sponge technology to inhibit miR-1 activity in cultured cervical cancer cells [24, 25]. Cultured Hela and Siha cells were transfected with pCMV-d2eGFP-miR-1 (destabilized eGFP with the miR-1 sponge in the 3′-UTR, named Hela/Siha-miR-1-sponge) or pCMV-d2eGFP-CXCR4 as a control (destabilized eGFP with the CXCR4 non-binding sponge sequence, named Hela/Siha-CX-control).

Additionally, Hela/Siha-plenti-miR-1 was co-transfected with plenti-G6PD using the lentiviral overexpression system described above (named Hela/Siha-plenti-miR-1 + plenti-G6PD). Hela/Siha-plenti-miR-1 co-transfected with lemiR served as a control (named Hela/Siha-plenti-miR-1 + G6PD control). Similarly, Hela/Siha-miR-1-sponge was co-transfected with G6PD-siRNA plasmid; co-transfection with Empty-siRNA served as a control (named Hela/Siha-miR-1-sponge + G6PD-siRNA and Hela/Siha-miR-1-sponge + Empty-siRNA, respectively).

### Bioinformatics predictions

To identify miRNAs that might target G6PD, the TargetScan, miRanda, and Diana microT computational algorithms were used [26]. Potential miRNAs were identified using the sequence of a combined G6PD 3′-UTR gene site. Among the many miRNAs identified, miR-1, miR-133a, and miR-206, each of which were predicted by all three software programs, were chosen for further validation.

### RIP-Chip procedure

We performed anti-AGO co-immunoprecipitation (co-IP) and downstream Affymetrix microarray analyses (“RIP-Chip”) to confirm that the computationally-predicted miRNAs targeted G6PD.

### Cell transfections

For RIP-Chip transfections, Hela and Siha cells were plated in 10-cm culture plates at a density of 2.5 × 10^6^/plate. After 24 hours, cells were transfected with 25 nM “Pre-miRNA” (Ambion) for has-miR-1, has-miR-133a, has-miR-206, or Negative Control (NC, Ambion, Austin, TX, sense sequence AGUACUGCUUACGAUACGG) using RNAiMAX (Invitrogen, Carlsbad, CA) according to the manufacturer's instructions [13]. Cells were cultured for 48 hours after transfection.

### Co-IP of miRNPs with anti-AGO antibodies

The RIP-Chip co-IP assay has previously been described in detail [13, 27]. Briefly, protein G-agarose beads (Invitrogen, Carlsbad, CA) were incubated with monoclonal anti-AGO or nonimmune mouse serum (NMS, Pierce Biotechnology, Rockford, IL). Cells were harvested 48 hours after transfection. Cell lysates were subjected to preclearance by incubation with pre-blocked protein G beads at 4°C for 60 minutes. Co-IP with either AGO-Protein G beads or NMS-protein G beads was then performed at 4°C for 90 minutes using lysate aliquots. After co-IP, the beads were washed at room temperature. Beads and lysates were then subjected to DNase treatment by shaking and incubating at 37°C for 20 minutes with 250 μL of DNA digestion solution. Co-IPed RNA and total RNA from lysates were then extracted using Trizol LS (Invitrogen, Carlsbad, CA) as described previously [28].

### Microarray analysis

Microarray analysis of RNAs isolated via co-IP was performed using an Affymetrix Human Gene 1.0 ST chip at the University of Kentucky Microarray Core Facility. Eight biological replicates from three individual experiments were performed for each transfection condition.

### Northern blot

Northern blot analyses were performed using RNA isolated from Hela and Siha cells 48 h after transfection with miRNAs as described previously [28].

### RNA preparation and miRNA microarray

After carefully rinsing in cooled PBS, cells were homogenized on ice in TRIzol (Invitrogen, Carlsbad, CA). Total RNA was isolated using TRIzol according to the manufacturer's instructions. RNA quality and quantity were measured using a Nanodrop spectrophotometer (ND-1000, Nanodrop Technologies), and RNA integrity was determined by gel electrophoresis.

To detect miRNAs, 100 ng of RNA was labeled and hybridized using the Human microRNA Microarray Kit (Rel. 12.0) (Agilent Technologies, CA, USA) according to the manufacturer's protocol. miR-1/133a/206 and control miRNAs were measured in eight biological replicates in three separate batches. Agilent miRNA microarrays (Version 1.0) were used. Hybridization signals were detected with an AgilentDNA G2505B microarray scanner, and scanned images were analyzed using Agilent feature extraction software (v10.10.1.1). All data were deposited in the NCBI Gene Expression Omnibus (GEO Series accession number GSE71953).

### miR-1:G6PD mRNA interaction

Wild-type (wt) and mutant (mut) human G6PD mRNA 3′-UTR seed regions, which included the potential target site for miR-1, were cloned. The cloned sequences were inserted downstream of the pGL3 luciferase reporter gene to generate the G6PD 3′-UTR-wt and G6PD 3′-UTR-mut vectors. Briefly, 293T cells were seeded in 96-well plates and co-transfected with 100 ng/mL of the individual pGL3-G6PD 3′-UTR-wt/mut vectors and 50 nM miR-1 mimics or NC (Ribobio, Guangzhou, China). Forty-eight hours after transfection, the effects of miR-1 treatment on luciferase activity were measured using a dual-luciferase reporter assay system kit (Promega) and a Tecan M200 luminescence reader (Tecan Group Ltd, Männedorf, Switzerland) according to the manufacturer's instructions. Values were double-normalized to firefly luciferase activity and to cells transfected with empty control vectors [29, 30].

### Patient population and clinical sample collection

Sixty cervical cancer patients infected with HPV who were treated at the Third People's Hospital of Yunnan Province between February and December 2014 participated in the study. All patients underwent operations at our hospital. Samples from three of the patients were necrotic and were unsuitable for analysis. The remaining fifty-seven samples were collected and cervical tumors were histopathologically confirmed. Matched normal adjacent tissues at least 1 cm distal to the tumor margins were also collected. Specimens were immediately stored at −80°C until nucleic acids and proteins were isolated.

### HPV detection and genotyping

Cervical cell samples were collected for HPV detection as described previously [5].

### Quantitative RT-PCR

miRNA was quantified using reverse transcription and quantitative real-time PCR (qRT-PCR). Small RNAs from cell and tissue samples were prepared using a TRIzol and miRNeasy mini kit (Qiagen, Valencia, USA) according to the manufacturer's instructions. cDNA was synthesized from total RNA using gene-specific primers according to the TaqMan MicroRNA Assay protocol provided by the manufacturer (Applied Biosystems). Quantitative PCR for miR-1/133a/206 was performed using an Applied Biosystems 7300 Sequence Detection system. The 10 μL PCR reaction contained 0.67 μL of reverse transcription product, 1 x TaqMan Universal PCR master mix, and 1 μL of primer and probe mix, according to the TaqMan MicroRNA Assay protocol (Applied Biosystems). Samples were normalized to snoRNA202 expression [31]. Relative gene expression was determined using the 2-delta delta CT (2^-ΔΔC^_T_) analysis method.

Cells and tumor samples were also obtained for G6PD detection as described previously [5]. The relative CT method was employed to compare differences between samples. Fold-decrease/increase was determined relative to a blank control after normalization to the housekeeping gene GAPDH using the 2^-ΔΔCT^ method.

### Western blots

G6PD protein expression was detected using Western blots in Hela and Siha cells transfected with miR-1 overexpression or sponge vectors according to a previously described protocol [5]. Briefly, cell samples were lysed on ice for 30 min in CytoBuster Protein Extraction Buffer (Novagen, USA), and 50 μg of protein was used for 10% sodium dodecyl sulfate polyacrylamide gel electrophoresis (SDS-PAGE). The protein was then transferred to a nitrocellulose (NC) membrane and sealed with Tris-Buffered Saline Tween-20 (TBST) containing 5% non-fat milk powder. The membrane was subsequently incubated with goat anti-human G6PD (1:500, Santa Cruz, sc-46971) and mouse anti-human GAPDH (1:500, Santa Cruz, sc-81545) antibodies at 4°C overnight. After washing in TBST, the membrane was incubated with horse radish peroxidase (HPR)-conjugated secondary antibodies (1:2000) at 25°C.

The mouse anti-AGO antibody used for IP was also used for western blots at a dilution of 1:1000; the secondary rabbit anti-mouse HPR-conjugated secondary antibody was used at a dilution of 1:1500. Protein was visualized and quantities determined using an electrochemiluminescence (ECL) technique (BestBio, USA). Photographs were taken using the JS Gel Imaging System (Peiqing, China), and gray densities were calculated using SensiAnsys software (Peiqing, China).

### MTT

Cell viability was determined using the tetrazolium salt MTT assay as described previously [5]. The optical density of each sample was measured using a microplate reader (BioRad, Hercules, CA, United States) at 490 nm.

### FCM

An annexin V-FITC-flow cytometry assay kit (4A Biotech Co. Ltd.) was used to detect cellular apoptosis rates after G6PD-siRNA plasmid transfection as previously described [32].

### Cervical cancer xenograft nude mouse model

A total of 85 BALB/c strain nude mice (4–5 weeks, 18–20 g, Beijing HFK Bioscience Co, Ltd, Beijing, China; animal license number SCXK 2014–00053, Beijing) were housed and raised in the laboratory animal center of the Affiliated Cancer Hospital of Sun Yat-sen University. Animal use and treatment was approved by the Animal Ethics Committee of Sun Yat-sen University.

Five mice were randomly assigned to each of the following 17 groups: normal human cervical epithelial H8 cells (H8)-treated group; miR-1 overexpression cervical cancer cell (Hela/Siha-plenti-miR-1)-treated groups; miR-1 deficient human cervical cancer cell (Hela/Siha-miR-1-sponge)-treated groups; matched control groups (Hela/Siha-lemiR and Hela/Siha-CX-control); G6PD rescue groups (Hela/Siha-plenti-miR-1 + plenti-G6PD, Hela/Siha-plenti-miR-1 + G6PD control); and G6PD inhibition groups (Hela/Siha-miR-1-sponge + G6PD-siRNA, and Hela/Siha-miR-1-sponge + Empty-siRNA). Cells in the log-phase growth stage were harvested and digested to obtain isolated cells using 0.25% pancreatin. Cells were washed with Dulbecco's Modified Eagle Medium (DMEM) without serum. A volume of 1 mL of each cell type (1.5 × 10^6^/mL) was injected intradermally into the left axilla of the mice. After seeding, liquid absorption at the injection site, tumor growth (volume and weight), and mouse survival were measured. Tumor volume was measured on days 5, 7, 12, 16, 19, 21, 25, and 27 post-injection. On day 27, all mice were sacrificed, tumors were isolated, and tumor weight and volume were determined. The largest (a) and smallest diameters (b) of each tumor were measured twice on days 5, 7, 12, 16, 19, 21, 25, and 27 to estimate tumor volume (V) using the formula V = 0.52 × a^2^ × b [6]. Mean tumor volumes were used to plot tumor growth curves for each group of mice.

### Statistical analysis

Values are presented as means ± SE. Between-group differences were evaluated using repeated-measure ANOVAs. Multivariate logistic regression analysis was performed to evaluate associations between HR-HPV infection status (HPV 16/18-positive or -negative), miRNA expression, and cervical cancer clinicopathologic characteristics, including clinical stage (FIGO stage, I-IV), cell grade (well-, moderately-, or poorly-differentiated), and tumor diameter (≤ 4 or > 4 cm). The strength of associations was determined using odds ratios (ORs) with 95% confidence intervals (CI); logistic regression was used to estimate adjusted ORs for ordinal data. *P* < 0.05 was considered statistically significant. All analyses were conducted using SPSS version 19.0 software.

## SUPPLEMENTARY TABLE





## ABBREVIATIONS

G6PD: Glucose-6-phosphate dehydrogenase
HR-HPV: high-risk human papillomaviruses
PPP: pentose phosphate pathway
AGO: Argonaute
microRNAs: miRNAs
miRNPs: microribonucleoparticles
RISC: RNA induced silencing complex
