# Supplementary material for: miR-1 inhibits progression of high-risk papillomavirus-associated human cervical cancer by targeting G6PD
Source: Oncotarget. 2016 Nov 3;7(52):86103–16. doi: 10.18632/oncotarget.13344 (PMC5349900; doi:10.18632/oncotarget.13344)
Supplement: Supplementary file 1 [file oncotarget-07-86103-s001.pdf]

miR-1 inhibits progression of high-risk papillomavirus-associated human cervical cancer by targeting G6PD

Supplementary Materials

Supplementary Table S1: Enriched mRNAs following miR-1 transfections.

See Supplementary_Table_S1
