# Supplementary material for: miR-1 inhibits progression of high-risk papillomavirus-associated human cervical cancer by targeting G6PD
Source: Oncotarget. 2016 Nov 3;7(52):86103–16. doi: 10.18632/oncotarget.13344 (PMC5349900; doi:10.18632/oncotarget.13344)
Supplement: Supplementary file 2 [file oncotarget-07-86103-s002.docx]

**Supplemental data**

**Table Enriched mRNAs following miR-1 transfections**

| **Accession** | **Ortholog of target gene** | **Fold-changes enrichment after miRNA transfection relative to negative control miRNA transfection** | **Gene name** |
| --- | --- | --- | --- |
| NG_009015 | G6PD | 56.4 | glucose-6-phosphate dehydrogenase |
| NC_000001 | POGK | 31.1 | pogo transposable element with KRAB domain |
| NM_001272077.1 | RIT2 | 29.4 | Ras-like without CAAX 2 |
| AB094663 | P2rx2 | 26.5 | P2X2e purinergic receptor, complete cds. |
| S53716 | Tec | 24 | tyrosine kinase |
| X72307 | Hgf | 23.8 | hepatocyte growth factor. |
| AB055004 | Slc38a4 | 22.1 | system A amino acid transporter 3, complete cds. |
| BC156383 | Cep63 | 21.7 | centrosomal protein 63 (Cep63) mRNA, encodes complete protein. |
| U65593 | Kcnab3 | 20.8 | K+ channel beta4 subunit mRNA, complete cds. |
| U75840 | Irf3 | 19.6 | alt 'C920001K05Rik#IRF-3#MGC91046' |
| BC156418 | Gpr116 | 19.2 | G protein-coupled receptor 116 (Gpr116) mRNA, encodes complete protein. |
| XM_001474722 | Gm2788 | 19.1 | hypothetical protein LOC100040465 (LOC100040465), mRNA. |
| AK004863 | Adh6-ps1 | 18.9 | ALCOHOL DEHYDROGENASE 2 (EC 1.1.1.1) [Peromyscus maniculatus], full insert sequence. |
| AK004201 | Nme6 | 18.7 | expressed in non-metastatic cells 6, protein, full insert sequence. |
| XM_001480986 | Gm4651 | 18.4 | hypothetical protein LOC100043793 (LOC100043793), mRNA. |
| NG_032138 | SRI | 18.2 | sorcin |
| NG_016361.1 | CDR1 | 18.2 | cerebellar degeneration-related protein 1, 34kDa |
| AJ132601 | Nr1h3 | 18.2 | nuclear oxysterol receptor LXR-alpha (LXR-alpha). |
| X82561 | Ptpn3 | 17.9 | protein tyrosine phosphatase. |
| NM_001319068.1 | ITGB1BP1 | 17.3 | integrin beta 1 binding protein 1 |
| NG_034190.1 | VPS53 | 16.8 | vacuolar protein sorting 53 homolog (S. cerevisiae) |
| XM_001472048 | Gm12711 | 16.8 | hypothetical protein LOC100038976 (LOC100038976), mRNA. |
| U20366 | Hoxa11as | 16.4 | Hoxa11 locus antisense-23A mRNA. |
| AK002574 | Serpina1f | 15.9 | hypothetical Serpins containing protein, full insert sequence. |
| AK004821 | Pvrl4 | 15.8 | NECTIN 4 homolog [Homo sapiens], full insert sequence. |
| AK002694 | Hpn | 14.8 | hepsin, full insert sequence. |
| AK003367 | Mrpl15 | 14.8 | mitochondrial ribosomal protein L15, full insert sequence. |
| NR_003559 | Mrpl48 | 14.6 | mitochondrial ribosomal protein L48 (Mrpl48), transcribed RNA. |
| U69888 | Ipw | 14.3 | Ipw mRNA, partial sequence. |
| AJ011416 | Fcgbp | 14.2 | IgG Fc binding protein, partial. |
| AJ242954 | N/A | 13.9 | partial mRNA for dysferlin (dysf gene). |
| AK006024 | 1700110I01Rik | 13.9 | unclassifiable, full insert sequence. |
| U56649 | Pde1a | 13.7 | cyclic nucleotide phosphodiesterase (PDE1A2) mRNA, complete cds. |
| XM_001471840 | Hjurp | 13.6 | hypothetical protein LOC100038822 (LOC100038822), mRNA. |
| XM_001474012 | N/A | 13.4 | hypothetical protein LOC100040058 (LOC100040058), mRNA. |
| U59230 | Nell2 | 12.6 | mel (MEL91) mRNA, complete cds. |
| XM_001004878 | Gm14490 | 12 | DNA cytosine-5 methyltransferase 3B2 (LOC668932), mRNA. |
| X97581 | Sall3 | 11.9 | alt 'B130022O04Rik#Msal#Msal-1#Sal#Salt#Spalt' |
| AK003315 | Myeov2 | 11.9 | unclassifiable, full insert sequence. |
| M13680 | Igh-6 | 11.4 | Ig active mu-chain mutant C-region mRNA, exons 3 and 4, from mutant 102 derived from hybridoma PC7. |
| X04482 | Igf1 | 11.3 | preproinsulin-like growth factor IB. |
| NG_011713 | IGF1 | 11.2 | insulin-like growth factor 1 (somatomedin C) |
| BC153031 | Cdk3 | 11.2 | cyclin-dependent kinase 3 (Cdk3) mRNA, encodes complete protein. |
| XM_001474669 | 1110021L09Rik | 11.2 | RIKEN cDNA 1110021L09 gene (1110021L09Rik), mRNA. |
| BC156421 | Ttc37 | 11 | cDNA sequence AK129128 (AK129128) mRNA, encodes complete protein. |
| NR_003518 | Pisd-ps3 | 11 | RIKEN cDNA 4933439C20 gene (4933439C20Rik) on chromosome Y. |
| XM_001480879 | BC030499 | 11 | hypothetical protein LOC100043544, transcript variant 1 (LOC100043544), mRNA. |
| BC006783 | Ctgf | 10.92 | connective tissue growth factor, mRNA (cDNA clone MGC:8122 IMAGE:3589136), complete cds. |
| BC018188 | Ergic2 | 10.9 | ERGIC and golgi 2, mRNA (cDNA clone MGC:25708 IMAGE:3711066), complete cds. |
| NM_001110513 | Ebf4 | 10.9 | early B-cell factor 4 (Ebf4), mRNA. |
| AK005712 | 4933406F09Rik | 10.8 | unclassifiable, full insert sequence. |
| BC046819 | Asb4 | 10.6 | ankyrin repeat and SOCS box-containing protein 4, mRNA (cDNA clone MGC:61185 IMAGE:5703017), complete cds. |
| XM_001480783 | Fam160b2 | 10.6 | retinoic acid induced 16 (Rai16), mRNA. |
| AK003543 | 5033414D02Rik | 10.6 | UNCHARACTERIZED HEMATOPOIETIC STEM/PROGENITOR CELLS PROTEIN MDS030 [Homo sapiens], full insert sequence. |
| L13171 | Mef2c | 10.4 | myocyte-specific enhancer factor 2 (MEF-2C) mRNA sequence. |
| XM_001480003 | Gm4379 | 10.4 | reproductive homeobox on X chromosome 8 (LOC100043350), mRNA. |
| AF074973 | Oprm1 | 10.4 | alt 'MOP-R#MOR-1#MOR-1O#Oprm#mor#muOR' |
| XM_994286 | Parp4 | 10.3 | poly (ADP-ribose) polymerase family, member 4 (Parp4), mRNA. |
| AF527954 | Bre | 10.3 | brain and reproductive organ-expressed isoform II3+ (Bre) mRNA, complete cds |
| AJ006140 | Matn4 | 10.3 | matrilin-4. |
| BC156370 | Rxfp1 | 10.2 | relaxin/insulin-like family peptide receptor 1 (Rxfp1) mRNA, encodes complete protein. |
| XM_001476840 | Robo3 | 10.2 | roundabout homolog 3 (Drosophila) (Robo3), mRNA. |
| AF125671 | Ncor2 | 9.7 | silencing mediator of retinoic acid and thyroid hormone receptor extended isoform (Smrte) mRNA, complete cds. |
| AK002723 | 0610031G08Rik | 9.7 | hypothetical protein, full insert sequence. |
| AB196497 | Nfkbiz | 9.6 | IkappaB-zeta mRNA for IkappaB-zeta(D), partial cds. |
| AK004674 | 1200009I06Rik | 9.6 | RIKEN cDNA 1200009I06 gene, full insert sequence. |
| XM_001481153 | Gm4706 | 9.5 | hypothetical protein LOC100043878 (LOC100043878), mRNA. |
| AF304376 | Rab27a | 9.5 | RAB27A (Rab27a) mRNA, complete cds. |
| AK003139 | 1810013D10Rik | 9.5 | RIKEN cDNA 1810013D10 gene, full insert sequence. |
| AK003308 | Chchd7 | 9.5 | RIKEN cDNA 1810049H20 gene, full insert sequence. |
| AK005131 | Mrps15 | 9.5 | Mus musculus adult male cerebellum cDNA, RIKEN full-length enriched library, clone:1500003E2mitochondrial ribosomal protein S15, full insert sequence. |
| NM_001111030 | Acvr1c | 9.4 | activin A receptor, type IC (Acvr1c), transcript variant 1, mRNA. |
| XM_001479143 | Gm4124 | 9.4 | UDP-glucuronate decarboxylase 1 (LOC100042952), mRNA. |
| XM_893730 | Gm6934 | 9.4 | predicted gene, EG628919 (EG628919), mRNA. |
| AB037541 | Cyp4f14 | 9.4 | Mus musculus CYP4F14 mRNA for leukotriene B4 omega-hydroxylase, complete cds. |
| AJ224740 | Cradd | 9.3 | death adaptor molecule (Raidd/Cradd), I.M.A.G.E clone 407755,. |
| BC152909 | Hnrnpul2 | 9.3 | heterogeneous nuclear ribonucleoprotein U-like 2 (Hnrpul2) mRNA, encodes complete protein. |
| XM_001472292 | Gm6804 | 9.3 | hypothetical LOC627881 (LOC627881), mRNA. |
| XM_001473424 | LOC100039744 | 9.3 | Nid1 protein (LOC100039744), mRNA. |
| XM_001475503 | Gm10362 | 9.3 | Rpl17 protein (LOC100040929), mRNA. |
| XM_990203 | Myo18b | 9.3 | myosin XVIIIb (Myo18b), mRNA. |
| AB001539 | Grwd1 | 9.3 | A301 protein, complete cds. |
| AF302503 | Peli1 | 9.3 | pellino 1 (Peli1) mRNA, complete cds. |
| BC156345 | Ttll5 | 9.2 | tubulin tyrosine ligase-like family, member 5 (Ttll5) mRNA, encodes complete protein. |
| XM_001477646 | Gm9790 | 9.2 | hypoxia induced gene 1, transcript variant 2 (LOC100042265), mRNA. |
| AF247161 | Gtf2ird1 | 9.2 | muscle TFII-I repeat domain-containing protein 1 beta 1 mRNA, complete cds. |
| AF479018 | Gm6273 | 9.2 | clone hybridoma 22b TCR V beta chain mRNA, partial cds. |
| AJ001261 | Gbas | 9.2 | NIPSNAP2 protein. |
| NR_002897 | Snora64 | 9.1 | small nucleolar RNA, H/ACA box 64 (Snora64) on chromosome 17. |
| XM_001479576 | Zscan4-ps2 | 9.1 | zinc finger and SCAN domain containing 4, pseudogene 2, transcript variant 1 (Zscan4-ps2), mRNA. |
| XM_979611 | 1700020N18Rik | 9.1 | RIKEN cDNA 1700020N18 gene (1700020N18Rik), mRNA. |
| AK005885 | Tmem53 | 8.9 | alpha/beta-Hydrolases structure containing protein, full insert sequence. |
| AK005758 | Lrrc51 | 8.7 | hypothetical Leucine-rich repeat containing protein, full insert sequence. |
| NM_001111029 | Zfp661 | 8.6 | zinc finger protein 661 (Zfp661), transcript variant 2, mRNA. |
| AK003877 | Vps25 | 8.6 | hypothetical Anticodon-binding domain of Class II aaRS structure containing protein, full insert sequence. |
| AK005168 | Cmtm5 | 8.6 | hypothetical protein, full insert sequence. |
| AJ490822 | Sspo | 8.5 | SCO-spondin (sco-spondin gene). |
| AK002569 | 2610110G12Rik | 8.5 | 0610011P08RIK PROTEIN, full insert sequence. |
| AK005333 | Pisd-ps3 | 8.5 | unclassifiable, full insert sequence. |
| AK005738 | 1700012B15Rik | 8.5 | hypothetical protein, full insert sequence. |
| M21041 | Mtap2 | 8.4 | microtubule-associated protein 2 (MAP2) mRNA, complete cds. |
| XM_620426 | Gm5889 | 8.4 | predicted gene, EG545921, transcript variant 1 (EG545921), mRNA. |
| XM_895007 | Gm11709 | 8.4 | CLM3 (LOC629970), mRNA. |
| AB004048 | Nnat | 8.4 | neuronatin, complete cds. |
| AK003411 | Sec11c | 8.4 | MICROSOMAL SIGNAL PEPTIDASE 21 KDA SUBUNIT (EC 3.4.-.-) (SPASE 21 KDA SUBUNIT) (SPC21) homolog [Rattus norvegicus], full insert sequence. |
| XM_001477842 | Gm3579 | 8.3 | hypothetical protein LOC100041932 (LOC100041932), mRNA. |
| XM_148801 | Prr22 | 8.3 | gene model 546, (NCBI) (Gm546), mRNA. |
| AF054842 | Insl5 | 8.3 | relaxin/insulin-like factor variant a mRNA, complete cds. |
| AF465243 | Hax1 | 8.3 | HAX1XS mRNA, complete cds. |
| AK005418 | 1600010M07Rik | 8.3 | unclassifiable, full insert sequence. |
| BC156260 | Hecw1 | 8.2 | HECT, C2 and WW domain containing E3 ubiquitin protein ligase 1 (Hecw1) mRNA, encodes complete protein. |
| NM_001098230 | Pdp1 | 8.1 | protein phosphatase 2C, magnesium dependent, catalytic subunit (Ppm2c), transcript variant 2, mRNA. |
| NM_001083967 | Tcf4 | 7.9 | transcription factor 4 (Tcf4), transcript variant 2, mRNA. |
| XM_001480223 | Gm9690 | 7.9 | U2-associated SR140 protein (LOC676758), mRNA. |
| AF004109 | Aanat | 7.9 | rylalkylamine N-acetyltransferase mRNA, complete cds. |
| AK002544 | Dolpp1 | 7.9 | LSFR2 PROTEIN (FRAGMENT) homolog [Fugu rubripes], full insert sequence. |
| AK004512 | 1810058I24Rik | 7.9 | unclassifiable, full insert sequence. |
| XM_001478601 | Gm3952 | 7.8 | Stretchin-Mlck CG18255-PD (LOC100042659), mRNA. |
| AK005538 | 1700123M08Rik | 7.8 | unclassifiable, full insert sequence. |
| M35246 | H2-T10 | 7.5 | MHC class I H2-TL-T10-b mRNA (b haplotype), complete cds. |
| AK005453 | Prrg2 | 7.5 | proline-rich Gla (G-carboxyglutamic acid) polypeptide 2, full insert sequence. |
| AF012151 | N/A | 7.4 | Mus musculus |
| AF020681 | Runx2 | 7.4 | core binding factor alpha1 subunit isoform (Cbfa-1) mRNA, partial cds. |
| AK005368 | Ntsr2 | 7.4 | LOW AFFINITY NEUROTENSIN RECEPTOR, full insert sequence. |
| NM_001111331 | Kcnip3 | 7.3 | Kv channel interacting protein 3, calsenilin (Kcnip3), transcript variant 2, mRNA. |
| NM_173444 | Nbeal1 | 7.3 | neurobeachin like 1 (Nbeal1), mRNA. |
| AB093278 | Ttll5 | 7.3 | mKIAA0998 protein. |
| AF399971 | Spata3 | 7.3 | MTSARG1 (Tsarg1) mRNA, complete cds. |
| XM_001475360 | Gm3014 | 7.2 | CG2839-PA (LOC100040872), mRNA. |
| XM_001472963 | Gm2260 | 6.9 | Annexin A11 (LOC100039484), mRNA. |
| AB259692 | Rnf125 | 6.9 | rnf125 mRNA for RNF125 protein, complete cds. |
| AF188008 | Plec1 | 6.9 | plectin isoform plec 1,2alpha (Plec1) mRNA, partial cds. |
| AK002372 | 1110038B12Rik | 6.9 | unclassifiable, full insert sequence. |
| AK005214 | N/A | 6.9 | atonal homolog 7 (Drosophila), full insert sequence. |
| AK005520 | Gm13306 | 6.9 | small inducible cytokine A27, full insert sequence. |
| AF071506 | Timeless | 6.7 | TIMELESS protein mRNA, complete cds. |
| AK004060 | Ubac1 | 6.7 | PUTATIVE GLIALBLASTOMA CELL DIFFERENTIATION-RELATED PROTEIN [Homo sapiens], full insert sequence. |
| AF076623 | Nes | 6.6 | intermediate filament protein nestin (Nes) mRNA, complete cds. |
| AJ621558 | Arap1 | 6.6 | ARAP1 (Centd2 gene). |
| AK004157 | Atp6v1c2 | 6.6 | ATPase, H+ transporting, V1 subunit C, isoform 2, full insert sequence. |
| NM_010399 | H2-T9 | 6.5 | histocompatibility 2, T region locus 9 (H2-T9), mRNA. |
| AB008928 | Klk6 | 6.5 | neurosin, complete cds. |
| NM_001111059 | Cd34 | 6.4 | CD34 antigen (Cd34), transcript variant 1, mRNA. |
| XM_619430 | 4930488N24Rik | 6.4 | RIKEN cDNA 4930488N24 gene (4930488N24Rik), mRNA. |
| AK003210 | Ptpmt1 | 6.4 | hypothetical Tyrosine specific protein phosphatase and dual specificity protein phosphatase family containing protein, full insert sequence. |
| AK003614 | Elof1 | 6.4 | hypothetical protein, full insert sequence. |
| AK004338 | Etnk1 | 6.4 | ethanolamine kinase 1, full insert sequence. |
| AF348509 | Pmf1 | 6.3 | polyamine modulated factor-1S mRNA, complete cds. |
| AJ242911 | Nagk | 6.3 | non coding mRNA for N-acetylglucosamine kinase splice variant. |
| AK002446 | Dad1 | 6.3 | defender against cell death 1, full insert sequence. |
| XM_001473139 | Gm2422 | 6.2 | hypothetical protein LOC100039780 (LOC100039780), mRNA. |
| XM_974871 | Gm7409 | 6.2 | predicted gene, EG664925 (EG664925), mRNA. |
| AK005295 | Sfrs13a | 6.2 | FUS interacting protein (serine-arginine rich) 1, full insert sequence. |
| NM_172505 | A730008H23Rik | 6.1 | RIKEN cDNA A730008H23 gene (A730008H23Rik), mRNA. |
| XM_001473370 | 1700066J24Rik | 6.1 | RIKEN cDNA 1700066J24 gene (1700066J24Rik), mRNA. |
| XM_001475076 | Gm2905 | 6.1 | retinitis pigmentosa GTPase regulator interacting protein 1 (LOC100040685), mRNA. |
| XM_001478449 | Gm3908 | 6.1 | development and differentiation enhancing factor 2 (LOC100042573), mRNA. |
| XM_001479413 | 9530077C14Rik | 6.1 | RIKEN cDNA 9530077C14 gene (9530077C14Rik), mRNA. |
| NM_001110218 | Ppm1h | 6 | protein phosphatase 1H (PP2C domain containing) (Ppm1h), transcript variant 1, mRNA. |
| AK003195 | 2610528E23Rik | 6 | unclassifiable, full insert sequence. |
| NR_003519 | Pisd-ps2 | 5.9 | RIKEN cDNA D030013I16 gene (D030013I16Rik) on chromosome 17. |
| XM_144076 | Lrrc38 | 5.9 | leucine rich repeat containing 38 (Lrrc38), mRNA. |
| AF400247 | Oprm1 | 5.9 | alt 'MOP-R#MOR-1#MOR-1O#Oprm#mor#muOR' |
| AK002926 | Klf13 | 5.9 | Kruppel-like factor 13, full insert sequence. |
| AK003209 | Phf14 | 5.9 | hypothetical PHD-finger containing protein, full insert sequence. |
| AK005166 | Ip6k2 | 5.9 | SIMILAR TO MAMMALIAN INOSITOL HEXAKISPHOSPHATE KINASE 2 homolog [Homo sapiens], full insert sequence. |
| AK005256 | Snrpa1 | 5.9 | small nuclear ribonucleoprotein polypeptide A', full insert sequence. |
| AK005481 | LOC628147 | 5.9 | KRUPPEL-RELATED ZINC FINGER PROTEIN F80-L [Mus musculus], full insert sequence. |
| AK003136 | 1010001N08Rik | 5.8 | unclassifiable, full insert sequence. |
| AK004252 | 1110054M08Rik | 5.8 | unclassifiable, full insert sequence. |
| AK004941 | Spr | 5.8 | sepiapterin reductase, full insert sequence. |
| AK005987 | 1700015C17Rik | 5.8 | unclassifiable, full insert sequence. |
| XM_001472593 | Gm11213 | 5.7 | hypothetical LOC670833 (LOC670833), mRNA. |
| AK003291 | Tomm6 | 5.7 | OVER-EXPRESSED BREAST TUMOR PROTEIN homolog [Homo sapiens], full insert sequence. |
| AK003573 | Snhg6 | 5.6 | unclassifiable, full insert sequence. |
| D50311 | N/A | 5.4 | MEF2B, complete cds. |
| AB092414 | Cadm1 | 5.4 | a secretion form of SgIGSF/TSLC1, complete cds. |
| AF123611 | N/A | 5.4 | phosphatidic acid phosphatase type 2c (Ppap2c) mRNA, complete cds. |
| AK004638 | Pde3a | 5.4 | phosphodiesterase 3A, cGMP inhibited, full insert sequence. |
| BC156459 | Mdga1 | 5.3 | MAM domain containing glycosylphosphatidylinositol anchor 1 (Mdga1) mRNA, encodes complete protein. |
| XM_888290 | Mast3 | 5.3 | microtubule associated serine/threonine kinase 3, transcript variant 2 (Mast3), mRNA. |
| XM_972614 | Gm7339 | 5.3 | hypothetical LOC664788 (LOC664788), mRNA. |
| Y07693 | Nfic | 5.3 | NfiC1B-protein, splice variant. |
| AB221641 | Rhox4d | 5.3 | cDNA pooled tissues:(tissue_type=brain,dev_stage=8-12 days neonate,strain=BALB/c),(tissue_type=testis,dev_stage=adult, strain=C57BL/6J), clone:V01Y038I07. |
| AF033195 | Rdh5 | 5.3 | IMAGE 776350 9-cis-retinol dehydrogenase mRNA, complete cds. |
| AF305087 | Ehbp1l1 | 5.3 | alt 'G430002G23Rik' |
| AK006051 | 1700016P03Rik | 5.3 | unclassifiable, full insert sequence. |
| BC152876 | Rspo2 | 5.2 | R-spondin 2 homolog (Xenopus laevis) (Rspo2) mRNA, encodes complete protein. |
| NM_001110202 | Trim9 | 5.2 | tripartite motif protein 9 (Trim9), transcript variant 2, mRNA. |
| XM_001473395 | Gm2486 | 5.2 | hypothetical protein LOC100039903 (LOC100039903), mRNA. |
| XM_891521 | 4930447A16Rik | 5.2 | RIKEN cDNA 4930447A16 gene (4930447A16Rik), mRNA. |
| XM_485253 | 1110032F04Rik | 4.9 | RIKEN cDNA 1110032F04 gene (1110032F04Rik), mRNA. |
| AF223414 | Asph | 4.9 | junctin-2 mRNA, complete cds. |
| AF464161 | N/A | 4.9 | protocadherin gamma C-II precursor RNA, partial sequence. |
| AJ276961 | Clasp2 | 4.9 | alt '1500004F14Rik#8030404L10Rik#C77448#CLASP2beta#mKIAA0627' |
| AK003286 | Ict1 | 4.9 | immature colon carcinoma transcript 1, full insert sequence. |
| AK005461 | Tbc1d5 | 4.9 | hypothetical RabGAP/TBC domain containing protein, full insert sequence. |
| AK005591 | 1700001G11Rik | 4.9 | hypothetical protein, full insert sequence. |
| AK005753 | Ccl27a | 4.9 | unclassifiable, full insert sequence. |
| AK005904 | Rshl2a | 4.9 | RIKEN cDNA 1700012G05 gene, full insert sequence. |
| AK006011 | Speer4c | 4.9 | 4933431D05RIK PROTEIN (FRAGMENT) homolog [Mus musculus], full insert sequence. |
| AK006186 | 4930556L07Rik | 4.9 | unclassifiable, full insert sequence. |
| NM_001099635 | Myh3 | 4.8 | myosin, heavy polypeptide 3, skeletal muscle, embryonic (Myh3), mRNA. |
| XM_001474409 | Gm9717 | 4.8 | MAS-related GPR, member A4 (LOC677457), mRNA. |
| AB031037 | Eomes | 4.8 | Tbr2, complete cds. |
| AB085837 | Emid2 | 4.8 | type XXVI collagen, complete cds. |
| AF021031 | Dgcr6 | 4.8 | gcr6 protein (Dgcr6) mRNA, partial cds. |
| AF082803 | Cd244 | 4.8 | NK cell receptor 2B4 splice variant mRNA, complete cds. |
| AK004668 | Tnfrsf13b | 4.8 | tumor necrosis factor receptor superfamily, member 13b TACI protein, full insert sequence. |
| AK005420 | Npm1 | 4.8 | nucleophosmin 1, full insert sequence. |
| NM_001110504 | Capn1 | 4.7 | calpain 1 (Capn1), transcript variant 2, mRNA. |
| AF247674 | Tacc3 | 4.7 | ERIC1 (Eric1) mRNA, complete cds. |
| AF296283 | Icam4 | 4.7 | ICAM-4S mRNA, complete cds. |
| AK005137 | Mkrn1 | 4.7 | makorin, ring finger protein, 1, full insert sequence. |
| NM_001109753 | Sv2b | 4.6 | synaptic vesicle glycoprotein 2 b (Sv2b), transcript variant 2, mRNA. |
| XM_132047 | 3110047P20Rik | 4.6 | RIKEN cDNA 3110047P20 gene (3110047P20Rik), mRNA. |
| AF439557 | Rwdd3 | 4.6 | alt '3110037C01Rik#AI035684#AI662458#MGC150017#X2CR1' |
| AK003377 | Slc35b2 | 4.6 | hypothetical protein, full insert sequence. |
| AK004030 | N/A | 4.6 | synaptogyrin 2, full insert sequence. |
| XM_981889 | Col22a1 | 4.4 | collagen, type XXII, alpha 1, transcript variant 2 (Col22a1), mRNA. |
| NM_029896 | Wdr82 | 4.3 | WD repeat domain containing 82 (Wdr82), mRNA. |
| XM_001474127 | Pisd-ps2 | 4.3 | hypothetical protein LOC100040128 (LOC100040128), mRNA. |
| XM_127466 | 3110006E14Rik | 4.3 | RIKEN cDNA 3110006E14 gene (3110006E14Rik), mRNA. |
| AF011426 | Vmn2r10 | 4.3 | putative pheromone receptor (VR16) mRNA, complete cds. |
| AK002300 | Alad | 4.3 | aminolevulinate, delta-, dehydratase, full insert sequence. |
| AK005202 | Ppih | 4.3 | U-SNRNP-ASSOCIATED CYCLOPHILIN (EC 5.2.1.8) homolog [Homo sapiens], full insert sequence. |
| NG_029691.1 | NXT2 | 4.2 | nuclear transport factor 2-like export factor 2 |
| BC156134 | Pax5 | 4.2 | paired box gene 5 (Pax5) mRNA, encodes complete protein. |
| XM_001478683 | 2310046A06Rik | 4.2 | RIKEN cDNA 2310046A06 gene (2310046A06Rik), mRNA. |
| AF497641 | Gtf2ird1 | 4.1 | TFII-I repeat domain-containing protein 3 beta 3 mRNA, complete cds. |
| AK005239 | Cdan1 | 4 | congenital dyserythropoietic anemia, type I (human), full insert sequence. |
| AK005305 | 1300002E11Rik | 4 | unclassifiable, full insert sequence. |
| L12367 | Cap1 | 3.9 | adenylyl cyclase-associated protein (CAP) mRNA, complete cds. |
| XM_001476722 | Gm3168 | 3.9 | hypothetical protein LOC100041156 (LOC100041156), mRNA. |
| AF118847 | BC018473 | 3.9 | Hitchhiker locus mRNA, complete sequence. |
| AF257304 | Syt2 | 3.9 | synaptotagmin II (Syt2) mRNA, complete cds. |
| AF287467 | Sla2 | 3.9 | Src-like adaptor protein-2 mRNA, complete cds. |
| AK003160 | Uqcrq | 3.9 | ubiquinol-cytochrome c reductase binding protein, full insert sequence. |
| AK003504 | Fxyd1 | 3.9 | FXYD domain-containing ion transport regulator 1, full insert sequence. |
| AK003571 | Ppfia4 | 3.9 | protein tyrosine phosphatase, receptor type, f polypeptide (PTPRF), interacting protein (liprin), alpha 4, full insert sequence. |
| AK004191 | Tctex1d2 | 3.9 | hypothetical protein, full insert sequence. |
| AK004207 | 1110049B09Rik | 3.9 | hypothetical protein, full insert sequence. |
| AK005363 | 1500035N22Rik | 3.9 | hypothetical protein, full insert sequence. |
| AK005807 | 4930588J15Rik | 3.9 | unclassifiable, full insert sequence. |
| XM_001474539 | 2810416G20Rik | 3.8 | splicing coactivator subunit SRm300 (LOC100040353), mRNA. |
| XM_989755 | 9130024F11Rik | 3.8 | predicted gene, EG329160 (EG329160), mRNA. |
| AB093248 | Greb1 | 3.8 | mKIAA0575 protein. |
| AB101633 | N/A | 3.8 | abnormal Notch1 cDNA, clone:425-1U. |
| AF545043 | Ffar2 | 3.8 | orphan GPCR protein (Lssig) mRNA, complete cds. |
| AK003688 | Dpy30 | 3.8 | DPY-30-LIKE PROTEIN homolog [Mus musculus], full insert sequence. |
| AK004654 | Syde1 | 3.8 | hypothetical RhoGAP domain containing protein, full insert sequence. |
| AK006111 | Nme5 | 3.8 | NUCLEOSIDE DIPHOSPHATE KINASE HOMOLOG 5 (NDK-H 5) (NDP KINASE HOMOLOG 5) , full insert sequence. |
| D37837 | Lcp1 | 3.7 | 65-kDa macrophage cytosolic protein, complete cds. |
| NM_001098237 | Zbtb3 | 3.7 | zinc finger and BTB domain containing 3 (Zbtb3), transcript variant 1, mRNA. |
| NM_001110780 | Syn1 | 3.7 | synapsin I (Syn1), transcript variant b, mRNA. |
| AB015206 | Klk6 | 3.7 | serine protease (BSP), complete cds. |
| AF536772 | Robo4 | 3.7 | roundabout-like protein ROBO4 mRNA, complete cds. |
| AK003103 | 1010001B22Rik | 3.7 | unclassifiable, full insert sequence. |
| AK004057 | N/A | 3.7 | U6 SNRNA-ASSOCIATED SM-LIKE PROTEIN LSM7 [Homo sapiens], full insert sequence. |
| AB029144 | Psmd4 | 3.6 | pUb-R3, complete cds. |
| AB083210 | Zap70 | 3.6 | runcated ZAP kinase, complete cds. |
| AF020946 | Tnnt1 | 3.6 | slow skeletal muscle troponin T (Tnnt1) mRNA, complete cds. |
| AF075263 | Slc6a18 | 3.6 | alt 'D630001K16Rik#XT2#Xtrp2' |
| BC152821 | Rimbp2 | 3.5 | RIMS binding protein 2 (Rimbp2) mRNA, encodes complete protein. |
| XM_001476287 | Gm3400 | 3.5 | hypothetical protein LOC100041544 (LOC100041544), mRNA. |
| XM_001487801 | Olfr471 | 3.5 | olfactory receptor 471 (Olfr471), mRNA. |
| DQ358971 | Shroom2 | 3.4 | Apxl protein mRNA, complete cds. |
| NM_001110228 | Cugbp2 | 3.4 | CUG triplet repeat, RNA binding protein 2 (Cugbp2), transcript variant 1, mRNA. |
| XM_001479316 | Gm4019 | 3.4 | hypothetical protein LOC100042766 (LOC100042766), mRNA. |
| AF195418 | Odz3 | 3.4 | ODZ3 (Odz3) mRNA, partial cds. |
| AK005849 | 1700010N08Rik | 3.4 | unclassifiable, full insert sequence. |
| AK006290 | Ppcs | 3.4 | hypothetical protein, full insert sequence. |
| AB255881 | Abca8a | 3.3 | abca8a mRNA for ABC transporter A subfamily member, A8a, complete cds. |
| AF302075 | Mmel1 | 3.3 | neprilysin-like peptidase alpha mRNA, complete cds. |
| M16120 | N/A | 3.2 | T-cell receptor insulin B-chain reactive beta chain VNDNJC, complete cds. |
| XM_001473015 | Gm2274 | 3.2 | Annexin A11 (LOC100039503), mRNA. |
| XM_001477939 | Gm3789 | 3.2 | phosphatidylserine decarboxylase (LOC100042322), mRNA. |
| XM_988922 | 4933436C20Rik | 3.2 | RIKEN cDNA 4933436C20 gene (4933436C20Rik), mRNA. |
| BC156690 | Depdc6 | 3.1 | DEP domain containing 6 (Depdc6) mRNA, encodes complete protein. |
| XM_001476672 | Gm3428 | 3.1 | hypothetical protein LOC100041605 (LOC100041605), mRNA. |
| XM_358761 | Tekt5 | 2.9 | tektin 5 (Tekt5), mRNA. |
| AB009369 | Ccr6 | 2.9 | Mus musculus mRNA for G protein-coupled receptor KY411, complete cds. |
| AB185844 | Wdr67 | 2.9 | 4-B-3 mRNA for hypothetical protein 4-B-3, complete cds. |
| AB294522 | N/A | 2.9 | RNA, mRNA-like noncoding RNA, partial sequence, clone: Gomafu isofom#3. |
| AF416641 | Hif3a | 2.9 | inhibitory PAS domain protein (Ipas) mRNA, complete cds. |
| AK002370 | Atp5sl | 2.9 | hypothetical RNI-like structure containing protein, full insert sequence. |
| EF662059 | Aldoa | 2.8 | alt 'Aldo-1#Aldo1#MGC107164' |
| XM_001480180 | Gm15583 | 2.8 | Zinc finger, BED domain containing 4 (LOC100043240), mRNA. |
| AB000096 | Gata2 | 2.8 | GATA-2 protein, complete cds. |
| AF490340 | Pramel4 | 2.8 | PRAMEl4 mRNA, complete cds. |
| AF515707 | Tnfrsf14 | 2.8 | herpes virus entry mediator (Hvem) mRNA, complete cds. |
| AK004382 | 1110065P20Rik | 2.8 | unclassifiable, full insert sequence. |
| AK005213 | Rsrc2 | 2.8 | SPLICING FACTOR, ARGININE/SERINE-RICH 6 (PRE-MRNA SPLICING FACTOR SRP55) (FRAGMENT) [Oryctolagus cuniculus], full insert sequence. |
| AK005973 | 4930519G04Rik | 2.8 | RIKEN cDNA 1700014B12 gene, full insert sequence. |
| DQ143894 | Tgfb1i1 | 2.7 | transforming growth factor beta 1 isoform alpha-C (Tgfb1i1) mRNA, complete cds. |
| AK005150 | Ssbp2 | 2.7 | hypothetical protein, full insert sequence. |
| BC152773 | Cbx6-Nptxr | 2.6 | neuronal pentraxin with chromo domain (Npcd) mRNA, encodes complete protein. |
| AF016695 | Muc2 | 2.6 | colonic mucin (MUC2) mRNA, partial cds. |
| AF188011 | Plec1 | 2.6 | plectin isoform plec 1b,2alpha (Plec1) mRNA, partial cds. |
| AF312018 | Krt81 | 2.6 | type II hair keratin mRNA, partial cds. |
| XM_001475797 | Iqcf6 | 2.5 | hCG1789065 (LOC100041096), mRNA. |
| XM_001481110 | Gm6936 | 2.5 | hypothetical protein LOC628951 (LOC628951), mRNA. |
| Z78161 | Rpe | 2.5 | partial cochlear mRNA (clone 20F5). |
| AJ279793 | Tspan32 | 2.5 | Tssc6 protein (tssc6 gene, transcript 3). |
| XM_006721795.2 | MMD | 2.4 | monocyte to macrophage differentiation-associated |
| BC156494 | Myo7a | 2.4 | myosin VIIa (Myo7a) mRNA, encodes complete protein. |
| XM_001480060 | 2410125D13Rik | 2.4 | RIKEN cDNA 2410125D13 gene (2410125D13Rik), mRNA. |
| NM_057810.4 | CPR | 2.3 | cytochrome P450 reductase |
| XM_892576 | Gm6801 | 2.3 | predicted gene, EG627866 (EG627866), mRNA. |
| AF128117 | Aire | 2.3 | alt 'MGC123374#MGC123375' |
| AF280811 | Dusp12 | 2.3 | T-DSP4 splice variant mRNA, complete cds. |
| NM_006148.3 | LASP1 | 2.1 | LIM and SH3 protein 1 |
| DQ157177 | Pttg1 | 2.1 | PTTG3 (Pttg3) mRNA, complete cds. |
| NG_012979.1 | PPIB | 1.9 | peptidylprolyl isomerase B (cyclophilin B) |
| BC152758 | Hs3st2 | 1.9 | heparan sulfate (glucosamine) 3-O-sulfotransferase 2 (Hs3st2) mRNA, encodes complete protein. |
| BC156087 | Gabbr2 | 1.9 | gamma-aminobutyric acid (GABA) B receptor 2 (Gabbr2) mRNA, encodes complete protein. |
| AK220380 | BC030046 | 1.8 | mKIAA1305 protein. |
| BC157086 | Abl2 | 1.8 | v-abl Abelson murine leukemia viral oncogene 2 (arg, Abelson-related gene) (Abl2) mRNA, encodes complete protein. |
| BC148725 | Dgke | 1.6 | diacylglycerol kinase, epsilon (Dgke) mRNA, encodes complete protein. |
| BC103530 | Cckbr | 1.49 | cholecystokinin B receptor, mRNA (cDNA clone MGC:123399 IMAGE:40039482), complete cds. |
| BC113753 | Dmp1 | 1.49 | dentin matrix protein 1, mRNA (cDNA clone MGC:130441 IMAGE:40039844), complete cds. |
| BC115586 | Aqp6 | 1.49 | aquaporin 6, mRNA (cDNA clone MGC:144473 IMAGE:40102369), complete cds. |
| BC119364 | Myadm | 1.49 | myeloid-associated differentiation marker, mRNA (cDNA clone MGC:155680 IMAGE:8734113), complete cds. |
| BC125366 | Il5 | 1.49 | interleukin 5, mRNA (cDNA clone MGC:159069 IMAGE:40129881), complete cds. |
| BC132380 | Htra4 | 1.49 | HtrA serine peptidase 4, mRNA (cDNA clone MGC:164011 IMAGE:40130657), complete cds. |
| BC132537 | Tll2 | 1.49 | tolloid-like 2, mRNA (cDNA clone MGC:164168 IMAGE:40130814), complete cds. |
| BC141529 | Otop2 | 1.49 | otopetrin 2 (Otop2) mRNA, encodes complete protein. |
| BC146438 | Tacc1 | 1.49 | transforming, acidic coiled-coil containing protein 1 (Tacc1) mRNA, encodes complete protein. |
| BC107201 | Rasl10a | 1.48 | RAS-like, family 10, member A, mRNA (cDNA clone MGC:130094 IMAGE:40051192), complete cds. |
| BC114395 | Ap3d1 | 1.48 | olfactory receptor 301, mRNA (cDNA clone MGC:140989 IMAGE:40045563), complete cds. |
| BC116220 | Map6d1 | 1.48 | MAP6 domain containing 1, mRNA (cDNA clone MGC:143618 IMAGE:40091320), complete cds. |
| BC116730 | Tmem169 | 1.48 | transmembrane protein 169, mRNA (cDNA clone MGC:151107 IMAGE:40126049), complete cds. |
| BC117006 | Prok1 | 1.48 | prokineticin 1, mRNA (cDNA clone MGC:151383 IMAGE:40126325), complete cds. |
| BC117800 | Galnt14 | 1.48 | UDP-N-acetyl-alpha-D-galactosamine:polypeptide N-acetylgalactosaminyltransferase 14, mRNA (cDNA clone MGC:143695 IMAGE:40092267), complete cds. |
| BC117803 | Slc16a7 | 1.48 | solute carrier family 16 (monocarboxylic acid transporters), member 7, mRNA (cDNA clone MGC:143699 IMAGE:40092297), complete cds. |
| BC117892 | Prickle1 | 1.48 | prickle like 1 (Drosophila), mRNA (cDNA clone MGC:144029 IMAGE:40096843), complete cds. |
| BC129890 | Tgm3 | 1.48 | transglutaminase 3, E polypeptide, mRNA (cDNA clone MGC:150256 IMAGE:40111487), complete cds. |
| BC140314 | Hrh1 | 1.48 | histamine receptor H 1 (Hrh1) mRNA, encodes complete protein. |
| BC141509 | Egr4 | 1.48 | early growth response 4 (Egr4) mRNA, encodes complete protein. |
| BC145743 | Htr3a | 1.48 | 5-hydroxytryptamine (serotonin) receptor 3A, mRNA (cDNA clone MGC:175630 IMAGE:40131046), complete cds. |
| BC148569 | Ipcef1 | 1.48 | RIKEN cDNA A130090K04 gene (A130090K04Rik) mRNA, encodes complete protein. |
| BC116306 | Kcnq3 | 1.47 | potassium voltage-gated channel, subfamily Q, member 3, mRNA (cDNA clone MGC:143935 IMAGE:40095195), complete cds. |
| BC126962 | Aspg | 1.47 | RIKEN cDNA A530050D06 gene, mRNA (cDNA clone MGC:143744 IMAGE:40092851), complete cds. |
| BC116790 | Creg2 | 1.46 | cellular repressor of E1A-stimulated genes 2, mRNA (cDNA clone MGC:151167 IMAGE:40126109), complete cds. |
| BC116881 | 1700029F09Rik | 1.46 | RIKEN cDNA 1700029F09 gene, mRNA (cDNA clone MGC:151258 IMAGE:40126200), complete cds. |
| BC126874 | Necab3 | 1.46 | amyloid beta (A4) precursor protein-binding, family A, member 2 binding protein, mRNA (cDNA clone MGC:123940 IMAGE:40044848), complete cds. |
| BC145940 | Lpar6 | 1.46 | purinergic receptor P2Y, G-protein coupled, 5, mRNA (cDNA clone MGC:175827 IMAGE:40131243), complete cds. |
| BC146393 | V1rh11 | 1.46 | vomeronasal 1 receptor, H11 (V1rh11) mRNA, encodes complete protein. |
| BC113146 | Ccbp2 | 1.45 | chemokine binding protein 2, mRNA (cDNA clone MGC:130576 IMAGE:40055448), complete cds. |
| BC116874 | Fut9 | 1.45 | fucosyltransferase 9, mRNA (cDNA clone MGC:151251 IMAGE:40126193), complete cds. |
| BC131903 | 2310046A06Rik | 1.45 | RIKEN cDNA 2310046A06 gene, mRNA (cDNA clone MGC:163534 IMAGE:40130180), complete cds. |
| BC148640 | Fcrlb | 1.45 | Fc receptor-like B (Fcrlb) mRNA, encodes complete protein. |
| BC103785 | Igh | 1.44 | immunoglobulin heavy chain complex, mRNA (cDNA clone MGC:118142 IMAGE:6477061), complete cds. |
| BC107265 | 2300002M23Rik | 1.44 | RIKEN cDNA 2300002M23 gene, mRNA (cDNA clone MGC:130211 IMAGE:40052572), complete cds. |
| BC115867 | Slc24a2 | 1.44 | solute carrier family 24 (sodium/potassium/calcium exchanger), member 2, mRNA (cDNA clone MGC:141478 IMAGE:40081382), complete cds. |
| BC132523 | Rtn4rl2 | 1.44 | reticulon 4 receptor-like 2, mRNA (cDNA clone MGC:164154 IMAGE:40130800), complete cds. |
| BC145696 | Chrd | 1.44 | chordin, mRNA (cDNA clone MGC:175583 IMAGE:40130999), complete cds. |
| BC100399 | Large | 1.43 | like-glycosyltransferase, mRNA (cDNA clone MGC:118078 IMAGE:30725031), complete cds. |
| BC107388 | Rap1gap2 | 1.43 | GTPase activating RANGAP domain-like 4, mRNA (cDNA clone MGC:130388 IMAGE:40057754), complete cds. |
| BC129839 | Fam13c | 1.43 | RIKEN cDNA 1200015N20 gene, mRNA (cDNA clone MGC:149985 IMAGE:40060810), complete cds. |
| BC104280 | Olfr138 | 1.42 | olfactory receptor 138, mRNA (cDNA clone MGC:129198 IMAGE:40041704), complete cds. |
| BC104406 | Erp27 | 1.42 | endoplasmic reticulum protein 27, mRNA (cDNA clone MGC:129514 IMAGE:40050532), complete cds. |
| BC111807 | Prkcc | 1.42 | protein kinase C, gamma, mRNA (cDNA clone MGC:130440 IMAGE:40039740), complete cds. |
| BC115640 | Cnih3 | 1.42 | cornichon homolog 3 (Drosophila), mRNA (cDNA clone MGC:144544 IMAGE:40103001), complete cds. |
| BC116289 | Kcnc2 | 1.42 | potassium voltage gated channel, Shaw-related subfamily, member 2, mRNA (cDNA clone MGC:143909 IMAGE:40094886), complete cds. |
| BC117003 | Plekho1 | 1.42 | pleckstrin homology domain containing, family O member 1, mRNA (cDNA clone MGC:151380 IMAGE:40126322), complete cds. |
| BC117833 | Lrrc4 | 1.42 | leucine rich repeat containing 4, mRNA (cDNA clone MGC:143757 IMAGE:40092947), complete cds. |
| BC125378 | H2-T3 | 1.42 | histocompatibility 2, T region locus 3, mRNA (cDNA clone MGC:159081 IMAGE:40129893), complete cds. |
| BC127076 | Pcdh12 | 1.42 | protocadherin 12, mRNA (cDNA clone IMAGE:40110769), complete cds. |
| BC129892 | Chrm3 | 1.42 | cholinergic receptor, muscarinic 3, cardiac, mRNA (cDNA clone MGC:150258 IMAGE:40111498), complete cds. |
| BC132390 | Grit | 1.42 | Rho GTPase-activating protein, mRNA (cDNA clone MGC:164021 IMAGE:40130667), complete cds. |
| BC148560 | Ephx4 | 1.42 | abhydrolase domain containing 7 (Abhd7) mRNA, encodes complete protein. |
| BC115655 | 2310042E22Rik | 1.41 | RIKEN cDNA 2310042E22 gene, mRNA (cDNA clone MGC:144564 IMAGE:40103183), complete cds. |
| BC128376 | Arpp21 | 1.41 | cyclic AMP-regulated phosphoprotein, 21, mRNA (cDNA clone IMAGE:40111791), complete cds. |
| BC132172 | C330023M02Rik | 1.41 | RIKEN cDNA C330023M02 gene, mRNA (cDNA clone MGC:163803 IMAGE:40130449), complete cds. |
| BC103675 | Crhr1 | 1.4 | corticotropin releasing hormone receptor 1, mRNA (cDNA clone MGC:124237 IMAGE:40046992), complete cds. |
| BC103568 | Egr3 | 1.39 | early growth response 3, mRNA (cDNA clone MGC:124006 IMAGE:40045218), complete cds. |
| BC107008 | Cdh6 | 1.39 | cadherin 6, mRNA (cDNA clone MGC:129338 IMAGE:40047565), complete cds. |
| BC107027 | Igfn1 | 1.39 | RIKEN cDNA 9830123M21 gene, mRNA (cDNA clone MGC:129490 IMAGE:40050318), complete cds. |
| BC108408 | Nr2f1 | 1.39 | nuclear receptor subfamily 2, group F, member 1, mRNA (cDNA clone MGC:118474 IMAGE:30245025), complete cds. |
| BC110562 | Rras | 1.39 | Harvey rat sarcoma oncogene, subgroup R, mRNA (cDNA clone MGC:129444 IMAGE:40049793), complete cds. |
| BC115871 | Cacna2d1 | 1.39 | calcium channel, voltage-dependent, alpha2/delta subunit 1, mRNA (cDNA clone MGC:141430 IMAGE:40061614), complete cds. |
| BC116909 | Icam4 | 1.39 | intercellular adhesion molecule 4, Landsteiner-Wiener blood group, mRNA (cDNA clone MGC:151286 IMAGE:40126228), complete cds. |
| BC117749 | Bmp3 | 1.39 | bone morphogenetic protein 3, mRNA (cDNA clone MGC:143536 IMAGE:40090489), complete cds. |
| BC117897 | Snph | 1.39 | syntaphilin, mRNA (cDNA clone MGC:144040 IMAGE:40096952), complete cds. |
| BC120674 | Grap | 1.39 | GRB2-related adaptor protein, mRNA (cDNA clone MGC:155911 IMAGE:40129597), complete cds. |
| BC125576 | Kbtbd5 | 1.39 | kelch repeat and BTB (POZ) domain containing 5, mRNA (cDNA clone MGC:159279 IMAGE:40130091), complete cds. |
| BC128477 | Kcnv1 | 1.39 | potassium channel, subfamily V, member 1, mRNA (cDNA clone MGC:156405 IMAGE:40092817), complete cds. |
| BC132521 | Dcn | 1.39 | decorin, mRNA (cDNA clone MGC:164152 IMAGE:40130798), complete cds. |
| BC148441 | Cdk3 | 1.39 | cyclin-dependent kinase 3 (Cdk3) mRNA, encodes complete protein. |
| BC109012 | Kcnh1 | 1.38 | potassium voltage-gated channel, subfamily H (eag-related), member 1, mRNA (cDNA clone MGC:124419 IMAGE:40048708), complete cds. |
| BC111107 | Eif4ebp3 | 1.38 | eukaryotic translation initiation factor 4E binding protein 3, mRNA (cDNA clone MGC:130577 IMAGE:40055518), complete cds. |
| BC114580 | N/A | 1.38 | cDNA clone IMAGE:40053280. |
| BC115981 | Olfm2 | 1.38 | olfactomedin 2, mRNA (cDNA clone MGC:144795 IMAGE:40106192), complete cds. |
| BC117795 | Slc35a4 | 1.38 | solute carrier family 35, member A4, mRNA (cDNA clone MGC:143687 IMAGE:40092185), complete cds. |
| BC120843 | Sstr3 | 1.38 | somatostatin receptor 3, mRNA (cDNA clone MGC:156080 IMAGE:40129766), complete cds. |
| BC129851 | Myrip | 1.38 | myosin VIIA and Rab interacting protein, mRNA (cDNA clone MGC:150051 IMAGE:40109680), complete cds. |
| BC130266 | Rapgefl1 | 1.38 | Rap guanine nucleotide exchange factor (GEF)-like 1, mRNA (cDNA clone MGC:161353 IMAGE:40143028), complete cds. |
| BC145775 | Acvr1b | 1.38 | activin A receptor, type 1B, mRNA (cDNA clone MGC:175662 IMAGE:40131078), complete cds. |
| BC148633 | Lmod1 | 1.38 | leiomodin 1 (smooth muscle) (Lmod1) mRNA, encodes complete protein. |
| BC115576 | 5430427O19Rik | 1.37 | RIKEN cDNA 5430427O19 gene, mRNA (cDNA clone MGC:144455 IMAGE:40102201), complete cds. |
| BC118970 | Col19a1 | 1.37 | collagen, type XIX, alpha 1, mRNA (cDNA clone MGC:144116 IMAGE:40097873), complete cds. |
| BC120790 | Mas1 | 1.37 | MAS1 oncogene, mRNA (cDNA clone MGC:156027 IMAGE:40129713), complete cds. |
| BC131648 | Fbxw7 | 1.37 | F-box and WD-40 domain protein 7, archipelago homolog (Drosophila), mRNA (cDNA clone MGC:150136 IMAGE:40110349), complete cds. |
| BC140301 | Dgke | 1.37 | diacylglycerol kinase, epsilon (Dgke) mRNA, encodes complete protein. |
| BC140417 | Lamc2 | 1.37 | laminin, gamma 2 (Lamc2) mRNA, encodes complete protein. |
| BC145659 | Ankle1 | 1.37 | ankyrin repeat domain 41, mRNA (cDNA clone MGC:175546 IMAGE:40130962), complete cds. |
| BC146289 | Grin2a | 1.37 | glutamate receptor, ionotropic, NMDA2A (epsilon 1) (Grin2a) mRNA, encodes complete protein. |
| BC116343 | Arhgef15 | 1.36 | Rho guanine nucleotide exchange factor (GEF) 15, mRNA (cDNA clone MGC:144141 IMAGE:40098168), complete cds. |
| BC119050 | Olfr419 | 1.36 | olfactory receptor 419, mRNA (cDNA clone MGC:155366 IMAGE:8733799), complete cds. |
| BC125586 | St6gal2 | 1.36 | beta galactoside alpha 2,6 sialyltransferase 2, mRNA (cDNA clone MGC:159289 IMAGE:40130101), complete cds. |
| BC145978 | Herc3 | 1.35 | hect domain and RLD 3, mRNA (cDNA clone MGC:175865 IMAGE:40131281), complete cds. |
| BC116996 | Msgn1 | 1.33 | mesogenin 1, mRNA (cDNA clone MGC:151373 IMAGE:40126315), complete cds. |
| BC118969 | Dlgap2 | 1.33 | discs, large (Drosophila) homolog-associated protein 2, mRNA (cDNA clone MGC:144101 IMAGE:40097764), complete cds. |
| BC125303 | Efhd2 | 1.33 | EF hand domain containing 2, mRNA (cDNA clone MGC:159006 IMAGE:40129818), complete cds. |
| BC131919 | A730008H23Rik | 1.33 | RIKEN cDNA A730008H23 gene, mRNA (cDNA clone MGC:163550 IMAGE:40130196), complete cds. |
| BC148411 | Fmnl1 | 1.33 | formin-like 1 (Fmnl1) mRNA, encodes complete protein. |
| BC100727 | Snai3 | 1.32 | snail homolog 3 (Drosophila), mRNA (cDNA clone MGC:124017 IMAGE:40045283), complete cds. |
| BC103533 | Mybl1 | 1.32 | myeloblastosis oncogene-like 1, mRNA (cDNA clone MGC:123416 IMAGE:40039678), complete cds. |
| BC103611 | Shc3 | 1.32 | src homology 2 domain-containing transforming protein C3, mRNA (cDNA clone MGC:124163 IMAGE:40046304), complete cds. |
| BC109166 | Npas2 | 1.32 | neuronal PAS domain protein 2, mRNA (cDNA clone MGC:129355 IMAGE:40048145), complete cds. |
| BC117890 | Slitrk4 | 1.32 | SLIT and NTRK-like family, member 4, mRNA (cDNA clone MGC:144017 IMAGE:40096585), complete cds. |
| BC117896 | Snph | 1.32 | syntaphilin, mRNA (cDNA clone MGC:144039 IMAGE:40096951), complete cds. |
| BC125383 | Dio2 | 1.32 | deiodinase, iodothyronine, type II, mRNA (cDNA clone MGC:159086 IMAGE:40129898), complete cds. |
| BC131933 | D18Ertd653e | 1.32 | DNA segment, Chr 18, ERATO Doi 653, expressed, mRNA (cDNA clone MGC:163564 IMAGE:40130210), complete cds. |
| BC141426 | Cntn3 | 1.32 | contactin 3 (Cntn3) mRNA, encodes complete protein. |
| BC145973 | Gabrb2 | 1.32 | gamma-aminobutyric acid (GABA-A) receptor, subunit beta 2, mRNA (cDNA clone MGC:175860 IMAGE:40131276), complete cds. |
| BC148622 | Afap1l1 | 1.32 | actin filament associated protein 1-like 1 (Afap1l1) mRNA, encodes complete protein. |
| BC109343 | Zswim7 | 1.31 | RIKEN cDNA 2410012H22 gene, mRNA (cDNA clone MGC:130274 IMAGE:40053371), complete cds. |
| BC119784 | Kcnk4 | 1.31 | potassium channel, subfamily K, member 4, mRNA (cDNA clone MGC:144821 IMAGE:40106379), complete cds. |
| BC148491 | Rnf222 | 1.31 | RIKEN cDNA 9930039A11 gene (9930039A11Rik) mRNA, encodes complete protein. |
| BC103672 | Kcnj3 | 1.3 | potassium inwardly-rectifying channel, subfamily J, member 3, mRNA (cDNA clone IMAGE:40046974), containing frame-shift errors. |
| BC109365 | Ulk4 | 1.3 | unc-51-like kinase 4 (C. elegans), mRNA (cDNA clone MGC:130433 IMAGE:40059233), complete cds. |
| AK010390 | 2410004I01Rik | 1.3 | unclassifiable, full insert sequence. |
| AK011021 | Polr2l | 1.3 | DNA-DIRECTED RNA POLYMERASE II 7.6 KDA POLYPEPTIDE (EC 2.7.7.6) (RPB10) (RPB7.6) homolog [Drosophila melanogaster], full insert sequence. |
| AK011549 | 2900053A13Rik | 1.3 | unclassifiable, full insert sequence. |
| AK012683 | Pmm2 | 1.3 | phosphomannomutase 2, full insert sequence. |
| AK013506 | 2900009J06Rik | 1.3 | unclassifiable, full insert sequence. |
| AK018164 | Pik3ca | 1.3 | unclassifiable, full insert sequence. |
| AK020074 | 6030458C11Rik | 1.3 | 6030458C11RIK PROTEIN homolog [Mus musculus], full insert sequence. |
| AK028305 | Zic4 | 1.3 | zinc finger protein of the cerebellum 4, full insert sequence. |
| AK033854 | 9330102E08Rik | 1.3 | unclassifiable, full insert sequence. |
| AK006431 | 1700025K23Rik | 1.29 | RIKEN cDNA 1700025K23 gene, full insert sequence. |
| AK007436 | Adamts9 | 1.29 | ADAMTS-9 PRECURSOR (EC 3.4.24.-) (A DISINTEGRIN AND METALLOPROTEINASE WITH THROMBOSPONDIN MOTIFS 9) (ADAM-TS 9) (ADAM-TS9) [Homo sapiens], full insert sequence. |
| AK007524 | Fam32a | 1.29 | hypothetical protein, full insert sequence. |
| AK007971 | 9430008C03Rik | 1.29 | unclassifiable, full insert sequence. |
| AK008240 | Snrpf | 1.29 | SMALL NUCLEAR RIBONUCLEOPROTEIN F (SNRNP-F) (SM PROTEIN F) (SM-F) (SMF) homolog [Homo sapiens], full insert sequence. |
| AK008515 | Tmem134 | 1.29 | RIKEN cDNA 2410001H17 gene, full insert sequence. |
| AK008724 | 2210013O21Rik | 1.29 | unclassifiable, full insert sequence. |
| AK009502 | Tia1 | 1.29 | cytotoxic granule-associated RNA binding protein 1, full insert sequence. |
| AK010095 | Col22a1 | 1.29 | COLLAGEN-LIKE PROTEIN [Herpesvirus saimiri], full insert sequence. |
| AK012310 | 9530068E07Rik | 1.29 | immunoglobulin kappa chain variable 4 (V4), full insert sequence. |
| AK013162 | Nkain1 | 1.29 | unclassifiable, full insert sequence. |
| AK013303 | Erp29 | 1.29 | endoplasmic retuclum protein 29, full insert sequence. |
| AK014231 | Casp3 | 1.29 | caspase 3, apoptosis related cysteine protease, full insert sequence. |
| AK014640 | Hmgxb4 | 1.29 | HIGH-MOBILITY GROUP PROTEIN 2-LIKE 1 (HMGBCG PROTEIN) [Homo sapiens], full insert sequence. |
| AK016316 | Mtfr1 | 1.29 | hypothetical protein, full insert sequence. |
| AK017516 | Snx21 | 1.29 | hypothetical protein, full insert sequence. |
| AK017893 | 5830403M04Rik | 1.29 | unclassifiable, full insert sequence. |
| AK018663 | 9130404H23Rik | 1.29 | hypothetical protein, full insert sequence. |
| AK018977 | 1700097N02Rik | 1.29 | RIKEN cDNA 1700097N02 gene, full insert sequence. |
| AK019365 | 5330430P22Rik | 1.29 | unclassifiable, full insert sequence. |
| AK019796 | 4930570D08Rik | 1.29 | unclassifiable, full insert sequence. |
| AK020054 | 6030426L16Rik | 1.29 | KRUPPEL-RELATED ZINC FINGER PROTEIN F80-L [Mus musculus], full insert sequence. |
| AK020307 | Lcn12 | 1.29 | hypothetical Lipocalin-related protein and Bos/Can/Equ allergen containing protein, full insert sequence. |
| AK028200 | Bbs9 | 1.29 | PTH-RESPONSIVE OSTEOSARCOMA B1 PROTEIN [Homo sapiens], full insert sequence. |
| AK030448 | 6030443J06Rik | 1.29 | unclassifiable, full insert sequence. |
| AK031352 | N/A | 1.29 | DNA polymerase epsilon, full insert sequence. |
| AK035242 | Rnf111 | 1.29 | arkadia, full insert sequence. |
| AK035387 | 9530028C05 | 1.29 | HISTOCOMPATIBILITY 2, CLASS II ANTIGEN E BETA [Mus musculus], full insert sequence. |
| AK035674 | Cdcp1 | 1.29 | CUB domain containing protein 1, full insert sequence. |
| AK038079 | A330009N23Rik | 1.29 | unclassifiable, full insert sequence. |
| AK039855 | Tns4 | 1.29 | Tensin (Fragment) [Rattus norvegicus], full insert sequence. |
| AK039938 | Parp11 | 1.29 | hypothetical protein, full insert sequence. |
| AK040218 | A430078G23Rik | 1.29 | unclassifiable, full insert sequence. |
| AK040553 | N/A | 1.29 | unclassifiable, full insert sequence. |
| AK040733 | 9330159M07Rik | 1.29 | unclassifiable, full insert sequence. |
| AK041330 | Dpp3 | 1.29 | DIPEPTIDYLPEPTIDASE III [Homo sapiens], full insert sequence. |
| AK041468 | A630012P03Rik | 1.29 | unclassifiable, full insert sequence. |
| AK041716 | N/A | 1.29 | unclassifiable, full insert sequence. |
| AK041849 | Ccdc90a | 1.29 | hypothetical protein, full insert sequence. |
| AK042745 | Agps | 1.29 | ALKYL-DIHYDROXYACETONEPHOSPHATE SYNTHASE [Rattus norvegicus], full insert sequence. |
| AK044132 | Lins2 | 1.29 | hypothetical protein, full insert sequence. |
| AK045252 | Pbx1 | 1.29 | pre B-cell leukemia transcription factor 1, full insert sequence. |
| AK046043 | Apol8 | 1.29 | hypothetical protein, full insert sequence. |
| AK046394 | Cdkl3 | 1.29 | cyclin-dependent kinase-like 3, full insert sequence. |
| AK047414 | Lrrc14 | 1.29 | hypothetical RNI-like structure containing protein, full insert sequence. |
| AK048254 | Rbbp8 | 1.29 | hypothetical protein, full insert sequence. |
| AK048772 | Ranbp10 | 1.29 | hypothetical protein, full insert sequence. |
| AK049843 | Spock3 | 1.29 | TESTICAN-3 PROTEIN PRECURSOR (2900045C01RIK PROTEIN) homolog [Mus musculus], full insert sequence. |
| AK050030 | Mipol1 | 1.29 | hypothetical protein, full insert sequence. |
| AK050922 | Erg | 1.29 | Erg mRNA, mouse homolog of Human ets-related gene ERG, transcript variant 1, full insert sequence. |
| AK051795 | Mcm5 | 1.29 | mini chromosome maintenance deficient 5 (S. cerevisiae), full insert sequence. |
| AK052187 | Insr | 1.29 | insulin receptor, full insert sequence. |
| AK052589 | Ano1 | 1.29 | HYPOTHETICAL 68.9 KDA PROTEIN homolog [Homo sapiens], full insert sequence. |
| AK052911 | Trim63 | 1.29 | RING ZINC FINGER PROTEIN SMRZ [Homo sapiens], full insert sequence. |
| AK053689 | Ntng1 | 1.29 | hypothetical protein, full insert sequence. |
| AK053903 | Fam150b | 1.29 | acid phosphatase 1, soluble, full insert sequence. |
| AK077331 | Trpm3 | 1.29 | MELASTATIN 2 (FRAGMENT) [Homo sapiens], full insert sequence. |
| AK007112 | 1700102J08Rik | 1.28 | unclassifiable, full insert sequence. |
| AK007151 | 1700109I08Rik | 1.28 | unclassifiable, full insert sequence. |
| AK007605 | Ggps1 | 1.28 | RIKEN cDNA 1810026C22 gene, full insert sequence. |
| AK008083 | Alkbh4 | 1.28 | hypothetical 2OG-Fe(II) oxygenase superfamily containing protein, full insert sequence. |
| AK008395 | Robld3 | 1.28 | mitogen activated protein binding protein interacting protein, full insert sequence. |
| AK011742 | Tspan18 | 1.28 | RIKEN cDNA 2610042G18 gene, full insert sequence. |
| AK011854 | Fam125b | 1.28 | hypothetical protein, full insert sequence. |
| AK012711 | Snx24 | 1.28 | hypothetical PX (Bem1/NCF1/PI3K) domain containing protein, full insert sequence. |
| AK012780 | Hspb2 | 1.28 | heat shock 27kD protein 2, full insert sequence. |
| AK013380 | Ncapd2 | 1.28 | KIAA0159 PROTEIN [Homo sapiens], full insert sequence. |
| AK014981 | 4921529N20Rik | 1.28 | unclassifiable, full insert sequence. |
| AK015272 | Armc10 | 1.28 | hypothetical protein, full insert sequence. |
| AK015826 | Galntl6 | 1.28 | hypothetical Ricin B-like lectin structure containing protein, full insert sequence. |
| AK016341 | 5133400G04Rik | 1.28 | hypothetical protein, full insert sequence. |
| AK017085 | Gm11149 | 1.28 | neural cell adhesion molecule, full insert sequence. |
| AK017548 | Tle4 | 1.28 | TRANSDUCIN-LIKE ENHANCER PROTEIN 4 homolog [Homo sapiens], full insert sequence. |
| AK019085 | Uqcrh | 1.28 | UBIQUINOL-CYTOCHROME C REDUCTASE COMPLEX 11 KDA PROTEIN, MITOCHONDRIAL PRECURSOR (EC 1.10.2.2) (MITOCHONDRIAL HINGE PROTEIN) (CYTOCHROME C1, NONHEME 11 KDA PROTEIN) (COMPLEX III SUBUNIT VIII) [Mus musculus], full insert sequence. |
| AK019598 | 4930435F05Rik | 1.28 | unclassifiable, full insert sequence. |
| AK019697 | 4930525C09Rik | 1.28 | unclassifiable, full insert sequence. |
| AK020764 | A430104N18Rik | 1.28 | unclassifiable, full insert sequence. |
| AK028605 | Akr1e1 | 1.28 | ldo-keto reductase family 1, member E1, full insert sequence. |
| AK029170 | Stard9 | 1.28 | Serine-rich region containing protein, full insert sequence. |
| AK029802 | Lrrc9 | 1.28 | hypothetical RNI-like structure and ADP-ribosylation structure containing protein, full insert sequence. |
| AK030091 | N/A | 1.28 | SDR1 PROTEIN homolog [Mus musculus], full insert sequence. |
| AK030946 | 6720401G13Rik | 1.28 | unclassifiable, full insert sequence. |
| AK031825 | Bcas1 | 1.28 | BREAST CARCINOMA AMPLIFIED SEQUENCE 1 (NOVEL AMPLIFIED IN BREAST CANCER 1) (AMPLIFIED AND OVEREXPRESSED IN BREAST CANCER) [Homo sapiens], full insert sequence. |
| AK032167 | Lrrfip2 | 1.28 | leucine rich repeat (in FLII) interacting protein 2, full insert sequence. |
| AK033154 | Scara5 | 1.28 | hypothetical protein, full insert sequence. |
| AK033461 | Acsm3 | 1.28 | SA rat hypertension-associated homolog, full insert sequence. |
| AK035105 | Stx8 | 1.28 | syntaxin 8, full insert sequence. |
| AK035767 | 4932438H23Rik | 1.28 | PUTATIVE PROTEIN C21ORF62 HOMOLOG, full insert sequence. |
| AK036110 | G630025P09Rik | 1.28 | unclassifiable, full insert sequence. |
| AK038886 | N/A | 1.28 | unclassifiable, full insert sequence. |
| AK039634 | A330076C08Rik | 1.28 | unclassifiable, full insert sequence. |
| AK040396 | Ptgr2 | 1.28 | RIKEN cDNA 1810016I24 gene, full insert sequence. |
| AK041109 | A530083I20Rik | 1.28 | unclassifiable, full insert sequence. |
| AK043617 | Slc35f4 | 1.28 | hypothetical protein, full insert sequence. |
| AK043879 | Abi3bp | 1.28 | Hypothetical fibronectin type III domain containing protein, full insert sequence. |
| AK044505 | A930017K11Rik | 1.28 | unclassifiable, full insert sequence. |
| AK044866 | Dpysl5 | 1.28 | collapsin response mediator protein 5, full insert sequence. |
| AK045167 | Lias | 1.28 | lipoic acid synthetase, full insert sequence. |
| AK046150 | N/A | 1.28 | unclassifiable, full insert sequence. |
| AK046225 | B230354K17Rik | 1.28 | unclassifiable, full insert sequence. |
| AK046596 | Cadps2 | 1.28 | CEREBELLUM POSTNATAL DEVELOPMENT ASSOCIATED PROTEIN 2 homolog [Mus musculus], full insert sequence. |
| AK047978 | D930049A15Rik | 1.28 | unclassifiable, full insert sequence. |
| AK048484 | Tmem194b | 1.28 | hypothetical protein, full insert sequence. |
| AK048995 | N/A | 1.28 | unclassifiable, full insert sequence. |
| AK049212 | C330013J06Rik | 1.28 | KRUPPEL-RELATED ZINC FINGER PROTEIN F80-L [Mus musculus], full insert sequence. |
| AK049364 | Zfp516 | 1.28 | unclassifiable, full insert sequence. |
| AK050516 | 5033421B08Rik | 1.28 | unclassifiable, full insert sequence. |
| AK051395 | Fgd3 | 1.28 | faciogenital dysplasia homolog 3, full insert sequence. |
| AK052146 | Ets1 | 1.28 | V-ETS AVIAN ERYTHROBLASTOSIS VIRUS E26 ONCOGENE HOMOLOG 1 homolog [Homo sapiens], full insert sequence. |
| AK052319 | Prrt4 | 1.28 | unclassifiable, full insert sequence. |
| AK052679 | Prlr | 1.28 | prolactin receptor related sequence 1, full insert sequence. |
| AK076937 | 4933407I05Rik | 1.28 | unclassifiable, full insert sequence. |
| AK006561 | 1700030M09Rik | 1.27 | unclassifiable, full insert sequence. |
| AK006859 | 1700061N14Rik | 1.27 | unclassifiable, full insert sequence. |
| AK007672 | Mrpl2 | 1.27 | mitochondrial ribosomal protein L2, full insert sequence. |
| AK007812 | Cinp | 1.27 | similar to FROM HELA CYCLIN-DEPENDENT KINASE 2 INTERACTING PROTEIN (CDNA FLJ31550 FIS, CLONE NT2RI2001054, HIGHLY SIMILAR TO HOMO SAPIENS FROM HELA CYCLIN-DEPENDENT KINASE 2 INTERACTING PROTEIN (CINP) MRNA) [Homo sapiens], full insert sequence. |
| AK008077 | 2010003O02Rik | 1.27 | RIKEN cDNA 2010003O02 gene, full insert sequence. |
| AK008095 | Slc35a2 | 1.27 | solute carrier family 35 (UDP-galactose transporter), member 2, full insert sequence. |
| AK008334 | 2010010A06Rik | 1.27 | RECEPTOR TYROSINE KINASE EPHA1 [Mus musculus], full insert sequence. |
| AK008458 | Hps4 | 1.27 | unclassifiable, full insert sequence. |
| AK008731 | Gtpbp2 | 1.27 | GTP binding protein 2, full insert sequence. |
| AK009137 | Prei4 | 1.27 | KIAA1434 PROTEIN (FRAGMENT) [Homo sapiens], full insert sequence. |
| AK009527 | N/A | 1.27 | hypothetical protein, full insert sequence. |
| AK009597 | Fam65c | 1.27 | DJ530I15.2 (NOVEL PROTEIN SIMILAR TO PLACENTAL PROTEIN DIFF40) (FRAGMENT) [Homo sapiens], full insert sequence. |
| AK010071 | Cd209g | 1.27 | DC-SIGN NECK-LESS ISOFORM [Mus musculus], full insert sequence. |
| AK010412 | Nras | 1.27 | NEUROBLASTOMA RAS ONCOGENE homolog [Mus musculus], full insert sequence. |
| AK011333 | Sftpa1 | 1.27 | surfactant associated protein A, full insert sequence. |
| AK011814 | Mtch2 | 1.27 | mitochondrial carrier homolog 2, full insert sequence. |
| AK012880 | 6820431F20Rik | 1.27 | CADHERIN (FRAGMENT) [Gallus gallus], full insert sequence. |
| AK014704 | 4833415N18Rik | 1.27 | unclassifiable, full insert sequence. |
| AK015641 | Crem | 1.27 | cAMP responsive element modulator, full insert sequence. |
| AK015753 | Wdr64 | 1.27 | hypothetical Trp-Asp (WD) repeats profile/Trp-Asp (WD) repeats circular profile/G-protein beta WD-40 repeats containing protein, full insert sequence. |
| AK015810 | 4930470P17Rik | 1.27 | 4930470P17RIK PROTEIN homolog [Mus musculus], full insert sequence. |
| AK016128 | 4930555F03Rik | 1.27 | hypothetical protein, full insert sequence. |
| AK016167 | Prkd3 | 1.27 | PROTEIN KINASE C, NU TYPE (EC 2.7.1.-) (NPKC-NU) (PROTEIN KINASE EPK2) homolog [Homo sapiens], full insert sequence. |
| AK017088 | Cacna1a | 1.27 | calcium channel, voltage-dependent, P/Q type, alpha 1A, full insert sequence. |
| AK017838 | Pnpla8 | 1.27 | CALCIUM-INDEPENDENT PHOSPHOLIPASE A2 (MEMBRANE-ASSOCIATED CALCIUM- INDEPENDENT PHOSPHOLIPASE A2 GAMMA) homolog [Homo sapiens], full insert sequence. |
| AK019114 | 2400009B08Rik | 1.27 | unclassifiable, full insert sequence. |
| AK019152 | Znrd1 | 1.27 | TRANSCRIPTION-ASSOCIATED ZINC RIBBON PROTEIN (NUCLEAR RNA POLYMERASE I SMALL SPECIFIC SUBUNIT RPA12) (SIMILAR TO ZINC RIBBON DOMAIN CONTAINING, 1) [Homo sapiens], full insert sequence. |
| AK019366 | 2900089D17Rik | 1.27 | unclassifiable, full insert sequence. |
| AK019582 | Gsto2 | 1.27 | BA127L20.1 (NOVEL GLUTATHIONE-S-TRANSFERASE) [Homo sapiens], full insert sequence. |
| AK019960 | Set | 1.27 | SET translocation, full insert sequence. |
| AK020367 | Pfkp | 1.27 | phosphofructokinase, platelet, full insert sequence. |
| AK021333 | Btn1a1 | 1.27 | butyrophilin, subfamily 1, member A1, full insert sequence. |
| AK027957 | 1110035M17Rik | 1.27 | unclassifiable, full insert sequence. |
| AK031385 | Arcn1 | 1.27 | COATOMER DELTA SUBUNIT (DELTA-COAT PROTEIN) (DELTA-COP) (ARCHAIN) [Homo sapiens], full insert sequence. |
| AK032552 | Cacna1d | 1.27 | VOLTAGE-GATED CALCIUM CHANNEL PORE FORMING SUBUNIT CAV1.3 ALPHA1D (FRAGMENT) homolog [Rattus norvegicus], full insert sequence. |
| AK033281 | Cep250 | 1.27 | CENTROSOMAL PROTEIN 2 homolog [Homo sapiens], full insert sequence. |
| AK036177 | 1110057K04Rik | 1.27 | hypothetical protein, full insert sequence. |
| AK036960 | Alox12 | 1.27 | arachidonate 12-lipoxygenase, full insert sequence. |
| AK037640 | Top3a | 1.27 | topoisomerase (DNA) III alpha, full insert sequence. |
| AK038638 | Sphkap | 1.27 | hypothetical Signal peptidase containing protein, full insert sequence. |
| AK038993 | LOC622552 | 1.27 | unclassifiable, full insert sequence. |
| AK039299 | AI854703 | 1.27 | Kruppel-type zinc finger protein ZNF72 (fragment) [Homo sapiens], full insert sequence. |
| AK043460 | N/A | 1.27 | unclassifiable, full insert sequence. |
| AK043991 | 3110039M20Rik | 1.27 | unclassifiable, full insert sequence. |
| AK045688 | N/A | 1.27 | hypothetical protein, full insert sequence. |
| AK046429 | 4930422G04Rik | 1.27 | hypothetical P-loop containing nucleotide triphosphate hydrolases structure containing protein, full insert sequence. |
| AK046639 | Eif4g3 | 1.27 | hypothetical protein, full insert sequence. |
| AK049123 | N/A | 1.27 | hypothetical Cysteinyl-tRNA synthetase containing protein, full insert sequence. |
| AK049297 | Prss41 | 1.27 | TESTIS SERINE PROTEASE-1 [Mus musculus], full insert sequence. |
| AK049453 | Sash1 | 1.27 | hypothetical protein, full insert sequence. |
| AK051277 | Nsmce2 | 1.27 | RIKEN cDNA 1110014D18 gene, full insert sequence. |
| AK053098 | Kctd18 | 1.27 | CDNA FLJ31322 FIS, CLONE LIVER2000033, WEAKLY SIMILAR TO TUMOR NECROSIS FACTOR, ALPHA-INDUCED PROTEIN 1, ENDOTHELIAL [Homo sapiens], full insert sequence. |
| AK076548 | 1700008F21Rik | 1.27 | hypothetical protein, full insert sequence. |
| AK077195 | Amz2 | 1.27 | hypothetical Metalloproteases ('zincins'), catalytic domain structure containing protein, full insert sequence. |
| AK077502 | Yipf1 | 1.27 | unclassifiable, full insert sequence. |
| AK006748 | Prdx2 | 1.26 | inferred: peroxiredoxin 2, full insert sequence. |
| AK008182 | Mtap7 | 1.26 | microtubule-associated protein 7, full insert sequence. |
| AK009422 | Gm2382 | 1.26 | 5, 10-methenyltetrahydrofolate synthetase, full insert sequence. |
| AK009887 | Psmg4 | 1.26 | unclassifiable, full insert sequence. |
| AK013026 | Anxa6 | 1.26 | annexin A6, full insert sequence. |
| AK013850 | Anxa3 | 1.26 | annexin A3, full insert sequence. |
| AK018487 | Ckmt1 | 1.26 | creatine kinase, mitochondrial 1, ubiquitous, full insert sequence. |
| AK018725 | Rps19 | 1.26 | ribosomal protein S19, full insert sequence. |
| AK019192 | Atp5l | 1.26 | ATP synthase, H+ transporting, mitochondrial F0 complex, subunit g, full insert sequence. |
| AK019375 | 3010003L10Rik | 1.26 | unclassifiable, full insert sequence. |
| AK020041 | 5930434B04Rik | 1.26 | hypothetical protein, full insert sequence. |
| AK020914 | A930031H19Rik | 1.26 | unclassifiable, full insert sequence. |
| AK027928 | Opn4 | 1.26 | unclassifiable, full insert sequence. |
| AK032967 | Polr3h | 1.26 | RIKEN cDNA 5031409G22 gene, full insert sequence. |
| AK034879 | Gucy1a2 | 1.26 | GUANYLATE CYCLASE SOLUBLE, ALPHA-2 CHAIN (EC 4.6.1.2) (GCS-ALPHA-2) homolog [Rattus norvegicus], full insert sequence. |
| AK037114 | Osbpl3 | 1.26 | oxysterol binding protein-like 3, full insert sequence. |
| AK037486 | 4931408A02Rik | 1.26 | PROTEIN C21ORF63 HOMOLOG PRECURSOR [Mus musculus], full insert sequence. |
| AK040430 | C230035I16Rik | 1.26 | unclassifiable, full insert sequence. |
| AK041155 | Cdc14b | 1.26 | TYROSINE PHOSPHATASE homolog [Homo sapiens], full insert sequence. |
| AK042881 | Pftk2 | 1.26 | hypothetical Protein kinase containing protein, full insert sequence. |
| AK043130 | 3830408C21Rik | 1.26 | RIKEN cDNA 3830408C21 gene, full insert sequence. |
| AK045050 | Rad51 | 1.26 | RAD51 homolog (S. cerevisiae), full insert sequence. |
| AK045560 | Disp2 | 1.26 | KIAA1742 protein {Homo sapiens}, full insert sequence. |
| AK046478 | 4933432B09Rik | 1.26 | hypothetical protein, full insert sequence. |
| AK046865 | Ranbp17 | 1.26 | RAN binding protein 17, full insert sequence. |
| AK049536 | Gm13235 | 1.26 | hypothetical KRAB box containing protein, full insert sequence. |
| AK050117 | N/A | 1.26 | unclassifiable, full insert sequence. |
| AK051512 | Tie1 | 1.26 | tyrosine kinase receptor 1, full insert sequence. |
| AK053180 | Slc35c2 | 1.26 | OVARIAN CANCER OVEREXPRESSED 1 homolog [Homo sapiens], full insert sequence. |
| AK053385 | Ints6 | 1.26 | CANDIDATE TUMOR SUPPRESSOR PROTEIN DICE1 homolog [Homo sapiens], full insert sequence. |
| AK054366 | Zmiz1 | 1.26 | hypothetical Alanine-rich region/Type I antifreeze protein containing protein, full insert sequence. |
| AK076657 | 1700061H18Rik | 1.26 | unclassifiable, full insert sequence. |
| AK077039 | Angel2 | 1.26 | hypothetical Endonuclease/Exonuclease/phosphatase family containing protein, full insert sequence. |
| AK077141 | Morn3 | 1.26 | hypothetical MORN motif containing protein, full insert sequence. |
| AK006653 | 1700040F15Rik | 1.25 | XMR PROTEIN [Mus musculus], full insert sequence. |
| AK007850 | Rps21 | 1.25 | 40S RIBOSOMAL PROTEIN S21 homolog [Rattus norvegicus], full insert sequence. |
| AK010004 | 2310061J03Rik | 1.25 | unclassifiable, full insert sequence. |
| AK010848 | Smc4 | 1.25 | SMC4 PROTEIN homolog [Microtus arvalis], full insert sequence. |
| AK010994 | Fxc1 | 1.25 | :MITOCHONDRIAL IMPORT INNER MEMBRANE TRANSLOCASE SUBUNIT TIM9 B homolog [Mus musculus], full insert sequence. |
| AK011205 | Rbms1 | 1.25 | unclassifiable, full insert sequence. |
| AK011488 | N/A | 1.25 | PSEUDOURIDINE SYNTHASE 3 [Mus musculus], full insert sequence. |
| AK011501 | Csnk2a1 | 1.25 | casein kinase II, alpha 1 polypeptide, full insert sequence. |
| AK013636 | Hspb11 | 1.25 | HSPCO34 PROTEIN [Homo sapiens], full insert sequence. |
| AK016185 | Ncrna00081 | 1.25 | RIKEN cDNA 4930560M04 gene, full insert sequence. |
| AK016677 | Vps24 | 1.25 | NEUROENDOCRINE DIFFERENTIATION FACTOR homolog [Homo sapiens], full insert sequence. |
| AK030186 | Nek11 | 1.25 | hypothetical Eukaryotic protein kinase containing protein, full insert sequence. |
| AK031163 | Col16a1 | 1.25 | procollagen, type XVI, alpha 1, full insert sequence. |
| AK036547 | N/A | 1.25 | arachidonate 5-lipoxygenase activating protein, full insert sequence. |
| AK037999 | Mdn1 | 1.25 | hypothetical protein, full insert sequence. |
| AK038414 | Pyroxd2 | 1.25 | hypothetical FAD/NAD(P)-binding domain structure containing protein, full insert sequence. |
| AK045385 | Gm6145 | 1.25 | unclassifiable, full insert sequence. |
| AK045995 | Rab37 | 1.25 | RAB37, member of RAS oncogene family, full insert sequence. |
| AK053419 | Rad54b | 1.25 | FIBRINOGEN SILENCER BINDING PROTEIN homolog [Homo sapiens], full insert sequence. |
| AK006485 | 1700029E06Rik | 1.24 | unclassifiable, full insert sequence. |
| AK010858 | Cyb5r3 | 1.24 | NADH-CYTOCHROME B5 REDUCTASE (EC 1.6.2.2) homolog [Rattus norvegicus], full insert sequence. |
| AK011460 | 2610019E17Rik | 1.24 | unclassifiable, full insert sequence. |
| AK011550 | Orc4l | 1.24 | origin recognition complex, subunit 4, full insert sequence. |
| AK012007 | 2610307P16Rik | 1.24 | unclassifiable, full insert sequence. |
| AK012387 | 1190002F15Rik | 1.24 | unclassifiable, full insert sequence. |
| AK012696 | Rps25 | 1.24 | ribosomal protein S25, full insert sequence. |
| AK013520 | Necab3 | 1.24 | amyloid beta (A4) precursor protein-binding, family A, member 1 binding protein, full insert sequence. |
| AK014446 | Pvr | 1.24 | RIKEN full-length enriched library, clone:3830421F03 product:hypothetical protein, full insert sequence. |
| AK016004 | Exd2 | 1.24 | :unclassifiable, full insert sequence. |
| AK018652 | Saps3 | 1.24 | SPORULATION-INDUCED TRANSCRIPT 4-ASSOCIATED PROTEIN SAPLB [Homo sapiens], full insert sequence. |
| AK018775 | 2900010J23Rik | 1.24 | RIKEN cDNA 2900010J23 gene, full insert sequence. |
| AK029284 | Wnt10b | 1.24 | WNT-10B PROTEIN PRECURSOR, full insert sequence. |
| AK029768 | N/A | 1.24 | unclassifiable, full insert sequence. |
| AK030090 | Aqr | 1.24 | aquarius, full insert sequence. |
| AK030392 | Fam83h | 1.24 | hypothetical protein, full insert sequence. |
| AK031221 | Uvrag | 1.24 | unclassifiable, full insert sequence. |
| AK032947 | Meis1 | 1.24 | myeloid ecotropic viral integration site 1, full insert sequence. |
| AK034565 | Adamts10 | 1.24 | a disintegrin-like and metalloprotease (reprolysin type) with thrombospondin type 1 motif, 10, full insert sequence. |
| AK035625 | 9130015G15Rik | 1.24 | RIKEN cDNA 9130015G15 gene, full insert sequence. |
| AK038726 | Miat | 1.24 | unclassifiable, full insert sequence. |
| AK038803 | Col4a3bp | 1.24 | collagen, type IV, alpha 3 (Goodpasture antigen) binding protein, full insert sequence. |
| AK039220 | C2cd2l | 1.24 | hypothetical C2 domain (Calcium/lipid-binding domain, CaLB) structure containing protein, full insert sequence. |
| AK039796 | 1700086O06Rik | 1.24 | hypothetical protein, full insert sequence. |
| AK041441 | 4930523C07Rik | 1.24 | hypothetical protein, full insert sequence. |
| AK043731 | Rsrc1 | 1.24 | BM-011 homolog [Homo sapiens], full insert sequence. |
| AK044160 | Bai3 | 1.24 | brain-specific angiogenesis inhibitor 3, full insert sequence. |
| AK044718 | Nr2e3 | 1.24 | nuclear receptor subfamily 2, group E, member 3, full insert sequence. |
| AK048205 | Mcf2l | 1.24 | mcf.2 transforming sequence-like, full insert sequence. |
| AK075948 | Cdc23 | 1.24 | CDC23 (cell division cycle 23, yeast, homolog), full insert sequence. |
| AK076691 | Acrbp | 1.24 | proacrosin binding protein, full insert sequence. |
| AK006348 | 1700025J12Rik | 1.23 | unclassifiable, full insert sequence. |
| AK006978 | 4930442L01Rik | 1.23 | RIKEN cDNA 4930442L01 gene, full insert sequence. |
| AK010463 | Tesc | 1.23 | tescalcin, full insert sequence. |
| AK010638 | N/A | 1.23 | suppressor of variegation 3-9 homolog 1 (Drosophila), full insert sequence. |
| AK010729 | Sult6b1 | 1.23 | SULFOTRANSFERASE [Gallus gallus], full insert sequence. |
| AK011286 | Mapk12 | 1.23 | MITOGEN-ACTIVATED PROTEIN KINASE 12 (EC 2.7.1.-) (EXTRACELLULAR SIGNAL-REGULATED KINASE 6) (ERK-6) (STRESS-ACTIVATED PROTEIN KINASE-3) (MITOGEN-ACTIVATED PROTEIN KINASE P38 GAMMA) (MAP KINASE P38 GAMMA) homolog [Mus musculus], full insert sequence. |
| AK012257 | Gtf2h5 | 1.23 | hypothetical protein, full insert sequence. |
| AK012850 | Serp2 | 1.23 | unclassifiable, full insert sequence. |
| AK013132 | Oxsr1 | 1.23 | overlaps exon 18 of Homo sapiens oxidative-stress responsive 1, full insert sequence. |
| AK014190 | Pms2 | 1.23 | postmeiotic segregation increased 2 (S. cerevisiae), full insert sequence. |
| AK015270 | Gpr62 | 1.23 | G protein-coupled receptor {Homo sapiens}, full insert sequence. |
| AK015918 | Nkiras2 | 1.23 | KAPPA B-RAS 2 (I-KAPPA-B-INTERACTING RAS-LIKE PROTEIN 2) homolog [Homo sapiens], full insert sequence. |
| AK016325 | 4930579J09Rik | 1.23 | IIIG9 LONG FORM homolog [Mus musculus], full insert sequence. |
| AK018169 | 3200002M19Rik | 1.23 | hypothetical Aspartic acid-rich region containing protein, full insert sequence. |
| AK019751 | 4930548J01Rik | 1.23 | unclassifiable, full insert sequence. |
| AK019924 | Gcnt2 | 1.23 | hypothetical Core-2/I-Branching enzyme containing protein, full insert sequence. |
| AK028913 | Itpripl1 | 1.23 | hypothetical protein, full insert sequence. |
| AK029118 | Cntln | 1.23 | hypothetical protein, full insert sequence. |
| AK030562 | Zfp30 | 1.23 | zinc finger protein 30, full insert sequence. |
| AK034866 | Pms2 | 1.23 | postmeiotic segregation increased 2 (S. cerevisiae), full insert sequence. |
| AK035379 | BB123696 | 1.23 | CALCYCLIN BINDING PROTEIN [Mus musculus], full insert sequence. |
| AK035798 | Actr3b | 1.23 | ACTIN-RELATED PROTEIN 3-BETA homolog [Homo sapiens], full insert sequence. |
| AK036059 | Pgls | 1.23 | 6-phosphogluconolactonase, full insert sequence. |
| AK036462 | N/A | 1.23 | unclassifiable, full insert sequence. |
| AK036584 | Zbtb40 | 1.23 | DJ61A9.4 (NOVEL BTB/POZ DOMAIN CONTAINING ZINC FINGER PROTEIN (PRESUMED PART OF DJ61A9.2.2)) (FRAGMENT) [Homo sapiens], full insert sequence. |
| AK036974 | 5930430L01Rik | 1.23 | unclassifiable, full insert sequence. |
| AK037416 | Slc16a5 | 1.23 | MONOCARBOXYLATE TRANSPORTER 6 (MCT 6) (MCT 5) [Homo sapiens], full insert sequence. |
| AK037969 | Etfdh | 1.23 | ELECTRON TRANSFER FLAVOPROTEIN-UBIQUINONE OXIDOREDUCTASE, MITOCHONDRIAL PRECURSOR (EC 1.5.5.1) (ETF-QO) (ETF-UBIQUINONE OXIDOREDUCTASE) (ETF DEHYDROGENASE) (ELECTRON-TRANSFERRING- FLAVOPROTEIN DEHYDROGENASE) homolog [Homo sapiens], full insert sequence. |
| AK039097 | Rpgrip1 | 1.23 | retinitis pigmentosa GTPase regulator interacting protein 1, full insert sequence. |
| AK039802 | Piga | 1.23 | phosphatidylinositol glycan, class A, full insert sequence. |
| AK040147 | Ints3 | 1.23 | hypothetical Glycine-rich region containing protein, full insert sequence. |
| AK040729 | Aoc3 | 1.23 | amine oxidase, copper containing 3, full insert sequence. |
| AK041061 | Ddi2 | 1.23 | unclassifiable, full insert sequence. |
| AK041254 | 1300015D01Rik | 1.23 | unclassifiable, full insert sequence. |
| AK041422 | Ctdspl2 | 1.23 | HSPC129 {Homo sapiens}, full insert sequence. |
| AK041937 | Asah2 | 1.23 | N-acylsphingosine amidohydrolase 2, full insert sequence. |
| AK042393 | Rfc3 | 1.23 | replication factor C (activator 1) 3 (38 kDa), full insert sequence. |
| AK042718 | 9630028H03Rik | 1.23 | unclassifiable, full insert sequence. |
| AK043365 | N/A | 1.23 | unclassifiable, full insert sequence. |
| AK044620 | Slc7a8 | 1.23 | solute carrier family 7 (cationic amino acid transporter, y+ system), member 8, full insert sequence. |
| AK048089 | 9130206I24Rik | 1.23 | unclassifiable, full insert sequence. |
| AK049368 | Slc39a11 | 1.23 | hypothetical ZIP Zinc transporter containing protein, full insert sequence. |
| AK049953 | Dock4 | 1.23 | hypothetical protein, full insert sequence. |
| AK050069 | N/A | 1.23 | unclassifiable, full insert sequence. |
| AK050287 | Rcor3 | 1.23 | CDNA FLJ10876 FIS, CLONE NT2RP4001838, WEAKLY SIMILAR TO HOMO SAPIENS COREST PROTEIN MRNA [Homo sapiens], full insert sequence. |
| AK052221 | Gmip | 1.23 | GEM-INTERACTING PROTEIN [Homo sapiens], full insert sequence. |
| AK052541 | Arhgap22 | 1.23 | Rho GTPase activating protein 22, full insert sequence. |
| AK052729 | Hmbox1 | 1.23 | Homeobox domain containing protein, full insert sequence. |
| AK052933 | Irak1 | 1.23 | interleukin-1 receptor-associated kinase 1, full insert sequence. |
| AK053703 | Lenep | 1.23 | lens epithelial protein, full insert sequence. |
| AK076488 | A330049M08Rik | 1.23 | hypothetical protein, full insert sequence. |
| AK015046 | Kif2c | 1.22 | KINESIN-LIKE 6 (MITOTIC CENTROMERE-ASSOCIATED KINESIN) homolog [Mus musculus], full insert sequence. |
| AK017530 | Nhsl1 | 1.22 | hypothetical protein, full insert sequence. |
| AK018309 | N/A | 1.22 | HIPPOCALCIN-LIKE 1 [Mus musculus], full insert sequence. |
| AK020406 | N/A | 1.22 | hypothetical protein, full insert sequence. |
| AK021362 | Fam55d | 1.22 | unclassifiable, full insert sequence. |
| AK028412 | Srrm1 | 1.22 | serine/arginine repetitive matrix 1, full insert sequence. |
| AK029747 | Bbx | 1.22 | bobby sox homolog (Drosophila), full insert sequence. |
| AK030642 | Dner | 1.22 | delta/notch-like EGF-related receptor, full insert sequence. |
| AK046721 | Gm3693 | 1.22 | unclassifiable, full insert sequence. |
| AK048491 | Whsc1l1 | 1.22 | CDNA FLJ12498 FIS, CLONE NT2RM2001668, WEAKLY SIMILAR TO HOMO SAPIENS PUTATIVE WHSC1 PROTEIN (WHSC1) (PUTATIVE CHROMATIN MODULATOR) (HYPOTHETICAL 72.6 KDA PROTEIN) (WOLF-HIRSCHHORN SYNDROME CANDIDATE 1-LIKE 1) [Homo sapiens], full insert sequence. |
| AK006454 | 1700028E10Rik | 1.21 | unclassifiable, full insert sequence. |
| AK007082 | 1700095J03Rik | 1.21 | serine/threonine kinase 33, full insert sequence. |
| AK010073 | 2310061C15Rik | 1.21 | unclassifiable, full insert sequence. |
| AK010366 | 2410003K15Rik | 1.21 | hypothetical Domain of unknown function DUF143 containing protein, full insert sequence. |
| AK012689 | Exosc3 | 1.21 | APOPTOSIS-RELATED PROTEIN PNAS-3 (FRAGMENT) homolog [Homo sapiens], full insert sequence. |
| AK012888 | Angptl1 | 1.21 | angiopoietin-like 1, full insert sequence. |
| AK013012 | Klhl35 | 1.21 | hypothetical BTB/POZ domain/Kelch repeat containing protein, full insert sequence. |
| AK013095 | Rfc3 | 1.21 | replication factor C (activator 1) 3, full insert sequence. |
| AK013805 | Pmm1 | 1.21 | phosphomannomutase 1, full insert sequence. |
| AK014757 | A330049M08Rik | 1.21 | hypothetical protein, full insert sequence. |
| AK014857 | 4921509O09Rik | 1.21 | unclassifiable, full insert sequence. |
| AK017182 | 5033421C21Rik | 1.21 | RIKEN full-length enriched library, clone:5033421C21 product:hypothetical protein, full insert sequence. |
| AK019083 | Ramp2 | 1.21 | receptor (calcitonin) activity modifying protein 2, full insert sequence. |
| AK020480 | 9430069I07Rik | 1.21 | hypothetical protein, full insert sequence. |
| AK021339 | 4930515G01Rik | 1.21 | unclassifiable, full insert sequence. |
| AK027935 | Gm13179 | 1.21 | unclassifiable, full insert sequence. |
| AK028217 | 0610010O12Rik | 1.21 | PUTATIVE NUCLEAR PROTEIN (PUTATIVE NUCLEAR PROTEIN ORF1-FL49) homolog [Homo sapiens], full insert sequence. |
| AK028383 | 3830431G21Rik | 1.21 | hypothetical Pleckstrin homology (PH) domain containing protein, full insert sequence. |
| AK028614 | Pla2g15 | 1.21 | LCAT-LIKE LYSOPHOSPHOLIPASE homolog [Mus musculus], full insert sequence. |
| AK029514 | Lysmd3 | 1.21 | hypothetical protein, MGC:7041, full insert sequence. |
| AK029875 | Gm3643 | 1.21 | hypothetical protein, full insert sequence. |
| AK029955 | N/A | 1.21 | retinitis pigmentosa GTPase regulator interacting protein 1, full insert sequence. |
| AK030127 | 4932443I19Rik | 1.21 | unclassifiable, full insert sequence. |
| AK031384 | Mfsd11 | 1.21 | ET PUTATIVE TRANSLATION PRODUCT (FRAGMENT), full insert sequence. |
| AK031410 | Dlgap1 | 1.21 | DISKS LARGE-ASSOCIATED PROTEIN 1 (DAP-1) (GUANYLATE KINASE-ASSOCIATED PROTEIN) (RGKAP) (SAP90/PSD-95-ASSOCIATED PROTEIN 1) (SAPAP1) (PSD- 95/SAP90 BINDING PROTEIN 1) [Rattus norvegicus], full insert sequence. |
| AK031658 | Prei4 | 1.21 | hypothetical Glycerophosphoryl diester phosphodiesterase/Glycosyl hydrolase, starch-binding domain containing protein, full insert sequence. |
| AK031847 | Rps6kl1 | 1.21 | RIBOSOMAL PROTEIN S6 KINASE, 52KD, POLYPEPTIDE 1 [Homo sapiens], full insert sequence. |
| AK031934 | Ubap2l | 1.21 | NICE-4 PROTEIN (FRAGMENT) homolog [Homo sapiens], full insert sequence. |
| AK032079 | Faah | 1.21 | fatty acid amide hydrolase, full insert sequence. |
| AK032776 | Pgm3 | 1.21 | PHOSPHOACETYLGLUCOSAMINE MUTASE (EC 5.4.2.3) (PAGM) (ACETYLGLUCOSAMINE PHOSPHOMUTASE) (N-ACETYLGLUCOSAMINE-PHOSPHATE MUTASE) homolog [Homo sapiens], full insert sequence. |
| AK032986 | Wdr60 | 1.21 | hypothetical protein, full insert sequence. |
| AK033426 | Gm12296 | 1.21 | unclassifiable, full insert sequence. |
| AK033964 | Ptbp2 | 1.21 | polypyrimidine tract binding protein 2, full insert sequence. |
| AK034587 | Dysf | 1.21 | dysferlin, full insert sequence. |
| AK035706 | N/A | 1.21 | unclassifiable, full insert sequence. |
| AK036288 | Gm10937 | 1.21 | hypothetical protein, full insert sequence. |
| AK037932 | Irf6 | 1.21 | interferon regulatory factor 6, full insert sequence. |
| AK039294 | Lysmd4 | 1.21 | RIKEN cDNA 4930506D23 gene, full insert sequence. |
| AK039487 | A330049N07Rik | 1.21 | unclassifiable, full insert sequence. |
| AK042863 | Phf21b | 1.21 | hypothetical FYVE/PHD zinc finger structure containing protein, full insert sequence. |
| AK043092 | 2810429I04Rik | 1.21 | unclassifiable, full insert sequence. |
| AK043153 | A730060N03Rik | 1.21 | hypothetical protein, full insert sequence. |
| AK044489 | Nfatc2 | 1.21 | NUCLEAR FACTOR OF ACTIVATED T-CELLS, CYTOPLASMIC 2 (T CELL TRANSCRIPTION FACTOR NFAT1) (NFAT PRE-EXISTING SUBUNIT) (NF-ATP) |
| AK045491 | Prrt3 | 1.21 | unnamed protein product {Macaca fascicularis}, full insert sequence. |
| AK046135 | Sfi1 | 1.21 | hypothetical protein, full insert sequence. |
| AK046307 | Luc7l2 | 1.21 | CDNA FLJ10657 FIS, CLONE NT2RP2006043, WEAKLY SIMILAR TO SPLICING FACTOR, ARGININE/SERINE-RICH 4 [Homo sapiens], full insert sequence. |
| AK046702 | Mast4 | 1.21 | unclassifiable, full insert sequence. |
| AK047323 | Gm9934 | 1.21 | hypothetical protein, full insert sequence. |
| AK047511 | Gemin8 | 1.21 | HYPOTHETICAL 28.6 KDA PROTEIN [Homo sapiens], full insert sequence. |
| AK047603 | Sufu | 1.21 | suppressor of fused homolog (Drosophila), full insert sequence. |
| AK048257 | Gm2301 | 1.21 | unclassifiable, full insert sequence. |
| AK049522 | N/A | 1.21 | unclassifiable, full insert sequence. |
| AK049768 | Clpb | 1.21 | suppressor of K+ transport defect 3, full insert sequence. |
| AK050485 | 9230105E10Rik | 1.21 | TRIPARTITE MOTIF PROTEIN TRIM12 homolog [Mus musculus], full insert sequence. |
| AK050533 | 0610037L13Rik | 1.21 | hypothetical protein, full insert sequence. |
| AK051727 | 6030446N20Rik | 1.21 | hypothetical protein, full insert sequence. |
| AK052312 | Dgkd | 1.21 | hypothetical protein, full insert sequence. |
| AK052713 | Hmcn1 | 1.21 | Hypothetical Microbodies C-terminal targeting signal containing protein, full insert sequence. |
| AK053708 | Smo | 1.21 | SMOOTHENED HOMOLOG PRECURSOR (SMO) homolog [Mus musculus], full insert sequence. |
| AK053800 | N/A | 1.21 | RIKEN cDNA 2310079F23 gene, full insert sequence. |
| AK053823 | Nkain3 | 1.21 | hypothetical protein, full insert sequence. |
| AK076707 | Sox17 | 1.21 | SRY-box containing gene 17, full insert sequence. |
| AK076905 | 1700040F15Rik | 1.21 | X-LINKED LYMPHOCYTE-REGULATED PROTEIN PM1 [Mus musculus], full insert sequence. |
| AK077117 | Dyrk4 | 1.21 | DUAL-SPECIFICITY TYROSINE-PHOSPHORYLATION REGULATED KINASE 4 (EC 2.7.1.-) (FRAGMENT) [Homo sapiens], full insert sequence. |
| AK077451 | Dus2l | 1.21 | hypothetical Double-stranded RNA binding (DsRBD) domain containing protein, full insert sequence. |
| AK006511 | Dydc1 | 1.2 | hypothetical protein, full insert sequence. |
| AK007443 | 2010107H07Rik | 1.2 | hypothetical Ribosomal protein S14 containing protein, full insert sequence. |
| AK008201 | Ndufa11 | 1.2 | hypothetical protein, full insert sequence. |
| AK012700 | D3Ertd751e | 1.2 | hypothetical protein, full insert sequence. |
| AK029728 | Smok4a | 1.2 | hypothetical Tyrosine protein kinase/Serine/Threonine protein kinase/Eukaryotic protein kinase containing protein, full insert sequence. |
| AK006503 | 1700029J11Rik | 1.19 | hypothetical protein, full insert sequence. |
| AK006973 | 1700081N11Rik | 1.19 | unclassifiable, full insert sequence. |
| AK007137 | 4931431B13Rik | 1.19 | unclassifiable, full insert sequence. |
| AK008004 | Txnrd2 | 1.19 | ADULT MALE SMALL INTESTINE CDNA, RIKEN FULL-LENGTH ENRICHED LIBRARY, CLONE:2010001F03, FULL INSERT SEQUENCE, full insert sequence. |
| AK009249 | Fggy | 1.19 | CDNA FLJ10986 FIS, CLONE PLACE1001869, WEAKLY SIMILAR TO L- RIBULOKINASE (EC 2.7.1.16) (HYPOTHETICAL 27.5 KDA PROTEIN) homolog [Homo sapiens], full insert sequence. |
| AK011603 | Bcas3 | 1.19 | K20D4 (K20D4) mRNA, complete cds, full insert sequence. |
| AK014841 | 4921508A21Rik | 1.19 | PALE EAR (EP MUTANT ALLELE) (FRAGMENT) [Mus musculus], full insert sequence. |
| AK016057 | Rbm34 | 1.19 | DJ835G14.2 (KIAA0117 (HAL845) PROTEIN) (FRAGMENT) [Homo sapiens], full insert sequence. |
| AK016300 | 6530401N04Rik | 1.19 | hypothetical alpha/beta-Hydrolases structure containing protein, full insert sequence. |
| AK017144 | 5031434O11Rik | 1.19 | RIKEN full-length enriched library, clone:5031434O11 product:unclassifiable, full insert sequence. |
| AK018584 | Zfp251 | 1.19 | unclassifiable, full insert sequence. |
| AK019623 | 4930451E10Rik | 1.19 | hypothetical Prenyl group binding site (CAAX box) containing protein, full insert sequence. |
| AK019846 | Lipe | 1.19 | lipase, hormone sensitive, full insert sequence. |
| AK029792 | N/A | 1.19 | cadherin-11 {Mus musculus}, full insert sequence. |
| AK032690 | Fam92a | 1.19 | hypothetical protein, full insert sequence. |
| AK033061 | 7530428D23Rik | 1.19 | EPHRIN TYPE-A RECEPTOR 5 PRECURSOR (EC 2.7.1.112) (TYROSINE-PROTEIN KINASE RECEPTOR EHK-1) (EPH HOMOLOGY KINASE-1) (BRAIN-SPECIFIC KINASE) (CEK-7) [Mus musculus], full insert sequence. |
| AK033316 | Dazl | 1.19 | deleted in azoospermia-like, full insert sequence. |
| AK034154 | Anks1b | 1.19 | E2A-PBX1-ASSOCIATED PROTEIN (FRAGMENT) homolog [Homo sapiens], full insert sequence. |
| AK034579 | Tmem175 | 1.19 | RIKEN cDNA 3010001K23 gene, full insert sequence. |
| AK034645 | Mterfd1 | 1.19 | RIKEN cDNA 2410017I18 gene, full insert sequence. |
| AK036033 | 9630028I04Rik | 1.19 | unclassifiable, full insert sequence. |
| AK037834 | Eif3k | 1.19 | MRNA OF MUSCLE SPECIFIC GENE M9, COMPLETE CDS (ARG134 PROTEIN) homolog [Homo sapiens], full insert sequence. |
| AK039192 | Fam109a | 1.19 | hypothetical Pleckstrin homology (PH) domain containing protein, full insert sequence. |
| AK040441 | Fcgbp | 1.19 | IGG FC BINDING PROTEIN (FRAGMENT) [Homo sapiens], full insert sequence. |
| AK043846 | A730098A19Rik | 1.19 | unclassifiable, full insert sequence. |
| AK044414 | A930011G23Rik | 1.19 | hypothetical protein, full insert sequence. |
| AK044716 | Sgsm1 | 1.19 | RUN and TBC1 domain containing 2, full insert sequence. |
| AK045519 | B230208H11Rik | 1.19 | unclassifiable, full insert sequence. |
| AK046182 | N/A | 1.19 | unclassifiable, full insert sequence. |
| AK047716 | Dclk1 | 1.19 | double cortin and calcium/calmodulin-dependent protein kinase-like 1, full insert sequence. |
| AK049204 | Hps3 | 1.19 | Hermansky-Pudlak syndrome 3 homolog (human), full insert sequence. |
| AK049897 | Hook1 | 1.19 | DJ782L23.1 (HOOK1) (FRAGMENT) homolog [Homo sapiens], full insert sequence. |
| AK052145 | Pum1 | 1.19 | pumilio 1 (Drosophila), full insert sequence. |
| AK052539 | Myom1 | 1.19 | myomesin 1, full insert sequence. |
| AK052768 | Acd | 1.19 | nuclear receptor-binding SET-domain protein 1, full insert sequence. |
| AK053727 | Ppm1d | 1.19 | protein phosphatase 1D magnesium-dependent, delta isoform, full insert sequence. |
| AK076054 | Ppm1a | 1.19 | protein phosphatase 1A, magnesium dependent, alpha isoform, full insert sequence. |
| AK007148 | 1700109F18Rik | 1.18 | RIKEN cDNA 1700109F18 gene, full insert sequence. |
| AK007351 | 1810006K21Rik | 1.18 | Protein C11orf10 (HSPC005) homolog [Homo sapiens], full insert sequence. |
| AK007818 | 1810018F18Rik | 1.18 | unclassifiable, full insert sequence. |
| AK008010 | Odf3b | 1.18 | ORF1 5' TO PD-ECGF/TP PROTEIN [Homo sapiens], full insert sequence. |
| AK008630 | Htatip2 | 1.18 | HIV-1 tat interactive protein 2, 30 kDa homolog (human), full insert sequence. |
| AK008961 | Mrpl24 | 1.18 | MITOCHONDRIAL RIBOSOMAL PROTEIN L24 (FRAGMENT) homolog [Homo sapiens], full insert sequence. |
| AK010610 | Rps21 | 1.18 | 40S RIBOSOMAL PROTEIN S21 homolog [Rattus norvegicus], full insert sequence. |
| AK011283 | Snhg7 | 1.18 | unclassifiable, full insert sequence. |
| AK011427 | Pla2r1 | 1.18 | phospholipase A2, group IB, pancreas, receptor, full insert sequence. |
| AK012214 | N/A | 1.18 | peroxisomal delta3, delta2-enoyl-Coenzyme A isomerase, full insert sequence. |
| AK012381 | 2700046A07Rik | 1.18 | hypothetical protein, full insert sequence. |
| AK019127 | Tmem33 | 1.18 | DB83 PROTEIN [Rattus norvegicus], full insert sequence. |
| AK019262 | Sfpq | 1.18 | unclassifiable, full insert sequence. |
| AK019723 | 4930538L07Rik | 1.18 | unclassifiable, full insert sequence. |
| AK020460 | 9430038I01Rik | 1.18 | hypothetical protein, full insert sequence. |
| AK027910 | 1110001A16Rik | 1.18 | unclassifiable, full insert sequence. |
| AK031468 | 1700012D14Rik | 1.18 | unclassifiable, full insert sequence. |
| AK031963 | Pstpip2 | 1.18 | hypothetical protein, full insert sequence. |
| AK032132 | Runx1t1 | 1.18 | CBFA2T1 identified gene homolog (human), full insert sequence. |
| AK034170 | Klhl12 | 1.18 | KELCH-LIKE PROTEIN C3IP1 (CDNA FLJ14750 FIS, CLONE NT2RP3002948, WEAKLY SIMILAR TO RING CANAL PROTEIN) homolog [Homo sapiens], full insert sequence. |
| AK034683 | Rasgrp2 | 1.18 | RAS, guanyl releasing protein 2, full insert sequence. |
| AK036406 | Lrp4 | 1.18 | low density lipoprotein receptor-related protein 4, full insert sequence. |
| AK039886 | Tube1 | 1.18 | hypothetical protein, full insert sequence. |
| AK040168 | 2310035C23Rik | 1.18 | hypothetical ARM repeat structure containing protein, full insert sequence. |
| AK040384 | Ep300 | 1.18 | p300 protein {Homo sapiens}, full insert sequence. |
| AK040460 | Nnt | 1.18 | nicotinamide nucleotide transhydrogenase, full insert sequence. |
| AK041628 | N/A | 1.18 | unclassifiable, full insert sequence. |
| AK042476 | Sh2d1a | 1.18 | SH2 domain protein 1A, full insert sequence. |
| AK043305 | 2210406O10Rik | 1.18 | unclassifiable, full insert sequence. |
| AK044339 | Esrrb | 1.18 | estrogen related receptor, beta, full insert sequence. |
| AK044551 | Syt6 | 1.18 | synaptotagmin 6, full insert sequence. |
| AK050642 | Cald1 | 1.18 | CALDESMON (CDM) homolog [Homo sapiens], full insert sequence. |
| AK052749 | Sypl2 | 1.18 | mitsugumin 29, full insert sequence. |
| AK053345 | Lrrtm1 | 1.18 | hypothetical RNI-like structure containing protein, full insert sequence. |
| AK053861 | Opa1 | 1.18 | Dynamin family containing protein, full insert sequence. |
| AK075861 | Sirt3 | 1.18 | SIR2L3 isoform B (Sir2L3) mRNA, full insert sequence. |
| AK077472 | Erp29 | 1.18 | unclassifiable, full insert sequence. |
| AK007558 | 1810020D17Rik | 1.17 | PTD015 [Homo sapiens], full insert sequence. |
| AK008046 | Abhd5 | 1.17 | unclassifiable, full insert sequence. |
| AK008317 | Dpagt1 | 1.17 | dolichyl-phosphate alpha-N-acetylglucosaminephosphotransferase 2, full insert sequence. |
| AK008466 | Fau | 1.17 | Finkel-Biskis-Reilly murine sarcoma virus (FBR-MuSV) ubiquitously expressed (fox derived), full insert sequence. |
| AK009067 | Fam100b | 1.17 | unclassifiable, full insert sequence. |
| AK009698 | Ndufab1 | 1.17 | ACYL CARRIER PROTEIN (ACP), full insert sequence. |
| AK010044 | 2310065F04Rik | 1.17 | unclassifiable, full insert sequence. |
| AK010427 | 2410006H16Rik | 1.17 | unclassifiable, full insert sequence. |
| AK012970 | Dpf3 | 1.17 | cerebellum D4, full insert sequence. |
| AK014004 | Srpk2 | 1.17 | serine/arginine-rich protein specific kinase 2, full insert sequence. |
| AK014905 | Ttll7 | 1.17 | hypothetical Tubulin-tyrosine ligase containing protein, full insert sequence. |
| AK015475 | 4930405A10Rik | 1.17 | unclassifiable, full insert sequence. |
| AK016790 | Orly | 1.17 | RIKEN cDNA 4933412F11 gene, full insert sequence. |
| AK017465 | Tiam1 | 1.17 | T-cell lymphoma invasion and metastasis 1, full insert sequence. |
| AK017891 | 5830403F22Rik | 1.17 | unclassifiable, full insert sequence. |
| AK018310 | Prdm5 | 1.17 | zinc-finger protein (ZNFpT7) (fragment) [Homo sapiens], full insert sequence. |
| AK019022 | Foxa3 | 1.17 | forkhead box A3, full insert sequence. |
| AK019154 | 2610017I09Rik | 1.17 | hypothetical protein, full insert sequence. |
| AK019370 | Extl2 | 1.17 | exotoses (multiple)-like 2, full insert sequence. |
| AK020013 | Ctnnb1 | 1.17 | catenin beta, full insert sequence. |
| AK020506 | 9430085M18Rik | 1.17 | unclassifiable, full insert sequence. |
| AK021197 | C330013E15Rik | 1.17 | unclassifiable, full insert sequence. |
| AK031921 | Tmem138 | 1.17 | hypothetical protein, full insert sequence. |
| AK032081 | Pmpca | 1.17 | MITOCHONDRIAL PROCESSING PEPTIDASE ALPHA SUBUNIT, MITOCHONDRIAL PRECURSOR (EC 3.4.24.64) (ALPHA-MPP) (P-55) homolog [Rattus norvegicus], full insert sequence. |
| AK043975 | Snx16 | 1.17 | SORTING NEXIN 16 [Rattus norvegicus], full insert sequence. |
| AK047062 | Supt5h | 1.17 | suppressor of Ty 5 homolog (S. cerevisiae), full insert sequence. |
| AK049707 | Dido1 | 1.17 | DJ885L7.9.1 (DEATH ASSOCIATED TRANSCRIPTION FACTOR 1 (CONTAINS KIAA0333), ISOFORM 1) (FRAGMENT) [Homo sapiens], full insert sequence. |
| AK007373 | A930005H10Rik | 1.16 | RIKEN cDNA A930005H10 gene, full insert sequence. |
| AK007757 | Mtch2 | 1.16 | mitochondrial carrier homolog 2, full insert sequence. |
| AK008341 | 2010107H07Rik | 1.16 | hypothetical Ribosomal protein S14 containing protein, full insert sequence. |
| AK010703 | Prkag2 | 1.16 | AMP ACTIVATED PROTEIN KINASE GAMMA 1 (FRAGMENT) [Mus musculus], full insert sequence. |
| AK010773 | Atg16l2 | 1.16 | hypothetical G-protein beta WD-40 repeats containing protein, full insert sequence. |
| AK010874 | Rab43 | 1.16 | RAB19-LIKE (FRAGMENT) [Caenorhabditis elegans], full insert sequence. |
| AK011892 | Plcd3 | 1.16 | PHOSPHOLIPASE C, DELTA [Homo sapiens], full insert sequence. |
| AK013123 | 4921524J17Rik | 1.16 | hypothetical protein, full insert sequence. |
| AK014174 | Chka | 1.16 | choline kinase, full insert sequence. |
| AK016952 | Gnb1l | 1.16 | guanine nucleotide binding protein (G protein), beta polypeptide 1-like, full insert sequence. |
| AK031365 | Msi2 | 1.16 | Musashi homolog 2 (Drosophila), full insert sequence. |
| AK035588 | Bspry | 1.16 | PUTATIVE NUCLEAR FACTOR homolog [Mus musculus], full insert sequence. |
| AK037997 | Gripap1 | 1.16 | GRIP-ASSOCIATED PROTEIN 1 SHORT FORM [Rattus norvegicus], full insert sequence. |
| AK038505 | Pnpla7 | 1.16 | NEUROPATHY target esterase, full insert sequence. |
| AK038786 | Pbx4 | 1.16 | pre-B-cell leukemia transcription factor 4, full insert sequence. |
| AK039284 | A830011K09Rik | 1.16 | unclassifiable, full insert sequence. |
| AK041315 | Alpk1 | 1.16 | LYMPHOCYTE ALPHA-KINASE homolog [Homo sapiens], full insert sequence. |
| AK043553 | Asphd1 | 1.16 | hypothetical protein, full insert sequence. |
| AK043789 | N/A | 1.16 | ENV POLYPROTEIN PRECURSOR (COAT POLYPROTEIN) [CONTAINS: SURFACE PROTEIN; TRANSMEMBRANE PROTEIN] |
| AK043958 | N/A | 1.16 | Mus musculus 10 days neonate cortex cDNA, RIKEN full-length enriched library, clone:A830059M14 product:unclassifiable, full insert sequence. |
| AK044786 | A230108P19Rik | 1.16 | unclassifiable, full insert sequence. |
| AK045601 | Rfwd3 | 1.16 | Peptidase S9A, prolyl oligopeptidase, N-terminal beta-propeller domain containing protein, full insert sequence. |
| AK046443 | A130049A11Rik | 1.16 | putative homeodomain transcription factor, full insert sequence. |
| AK046670 | Phf20l1 | 1.16 | CDNA: FLJ21615 FIS, CLONE COL07393 [Homo sapiens], full insert sequence. |
| AK049264 | C330018A13Rik | 1.16 | unclassifiable, full insert sequence. |
| AK049326 | Cep70 | 1.16 | P10-BINDING PROTEIN [Homo sapiens], full insert sequence. |
| AK051008 | D030054H15Rik | 1.16 | unclassifiable, full insert sequence. |
| AK052441 | Tubgcp6 | 1.16 | hypothetical Phospholipase A2 containing protein, full insert sequence. |
| AK052980 | Ltbp2 | 1.16 | latent transforming growth factor beta binding protein 2, full insert sequence. |
| AK053296 | E130008D07Rik | 1.16 | unclassifiable, full insert sequence. |
| AK076000 | Skap2 | 1.16 | SRC-ASSOCIATED ADAPTOR PROTEIN homolog [Homo sapiens], full insert sequence. |
| AK007615 | Mrpl51 | 1.15 | mitochondrial ribosomal protein 64, full insert sequence. |
| AK015208 | 4933406K04Rik | 1.15 | unclassifiable, full insert sequence. |
| AK015772 | 4930512H18Rik | 1.15 | RIKEN cDNA 4930512H18 gene, full insert sequence. |
| AK016343 | 4930583K01Rik | 1.15 | hypothetical ATP/GTP-binding site motif A (P-loop) containing protein, full insert sequence. |
| AK018458 | Cyp2u1 | 1.15 | RAT CYTOCHROME P-450B TYPE E (FRAGMENT) [Rattus norvegicus], full insert sequence. |
| AK028859 | Tor1b | 1.15 | torsin family 1, member B, full insert sequence. |
| AK029086 | 4732490B19Rik | 1.15 | unclassifiable, full insert sequence. |
| AK029303 | Atp13a4 | 1.15 | E1-E2 ATPases/Haloacid dehalogenase/epoxide hydrolase family containing protein, full insert sequence. |
| AK034539 | C730027H18Rik | 1.15 | unclassifiable, full insert sequence. |
| AK044186 | Pex26 | 1.15 | KAIA2502 PROTEIN [Homo sapiens], full insert sequence. |
| AK045112 | B130034C11Rik | 1.15 | unclassifiable, full insert sequence. |
| AK047271 | Bicc1 | 1.15 | bicaudal C homolog 1 (Drosophila), full insert sequence. |
| AK048515 | Tle1 | 1.15 | TRANSDUCIN-LIKE ENHANCER PROTEIN 1 (GROUCHO-RELATED PROTEIN 1) homolog [Mus musculus], full insert sequence. |
| AK052773 | D630048o14rik | 1.15 | unclassifiable, full insert sequence. |
| AK053804 | Rgs12 | 1.15 | G-protein signaling regulator 12 homolog [Rattus norvegicus], full insert sequence. |
| AK076943 | Pias4 | 1.15 | protein inhibitor of activated STAT gamma, full insert sequence. |
| AK006321 | 1700025B11Rik | 1.14 | unclassifiable, full insert sequence. |
| AK006782 | Nipsnap3a | 1.14 | NIPSNAP4 PROTEIN (MGC:14553) (DKFZP564D177) (FLJ13953) (HSPC299) [Homo sapiens], full insert sequence. |
| AK007293 | Pkd1l2 | 1.14 | hypothetical protein, full insert sequence. |
| AK007836 | N/A | 1.14 | unclassifiable, full insert sequence. |
| AK008432 | 2010203P06Rik | 1.14 | unclassifiable, full insert sequence. |
| AK009351 | 2310015B20Rik | 1.14 | hypothetical protein, full insert sequence. |
| AK011194 | Pole2 | 1.14 | polymerase (DNA directed), epsilon 2 (p59 subunit), full insert sequence. |
| AK011687 | 2610036A22Rik | 1.14 | BC39498_3 [Homo sapiens], full insert sequence. |
| AK013126 | Mrpl38 | 1.14 | mitochondrial ribosomal protein L38, full insert sequence. |
| AK013290 | 2810442I21Rik | 1.14 | unclassifiable, full insert sequence. |
| AK015271 | Zdbf2 | 1.14 | Mus musculus adult male testis cDNA, RIKEN full-lenghypothetical protein, full insert sequence. |
| AK018713 | Cyba | 1.14 | cytochrome b-245, alpha polypeptide, full insert sequence. |
| AK018745 | Zfp787 | 1.14 | RIKEN cDNA 2210018M03 gene, full insert sequence. |
| AK019009 | Pla2g1b | 1.14 | phospholipase A2, group IB, pancreas, full insert sequence. |
| AK020011 | 5830415L20Rik | 1.14 | 5830415L20RIK PROTEIN, full insert sequence. |
| AK020895 | Gnas | 1.14 | unclassifiable, full insert sequence. |
| AK021162 | C130038G02Rik | 1.14 | hypothetical Glutamine-rich region containing protein, full insert sequence. |
| AK021325 | D730003I15Rik | 1.14 | hypothetical protein, full insert sequence. |
| AK030238 | 1700025I17Rik | 1.14 | unclassifiable, full insert sequence. |
| AK034798 | Haghl | 1.14 | hypothetical Metallo-beta-lactamase superfamily containing protein, full insert sequence. |
| AK035371 | Ube2k | 1.14 | huntingtin interacting protein 2, full insert sequence. |
| AK035728 | Ccnc | 1.14 | cyclin C, full insert sequence. |
| AK035975 | Disp2 | 1.14 | KIAA1742 PROTEIN (FRAGMENT) [Homo sapiens], full insert sequence. |
| AK038701 | Pigz | 1.14 | unclassifiable, full insert sequence. |
| AK038992 | N/A | 1.14 | hypothetical protein, full insert sequence. |
| AK039576 | Kif13a | 1.14 | kinesin 13A, full insert sequence. |
| AK041267 | N/A | 1.14 | unclassifiable, full insert sequence. |
| AK045173 | Suhw4 | 1.14 | DJ322P7.1 (ZINC FINGER) (FRAGMENT) [Homo sapiens], full insert sequence. |
| AK045962 | Cdadc1 | 1.14 | PROTEIN KINASE NYD-SP15 homolog [Mus musculus], full insert sequence. |
| AK048244 | Ccnl2 | 1.14 | CYCLIN ANIA-6A [Rattus norvegicus], full insert sequence. |
| AK048685 | Uggt2 | 1.14 | UDP-GLUCOSE:GLYCOPROTEIN GLUCOSYLTRANSFERASE 2 PRECURSOR [Homo sapiens], full insert sequence. |
| AK050963 | Ddc | 1.14 | dopa decarboxylase, full insert sequence. |
| AK053037 | Heatr5b | 1.14 | ARM repeat structure containing protein, full insert sequence. |
| AK053193 | E030030I06Rik | 1.14 | CYCLIN-DEPENDENT KINASE 6 (FRAGMENT) [Rattus norvegicus], full insert sequence. |
| AK053578 | Rassf4 | 1.14 | AD037 [Homo sapiens], full insert sequence. |
| AK076883 | 1700023L04Rik | 1.14 | unclassifiable, full insert sequence. |
| AK077136 | Ppm1b | 1.14 | protein phosphatase 1B, magnesium dependent, beta isoform, full insert sequence. |
| AK006304 | 1700024I08Rik | 1.13 | unclassifiable, full insert sequence. |
| AK008222 | 2010013B24Rik | 1.13 | unclassifiable, full insert sequence. |
| AK009175 | Snhg12 | 1.13 | unclassifiable, full insert sequence. |
| AK010054 | Fgf22 | 1.13 | fibroblast growth factor 22, full insert sequence. |
| AK012733 | Mtif3 | 1.13 | DC38 (MITOCHONDRIAL TRANSLATIONAL INITIATION FACTOR 3) [Homo sapiens], full insert sequence. |
| AK013308 | C130071C03Rik | 1.13 | unclassifiable, full insert sequence. |
| AK014969 | 4921525O09Rik | 1.13 | unclassifiable, full insert sequence. |
| AK015878 | Slc31a2 | 1.13 | solute carrier family 31, member 2, full insert sequence. |
| AK016163 | N/A | 1.13 | unclassifiable, full insert sequence. |
| AK016557 | Mllt4 | 1.13 | myeloid/lymphoid or mixed lineage-leukemia translocation to 4 homolog (Drosophila), full insert sequence. |
| AK017087 | Cpvl | 1.13 | CARBOXYPEPTIDASE, VITELLOGENIC-LIKE [Homo sapiens], full insert sequence. |
| AK019420 | Csnk1a1 | 1.13 | Mus musculus 13 days embryo head cDNA, RIKEN full-length enriched library, clone:311unclassifiable, full insert sequence. |
| AK020043 | 5930435M05Rik | 1.13 | unclassifiable, full insert sequence. |
| AK020083 | Nat9 | 1.13 | hypothetical protein, full insert sequence. |
| AK020812 | A930005H10Rik | 1.13 | hypothetical protein, full insert sequence. |
| AK028224 | N/A | 1.13 | unclassifiable, full insert sequence. |
| AK029949 | 4933409K07Rik | 1.13 | hypothetical protein, full insert sequence. |
| AK031165 | Cnot3 | 1.13 | unclassifiable, full insert sequence. |
| AK031278 | Ccdc28b | 1.13 | unclassifiable, full insert sequence. |
| AK033289 | Mycbp | 1.13 | c-myc binding protein, full insert sequence. |
| AK034995 | Cdh13 | 1.13 | cadherin 13, full insert sequence. |
| AK035122 | Zmym6 | 1.13 | hypothetical Microbodies C-terminal targeting signal containing protein, full insert sequence. |
| AK035400 | Baz2a | 1.13 | bromodomain adjacent to zinc finger domain, 2A, full insert sequence. |
| AK036614 | March2 | 1.13 | HSPC240 [Homo sapiens], full insert sequence. |
| AK038627 | N/A | 1.13 | heat shock 70 kDa protein 4, full insert sequence. |
| AK039440 | Polr3a | 1.13 | hypothetical protein, full insert sequence. |
| AK039594 | Pitpnm3 | 1.13 | NIR1 [Homo sapiens], full insert sequence. |
| AK039819 | A930013B10Rik | 1.13 | hypothetical protein, full insert sequence. |
| AK041795 | N/A | 1.13 | unclassifiable, full insert sequence. |
| AK042657 | Cdh7 | 1.13 | hypothetical Cadherin structure containing protein, full insert sequence. |
| AK043122 | 4930579E17Rik | 1.13 | hypothetical 4-diphosphocytidyl-2C-methyl-D-erythritol synthase containing protein, full insert sequence. |
| AK043473 | Ece2 | 1.13 | ENDOTHELIN CONVERTING ENZYME-2 homolog [Mus musculus], full insert sequence. |
| AK045744 | Tmem181d-ps | 1.13 | unclassifiable, full insert sequence. |
| AK047723 | C030017K20Rik | 1.13 | unclassifiable, full insert sequence. |
| AK047841 | Mbtps2 | 1.13 | MEMBRANE-BOUND TRANSCRIPTION FACTOR SITE 2 PROTEASE (EC 3.4.24.-) (SITE-2 PROTEASE) (STEROL-REGULATORY ELEMENT-BINDING PROTEINS INTRAMEMBRANE PROTEASE) homolog [Homo sapiens], full insert sequence. |
| AK048250 | Cdc14a | 1.13 | CDC14A3 PHOSPHATASE [Homo sapiens], full insert sequence. |
| AK053889 | Pomt1 | 1.13 | protein O-mannosyltransferase 1 {Rattus norvegicus}, full insert sequence. |
| AK053906 | Lig1 | 1.13 | ligase I, DNA, ATP-dependent, full insert sequence. |
| AK075939 | Card11 | 1.13 | CASPASE RECRUITMENT DOMAIN PROTEIN 11 (CARD-CONTAINING MAGUK PROTEIN 3) (CARMA 1) homolog [Homo sapiens], full insert sequence. |
| AK076052 | Arpp19 | 1.13 | unclassifiable, full insert sequence. |
| AK076622 | N/A | 1.13 | unclassifiable, full insert sequence. |
| BC099946 | 9930104L06Rik | 1.12 | RIKEN cDNA 9930104L06 gene, mRNA (cDNA clone MGC:107426 IMAGE:30102988), complete cds. |
| AK006422 | Myl10 | 1.12 | myosin light chain 2, precursor lymphocyte-specific, full insert sequence. |
| AK006734 | 1700048O20Rik | 1.12 | unclassifiable, full insert sequence. |
| AK006996 | 1700065I16Rik | 1.12 | unclassifiable, full insert sequence. |
| AK007495 | Ndufb2 | 1.12 | NADH-UBIQUINONE OXIDOREDUCTASE AGGG SUBUNIT homolog [Homo sapiens], full insert sequence. |
| AK007912 | Cuta | 1.12 | CICK0721Q.5 (POLYPEPTIDE FROM PATENTED CDNA EMBL:E06811) (DIVALENT CATION TOLERANT PROTEIN CUTA) [Homo sapiens], full insert sequence. |
| AK008611 | Prr13 | 1.12 | UNCHARACTERIZED BONE MARROW PROTEIN BM041 (DKFZP564J157 PROTEIN) (SIMILAR TO DKFZP564J157 PROTEIN) [Homo sapiens], full insert sequence. |
| AK008984 | 2210418O10Rik | 1.12 | DNA-BINDING PROTEIN (FRAGMENT) [Homo sapiens], full insert sequence. |
| AK009785 | 2310043M15Rik | 1.12 | unclassifiable, full insert sequence. |
| AK010403 | 2410004N09Rik | 1.12 | unclassifiable, full insert sequence. |
| AK011905 | Ncam1 | 1.12 | hypothetical protein, full insert sequence. |
| AK012380 | 2700045P11Rik | 1.12 | hypothetical protein, full insert sequence. |
| AK012393 | Sec61g | 1.12 | SEC61, gamma subunit (S. cerevisiae), full insert sequence. |
| AK012698 | Smn1 | 1.12 | survival motor neuron, full insert sequence. |
| AK012732 | Secisbp2 | 1.12 | SECIS-BINDING PROTEIN 2 homolog [Rattus norvegicus], full insert sequence. |
| AK012787 | Yaf2 | 1.12 | YY1 associated factor 2, full insert sequence. |
| AK014046 | Cenpv | 1.12 | hypothetical protein, full insert sequence. |
| AK014270 | 3110082D06Rik | 1.12 | hypothetical protein, full insert sequence. |
| AK015218 | Chd1 | 1.12 | chromodomain helicase DNA binding protein 1, full insert sequence. |
| AK015817 | 4930517N10Rik | 1.12 | hypothetical protein, full insert sequence. |
| AK016926 | 4933426D04Rik | 1.12 | hypothetical protein, full insert sequence. |
| AK017040 | 4933433G15Rik | 1.12 | unclassifiable, full insert sequence. |
| AK018500 | Zfp655 | 1.12 | unclassifiable, full insert sequence. |
| AK019701 | 4930505M18Rik | 1.12 | unclassifiable, full insert sequence. |
| AK019801 | 4933406F09Rik | 1.12 | unclassifiable, full insert sequence. |
| AK020320 | Gm9897 | 1.12 | unclassifiable, full insert sequence. |
| AK020722 | Qk | 1.12 | unclassifiable, full insert sequence. |
| AK021276 | Spink8 | 1.12 | hypothetical Ovomucoid/PCI-1 like inhibitors structure containing protein, full insert sequence. |
| AK028180 | 3632451O06Rik | 1.12 | RIKEN cDNA 3632451O06 gene, full insert sequence. |
| AK028394 | 1190007F08Rik | 1.12 | unclassifiable, full insert sequence. |
| AK029776 | Kif9 | 1.12 | Kinesin superfamily protein 9, full insert sequence. |
| AK030112 | Cnot6l | 1.12 | hypothetical Leucine-rich repeat, typical subtype containing protein, full insert sequence. |
| AK030530 | Gpr158 | 1.12 | hypothetical G-protein coupled receptors family 3 (Metabotropic glutamate receptor-like) containing protein, full insert sequence. |
| AK030632 | Zfp14 | 1.12 | zinc finger protein 14, full insert sequence. |
| AK031337 | Fads2 | 1.12 | fatty acid desaturase 2, full insert sequence. |
| AK031668 | 6430562O15Rik | 1.12 | unclassifiable, full insert sequence. |
| AK032251 | Wdr18 | 1.12 | HYPOTHETICAL 48.1 KDA PROTEIN (FRAGMENT) homolog [Homo sapiens], full insert sequence. |
| AK032644 | Psmf1 | 1.12 | PROTEASOME INHIBITOR PI31 SUBUNIT (HPI31) [Homo sapiens], full insert sequence. |
| AK034780 | Zic3 | 1.12 | zinc finger protein of the cerebellum 3, full insert sequence. |
| AK035315 | Ocrl | 1.12 | LOWE OCULOCEREBRORENAL SYNDROME PROTEIN homolog [Homo sapiens], full insert sequence. |
| AK036056 | 5430435G22Rik | 1.12 | RAB7, MEMBER RAS ONCOGENE FAMILY [Homo sapiens], full insert sequence. |
| AK036757 | Ccdc64 | 1.12 | hypothetical protein, full insert sequence. |
| AK037348 | Phc3 | 1.12 | polyhomeotic-like 3 (Drosophila), full insert sequence. |
| AK037770 | Gm15623 | 1.12 | hypothetical protein, full insert sequence. |
| AK038021 | D330041H03Rik | 1.12 | hypothetical protein, full insert sequence. |
| AK038915 | Med15 | 1.12 | positive cofactor 2, multiprotein complex, glutamine/Q-rich-associated protein, full insert sequence. |
| AK039184 | Zfp783 | 1.12 | WUGSC:H_DJ0800G07.4 PROTEIN (FRAGMENT) [Homo sapiens], full insert sequence. |
| AK039953 | Pglyrp2 | 1.12 | peptidoglycan recognition protein-like, full insert sequence. |
| AK040575 | LOC100270707 | 1.12 | unclassifiable, full insert sequence. |
| AK040736 | Fkbp8 | 1.12 | FK506 binding protein 8 (38 kDa), full insert sequence. |
| AK041609 | Epm2a | 1.12 | hypothetical Glycosyl hydrolase, starch-binding domain containing protein, full insert sequence. |
| AK042186 | Arid4a | 1.12 | RETINOBLASTOMA-BINDING PROTEIN 1 (RBBP-1) homolog [Homo sapiens], full insert sequence. |
| AK044651 | Cyfip1 | 1.12 | INDUCIBLE PROTEIN (FRAGMENT) homolog [Mus musculus], full insert sequence. |
| AK044872 | Camk2d | 1.12 | calcium/calmodulin-dependent protein kinase II, delta, full insert sequence. |
| AK045413 | 1190007I07Rik | 1.12 | RIKEN cDNA 1810014B01 gene, full insert sequence. |
| AK046172 | AW495222 | 1.12 | unclassifiable, full insert sequence. |
| AK046517 | Stat1 | 1.12 | signal transducer and activator of transcription 1, full insert sequence. |
| AK046628 | Mtss1 | 1.12 | METASTASIS SUPPRESSOR PROTEIN homolog [Homo sapiens], full insert sequence. |
| AK046920 | Asb7 | 1.12 | unclassifiable, full insert sequence. |
| AK047944 | Taf6 | 1.12 | TAF6 RNA polymerase II, TATA box binding protein (TBP)-associated factor, 80 kDa, full insert sequence. |
| AK049588 | Nipbl | 1.12 | hypothetical Glutamine-rich region containing protein, full insert sequence. |
| AK051324 | Fignl1 | 1.12 | fidgetin-like 1, full insert sequence. |
| AK051834 | Mtrf1 | 1.12 | PEPTIDE CHAIN RELEASE FACTOR 1, MITOCHONDRIAL PRECURSOR (MRF-1) [Homo sapiens], full insert sequence. |
| AK052408 | Bat2d | 1.12 | hypothetical protein, full insert sequence. |
| AK052878 | N/A | 1.12 | unclassifiable, full insert sequence. |
| AK054341 | Slc38a6 | 1.12 | transient receptor protein 7, full insert sequence. |
| AK076700 | 4930417O22Rik | 1.12 | hypothetical protein, full insert sequence. |
| AK077286 | Pelo | 1.12 | hypothetical protein, full insert sequence. |
| AK077481 | N/A | 1.12 | unclassifiable, full insert sequence. |
| AK007015 | Hrasls5 | 1.11 | Mus musculus adult male testis cDNA, RIKEN full-length enriched library, clone:1700086E08 product:1700086E08RIK PROTEIN (FRAGMENT) homolog [Mus musculus], full insert sequence. |
| AK007770 | 1810044D09Rik | 1.11 | unclassifiable, full insert sequence. |
| AK007998 | 2010001A14Rik | 1.11 | unclassifiable, full insert sequence. |
| AK008207 | Acbd4 | 1.11 | hypothetical protein, full insert sequence. |
| AK009603 | 0610040B10Rik | 1.11 | unclassifiable, full insert sequence. |
| AK010285 | Nkiras1 | 1.11 | KAPPA B-RAS 2 (I-KAPPA-B-INTERACTING RAS-LIKE PROTEIN 2) [Homo sapiens], full insert sequence. |
| AK011342 | Atp5k | 1.11 | ATP synthase, H+ transporting, mitochondrial F1F0 complex, subunit e, full insert sequence. |
| AK012595 | 2700090O03Rik | 1.11 | unclassifiable, full insert sequence. |
| AK012857 | Igsf3 | 1.11 | RIKEN cDNA 2810035F16 gene, full insert sequence. |
| AK012920 | Nudt16 | 1.11 | CDNA FLJ31265 FIS, CLONE KIDNE2006030, MODERATELY SIMILAR TO GALLUS GALLUS SYNDESMOS MRNA [Homo sapiens], full insert sequence. |
| AK013548 | Rasgrp1 | 1.11 | RAS guanyl releasing protein 1, full insert sequence. |
| AK013693 | D10Ertd322e | 1.11 | MITOCHONDRIAL 28S RIBOSOMAL PROTEIN S32 (MRP-S32) [Homo sapiens], full insert sequence. |
| AK013883 | 1810013L24Rik | 1.11 | RIKEN cDNA 3010002C02 gene, full insert sequence. |
| AK014541 | Vti1a | 1.11 | vesicle transport through interaction with t-SNAREs 1 homolog, full insert sequence. |
| AK015657 | 2810442I21Rik | 1.11 | unclassifiable, full insert sequence. |
| AK015969 | AV039307 | 1.11 | unclassifiable, full insert sequence. |
| AK016290 | Yeats4 | 1.11 | glioma-amplified sequence-41, full insert sequence. |
| AK019124 | 2410080I02Rik | 1.11 | hypothetical protein, full insert sequence. |
| AK019229 | 2700046G09Rik | 1.11 | hypothetical protein, full insert sequence. |
| AK019339 | 2900016B01Rik | 1.11 | unclassifiable, full insert sequence. |
| AK028130 | Magi2 | 1.11 | hypothetical protein, full insert sequence. |
| AK028679 | Asph | 1.11 | aspartate-beta-hydroxylase, full insert sequence. |
| AK029544 | Ccdc81 | 1.11 | RIKEN cDNA 4921513D09 gene, full insert sequence. |
| AK029890 | Usp20 | 1.11 | ubiquitin specific protease 20, full insert sequence. |
| AK030206 | Gm13112 | 1.11 | unclassifiable, full insert sequence. |
| AK031787 | Pkmyt1 | 1.11 | membrane-associated tyrosine-and threonine-specific cdc2-inhibitory kinase, full insert sequence. |
| AK033049 | A730085K08Rik | 1.11 | unclassifiable, full insert sequence. |
| AK034133 | Slc29a3 | 1.11 | EQUILIBRATIVE NUCLEOSIDE TRANSPORTER 3, full insert sequence. |
| AK034749 | 4933434E20Rik | 1.11 | RIKEN cDNA 5730552F22 gene, full insert sequence. |
| AK036843 | LOC623078 | 1.11 | unclassifiable transcript, full insert sequence. |
| AK038306 | LOC545086 | 1.11 | putative {Mus musculus}, full insert sequence. |
| AK039866 | Pank4 | 1.11 | FANG1 homolog [Rattus norvegicus], full insert sequence. |
| AK040174 | Ect2 | 1.11 | ect2 oncogene, full insert sequence. |
| AK040289 | D18Ertd653e | 1.11 | PROTEIN C18ORF1 homolog [Homo sapiens], full insert sequence. |
| AK041755 | N/A | 1.11 | cadherin 1, full insert sequence. |
| AK042346 | Med23 | 1.11 | TRANSCRIPTIONAL CO-ACTIVATOR CRSP130 (FRAGMENT) [Homo sapiens], full insert sequence. |
| AK044888 | Jarid2 | 1.11 | jumonji, full insert sequence. |
| AK046382 | Neurl1a | 1.11 | neuralized homolog (Drosophila), full insert sequence. |
| AK046465 | Dhrs13 | 1.11 | Hypothetical short-chain dehydrogenase/reductase homolog [Mus musculus], full insert sequence. |
| AK047522 | Adc | 1.11 | ORNITHINE DECARBOXYLASE-LIKE PROTEIN VARIANT 1 (SIMILAR TO ORNITHINE DECARBOXYLASE 1) homolog [Homo sapiens], full insert sequence. |
| AK048828 | N/A | 1.11 | RIKEN cDNA 1700047I17 gene, full insert sequence. |
| AK049059 | Lrrc16a | 1.11 | hypothetical RNI-like structure containing protein, full insert sequence. |
| AK049320 | Dock6 | 1.11 | CDNA FLJ30839 FIS, CLONE FEBRA2002429 [Homo sapiens], full insert sequence. |
| AK049940 | Gucy2g | 1.11 | KINASE-LIKE DOMAIN CONTAINING SOLUBLE GUANYLYL CYCLASE (EC 4.6.1.2) (48 KDA CHAIN) (KSGC) homolog [Rattus norvegicus], full insert sequence. |
| AK050492 | Mettl13 | 1.11 | CGI-01 PROTEIN homolog [Homo sapiens], full insert sequence. |
| AK051750 | 1110012J17Rik | 1.11 | hypothetical protein, full insert sequence. |
| AK052884 | Asb14 | 1.11 | ankyrin repeat domain-containing SOCS box protein 14, full insert sequence. |
| AK053198 | Papln | 1.11 | papilin mRNA, full insert sequence. |
| AK076749 | Rpusd3 | 1.11 | Pseudouridine synthase containing protein, full insert sequence. |
| AK077492 | N/A | 1.11 | unclassifiable, full insert sequence. |
| NM_001286099.1 | GPR6 | 1.1 | G protein-coupled receptor 6 |
| NM_182691.2 | SRPK2 | 1.1 | SRSF protein kinase 2 |
| AK006355 | 1700025L06Rik | 1.1 | unclassifiable, full insert sequence. |
| AK009205 | N/A | 1.1 | protein phosphatase 1, regulatory (inhibitor) subunit 2, full insert sequence. |
| AK012589 | Zcrb1 | 1.1 | MADP-1 PROTEIN homolog [Homo sapiens], full insert sequence. |
| AK014460 | 3930402G23Rik | 1.1 | hypothetical protein, full insert sequence. |
| AK019348 | 2900060B14Rik | 1.1 | unclassifiable, full insert sequence. |
| AK034780 | Zic3 | 1.09 | Zinc finger protein of the cerebellum 3, full insert sequence. |
| BC140314 | Hrh1 | 1.09 | Histamine receptor H 1 (Hrh1) mRNA, encodes complete protein. |
| BC145807 | Pgr | 1.09 | Progesterone receptor, mRNA (cDNA clone MGC:175694 IMAGE:40131110), complete cds. |
| BC152773 | Cbx6-Nptxr | 1.09 | Neuronal pentraxin with chromo domain (Npcd) mRNA, encodes complete protein. |
| BC156134 | Pax5 | 1.09 | Paired box gene 5 (Pax5) mRNA, encodes complete protein. |
| NG_015841.1 | TPM4 | 1.09 | tropomyosin 4 |
| NG_009303.1 | SMARCB1 | 1.09 | SWI/SNF related, matrix associated, actin dependent regulator of chromatin, subfamily b, member 1 |
| NG_008621.1 | TPM3 | 1.09 | tropomyosin 3 |
| NG_045229.1 | NAP1L5 | 1.09 | nucleosome assembly protein 1-like 5 |
| NG_027876.1 | WDR1 | 1.09 | WD repeat domain 1 |
| NM_003299.2 | HSP90B1 | 1.09 | heat shock protein 90kDa beta (Grp94), member 1 |
| NM_003203.4 | GCFC2 | 1.09 | GC-rich sequence DNA-binding factor 2 |
| NG_028279.1 | MECOM | 1.09 | MDS1 and EVI1 complex locus |
| NG_047204.1 | COL25A1 | 1.09 | collagen, type XXV, alpha 1 |
| NM_032826.4 | SLC35B4 | 1.09 | solute carrier family 35 (UDP-xylose/UDP-N-acetylglucosamine transporter), member B4 |
| NM_001110209 | Lnp | 1.09 | limb and neural patterns (Lnp), transcript variant 2, mRNA. |
| NM_010399 | H2-T9 | 1.09 | histocompatibility 2, T region locus 9 (H2-T9), mRNA. |
| NR_003559 | Mrpl48 | 1.09 | mitochondrial ribosomal protein L48 (Mrpl48), transcribed RNA. |
| XM_001001226 | C2cd4d | 1.09 | gene model 659, (NCBI) (Gm659), mRNA. |
| AJ271477 | Scn5a | 1.09 | voltage-gated sodium channel (Scn5a gene). |
| AK005989 | Pdia6 | 1.09 | protein disulfide-isomerase homolog P5 precursor (fragment) homolog [Rattus norvegicus], full insert sequence. |
| AK006422 | Myl10 | 1.09 | myosin light chain 2, precursor lymphocyte-specific, full insert sequence. |
| AK008222 | 2010013B24Rik | 1.09 | unclassifiable, full insert sequence. |
| AK011205 | Rbms1 | 1.09 | unclassifiable, full insert sequence. |
| AK011630 | Set | 1.09 | SET translocation, full insert sequence. |
| AK012880 | 6820431F20Rik | 1.09 | similar to CADHERIN (FRAGMENT) [Gallus gallus], full insert sequence. |
| AK015921 | Sv2c | 1.09 | weakly similar to SYNAPTIC VESICLE PROTEIN 2C [Rattus norvegicus], full insert sequence. |
| AK017087 | Cpvl | 1.09 | similar to CARBOXYPEPTIDASE, VITELLOGENIC-LIKE [Homo sapiens], full insert sequence. |
| AK018993 | E030003E18Rik | 1.09 | unclassifiable, full insert sequence. |
| AK019122 | Cops7a | 1.09 | COP9 (constitutive photomorphogenic) homolog, subunit 7a (Arabidopsis thaliana), full insert sequence. |
| AK029949 | 4933409K07Rik | 1.09 | hypothetical protein, full insert sequence. |
| AK032947 | Meis1 | 1.09 | myeloid ecotropic viral integration site 1, full insert sequence. |
| AK037090 | 2700078K21Rik | 1.09 | female vagina cDNA, RIKEN full-length enriched library |
| AK038979 | Ints9 | 1.09 | hypothalamus cDNA, RIKEN full-length enriched library, clone:A230081B12 product:similar to CDNA FLJ10871 FIS, CLONE NT2RP4001696, WEAKLY SIMILAR TO CLEAVAGE AND POLYADENYLATION SPECIFICITY FACTOR, 100 KDA SUBUNIT [Homo sapiens], full insert sequence. |
| AK042718 | 9630028H03Rik | 1.09 | unclassifiable, full insert sequence. |
| AK042884 | 2510048L02Rik | 1.09 | hypothetical ATP/GTP-binding site motif A (P-loop) containing protein, full insert sequence. |
| AK044453 | Ppp1r16b | 1.09 | PROTEIN PHOSPHATASE 1 REGULATORY SUBUNIT 16B homolog [Mus musculus], full insert sequence. |
| AK046465 | Dhrs13 | 1.09 | Hypothetical short-chain dehydrogenase/reductase homolog [Mus musculus], full insert sequence. |
| AK049843 | Spock3 | 1.09 | TESTICAN-3 PROTEIN PRECURSOR (2900045C01RIK PROTEIN) homolog [Mus musculus], full insert sequence. |
| AK081641 | Plaa | 1.09 | phospholipase A2, activating protein, full insert sequence. |
| AK084071 | Gm10021 | 1.09 | hypothetical protein, full insert sequence. |
| AK085944 | D430020J02Rik | 1.09 | hypothetical protein, full insert sequence. |
| AK087445 | Nat10 | 1.09 | hypothetical Putative ATPase DUF699 containing protein, full insert sequence. |
| AK089791 | Cdh17 | 1.09 | cadherin 17, full insert sequence. |
| AK129425 | Ddhd1 | 1.09 | mKIAA1705 protein. |
| AK132234 | Sgk1 | 1.09 | serum/glucocorticoid regulated kinase, full insert sequence. |
| AK134268 | Tmem87a | 1.09 | hypothetical Lung seven transmembrane receptor containing protein, full insert sequence. |
| AK135414 | Nfatc2 | 1.09 | nuclear factor of activated T-cells, cytoplasmic, calcineurin-dependent 2, full insert sequence. |
| AK139480 | Gnrh1 | 1.09 | Progonadoliberin I precursor [Contains: Gonadoliberin I (LH-RH I) (Luteinizing hormone-releasing hormone I) (Gonadotropin-releasing hormone I) (GnRH I) (Luliberin I); Prolactin release-inhibiting factor I], full insert sequence. |
| AK140485 | Epb4.1l4a | 1.09 | erythrocyte protein band 4.1-like 4a, full insert sequence. |
| AK142504 | Trpm3 | 1.09 | transient receptor potential cation channel, subfamily M, member 3, full insert sequence. |
| AK144632 | N/A | 1.09 | unclassifiable, full insert sequence. |
| AK146950 | H2-DMa | 1.09 | histocompatibility 2, class II, locus DMa, full insert sequence. |
| AK147659 | Bcl9 | 1.09 | B-cell CLL/lymphoma 9, full insert sequence. |
| AK148481 | Hadh | 1.09 | L-3-hydroxyacyl-Coenzyme A dehydrogenase, short chain, full insert sequence. |
| AK154337 | Rrp8 | 1.09 | Hypothetical protein KIAA0409 (Cerebral protein-1) (Fragment) [Homo sapiens], full insert sequence. |
| AK160604 | Ddx3y | 1.09 | DEAD (Asp-Glu-Ala-Asp) box polypeptide 3, Y-linked, full insert sequence. |
| AK162780 | Sltm | 1.09 | Hypothetical RNA-binding domain (Fragment) homolog [Mus musculus], full insert sequence. |
| AY534252 | Xkr6 | 1.09 | XK-related protein 6 mRNA, partial cds. |
| BC005452 | Bgn | 1.09 | biglycan, mRNA (cDNA clone MGC:6237 IMAGE:3594469), complete cds. |
| BC005556 | Sdcbp2 | 1.09 | syndecan binding protein (syntenin) 2, mRNA (cDNA clone MGC:11704 IMAGE:3964815), complete cds. |
| BC006611 | Angptl4 | 1.09 | angiopoietin-like 4, mRNA (cDNA clone MGC:11598 IMAGE:3966729), complete cds. |
| BC007146 | Kdelr2 | 1.09 | KDEL (Lys-Asp-Glu-Leu) endoplasmic reticulum protein retention receptor 2, mRNA (cDNA clone MGC:6366 IMAGE:3496891), complete cds. |
| BC007466 | Dnmt3a | 1.09 | DNA methyltransferase 3A, mRNA (cDNA clone MGC:5662 IMAGE:3492853), complete cds. |
| BC011230 | Nt5dc2 | 1.09 | 5'-nucleotidase domain containing 2, mRNA (cDNA clone MGC:19108 IMAGE:4207917), complete cds. |
| BC011289 | Dynll2 | 1.09 | dynein light chain LC8-type 2, mRNA (cDNA clone MGC:18733 IMAGE:3981362), complete cds. |
| BC013525 | Pipox | 1.09 | pipecolic acid oxidase, mRNA (cDNA clone MGC:19202 IMAGE:4237443), complete cds. |
| BC014693 | Tlr2 | 1.09 | toll-like receptor 2, mRNA (cDNA clone MGC:11394 IMAGE:3966249), complete cds. |
| BC014815 | Cbx8 | 1.09 | chromobox homolog 8 (Drosophila Pc class), mRNA (cDNA clone MGC:25653 IMAGE:4456898), complete cds. |
| BC015276 | Itih3 | 1.09 | inter-alpha trypsin inhibitor, heavy chain 3, mRNA (cDNA clone MGC:18839 IMAGE:4212290), complete cds. |
| BC019496 | Agt | 1.09 | angiotensinogen (serpin peptidase inhibitor, clade A, member 8), mRNA (cDNA clone MGC:28582 IMAGE:4211501), complete cds. |
| BC023719 | BC023719 | 1.09 | BC023719, mRNA (cDNA clone IMAGE:5347090), with apparent retained intron. |
| BC027554 | Cmtm8 | 1.09 | CKLF-like MARVEL transmembrane domain containing 8, mRNA (cDNA clone MGC:41386 IMAGE:1382818), complete cds. |
| BC027773 | Adamts4 | 1.09 | disintegrin-like and metallopeptidase (reprolysin type) with thrombospondin type 1 motif, 4, mRNA (cDNA clone MGC:38401 IMAGE:5345809), complete cds. |
| BC031891 | Serpina4-ps1 | 1.09 | serine (or cysteine) peptidase inhibitor, clade A, member 4, pseudogene 1, mRNA (cDNA clone MGC:29921 IMAGE:5123840), complete cds. |
| BC037464 | Cytsa | 1.09 | SPECC1-like, mRNA (cDNA clone MGC:47273 IMAGE:4194868), complete cds. |
| BC040367 | Lnx1 | 1.09 | ligand of numb-protein X 1, mRNA (cDNA clone IMAGE:4511688), complete cds. |
| BC046332 | Nap1l5 | 1.09 | nucleosome assembly protein 1-like 5, mRNA (cDNA clone MGC:54525 IMAGE:6491916), complete cds. |
| BC047068 | 2810432D09Rik | 1.09 | RIKEN cDNA 2810432D09 gene, mRNA (cDNA clone MGC:32232 IMAGE:5009034), complete cds. |
| BC048943 | BC048943 | 1.09 | cDNA clone IMAGE:6465364. |
| BC049101 | Lca5 | 1.09 | Leber congenital amaurosis 5 (human), mRNA (cDNA clone MGC:61394 IMAGE:5702365), complete cds. |
| BC049584 | Rsph1 | 1.09 | radial spoke head 1 homolog (Chlamydomonas), mRNA (cDNA clone MGC:58207 IMAGE:6744132), complete cds. |
| BC051046 | Ddx3y | 1.09 | DEAD (Asp-Glu-Ala-Asp) box polypeptide 3, Y-linked, mRNA (cDNA clone IMAGE:4502135), containing frame-shift errors. |
| BC051178 | Tmem117 | 1.09 | transmembrane protein 117, mRNA (cDNA clone MGC:56930 IMAGE:6314268), complete cds. |
| BC053061 | Uty | 1.09 | cDNA clone IMAGE:6409888, containing frame-shift errors. |
| BC055299 | A430033K04Rik | 1.09 | RIKEN cDNA A430033K04 gene, mRNA (cDNA clone MGC:62828 IMAGE:6492709), complete cds. |
| BC055930 | Alad | 1.09 | aminolevulinate, delta-, dehydratase, mRNA (cDNA clone MGC:68347 IMAGE:4207420), complete cds. |
| BC057196 | Agtrap | 1.09 | angiotensin II, type I receptor-associated protein, mRNA (cDNA clone MGC:68197 IMAGE:5249164), complete cds. |
| BC058659 | Mll2 | 1.09 | myeloid/lymphoid or mixed-lineage leukemia 2, mRNA (cDNA clone IMAGE:6408644), partial cds. |
| BC062910 | Ddx17 | 1.09 | DEAD (Asp-Glu-Ala-Asp) box polypeptide 17, mRNA (cDNA clone MGC:79147 IMAGE:6816663), complete cds. |
| BC067004 | Tmem130 | 1.09 | transmembrane protein 130, mRNA (cDNA clone MGC:90040 IMAGE:6416222), complete cds. |
| BC070401 | Sgk1 | 1.09 | serum/glucocorticoid regulated kinase, mRNA (cDNA clone MGC:99446 IMAGE:5695396), complete cds. |
| BC075622 | Cd24a | 1.09 | CD24a antigen, mRNA (cDNA clone MGC:99849 IMAGE:6415159), complete cds. |
| BC087877 | E030010A14Rik | 1.09 | RIKEN cDNA E030010A14 gene, mRNA (cDNA clone MGC:107144 IMAGE:30302311), complete cds. |
| BC096540 | Wdr92 | 1.09 | WD repeat domain 92, mRNA (cDNA clone MGC:106509 IMAGE:6403681), complete cds. |
| BC099486 | LOC100043371 | 1.09 | RIKEN cDNA 6030426L16 gene, mRNA (cDNA clone MGC:117729 IMAGE:30937101), complete cds. |
| BC099694 | 4831426I19Rik | 1.09 | RIKEN cDNA 4831426I19 gene, mRNA (cDNA clone MGC:106312 IMAGE:4507746), complete cds. |
| BC100393 | Klhl13 | 1.09 | kelch-like 13 (Drosophila), mRNA (cDNA clone MGC:118045 IMAGE:30737426), complete cds. |
| BC115435 | Slc27a6 | 1.09 | solute carrier family 27 (fatty acid transporter), member 6, mRNA (cDNA clone MGC:143900 IMAGE:40094587), complete cds. |
| BC116365 | Ly6g6c | 1.09 | lymphocyte antigen 6 complex, locus G6C, mRNA (cDNA clone MGC:144235 IMAGE:40100048), complete cds. |
| BC118022 | Sh3pxd2a | 1.09 | SH3 and PX domains 2A, mRNA (cDNA clone MGC:144076 IMAGE:40097448), complete cds. |
| BC119391 | Tmem212 | 1.09 | RIKEN cDNA E030011K20 gene, mRNA (cDNA clone MGC:155707 IMAGE:8734140), complete cds. |
| BC127616 | Tmem130 | 1.09 | transmembrane protein 130, mRNA (cDNA clone MGC:157450 IMAGE:40093666), complete cds. |
| BC132407 | Lypd2 | 1.09 | Ly6/Plaur domain containing 2, mRNA (cDNA clone MGC:164038 IMAGE:40130684), complete cds. |
| BC140224 | Tnfrsf18 | 1.09 | tumor necrosis factor receptor superfamily, member 18 (Tnfrsf18) mRNA, encodes complete protein. |
| BC140418 | Olfr126 | 1.09 | olfactory receptor 126 (Olfr126) mRNA, encodes complete protein. |
| BC146299 | Usp29 | 1.09 | ubiquitin specific peptidase 29 (Usp29) mRNA, encodes complete protein. |
| BC156486 | C4a | 1.09 | complement component 4A (Rodgers blood group) (C4a) mRNA, encodes complete protein. |
| CT010181 | ENSMUSG00000068790 | 1.09 | full open reading frame cDNA clone RZPDo836C0450D for gene 2610042L04Rik, RIKEN cDNA 2610042L04 gene; complete cds, incl. stopcodon. |
| DQ397205 | Tssk4 | 1.09 | alt '1700020B19Rik#4933424F08Rik' |
| EF651802 | Gm10014 | 1.09 | alpha5-takusan mRNA, complete cds. |
| M26448 | N/A | 1.09 | TCR delta chain mRNA, signal peptide and VDJC segments. |
| NM_001103366 | Vmn2r87 | 1.09 | vomeronasal 2, receptor 87 (Vmn2r87), mRNA. |
| NM_010057 | Dlx6 | 1.09 | distal-less homeobox 6 (Dlx6), mRNA. |
| XM_001004898 | Gm14217 | 1.09 | similar to ribosomal protein L5 (LOC668936), mRNA. |
| XM_001471507 | LOC100038847 | 1.09 | hypothetical protein LOC100038847 (LOC100038847), mRNA. |
| XM_001473318 | 1700066C05Rik | 1.09 | RIKEN cDNA 1700066C05 gene (1700066C05Rik), mRNA. |
| AB014889 | Nr4a2 | 1.09 | for Nurr2, complete cds. |
| AF093258 | Homer1 | 1.09 | homer-1b mRNA, complete cds. |
| AK003781 | Klraq1 | 1.09 | hypothetical protein, full insert sequence. |
| AK004638 | Pde3a | 1.09 | phosphodiesterase 3A, cGMP inhibited, full insert sequence. |
| AK007011 | Kcnj9 | 1.09 | unclassifiable, full insert sequence. |
| AK014061 | Map3k6 | 1.09 | mitogen-activated protein kinase kinase kinase 6, full insert sequence. |
| AK014352 | Tekt5 | 1.09 | weakly similar to CDNA FLJ32828 FIS, CLONE TESTI2003117, WEAKLY SIMILAR TO TEKTIN A1 [Homo sapiens], full insert sequence. |
| AK016850 | Golga7b | 1.09 | HYPOTHETICAL PROTEIN, MNCB-1213 [Homo sapiens], full insert sequence. |
| AK018575 | Wfdc1 | 1.09 | PROSTATE STROMAL PROTEIN PS20, full insert sequence. |
| AK029123 | Trak1 | 1.09 | weakly similar to CDNA FLJ30201 FIS, CLONE BRACE2001477, WEAKLY SIMILAR TO HUNTINGTIN ASSOCIATED PROTEIN 1 [Homo sapiens], full insert sequence. |
| AK029503 | N/A | 1.09 | unclassifiable, full insert sequence. |
| AK029750 | Kcnab3 | 1.09 | potassium voltage-gated channel, shaker-related subfamily, beta member 3, full insert sequence. |
| AK030920 | 4933439C10Rik | 1.09 | unclassifiable, full insert sequence. |
| AK031018 | Bcl2l13 | 1.09 | mitochondria located 1 homolog (human), full insert sequence. |
| AK033662 | Lpin2 | 1.09 | lipin 2, full insert sequence. |
| AK034635 | Usp52 | 1.09 | RIKEN cDNA 1200014O24 gene, full insert sequence. |
| AK039783 | Pcsk2 | 1.09 | proprotein convertase subtilisin/kexin type 2, full insert sequence. |
| AK044089 | Gm12408 | 1.09 | unclassifiable, full insert sequence. |
| AK046042 | N/A | 1.09 | unclassifiable, full insert sequence. |
| AK052518 | D430041D05Rik | 1.09 | G2 PROTEIN (FRAGMENT) homolog [Homo sapiens], full insert sequence. |
| AK053351 | Cap1 | 1.09 | adenylyl cyclase-associated CAP protein homolog 1 (S. cerevisiae, S. pombe), full insert sequence. |
| AK053385 | Ints6 | 1.09 | CANDIDATE TUMOR SUPPRESSOR PROTEIN DICE1 homolog [Homo sapiens], full insert sequence. |
| AK076278 | Col12a1 | 1.09 | procollagen, type XII, alpha 1, full insert sequence. |
| AK088246 | Tm2d2 | 1.09 | hypothetical protein, full insert sequence. |
| AK132188 | Mybpc1 | 1.09 | Myosin-binding protein C, slow-type (Slow MyBP-C) (C-protein, skeletal muscle slow-isoform) homolog [Homo sapiens], full insert sequence. |
| AK133871 | Rpusd3 | 1.09 | Hypothetical pseudouridine synthase containing protein homolog [Mus musculus], full insert sequence. |
| AK134620 | Nvl | 1.09 | nuclear VCP-like, full insert sequence. |
| AK138505 | N/A | 1.09 | unclassifiable, full insert sequence. |
| AK139388 | Sorbs2 | 1.09 | ARG/ABL-interacting protein ARGBP2A homolog [Mus musculus], full insert sequence. |
| AK141945 | Ndst3 | 1.09 | N-deacetylase/N-sulfotransferase (heparan glucosaminyl) 3, full insert sequence. |
| AK143166 | Kcnab2 | 1.09 | potassium voltage-gated channel, shaker-related subfamily, beta member 2, full insert sequence. |
| AK143886 | Ghr | 1.09 | growth hormone receptor, full insert sequence. |
| AK146714 | BC046404 | 1.09 | Hypothetical gene supported by BC017595 (Fragment) [Homo sapiens], full insert sequence. |
| AK147417 | Rimbp2 | 1.09 | RIM binding protein 2 (RIM-BP2) homolog [Mus musculus], full insert sequence. |
| AK147633 | Cobl | 1.09 | cordon-bleu, full insert sequence. |
| AK148302 | Sgip1 | 1.09 | Hypothetical second domain of Mu2 adaptin subunit homolog [Mus musculus], full insert sequence. |
| AK154250 | Cd37 | 1.09 | CD37 antigen, full insert sequence. |
| AK156757 | Ankrd33b | 1.09 | Hypothetical ankyrin repeat region circular profile/yeast DNA-binding domain containing protein homolog [Mus musculus], full insert sequence. |
| AK158709 | Csnk1e | 1.09 | casein kinase 1, epsilon, full insert sequence. |
| AK159028 | Sv2b | 1.09 | synaptic vesicle glycoprotein 2 b, full insert sequence. |
| AK164635 | Mri1 | 1.09 | Putative translation initiation factor [Anopheles gambiae], full insert sequence. |
| AK167554 | Pisd | 1.09 | RIKEN full-length enriched library, clone:I530020L18 product:Phosphatidylserine decarboxylase proenzyme (EC 4.1.1.65) [Contains: Phosphatidylserine decarboxylase alpha chain; Phosphatidylserine decarboxylase beta chain] homolog [Mus musculus], full insert sequence. |
| AK220353 | Mical2 | 1.09 | mKIAA0750 protein. |
| AY823997 | Synj2 | 1.09 | alt 'AI481647#SJ2#mKIAA0348' |
| BC002016 | Cldn5 | 1.09 | claudin 5, mRNA (cDNA clone MGC:5820 IMAGE:3488769), complete cds. |
| BC003224 | Rcbtb2 | 1.09 | regulator of chromosome condensation (RCC1) and BTB (POZ) domain containing protein 2, mRNA (cDNA clone MGC:6577 IMAGE:3482729), complete cds. |
| BC003821 | Irf1 | 1.09 | interferon regulatory factor 1, mRNA (cDNA clone MGC:6190 IMAGE:3600525), complete cds. |
| BC004649 | Iigp1 | 1.09 | interferon inducible GTPase 1, mRNA (cDNA clone MGC:5713 IMAGE:3483102), complete cds. |
| BC005429 | Matn2 | 1.09 | matrilin 2, mRNA (cDNA clone MGC:5875 IMAGE:3492881), complete cds. |
| BC005769 | Spint1 | 1.09 | serine protease inhibitor, Kunitz type 1, mRNA (cDNA clone MGC:6844 IMAGE:2650110), complete cds. |
| BC006607 | Cd34 | 1.09 | CD34 antigen, mRNA (cDNA clone MGC:11720 IMAGE:3966337), complete cds. |
| BC006777 | Sft2d1 | 1.09 | SFT2 domain containing 1, mRNA (cDNA clone MGC:8112 IMAGE:3588866), complete cds. |
| BC009120 | N/A | 1.09 | cDNA clone IMAGE:3710932, **** WARNING: chimeric clone ****. |
| BC009660 | Prkcdbp | 1.09 | protein kinase C, delta binding protein, mRNA (cDNA clone MGC:6598 IMAGE:3486461), complete cds. |
| BC010807 | Tcea3 | 1.09 | transcription elongation factor A (SII), 3, mRNA (cDNA clone MGC:18902 IMAGE:4240584), complete cds. |
| BC011152 | Golm1 | 1.09 | golgi membrane protein 1, mRNA (cDNA clone MGC:19040 IMAGE:4188434), complete cds. |
| BC012723 | Igfbp6 | 1.09 | insulin-like growth factor binding protein 6, mRNA (cDNA clone MGC:14073 IMAGE:4224052), complete cds. |
| BC013477 | Adh1 | 1.09 | alcohol dehydrogenase 1 (class I), mRNA (cDNA clone MGC:18885 IMAGE:4238555), complete cds. |
| BC014293 | Tcf4 | 1.09 | transcription factor 4, mRNA (cDNA clone MGC:13998 IMAGE:4014231), complete cds. |
| BC015075 | Osbpl1a | 1.09 | oxysterol binding protein-like 1A, mRNA (cDNA clone IMAGE:4014130), partial cds. |
| BC016077 | Sertad1 | 1.09 | SERTA domain containing 1, mRNA (cDNA clone MGC:27573 IMAGE:4486318), complete cds. |
| BC016551 | Fcrls | 1.09 | macrophage scavenger receptor 2, mRNA (cDNA clone MGC:28009 IMAGE:3602803), complete cds. |
| BC022759 | Mphosph8 | 1.09 | RIKEN cDNA 4930548G07 gene, mRNA (cDNA clone IMAGE:4923815), partial cds. |
| BC023492 | Ppm1d | 1.09 | protein phosphatase 1D magnesium-dependent, delta isoform, mRNA (cDNA clone MGC:32306 IMAGE:5026376), complete cds. |
| BC025646 | Fam81a | 1.09 | RIKEN cDNA 6430514L14 gene, mRNA (cDNA clone MGC:38269 IMAGE:5324986), complete cds. |
| BC025895 | Camkk2 | 1.09 | calcium/calmodulin-dependent protein kinase kinase 2, beta, mRNA (cDNA clone IMAGE:5026840), with apparent retained intron. |
| BC027424 | Pvalb | 1.09 | parvalbumin, mRNA (cDNA clone MGC:35957 IMAGE:4925213), complete cds. |
| BC029546 | Fkbp10 | 1.09 | FK506 binding protein 10, mRNA (cDNA clone MGC:36024 IMAGE:5059670), complete cds. |
| BC031429 | Prss12 | 1.09 | protease, serine, 12 neurotrypsin (motopsin), mRNA (cDNA clone MGC:18371 IMAGE:3665834), complete cds. |
| BC031498 | N/A | 1.09 | cDNA clone MGC:27817 IMAGE:3482714, complete cds. |
| BC031847 | Nvl | 1.09 | nuclear VCP-like, mRNA (cDNA clone MGC:25549 IMAGE:3964470), complete cds. |
| BC033350 | 2610034M16Rik | 1.09 | RIKEN cDNA 2610034M16 gene, mRNA (cDNA clone MGC:38934 IMAGE:5362778), complete cds. |
| BC034414 | Col6a2 | 1.09 | collagen, type VI, alpha 2, mRNA (cDNA clone MGC:36205 IMAGE:3594974), complete cds. |
| BC034723 | 1700025G04Rik | 1.09 | RIKEN cDNA 1700025G04 gene, mRNA (cDNA clone MGC:28137 IMAGE:3981517), complete cds. |
| BC036129 | Vps18 | 1.09 | vacuolar protein sorting 18 (yeast), mRNA (cDNA clone MGC:36354 IMAGE:4972693), complete cds. |
| BC040808 | Uimc1 | 1.09 | ubiquitin interaction motif containing 1, mRNA (cDNA clone MGC:49564 IMAGE:4038778), complete cds. |
| BC043671 | Zfp871 | 1.09 | RIKEN cDNA 9030612M13 gene, mRNA (cDNA clone MGC:49226 IMAGE:5040597), complete cds. |
| BC049553 | Ccdc70 | 1.09 | coiled-coil domain containing 70, mRNA (cDNA clone MGC:58141 IMAGE:6773824), complete cds. |
| BC052149 | Upf1 | 1.09 | UPF1 regulator of nonsense transcripts homolog (yeast), mRNA (cDNA clone MGC:59259 IMAGE:6332288), complete cds. |
| BC052321 | Faah | 1.09 | fatty acid amide hydrolase, mRNA (cDNA clone MGC:58928 IMAGE:6515634), complete cds. |
| BC052487 | Podn | 1.09 | podocan, mRNA (cDNA clone IMAGE:6392159), containing frame-shift errors. |
| BC053409 | Igh-6 | 1.09 | immunoglobulin heavy chain 6 (heavy chain of IgM), mRNA (cDNA clone MGC:60843 IMAGE:30076516), complete cds. |
| BC053430 | Pdgfb | 1.09 | platelet derived growth factor, B polypeptide, mRNA (cDNA clone MGC:59331 IMAGE:6330609), complete cds. |
| BC055695 | Cldnd1 | 1.09 | claudin domain containing 1, mRNA (cDNA clone MGC:66639 IMAGE:6414170), complete cds. |
| BC055811 | Igsf21 | 1.09 | immunoglobin superfamily, member 21, mRNA (cDNA clone MGC:67646 IMAGE:6414125), complete cds. |
| BC058749 | Synj2 | 1.09 | synaptojanin 2, mRNA (cDNA clone MGC:67830 IMAGE:6390688), complete cds. |
| BC061489 | N/A | 1.09 | CD74 antigen (invariant polypeptide of major histocompatibility complex, class II antigen-associated), mRNA (cDNA clone MGC:70236 IMAGE:4922119), complete cds. |
| BC062650 | Ccdc148 | 1.09 | cDNA sequence BC062650, mRNA (cDNA clone MGC:69717 IMAGE:6417188), complete cds. |
| BC063756 | Serpinb3a | 1.09 | serine (or cysteine) peptidase inhibitor, clade B (ovalbumin), member 3A, mRNA (cDNA clone MGC:70040 IMAGE:30288465), complete cds. |
| BC065118 | Ncan | 1.09 | neurocan, mRNA (cDNA clone MGC:86112 IMAGE:6853253), complete cds. |
| BC066066 | Scube1 | 1.09 | signal peptide, CUB domain, EGF-like 1, mRNA (cDNA clone MGC:91355 IMAGE:6850999), complete cds. |
| BC070445 | Fbxo41 | 1.09 | F-box protein 41, mRNA (cDNA clone MGC:91304 IMAGE:30536738), complete cds. |
| BC079612 | Gm9954 | 1.09 | cDNA clone IMAGE:30361372. |
| BC085129 | Syn2 | 1.09 | synapsin II, mRNA (cDNA clone MGC:113746 IMAGE:5701861), complete cds. |
| BC088998 | Zfp128 | 1.09 | zinc finger protein 128, mRNA (cDNA clone MGC:113768 IMAGE:6417536), complete cds. |
| BC092534 | Zfp697 | 1.09 | zinc finger protein 697, mRNA (cDNA clone MGC:107223 IMAGE:30249621), complete cds. |
| BC094348 | Schip1 | 1.09 | schwannomin interacting protein 1, mRNA (cDNA clone MGC:106748 IMAGE:6414097), complete cds. |
| BC095931 | N/A | 1.09 | RIKEN cDNA D930048N14 gene, mRNA (cDNA clone MGC:106227 IMAGE:5341501), complete cds. |
| BC096761 | Pstpip1 | 1.09 | proline-serine-threonine phosphatase-interacting protein 1, mRNA (cDNA clone MGC:107375 IMAGE:30040955), complete cds. |
| BC099457 | Vsig2 | 1.09 | V-set and immunoglobulin domain containing 2, mRNA (cDNA clone MGC:117653 IMAGE:30789633), complete cds. |
| BC152743 | Nkx2-4 | 1.08 | NK2 transcription factor related, locus 4 (Drosophila) (Nkx2-4) mRNA, encodes complete protein. |
| NM_024828.3 | CAAP1 | 1.08 | caspase activity and apoptosis inhibitor 1 |
| XM_004936566.2 | LPPR4 | 1.08 | Lipid phosphate phosphatase-related protein type 4 |
| NG_011650.1 | RASA1 | 1.08 | RAS p21 protein activator (GTPase activating protein) 1 |
| NM_004965.6 | HMGN1 | 1.08 | high mobility group nucleosome binding domain 1 |
| NM_001320698.1 | ANXA4 | 1.08 | annexin A4 |
| NM_018509.3 | LRRC59 | 1.08 | leucine rich repeat containing 59 |
| NM_006467.2 | POLR3G | 1.08 | polymerase (RNA) III (DNA directed) polypeptide G (32kD) |
| NM_021913.4 | AXL | 1.08 | AXL receptor tyrosine kinase |
| NG_029847.1 | MIER1 | 1.08 | mesoderm induction early response 1, transcriptional regulator |
| BC156193 | Sox30 | 1.08 | SRY-box containing gene 30 (Sox30) mRNA, encodes complete protein. |
| NM_001109985 | Nos1ap | 1.08 | nitric oxide synthase 1 (neuronal) adaptor protein (Nos1ap), transcript variant 1, mRNA. |
| U69888 | Ipw | 1.08 | Ipw mRNA, partial sequence. |
| XM_001474246 | Gm2661 | 1.08 | hypothetical protein LOC100040205 (LOC100040205), mRNA. |
| AF498300 | Rian | 1.08 | CZECHII/Ei imprinted mMeg8 mRNA, partial sequence. |
| AJ223070 | Tcf7l2 | 1.08 | TCF-4 protein. |
| AK005522 | Dlk1 | 1.08 | delta-like 1 homolog (Drosophila), full insert sequence. |
| AK011748 | 2610042L04Rik | 1.08 | hypothetical protein, full insert sequence. |
| AK015181 | Spsb1 | 1.08 | SPRY DOMAIN-CONTAINING SOCS BOX PROTEIN SSB-1 homolog [Mus musculus], full insert sequence. |
| AK030726 | Galnt13 | 1.08 | 5730414I04 product:similar to POLYPEPTIDE N-ACETYLGALACTOSAMINYLTRANSFERASE (EC 2.4.1.41) (PROTEIN- UDP ACETYLGALACTOSAMINYLTRANSFERASE) (UDP-GALNAC:POLYPEPTIDE, N-ACETYLGALACTOSAMINYLTRANSFERASE) (GALNAC-T1) [Bos taurus], full insert sequence. |
| AK037486 | 4931408A02Rik | 1.08 | similar to PROTEIN C21ORF63 HOMOLOG PRECURSOR [Mus musculus], full insert sequence. |
| AK038997 | Prune2 | 1.08 | BCL2/ADENOVIRUS E1B 19-KDA PROTEIN-INTERACTING PROTEIN 2 [Homo sapiens], full insert sequence. |
| AK044603 | Cacna2d2 | 1.08 | calcium channel, voltage-dependent, alpha 2/delta subunit 2, full insert sequence. |
| AK044744 | Epb4.1l1 | 1.08 | erythrocyte protein band 4.1-like 1, full insert sequence. |
| AK079258 | Gabre | 1.08 | GABA-A RECEPTOR EPSILON-LIKE SUBUNIT homolog [Mus musculus], full insert sequence. |
| AK081086 | N/A | 1.08 | unclassifiable, full insert sequence. |
| AK086373 | Slc36a2 | 1.08 | weakly similar to LYSOSOMAL AMINO ACID TRANSPORTER 1 [Rattus norvegicus], full insert sequence. |
| AK090372 | Gk5 | 1.08 | baculoviral IAP repeat-containing 1c, pseudogene 2, full insert sequence. |
| AK142837 | Pcgf2 | 1.08 | ring finger protein 110, full insert sequence. |
| AK166404 | Gm10791 | 1.08 | hypothetical protein, full insert sequence. |
| AY640621 | Ppp1r1b | 1.08 | alt 'AU040756#DARPP-32#Darpp32' |
| BC011320 | Tmem70 | 1.08 | transmembrane protein 70, mRNA (cDNA clone MGC:18940 IMAGE:3979025), complete cds. |
| BC013715 | Oas1a | 1.08 | 2'-5' oligoadenylate synthetase 1A, mRNA (cDNA clone MGC:6224 IMAGE:3257027), complete cds. |
| BC028942 | Kcne1l | 1.08 | potassium voltage-gated channel, Isk-related family, member 1-like, mRNA (cDNA clone MGC:35864 IMAGE:5363239), complete cds. |
| BC038365 | Ngfr | 1.08 | nerve growth factor receptor (TNFR superfamily, member 16), mRNA (cDNA clone MGC:35588 IMAGE:5367638), complete cds. |
| BC048074 | Heatr3 | 1.08 | HEAT repeat containing 3, mRNA (cDNA clone IMAGE:5397217), complete cds. |
| BC049947 | Padi2 | 1.08 | peptidyl arginine deiminase, type II, mRNA (cDNA clone MGC:58948 IMAGE:3600978), complete cds. |
| BC050894 | Wdr6 | 1.08 | WD repeat domain 6, mRNA (cDNA clone MGC:63325 IMAGE:5708880), complete cds. |
| BC056927 | Vat1l | 1.08 | expressed sequence AI427515, mRNA (cDNA clone MGC:66522 IMAGE:5719211), complete cds. |
| BC059060 | 4933409K07Rik | 1.08 | RIKEN cDNA 4933409K07 gene, mRNA (cDNA clone MGC:69869 IMAGE:6822098), complete cds. |
| BC061020 | Usp50 | 1.08 | ubiquitin specific peptidase 50, mRNA (cDNA clone MGC:74126 IMAGE:6772471), complete cds. |
| BC063087 | Shh | 1.08 | sonic hedgehog, mRNA (cDNA clone MGC:69975 IMAGE:6516263), complete cds. |
| BC075669 | A730017C20Rik | 1.08 | RIKEN cDNA A730017C20 gene, mRNA (cDNA clone MGC:100035 IMAGE:30550997), complete cds. |
| BC075699 | Cirbp | 1.08 | cold inducible RNA binding protein, mRNA (cDNA clone MGC:99900 IMAGE:30656047), complete cds. |
| BC086692 | Rpgrip1 | 1.08 | retinitis pigmentosa GTPase regulator interacting protein 1, mRNA (cDNA clone MGC:113764 IMAGE:30682813), complete cds. |
| BC089029 | Nosip | 1.08 | nitric oxide synthase interacting protein, mRNA (cDNA clone MGC:115777 IMAGE:30624825), complete cds. |
| BC089510 | Krr1 | 1.08 | KRR1, small subunit (SSU) processome component, homolog (yeast), mRNA (cDNA clone MGC:107200 IMAGE:30246541), complete cds. |
| BC100428 | Pld5 | 1.08 | phospholipase D family, member 5, mRNA (cDNA clone MGC:118236 IMAGE:30247182), complete cds. |
| BC115473 | Lipt2 | 1.08 | RIKEN cDNA 2610209A20 gene, mRNA (cDNA clone MGC:144281 IMAGE:40100449), complete cds. |
| BC118951 | Nhlrc1 | 1.08 | NHL repeat containing 1, mRNA (cDNA clone MGC:143773 IMAGE:40093176), complete cds. |
| BC125533 | Mgat5 | 1.08 | mannoside acetylglucosaminyltransferase 5, mRNA (cDNA clone MGC:159236 IMAGE:40130048), complete cds. |
| BC132111 | Sv2c | 1.08 | synaptic vesicle glycoprotein 2c, mRNA (cDNA clone MGC:163742 IMAGE:40130388), complete cds. |
| BC145881 | Olfr750 | 1.08 | olfactory receptor 750, mRNA (cDNA clone MGC:175768 IMAGE:40131184), complete cds. |
| BC156806 | Bcl3 | 1.08 | B-cell leukemia/lymphoma 3 (Bcl3) mRNA, encodes complete protein. |
| M26925 | Ggta1 | 1.08 | galactosyltransferase mRNA, complete cds. |
| NM_001099319 | OTTMUSG00000009332 | 1.08 | similar to transmembrane protein 35 (LOC100039968), mRNA. |
| NM_177940 | Ece2 | 1.08 | endothelin converting enzyme 2 (Ece2), transcript variant 2, mRNA. |
| XM_001000061 | 1500017E21Rik | 1.08 | hypothetical LOC668215 (LOC668215), mRNA. |
| XM_001001069 | Gm9121 | 1.08 | predicted gene, EG668353 (EG668353), mRNA. |
| XM_001474596 | 2900016B01Rik | 1.08 | cDNA 2900016B01 gene (2900016B01Rik), mRNA. |
| AB016248 | Sc5d | 1.08 | sterol-C5-desaturase, complete cds. |
| AK005153 | Slc13a1 | 1.08 | solute carrier family 13 (sodium/sulphate symporters), member 1, full insert sequence. |
| AK007918 | N/A | 1.08 | immunoglobulin heavy chain 1 (serum IgG2a), full insert sequence. |
| AK014353 | Khdrbs3 | 1.08 | etoile, full insert sequence. |
| AK040197 | Mgea5 | 1.08 | unclassifiable, full insert sequence. |
| AK044219 | Fam107a | 1.08 | DRR1 PROTEIN (TU3A PROTEIN) homolog [Homo sapiens], full insert sequence. |
| AK046737 | 9130024F11Rik | 1.08 | hypothetical protein, full insert sequence. |
| AK052275 | Ptprk | 1.08 | protein tyrosine phosphatase, receptor type, K, full insert sequence. |
| AK086736 | LOC433082 | 1.08 | unclassifiable, full insert sequence. |
| AK133063 | Poln | 1.08 | DNA polymerase N, full insert sequence. |
| AK140104 | Sv2b | 1.08 | synaptic vesicle glycoprotein 2 b, full insert sequence. |
| AK141312 | Sept9 | 1.08 | septin 9, full insert sequence. |
| AK158758 | Otub2 | 1.08 | Hypothetical protein FLJ21916 (Ubiquitin-specific protease otubain 2) homolog [Homo sapiens], full insert sequence. |
| AK159067 | Kctd6 | 1.08 | potassium channel tetramerisation domain containing 6, full insert sequence. |
| AK161075 | Mical2 | 1.08 | Hypothetical calponin homology homolog [Mus musculus], full insert sequence. |
| AK165200 | Astn1 | 1.08 | astrotactin 1, full insert sequence. |
| BC006587 | Zfp46 | 1.08 | zinc finger protein 46, mRNA (cDNA clone MGC:6166 IMAGE:3587306), complete cds. |
| BC010719 | Nfyb | 1.08 | nuclear transcription factor-Y beta, mRNA (cDNA clone MGC:6315 IMAGE:2811674), complete cds. |
| BC024634 | C1ql3 | 1.08 | C1q-like 3, mRNA (cDNA clone MGC:25969 IMAGE:4240136), complete cds. |
| BC027329 | Cmpk2 | 1.08 | thymidylate kinase family LPS-inducible member, mRNA (cDNA clone MGC:28140 IMAGE:3982036), complete cds. |
| BC043050 | Tcf4 | 1.08 | transcription factor 4, mRNA (cDNA clone MGC:57950 IMAGE:5708370), complete cds. |
| BC044927 | Gstm3 | 1.08 | glutathione S-transferase, mu 3, mRNA (cDNA clone MGC:49522 IMAGE:5051815), complete cds. |
| BC046307 | Grasp | 1.08 | GRP1 (general receptor for phosphoinositides 1)-associated scaffold protein, mRNA (cDNA clone MGC:54562 IMAGE:6306542), complete cds. |
| BC048453 | 1600012F09Rik | 1.08 | RIKEN cDNA 1600012F09 gene, mRNA (cDNA clone MGC:58173 IMAGE:6595060), complete cds. |
| BC051232 | Akap13 | 1.08 | A kinase (PRKA) anchor protein 13, mRNA (cDNA clone IMAGE:5255233). |
| BC052913 | Stk25 | 1.08 | serine/threonine kinase 25 (yeast), mRNA (cDNA clone MGC:60477 IMAGE:30057101), complete cds. |
| BC054474 | Elk1 | 1.08 | ELK1, member of ETS oncogene family, mRNA (cDNA clone MGC:60658 IMAGE:30045506), complete cds. |
| BC054529 | Zfp238 | 1.08 | zinc finger protein 238, mRNA (cDNA clone MGC:62253 IMAGE:5721393), complete cds. |
| BC057892 | Stx1a | 1.08 | cDNA clone IMAGE:4979880, containing frame-shift errors. |
| BC059938 | Pisd-ps3 | 1.08 | RIKEN cDNA 4933439C20 gene, mRNA (cDNA clone MGC:65558 IMAGE:6485174), complete cds. |
| BC061088 | Acyp1 | 1.08 | acylphosphatase 1, erythrocyte (common) type, mRNA (cDNA clone MGC:74203 IMAGE:6773293), complete cds. |
| BC069183 | Sp100 | 1.08 | nuclear antigen Sp100, mRNA (cDNA clone MGC:70218 IMAGE:4911025), complete cds. |
| BC076571 | Pgm2l1 | 1.08 | phosphoglucomutase 2-like 1, mRNA (cDNA clone MGC:100279 IMAGE:30356163), complete cds. |
| BC085289 | N/A | 1.08 | cDNA clone IMAGE:5353598, **** WARNING: chimeric clone ****. |
| BC089626 | 2310046A06Rik | 1.08 | RIKEN cDNA 2310046A06 gene, mRNA (cDNA clone MGC:107700 IMAGE:6757304), complete cds. |
| BC096478 | 9130230L23Rik | 1.08 | cDNA clone IMAGE:4985908. |
| BC127061 | Lrp8 | 1.07 | Low density lipoprotein receptor-related protein 8, apolipoprotein e receptor, mRNA (cDNA clone IMAGE:40109899), with apparent retained intron. |
| NR_023318.1 | ADPGK | 1.07 | ADP-dependent glucokinase |
| NM_004874.3 | BAG4 | 1.07 | BCL2-associated athanogene 4 |
| NM_001256279.1 | ZNF26 | 1.07 | zinc finger protein 26 |
| NM_006454.2 | MXD4 | 1.07 | MAX dimerization protein 4 |
| NG_029555.1 | ETS1 | 1.07 | v-ets avian erythroblastosis virus E26 oncogene homolog 1 |
| NM_001013699.2 | H3F3C | 1.07 | H3 histone, family 3C |
| AK012527 | Zfp64 | 1.07 | zinc finger protein 64, full insert sequence. |
| AK015529 | 4930469K13Rik | 1.07 | unclassifiable, full insert sequence. |
| AK033281 | Cep250 | 1.07 | SIMILAR TO CENTROSOMAL PROTEIN 2 homolog [Homo sapiens], full insert sequence. |
| AK077039 | Angel2 | 1.07 | testis cDNA, RIKEN full-length enriched library, clone:4932427P06 product:hypothetical Endonuclease/Exonuclease/phosphatase family containing protein, full insert sequence. |
| AK079328 | Kcnq2 | 1.07 | VOLTAGE-GATED POTASSIUM CHANNEL PROTEIN KQT-LIKE 2 homolog [Mus musculus], full insert sequence. |
| AK088331 | Lrch4 | 1.07 | SIMILAR TO LEUCINE-RICH NEURONAL PROTEIN homolog [Mus musculus], full insert sequence. |
| AK122566 | Kirrel3 | 1.07 | mKIAA1867 protein. |
| AK162302 | Mga | 1.07 | MAX gene associated, full insert sequence. |
| AK166015 | Arhgap6 | 1.07 | Rho GTPase activating protein 6, full insert sequence. |
| BC005643 | Gch1 | 1.07 | GTP cyclohydrolase 1, mRNA (cDNA clone MGC:12095 IMAGE:3709238), complete cds. |
| BC014742 | Mesdc2 | 1.07 | mesoderm development candidate 2, mRNA (cDNA clone MGC:25959 IMAGE:4239248), complete cds. |
| BC018323 | Dbp | 1.07 | D site albumin promoter binding protein, mRNA (cDNA clone MGC:18803 IMAGE:4195116), complete cds. |
| BC038310 | Hddc3 | 1.07 | HD domain containing 3, mRNA (cDNA clone MGC:47986 IMAGE:5119020), complete cds. |
| BC042713 | Dnajb9 | 1.07 | DnaJ (Hsp40) homolog, subfamily B, member 9, mRNA (cDNA clone MGC:51352 IMAGE:4039558), complete cds. |
| BC056342 | Glra2 | 1.07 | glycine receptor, alpha 2 subunit, mRNA (cDNA clone MGC:73412 IMAGE:5708257), complete cds. |
| BC056486 | A730011L01Rik | 1.07 | RIKEN cDNA A730011L01 gene, mRNA (cDNA clone MGC:67465 IMAGE:5697870), complete cds. |
| BC060989 | Ddc | 1.07 | cDNA clone IMAGE:30310870, containing frame-shift errors. |
| BC061183 | Ssbp1 | 1.07 | single-stranded DNA binding protein 1, mRNA (cDNA clone MGC:74323 IMAGE:30295766), complete cds. |
| BC075624 | Grid2ip | 1.07 | glutamate receptor, ionotropic, delta 2 (Grid2) interacting protein 1, mRNA (cDNA clone MGC:92989 IMAGE:6841572), complete cds. |
| BC096586 | Prlr | 1.07 | prolactin receptor, mRNA (cDNA clone MGC:106204 IMAGE:5337438), complete cds. |
| EF408739 | Ace2 | 1.07 | alt '2010305L05Rik' |
| AF102817 | Lmo4 | 1.07 | nuclear LIM-only 4 protein (Lmo4) mRNA, complete cds. |
| AK003073 | 1110038B12Rik | 1.07 | unclassifiable, full insert sequence. |
| AK014098 | Otud3 | 1.07 | hypothetical protein, full insert sequence. |
| AK018970 | Dclk1 | 1.07 | SERINE/THREONINE-PROTEIN KINASE DCAMKL1 (EC 2.7.1.-) (DOUBLECORTIN- LIKE AND CAM KINASE-LIKE 1) homolog [Mus musculus], full insert sequence. |
| AK034029 | N/A | 1.07 | tripartite motif protein 9, full insert sequence. |
| AK034517 | Pisd-ps3 | 1.07 | DJ858B16.2 (PHOSPHATIDYLSERINE DECARBOXYLASE (PSSC, EC 4.1.1.65)) [Homo sapiens], full insert sequence. |
| AK046093 | Slc35f4 | 1.07 | BRAIN CDNA, CLONE MNCB-0335 [Mus musculus], full insert sequence. |
| AK046740 | Ttc7b | 1.07 | HYPOTHETICAL PROTEIN KIAA1140 (FRAGMENT) [Homo sapiens], full insert sequence. |
| BC004663 | Dsc2 | 1.07 | desmocollin 2, mRNA (cDNA clone IMAGE:3498302), partial cds. |
| BC012692 | Akr1e1 | 1.07 | aldo-keto reductase family 1, member E1, mRNA (cDNA clone MGC:13946 IMAGE:3986863), complete cds. |
| BC016100 | Slc7a4 | 1.07 | solute carrier family 7 (cationic amino acid transporter, y+ system), member 4, mRNA (cDNA clone MGC:27672 IMAGE:4911158), complete cds. |
| BC017127 | Atp6v1b1 | 1.07 | ATPase, H+ transporting, lysosomal V1 subunit B1, mRNA (cDNA clone MGC:27582 IMAGE:4489144), complete cds. |
| BC033606 | Plb1 | 1.07 | phospholipase B1, mRNA (cDNA clone IMAGE:5373588), complete cds. |
| BC045198 | N/A | 1.07 | cDNA sequence BC026585, mRNA (cDNA clone MGC:49259 IMAGE:5065108), complete cds. |
| BC046627 | Rapgef5 | 1.07 | Rap guanine nucleotide exchange factor (GEF) 5, mRNA (cDNA clone MGC:54925 IMAGE:6494175), complete cds. |
| BC050750 | Csl | 1.07 | citrate synthase like, mRNA (cDNA clone MGC:58125 IMAGE:6775049), complete cds. |
| BC057460 | Rasa4 | 1.07 | RAS p21 protein activator 4, mRNA (cDNA clone MGC:65435 IMAGE:4527934), complete cds. |
| BC088983 | BC088983 | 1.07 | cDNA sequence BC088983, mRNA (cDNA clone MGC:113766 IMAGE:5700628), complete cds. |
| BC094417 | Tbc1d24 | 1.07 | TBC1 domain family, member 24, mRNA (cDNA clone MGC:117111 IMAGE:30943242), complete cds. |
| AK034587 | Dysf | 1.06 | Dysferlin, full insert sequence. |
| NM_001101387.1 | PIRT | 1.06 | phosphoinositide-interacting regulator of transient receptor potential channels |
| NM_152924.4 | ABHD2 | 1.06 | abhydrolase domain containing 2 |
| NM_001278469.1 | MON2 | 1.06 | MON2 homolog (S. cerevisiae) |
| NM_001286657.1 | TMEM68 | 1.06 | transmembrane protein 68 |
| NM_032784.4 | RSPO3 | 1.06 | R-spondin 3 |
| NM_001303247.1 | WDR61 | 1.06 | WD repeat domain 61 |
| NM_001083913.1 | WBP1L | 1.06 | WW domain binding protein 1-like |
| AK012409 | Pan3 | 1.06 | hypothetical protein, full insert sequence. |
| AK012981 | Oc90 | 1.06 | otoconin 90, full insert sequence. |
| AK015826 | Galntl6 | 1.06 | hypothetical Ricin B-like lectin structure containing protein, full insert sequence. |
| AK018169 | 3200002M19Rik | 1.06 | hypothetical Aspartic acid-rich region containing protein, full insert sequence. |
| AK031337 | Fads2 | 1.06 | fatty acid desaturase 2, full insert sequence. |
| AK044706 | Grik1 | 1.06 | weakly similar to GLUTAMATE RECEPTOR, IONOTROPIC KAINATE 1 PRECURSOR (GLUTAMATE RECEPTOR 5) (GLUR-5) (EXCITATORY AMINO ACID RECEPTOR 3) (EAA3) [Homo sapiens], full insert sequence. |
| AK046748 | Foxn3 | 1.06 | checkpoint suppressor 1, full insert sequence. |
| AK077656 | Ubxn10 | 1.06 | hypothetical UBX domain containing protein, full insert sequence. |
| AK081549 | BC024139 | 1.06 | hypothetical Growth-Arrest-Specific Protein 2 Domain/Bacterial regulatory protein, LysR family containing protein, full insert sequence. |
| AK090021 | Pla2g5 | 1.06 | CALCIUM-DEPENDENT PHOSPHOLIPASE A2 PRECURSOR (EC 3.1.1.4) (PHOSPHATIDYLCHOLINE 2-ACYLHYDROLASE) (PLA2-10) (GROUP V PHOSPHOLIPASE A2) homolog [Mus musculus], full insert sequence. |
| AK134715 | N/A | 1.06 | unclassifiable, full insert sequence. |
| AK147466 | Phf17 | 1.06 | PHD finger protein 17, full insert sequence. |
| AK172927 | Fcho1 | 1.06 | mKIAA0290 protein. |
| AY389983 | Ache | 1.06 | stress-associated acetylcholinesterase readthough variant AChE-R (Ache) mRNA, partial sequence. |
| BC005555 | Prlr | 1.06 | prolactin receptor, mRNA (cDNA clone MGC:6165 IMAGE:3586308), complete cds. |
| BC007166 | Epb4.1l4a | 1.06 | erythrocyte protein band 4.1-like 4a, mRNA (cDNA clone MGC:7458 IMAGE:3490061), complete cds. |
| BC012232 | S1pr5 | 1.06 | endothelial differentiation, sphingolipid G-protein-coupled receptor, 8, mRNA (cDNA clone MGC:18797 IMAGE:4194177), complete cds. |
| BC014795 | Tmem63a | 1.06 | transmembrane protein 63a, mRNA (cDNA clone MGC:11687 IMAGE:3961992), complete cds. |
| BC019122 | Hist2h2bb | 1.06 | histone cluster 2, H2bb, mRNA (cDNA clone MGC:29103 IMAGE:5003093), complete cds. |
| BC027570 | Atl1 | 1.06 | spastic paraplegia 3A homolog (human), mRNA (cDNA clone MGC:41702 IMAGE:1362466), complete cds. |
| BC028321 | 2210012G02Rik | 1.06 | RIKEN cDNA 2210012G02 gene, mRNA (cDNA clone MGC:35728 IMAGE:3992258), complete cds. |
| BC038083 | Ptgds | 1.06 | prostaglandin D2 synthase (brain), mRNA (cDNA clone MGC:47365 IMAGE:4481308), complete cds. |
| BC038167 | Ccdc153 | 1.06 | cDNA sequence BC038167, mRNA (cDNA clone IMAGE:3326572), partial cds. |
| BC042462 | Lpin1 | 1.06 | lipin 1, mRNA (cDNA clone MGC:36848 IMAGE:4211202), complete cds. |
| BC049569 | 1700054O13Rik | 1.06 | RIKEN cDNA 1700054O13 gene, mRNA (cDNA clone MGC:58166 IMAGE:6772969), complete cds. |
| BC049857 | Trps1 | 1.06 | trichorhinophalangeal syndrome I (human), mRNA (cDNA clone MGC:54437 IMAGE:4460789), complete cds. |
| BC051168 | Snx24 | 1.06 | sorting nexing 24, mRNA (cDNA clone MGC:56843 IMAGE:6306904), complete cds. |
| BC051472 | Ccl19 | 1.06 | chemokine (C-C motif) ligand 19, mRNA (cDNA clone MGC:62903 IMAGE:1349213), complete cds. |
| BC051997 | Avp | 1.06 | arginine vasopressin, mRNA (cDNA clone MGC:62313 IMAGE:5683720), complete cds. |
| BC055280 | Fabp7 | 1.06 | fatty acid binding protein 7, brain, mRNA (cDNA clone MGC:66923 IMAGE:5710017), complete cds. |
| BC055893 | Gbas | 1.06 | glioblastoma amplified sequence, mRNA (cDNA clone MGC:68220 IMAGE:4023066), complete cds. |
| BC057555 | Igsf11 | 1.06 | immunoglobulin superfamily, member 11, mRNA (cDNA clone MGC:66646 IMAGE:6416190), complete cds. |
| BC061254 | Fam183b | 1.06 | RIKEN cDNA 3100002J23 gene, mRNA (cDNA clone IMAGE:30250739). |
| BC061928 | Slc7a14 | 1.06 | solute carrier family 7 (cationic amino acid transporter, y+ system), member 14, mRNA (cDNA clone MGC:67673 IMAGE:5359765), complete cds. |
| BC062805 | Wdr65 | 1.06 | RIKEN cDNA 1110020C03 gene, mRNA (cDNA clone MGC:73552 IMAGE:1478343), complete cds. |
| BC071263 | N/A | 1.06 | cDNA clone IMAGE:6511278, partial cds. |
| BC072564 | N/A | 1.06 | cDNA clone IMAGE:6808940, **** WARNING: chimeric clone ****. |
| BC089351 | Ppcdc | 1.06 | phosphopantothenoylcysteine decarboxylase, mRNA (cDNA clone MGC:102190 IMAGE:30603115), complete cds. |
| BC099973 | Rhobtb1 | 1.06 | Rho-related BTB domain containing 1, mRNA (cDNA clone MGC:107538 IMAGE:6432524), complete cds. |
| BC115498 | Arl10 | 1.06 | ADP-ribosylation factor-like 10, mRNA (cDNA clone MGC:144317 IMAGE:40100921), complete cds. |
| BC118619 | Dnali1 | 1.06 | dynein, axonemal, light intermediate polypeptide 1, mRNA (cDNA clone MGC:148228 IMAGE:40107304), complete cds. |
| BC132529 | Slc10a4 | 1.06 | solute carrier family 10 (sodium/bile acid cotransporter family), member 4, mRNA (cDNA clone MGC:164160 IMAGE:40130806), complete cds. |
| BC140435 | Sik1 | 1.06 | SNF1-like kinase (Snf1lk) mRNA, encodes complete protein. |
| BC156234 | Cables2 | 1.06 | Cdk5 and Abl enzyme substrate 2 (Cables2) mRNA, encodes complete protein. |
| DQ115983 | Cmtm5 | 1.06 | alt '1500005P16Rik#2900052H21Rik#Cklfsf5' |
| XM_001002721 | Fam183b | 1.06 | RIKEN cDNA 3100002J23 gene, transcript variant 1 (3100002J23Rik), mRNA. |
| AK002701 | 0610030E20Rik | 1.06 | hypothetical protein, full insert sequence. |
| AK005746 | Ppp4r1l | 1.06 | unclassifiable, full insert sequence. |
| AK030987 | Lcp1 | 1.06 | plastin 2, L, full insert sequence. |
| AK031384 | Mfsd11 | 1.06 | ET PUTATIVE TRANSLATION PRODUCT (FRAGMENT), full insert sequence. |
| AK039999 | Cdk5r1 | 1.06 | cyclin-dependent kinase 5, regulatory subunit (p35), full insert sequence. |
| AK041338 | Nsg2 | 1.06 | neuron specific gene family member 2, full insert sequence. |
| AK042116 | Ralgapa1 | 1.06 | CDNA FLJ12926 FIS, CLONE NT2RP2004732, WEAKLY SIMILAR TO NEUROFILAMENT TRIPLET M PROTEIN homolog [Homo sapiens], full insert sequence. |
| AK046577 | Tlcd1 | 1.06 | hypothetical TRAM, LAG1 and CLN8 homology containing protein, full insert sequence. |
| AK080507 | N/A | 1.06 | HYPOTHETICAL 64.2 KDA PROTEIN [Mus musculus], full insert sequence. |
| AK135097 | Taf9b | 1.06 | TAF9-like RNA polymerase II, TATA box binding protein (TBP)-associated factor, 31 kDa (Fragment), full insert sequence. |
| AK138921 | N/A | 1.06 | unclassifiable, full insert sequence. |
| AK161191 | AI506816 | 1.06 | unclassifiable, full insert sequence. |
| BC006593 | Crot | 1.06 | carnitine O-octanoyltransferase, mRNA (cDNA clone MGC:7053 IMAGE:3156501), complete cds. |
| BC019131 | 1110067D22Rik | 1.06 | RIKEN cDNA 1110067D22 gene, mRNA (cDNA clone MGC:29165 IMAGE:5036749), complete cds. |
| BC046322 | B3galt2 | 1.06 | UDP-Gal:betaGlcNAc beta 1,3-galactosyltransferase, polypeptide 2, mRNA (cDNA clone MGC:54549 IMAGE:6392262), complete cds. |
| BC053093 | Zfp324 | 1.06 | zinc finger protein 324, mRNA (cDNA clone MGC:62482 IMAGE:6400158), complete cds. |
| BC079860 | Spnb3 | 1.06 | spectrin beta 3, mRNA (cDNA clone MGC:99874 IMAGE:6834745), complete cds. |
| BC082806 | Habp4 | 1.06 | hyaluronic acid binding protein 4, mRNA (cDNA clone MGC:90660 IMAGE:30361937), complete cds. |
| BC092227 | Akna | 1.06 | AT-hook transcription factor, mRNA (cDNA clone MGC:116630 IMAGE:30531788), complete cds. |
| AK034154 | Anks1b | 1.05 | E2A-PBX1-ASSOCIATED PROTEIN (FRAGMENT) homolog, full insert sequence. |
| NG_029944.1 | RNF138 | 1.05 | ring finger protein 138, E3 ubiquitin protein ligase |
| NM_080626.5 | BRI3BP | 1.05 | BRI3 binding protein |
| NM_001110504 | Capn1 | 1.05 | calpain 1 (Capn1), transcript variant 2, mRNA. |
| AK014173 | Tbkbp1 | 1.05 | unclassifiable, full insert sequence. |
| AK034254 | Fam161b | 1.05 | similar to CDNA FLJ31697 FIS, CLONE NT2RI2005851, WEAKLY SIMILAR TO PLECTIN [Homo sapiens], full insert sequence. |
| AK086508 | Gdpd2 | 1.05 | OSTEOBLAST DIFFERENTIATION PROMOTING FACTOR (9130017L10RIK PROTEIN), full insert sequence. |
| AK146009 | Cdc7 | 1.05 | cell division cycle 7 (S. cerevisiae), full insert sequence. |
| AK154366 | Mmel1 | 1.05 | mel transforming oncogene-like 1, full insert sequence. |
| AY351590 | Msh4 | 1.05 | strain C57BL/6J MutS homolog 4 variant theta 1 and MutS homolog 4 variant theta 2 (Msh4) bicistronic mRNA, complete cds. |
| AY626783 | Mtus1 | 1.05 | ATBP135 mRNA, complete cds. |
| BC007478 | Wbp5 | 1.05 | WW domain binding protein 5, mRNA (cDNA clone MGC:7478 IMAGE:3490650), complete cds. |
| BC014746 | Dph2 | 1.05 | cDNA clone IMAGE:4236184. |
| BC014821 | Renbp | 1.05 | renin binding protein, mRNA (cDNA clone MGC:25952 IMAGE:4238244), complete cds. |
| BC038340 | Hsd17b11 | 1.05 | hydroxysteroid (17-beta) dehydrogenase 11, mRNA (cDNA clone MGC:28723 IMAGE:4458725), complete cds. |
| BC038375 | Slc17a6 | 1.05 | solute carrier family 17 (sodium-dependent inorganic phosphate cotransporter), member 6, mRNA (cDNA clone MGC:36456 IMAGE:5357143), complete cds. |
| BC049818 | Tnrc18 | 1.05 | zinc finger protein 469, mRNA (cDNA clone MGC:59613 IMAGE:6511384), complete cds. |
| BC051667 | Meg3 | 1.05 | cDNA clone IMAGE:6431174, partial cds. |
| BC056616 | Ttc12 | 1.05 | tetratricopeptide repeat domain 12, mRNA (cDNA clone MGC:67683 IMAGE:4974294), complete cds. |
| BC057573 | Tle1 | 1.05 | transducin-like enhancer of split 1, homolog of Drosophila E(spl), mRNA (cDNA clone MGC:67016 IMAGE:6401650), complete cds. |
| BC057607 | Nkx2-1 | 1.05 | NK2 homeobox 1, mRNA (cDNA clone MGC:67328 IMAGE:6416507), complete cds. |
| BC057634 | Tbc1d16 | 1.05 | TBC1 domain family, member 16, mRNA (cDNA clone MGC:67717 IMAGE:2866041), complete cds. |
| BC072647 | 4933409K07Rik | 1.05 | RIKEN cDNA 4933409K07 gene, mRNA (cDNA clone MGC:99968 IMAGE:30532552), complete cds. |
| BC075728 | Tgs1 | 1.05 | trimethylguanosine synthase homolog (S. cerevisiae), mRNA (cDNA clone MGC:78321 IMAGE:6513964), complete cds. |
| BC104341 | 2310057N15Rik | 1.05 | RIKEN cDNA 2310057N15 gene, mRNA (cDNA clone MGC:129405 IMAGE:40049406), complete cds. |
| BC119256 | Olfr124 | 1.05 | olfactory receptor 124, mRNA (cDNA clone MGC:155572 IMAGE:8734005), complete cds. |
| BC119264 | Igsf1 | 1.05 | immunoglobulin superfamily, member 1, mRNA (cDNA clone MGC:155580 IMAGE:8734013), complete cds. |
| BC126883 | N/A | 1.05 | cDNA clone IMAGE:40048792. |
| XM_001003796 | OTTMUSG00000015077 | 1.05 | similar to olfactory receptor MOR248-10 (LOC668822), mRNA. |
| AK031399 | Camkk2 | 1.05 | CA+/CALMODULIN-DEPENDENT PROTEIN KINASE KINASE BETA (CAM-KINASE KINASE BETA) homolog [Rattus norvegicus], full insert sequence. |
| AK041026 | Hrnr | 1.05 | DJ14N1.2 (NOVEL S-100/ICABP TYPE CALCIUM BINDING DOMAIN PROTEIN, SIMILAR TO TRICHOHYALIN) (FRAGMENT) [Homo sapiens], full insert sequence. |
| AK042181 | Taok3 | 1.05 | STE20-like kinase homolog (Fragment), full insert sequence. |
| AK053540 | Miat | 1.05 | unclassifiable, full insert sequence. |
| AK085336 | Ints7 | 1.05 | hypothetical ARM repeat structure containing protein, full insert sequence. |
| AY251056 | Efemp1 | 1.05 | EFEMP1 (Efemp1) mRNA, complete cds. |
| BC037079 | 1300014I06Rik | 1.05 | RIKEN cDNA 1300014I06 gene, mRNA (cDNA clone MGC:46991 IMAGE:5051974), complete cds. |
| BC046304 | Dkk3 | 1.05 | dickkopf homolog 3 (Xenopus laevis), mRNA (cDNA clone MGC:54590 IMAGE:6493718), complete cds. |
| BC049639 | 1110008P14Rik | 1.05 | RIKEN cDNA 1110008P14 gene, mRNA (cDNA clone MGC:58399 IMAGE:6590746), complete cds. |
| BC049885 | Tceb3 | 1.05 | transcription elongation factor B (SIII), polypeptide 3, mRNA (cDNA clone MGC:54593 IMAGE:5387774), complete cds. |
| BC055373 | Cdsn | 1.05 | corneodesmosin, mRNA (cDNA clone MGC:60763 IMAGE:30058959), complete cds. |
| BC059785 | Blnk | 1.05 | B-cell linker, mRNA (cDNA clone MGC:67755 IMAGE:5339723), complete cds. |
| BC085130 | Lhfpl4 | 1.05 | lipoma HMGIC fusion partner-like protein 4, mRNA (cDNA clone MGC:65621 IMAGE:5704741), complete cds. |
| AK033352 | Alkbh8 | 1.04 | Similar to HYPOTHETICAL 75.2 KDA PROTEIN |
| AK034731 | Kctd15 | 1.04 | Hypothetical BTB/POZ domain containing protein, full insert sequence. |
| BC140312 | Ubash3a | 1.04 | Ubiquitin associated and SH3 domain containing, A (Ubash3a) mRNA, encodes complete protein. |
| NM_001099668.1 | HIGD1A | 1.04 | HIG1 hypoxia inducible domain family, member 1A |
| NG_009117.1 | TIMP3 | 1.04 | TIMP metallopeptidase inhibitor 3 |
| NM_004926.3 | ZFP36L1 | 1.04 | ZFP36 ring finger protein-like 1 |
| NM_003344.3 | UBE2H | 1.04 | ubiquitin-conjugating enzyme E2H |
| NM_018704.2 | CTTNBP2NL | 1.04 | CTTNBP2 N-terminal like |
| NR_130138.1 | HS3ST3B1 | 1.04 | heparan sulfate (glucosamine) 3-O-sulfotransferase 3B1 |
| NM_032377.3 | ELOF1 | 1.04 | elongation factor 1 homolog (S. cerevisiae) |
| NM_005868.5 | BET1 | 1.04 | Bet1 golgi vesicular membrane trafficking protein |
| NG_029114.1 | PFN2 | 1.04 | profilin 2 |
| NM_181712.4 | KANK4 | 1.04 | KN motif and ankyrin repeat domains 4 |
| NG_047042.1 | CDC42 | 1.04 | cell division cycle 42 |
| AB031037 | Eomes | 1.04 | Tbr2, complete cds. |
| AF399828 | Timd2 | 1.04 | DBA/2 TIM2 mRNA, complete cds. |
| AK042943 | Ppp2r3a | 1.04 | similar to SERINE/THREONINE PROTEIN PHOSPHATASE 2A, 72/130 KDA REGULATORY SUBUNIT B (PP2A, SUBUNIT B, B''-PR72/PR130) (PP2A, SUBUNIT B, B72/B130 ISOFORMS) (PP2A, SUBUNIT B, PR72/PR130 ISOFORMS) (PP2A, SUBUNIT B, R3 ISOFORM) [Homo sapiens], full insert sequence. |
| BC015268 | N/A | 1.04 | necdin, mRNA (cDNA clone MGC:18530 IMAGE:4217666), complete cds. |
| BC027737 | Ppp1r11 | 1.04 | protein phosphatase 1, regulatory (inhibitor) subunit 11, mRNA (cDNA clone MGC:29178 IMAGE:5003418), complete cds. |
| BC104370 | Clec4a4 | 1.04 | C-type lectin domain family 4, member a4, mRNA (cDNA clone MGC:129456 IMAGE:40049888), complete cds. |
| BC115691 | Rwdd2a | 1.04 | RWD domain containing 2A, mRNA (cDNA clone MGC:144625 IMAGE:40104313), complete cds. |
| BC115961 | N/A | 1.04 | cDNA clone IMAGE:40053282. |
| BC118541 | Wdr16 | 1.04 | WD repeat domain 16, mRNA (cDNA clone MGC:141350 IMAGE:40058756), complete cds. |
| BC119074 | Gabrq | 1.04 | gamma-aminobutyric acid (GABA-A) receptor, subunit theta, mRNA (cDNA clone MGC:155390 IMAGE:8733823), complete cds. |
| BC119182 | Ntn4 | 1.04 | netrin 4, mRNA (cDNA clone MGC:155498 IMAGE:8733931), complete cds. |
| BC119268 | Nt5e | 1.04 | 5' nucleotidase, ecto, mRNA (cDNA clone MGC:155584 IMAGE:8734017), complete cds. |
| BC126873 | Mrgpra1 | 1.04 | MAS-related GPR, member A1, mRNA (cDNA clone MGC:123921 IMAGE:40044758), complete cds. |
| BC126966 | N/A | 1.04 | cDNA clone IMAGE:40096645. |
| BC127222 | Olfr518 | 1.04 | olfactory receptor 518, mRNA (cDNA clone MGC:157426 IMAGE:40044607), complete cds. |
| BC132534 | Syce2 | 1.04 | synaptonemal complex central element protein 2, mRNA (cDNA clone MGC:164165 IMAGE:40130811), complete cds. |
| BC132600 | Il1f5 | 1.04 | interleukin 1 family, member 5 (delta), mRNA (cDNA clone MGC:164231 IMAGE:40130877), complete cds. |
| BC132612 | Krtap16-8 | 1.04 | keratin associated protein 16-8, mRNA (cDNA clone MGC:164243 IMAGE:40130889), complete cds. |
| BC141492 | Gm5409 | 1.04 | predicted gene, EG386551 (EG386551) mRNA, encodes complete protein. |
| BC141631 | Tas2r123 | 1.04 | taste receptor, type 2, member 123 (Tas2r123) mRNA, encodes complete protein. |
| BC156285 | Wasf2 | 1.04 | WAS protein family, member 2 (Wasf2) mRNA, encodes complete protein. |
| BC157095 | Blm | 1.04 | Bloom syndrome homolog (human) (Blm) mRNA, encodes complete protein. |
| EF651814 | Gm10340 | 1.04 | alpha17-takusan mRNA, complete cds. |
| EF651817 | ENSMUSG00000072735 | 1.04 | alpha20-takusan mRNA, complete cds. |
| NM_021304 | Abhd1 | 1.04 | abhydrolase domain containing 1 (Abhd1), mRNA. |
| NM_029288 | 1700001E04Rik | 1.04 | RIKEN cDNA 1700001E04 gene (1700001E04Rik), mRNA. |
| NM_130877 | Peg10 | 1.04 | paternally expressed 10 (Peg10), transcript variant 1, mRNA. |
| U35310 | N/A | 1.04 | mel-13b transcript. |
| XM_001006595 | Gm14354 | 1.04 | similar to 4930408F14Rik protein (LOC668961), mRNA. |
| XM_001472168 | Gm2023 | 1.04 | similar to EBI-1 ligand chemokine (LOC100039053), mRNA. |
| AB101295 | Ovol2 | 1.04 | MOVO mRNA for MOVO-B zinc finger protein, complete cds. |
| AB125594 | Etl4 | 1.04 | Skt-a mRNA for Sickle tail-a, complete cds. |
| AK006290 | Ppcs | 1.04 | hypothetical protein, full insert sequence. |
| AK014376 | Caskin1 | 1.04 | CASK-INTERACTING PROTEIN 1 homolog [Rattus norvegicus], full insert sequence. |
| AK018352 | Etl4 | 1.04 | unclassifiable, full insert sequence. |
| AK030323 | Bnc2 | 1.04 | CDNA FLJ20043 FIS, CLONE COL00430 homolog [Homo sapiens], full insert sequence. |
| AK031576 | Fam53b | 1.04 | HYPOTHETICAL PROTEIN KIAA0140 homolog [Homo sapiens], full insert sequence. |
| AK031750 | Mpped1 | 1.04 | hypothetical Metallo-dependent phosphatases structure containing protein, full insert sequence. |
| AK044685 | Rimbp2 | 1.04 | RIM BINDING PROTEIN 2 (FRAGMENT) homolog [Rattus norvegicus], full insert sequence. |
| AK080536 | Samd12 | 1.04 | unclassifiable, full insert sequence. |
| AK122319 | Pip5k1c | 1.04 | mKIAA0589 protein. |
| AK122550 | Syne1 | 1.04 | mKIAA1756 protein. |
| AK135347 | Nipbl | 1.04 | IDN3-B protein homolog [Homo sapiens], full insert sequence. |
| AK135491 | Rsu1 | 1.04 | unclassifiable, full insert sequence. |
| AK138967 | Wnt11 | 1.04 | wingless-related MMTV integration site 11, full insert sequence. |
| AK140817 | Sema3a | 1.04 | sema domain, immunoglobulin domain (Ig), short basic domain, secreted, (semaphorin) 3A, full insert sequence. |
| AK141439 | Klc2 | 1.04 | kinesin light chain 2, full insert sequence. |
| AK144210 | Gprin1 | 1.04 | G protein-regulated inducer of neurite outgrowth 1, full insert sequence. |
| AK146545 | Asap1 | 1.04 | development and differentiation enhancing, full insert sequence. |
| AK147645 | Ppfia3 | 1.04 | Liprin alpha 3 (Fragment) homolog [Rattus norvegicus], full insert sequence. |
| AK154457 | Tec | 1.04 | cytoplasmic tyrosine kinase, Dscr28C related (Drosophila), full insert sequence. |
| AK164214 | Gemin5 | 1.04 | gem (nuclear organelle) associated protein 5, full insert sequence. |
| AK169887 | Fcgr3 | 1.04 | Fc receptor, IgG, low affinity III, full insert sequence. |
| BC005431 | Anpep | 1.04 | alanyl (membrane) aminopeptidase, mRNA (cDNA clone MGC:5920 IMAGE:3593307), complete cds. |
| BC018216 | Slc16a8 | 1.04 | solute carrier family 16 (monocarboxylic acid transporters), member 8, mRNA (cDNA clone MGC:25469 IMAGE:4481698), complete cds. |
| BC019416 | Tmem40 | 1.04 | transmembrane protein 40, mRNA (cDNA clone MGC:30375 IMAGE:5151159), complete cds. |
| BC019760 | N/A | 1.04 | Ig kappa chain, mRNA (cDNA clone MGC:30228 IMAGE:4206515), complete cds. |
| BC021383 | Pip4k2c | 1.04 | phosphatidylinositol-5-phosphate 4-kinase, type II, gamma, mRNA (cDNA clone MGC:29371 IMAGE:5042933), complete cds. |
| BC021468 | Fhl2 | 1.04 | four and a half LIM domains 2, mRNA (cDNA clone MGC:29060 IMAGE:5066565), complete cds. |
| BC026554 | Chga | 1.04 | chromogranin A, mRNA (cDNA clone MGC:36117 IMAGE:4990941), complete cds. |
| BC031841 | Pamr1 | 1.04 | RIKEN cDNA E430002G05 gene, mRNA (cDNA clone MGC:30636 IMAGE:3675412), complete cds. |
| BC044883 | Ociad2 | 1.04 | OCIA domain containing 2, mRNA (cDNA clone MGC:51598 IMAGE:4971755), complete cds. |
| BC052184 | 6430573F11Rik | 1.04 | RIKEN cDNA 6430573F11 gene, mRNA (cDNA clone MGC:59528 IMAGE:6336362), complete cds. |
| BC053007 | Bhlhe22 | 1.04 | basic helix-loop-helix domain containing, class B5, mRNA (cDNA clone MGC:62191 IMAGE:5686844), complete cds. |
| BC056386 | Edil3 | 1.04 | EGF-like repeats and discoidin I-like domains 3, mRNA (cDNA clone MGC:73461 IMAGE:6854497), complete cds. |
| BC064737 | Mapk11 | 1.04 | mitogen-activated protein kinase 11, mRNA (cDNA clone MGC:76486 IMAGE:30112690), complete cds. |
| BC079630 | Dbc1 | 1.04 | deleted in bladder cancer 1 (human), mRNA (cDNA clone MGC:90661 IMAGE:5694980), complete cds. |
| BC080824 | Zfp874 | 1.04 | RIKEN cDNA C330011K17 gene, mRNA (cDNA clone MGC:91167 IMAGE:30458333), complete cds. |
| BC089587 | Slc26a10 | 1.04 | expressed sequence C78409, mRNA (cDNA clone MGC:107595 IMAGE:6757541), complete cds. |
| BC098187 | Hdac9 | 1.04 | histone deacetylase 9, mRNA (cDNA clone MGC:106408 IMAGE:5363624), complete cds. |
| BC099409 | Pgc | 1.04 | progastricsin (pepsinogen C), mRNA (cDNA clone MGC:117575 IMAGE:30790663), complete cds. |
| BC145775 | Acvr1b | 1.03 | Activin A receptor, type 1B, mRNA (cDNA clone MGC:175662 IMAGE:40131078), complete cds. |
| BC145949 | Vgll1 | 1.03 | Vestigial like 1 homolog (Drosophila), mRNA (cDNA clone MGC:175836 IMAGE:40131252), complete cds. |
| NC_003313.1 | COX | 1.03 | cox putative regulator |
| NG_008158.1 | PDCD10 | 1.03 | programmed cell death 10 |
| NM_031286.3 | SH3BGRL3 | 1.03 | SH3 domain binding glutamic acid-rich protein like 3 |
| NG_031908.1 | CHSY1 | 1.03 | chondroitin sulfate synthase 1 |
| NM_015878.5 | AZIN1 | 1.03 | antizyme inhibitor 1 |
| NG_047043.1 | CLTC | 1.03 | clathrin, heavy chain (Hc) |
| NM_152667.2 | NANP | 1.03 | N-acetylneuraminic acid phosphatase |
| NM_001839.4 | CNN3 | 1.03 | calponin 3, acidic |
| NG_042185.1 | KIF2A | 1.03 | kinesin heavy chain member 2A |
| NG_027725.1 | FBXO33 | 1.03 | F-box protein 33 |
| NM_005190.3 | CCNC | 1.03 | cyclin C |
| NG_028084.1 | SH3GL1 | 1.03 | SH3-domain GRB2-like 1 |
| NM_001001449 | AF067063 | 1.03 | AF067063 (AF067063), mRNA. |
| XM_001474560 | Gm2739 | 1.03 | hypothetical protein LOC100040370 (LOC100040370), mRNA. |
| AB185844 | Wdr67 | 1.03 | 4-B-3 mRNA for hypothetical protein 4-B-3, complete cds. |
| AF475070 | Ntng1 | 1.03 | domesticus laminet-1B (Lmnt1) mRNA, complete cds |
| AJ010604 | Sox5 | 1.03 | transcription factor L-Sox5. |
| AJ276961 | Clasp2 | 1.03 | alt '1500004F14Rik#8030404L10Rik#C77448#CLASP2beta#mKIAA0627' |
| AK029955 | N/A | 1.03 | retinitis pigmentosa GTPase regulator interacting protein 1, full insert sequence. |
| AK030632 | Zfp14 | 1.03 | zinc finger protein 14, full insert sequence. |
| AK045614 | Sox2ot | 1.03 | unclassifiable, full insert sequence. |
| AK049619 | ENSMUSG00000068790 | 1.03 | hypothetical protein, full insert sequence. |
| AK076000 | Skap2 | 1.03 | SRC-ASSOCIATED ADAPTOR PROTEIN homolog [Homo sapiens], full insert sequence. |
| AK078894 | Ppp2r5e | 1.03 | protein phosphatase 2, regulatory subunit B (B56), epsilon isoform, full insert sequence. |
| AK083632 | Derl2 | 1.03 | hypothetical protein, MGC: 11613, full insert sequence. |
| AK140446 | Gm2464 | 1.03 | unclassifiable, full insert sequence. |
| AK160563 | Rasgrp2 | 1.03 | RAS, guanyl releasing protein 2, full insert sequence. |
| BC005708 | 2610018G03Rik | 1.03 | RIKEN cDNA 2610018G03 gene, mRNA (cDNA clone MGC:6448 IMAGE:2598933), complete cds. |
| BC024263 | Ngb | 1.03 | neuroglobin, mRNA (cDNA clone MGC:35839 IMAGE:5133442), complete cds. |
| BC024605 | Cml5 | 1.03 | camello-like 5, mRNA (cDNA clone MGC:25942 IMAGE:4236653), complete cds. |
| BC028345 | Slc26a2 | 1.03 | solute carrier family 26 (sulfate transporter), member 2, mRNA (cDNA clone MGC:14061 IMAGE:4014785), complete cds. |
| BC040350 | Padi2 | 1.03 | peptidyl arginine deiminase, type II, mRNA (cDNA clone MGC:18700 IMAGE:4192742), complete cds. |
| BC042484 | Nr2f2 | 1.03 | nuclear receptor subfamily 2, group F, member 2, mRNA (cDNA clone MGC:36098 IMAGE:5368085), complete cds. |
| BC046624 | Mns1 | 1.03 | meiosis-specific nuclear structural protein 1, mRNA (cDNA clone MGC:54738 IMAGE:6314904), complete cds. |
| BC055874 | 544988 | 1.03 | hypothetical protein LOC544988, mRNA (cDNA clone MGC:68059 IMAGE:3491523), complete cds. |
| BC056431 | Cartpt | 1.03 | CART prepropeptide, mRNA (cDNA clone MGC:66635 IMAGE:6817832), complete cds. |
| BC061211 | AW551984 | 1.03 | expressed sequence AW551984, mRNA (cDNA clone MGC:74355 IMAGE:30250574), complete cds. |
| BC066803 | Fermt3 | 1.03 | cDNA sequence BC032204, mRNA (cDNA clone MGC:76489 IMAGE:30043017), complete cds. |
| BC067018 | Ebf3 | 1.03 | early B-cell factor 3, mRNA (cDNA clone MGC:79225 IMAGE:6833120), complete cds. |
| BC068299 | Ttc34 | 1.03 | RIKEN cDNA B230396O12 gene, mRNA (cDNA clone MGC:76433 IMAGE:30094917), complete cds. |
| BC088981 | Psme1 | 1.03 | proteasome (prosome, macropain) 28 subunit, alpha, mRNA (cDNA clone MGC:113815 IMAGE:5689048), complete cds. |
| BC088982 | Nrsn2 | 1.03 | neurensin 2, mRNA (cDNA clone MGC:113772 IMAGE:5700110), complete cds. |
| BC099519 | Enkur | 1.03 | RIKEN cDNA 4933434I06 gene, mRNA (cDNA clone MGC:117794 IMAGE:30848009), complete cds. |
| BC104271 | Olfr447 | 1.03 | olfactory receptor 447, mRNA (cDNA clone MGC:129177 IMAGE:40040950), complete cds. |
| BC116293 | Ttll8 | 1.03 | RIKEN cDNA 1700019P01 gene, mRNA (cDNA clone MGC:143913 IMAGE:40094952), complete cds. |
| BC116314 | Slc2a12 | 1.03 | solute carrier family 2 (facilitated glucose transporter), member 12, mRNA (cDNA clone MGC:143958 IMAGE:40095492), complete cds. |
| BC118523 | 4930579J09Rik | 1.03 | RIKEN cDNA 4930579J09 gene, mRNA (cDNA clone MGC:144717 IMAGE:40105249), complete cds. |
| BC119245 | Pld6 | 1.03 | RIKEN cDNA 4933433K01 gene, mRNA (cDNA clone MGC:155561 IMAGE:8733994), complete cds. |
| BC119324 | Galnt6 | 1.03 | UDP-N-acetyl-alpha-D-galactosamine:polypeptide N-acetylgalactosaminyltransferase 6, mRNA (cDNA clone MGC:155640 IMAGE:8734073), complete cds. |
| BC127237 | Fbxw13 | 1.03 | F-box and WD-40 domain protein 13, mRNA (cDNA clone MGC:157442 IMAGE:40056507), complete cds. |
| BC145645 | 1100001E04Rik | 1.03 | RIKEN cDNA 1100001E04 gene, mRNA (cDNA clone MGC:175532 IMAGE:40130948), complete cds. |
| BC156111 | Agt | 1.03 | angiotensinogen (serpin peptidase inhibitor, clade A, member 8) (Agt) mRNA, encodes complete protein. |
| BC156697 | Arhgef6 | 1.03 | Rac/Cdc42 guanine nucleotide exchange factor (GEF) 6 (Arhgef6) mRNA, encodes complete protein. |
| BC156773 | Hba-a2 | 1.03 | hemoglobin alpha, adult chain 2 (Hba-a2) mRNA, encodes complete protein. |
| BC156821 | Pcsk4 | 1.03 | proprotein convertase subtilisin/kexin type 4 (Pcsk4) mRNA, encodes complete protein. |
| BC156948 | Rdh19 | 1.03 | retinol dehydrogenase similar (Rdhs) mRNA, encodes complete protein. |
| DQ459435 | Gm4924 | 1.03 | inhibitor of four 1 (Mif1) mRNA, complete cds. |
| EF651803 | 100039441 | 1.03 | alpha6-takusan mRNA, complete cds. |
| U33012 | Aqp4 | 1.03 | mercurial-insensitive water channel mRNA, complete cds. |
| XM_001471958 | Mup9 | 1.03 | smilar to major urinary protein 1 (LOC100038948), mRNA. |
| XM_001472257 | Gm11496 | 1.03 | hypothetical protein LOC100039051 (LOC100039051), mRNA. |
| XM_001472300 | Gm2058 | 1.03 | similar to Ubiquitin-conjugating enzyme UbcH2, transcript variant 2 (LOC100039133), mRNA. |
| AB099695 | Shank2 | 1.03 | Shank2 mRNA, partial cds. |
| AK006119 | Prss23 | 1.03 | hypothetical protein, full insert sequence. |
| AK007941 | 1810063B07Rik | 1.03 | hypothetical protein, full insert sequence. |
| AK007947 | 1810063I02Rik | 1.03 | unclassifiable, full insert sequence. |
| AK018049 | Nvl | 1.03 | NUCLEAR VCP-LIKE PROTEIN (FRAGMENT) homolog [Homo sapiens], full insert sequence. |
| AK018801 | Ctla2a | 1.03 | cytotoxic T lymphocyte-associated protein 2 alpha, full insert sequence. |
| AK034269 | Il1rap | 1.03 | interleukin 1 receptor accessory protein, full insert sequence. |
| AK040796 | Arhgap15 | 1.03 | UNCHARACTERIZED BONE MARROW PROTEIN BM046 [Homo sapiens], full insert sequence. |
| AK044512 | Mprip | 1.03 | Rho interacting protein 3, full insert sequence. |
| AK053370 | Mical2 | 1.03 | hypothetical Calponin homology (CH) domain/LIM domain/Calponin homology domain profile/LIM domain profile containing protein, full insert sequence. |
| AK076418 | Ogt | 1.03 | UDP-N-ACETYLGLUCOSAMINE: POLYPEPTIDE-N-ACETYLGLUCOSAMINYL TRANSFERASE homolog [Homo sapiens], full insert sequence. |
| AK085201 | D530008I23 | 1.03 | unclassifiable, full insert sequence. |
| AK087410 | Gpr68 | 1.03 | PROBABLE G PROTEIN-COUPLED RECEPTOR GPR68 (OVARIAN CANCER G PROTEIN- COUPLED RECEPTOR 1) (OGR-1) homolog [Homo sapiens], full insert sequence. |
| AK087732 | Hspa12a | 1.03 | Mus musculus 2 days pregnant adult female ovary cDNA, RIKEN full-length enriched library, clone:E330013L02 product: |
| AK122227 | Slc23a2 | 1.03 | mKIAA0238 protein. |
| AK122265 | Erc2 | 1.03 | mKIAA0378 protein. |
| AK122424 | Rap1gap2 | 1.03 | mKIAA1039 protein. |
| AK134600 | Ak3l1 | 1.03 | adenylate kinase 4, full insert sequence. |
| AK139698 | Gm8016 | 1.03 | unclassifiable, full insert sequence. |
| AK140224 | Dnajc21 | 1.03 | hypothetical protein, full insert sequence. |
| AK148196 | Upf1 | 1.03 | regulator of nonsense transcripts 1, full insert sequence. |
| AK161467 | Synj2 | 1.03 | synaptojanin 2, full insert sequence. |
| AK169527 | Wdr73 | 1.03 | 2410008B13Rik protein (Hypothetical Trp-Asp repeat), full insert sequence. |
| AK220283 | Kif3c | 1.03 | mKIAA4058 protein. |
| AY211262 | Slc36a1 | 1.03 | amino acid transport protein (Slc36a1) mRNA, complete cds. |
| AY821853 | Myo7a | 1.03 | alt 'Myo7#USH1B#nmf371#sh-1#sh1' |
| AY989856 | H2-Bl | 1.03 | strain C3H/HeJ truncated MHC class I antigen splice variant Bl.2 (H2-Bl) mRNA, H2-Bl-k allele, complete cds. |
| BC003774 | Nov | 1.03 | nephroblastoma overexpressed gene, mRNA (cDNA clone MGC:5979 IMAGE:3494896), complete cds. |
| BC003819 | H2-T22 | 1.03 | histocompatibility 2, T region locus 22, mRNA (cDNA clone MGC:6180 IMAGE:3484613), complete cds. |
| BC009104 | Tceb1 | 1.03 | transcription elongation factor B (SIII), polypeptide 1, mRNA (cDNA clone MGC:7222 IMAGE:3483215), complete cds. |
| BC009659 | Adk | 1.03 | adenosine kinase, mRNA (cDNA clone MGC:6593 IMAGE:3485959), complete cds. |
| BC011424 | Prkar1b | 1.03 | protein kinase, cAMP dependent regulatory, type I beta, mRNA (cDNA clone MGC:18526 IMAGE:3674751), complete cds. |
| BC012875 | Anxa11 | 1.03 | annexin A11, mRNA (cDNA clone MGC:5899 IMAGE:3500318), complete cds. |
| BC018613 | Scamp5 | 1.03 | secretory carrier membrane protein 5, mRNA (cDNA clone MGC:27646 IMAGE:4511417), complete cds. |
| BC019386 | Vti1a | 1.03 | vesicle transport through interaction with t-SNAREs homolog 1A (yeast), mRNA (cDNA clone MGC:30354 IMAGE:5012235), complete cds. |
| BC019420 | 5730437N04Rik | 1.03 | RIKEN cDNA 5730437N04 gene, mRNA (cDNA clone MGC:30293 IMAGE:5053733), complete cds. |
| BC023179 | Zfp772 | 1.03 | cDNA sequence BC023179, mRNA (cDNA clone MGC:37070 IMAGE:4951074), complete cds. |
| BC023922 | Osr1 | 1.03 | odd-skipped related 1 (Drosophila), mRNA (cDNA clone MGC:36055 IMAGE:5323948), complete cds. |
| BC024788 | Tmem38a | 1.03 | transmembrane protein 38A, mRNA (cDNA clone MGC:36643 IMAGE:5360106), complete cds. |
| BC026547 | Olfm1 | 1.03 | olfactomedin 1, mRNA (cDNA clone MGC:35933 IMAGE:5036675), complete cds. |
| BC028994 | Ythdf2 | 1.03 | YTH domain family 2, mRNA (cDNA clone MGC:36628 IMAGE:5355331), complete cds. |
| BC031420 | Itm2a | 1.03 | integral membrane protein 2A, mRNA (cDNA clone MGC:18323 IMAGE:3668557), complete cds. |
| BC033455 | 0610007L01Rik | 1.03 | RIKEN cDNA 0610007L01 gene, mRNA (cDNA clone MGC:28353 IMAGE:4018584), complete cds. |
| BC034680 | Etv5 | 1.03 | ets variant gene 5, mRNA (cDNA clone MGC:28414 IMAGE:4036564), complete cds. |
| BC043474 | Tmem121 | 1.03 | transmembrane protein 121, mRNA (cDNA clone MGC:49374 IMAGE:5364389), complete cds. |
| BC048715 | Wfikkn1 | 1.03 | WAP, FS, Ig, KU, and NTR-containing protein 1, mRNA (cDNA clone IMAGE:6333959), containing frame-shift errors. |
| BC049603 | Sergef | 1.03 | secretion regulating guanine nucleotide exchange factor, mRNA (cDNA clone MGC:58261 IMAGE:6591072), complete cds. |
| BC050901 | Bap1 | 1.03 | Brca1 associated protein 1, mRNA (cDNA clone MGC:63252 IMAGE:6402443), complete cds. |
| BC051133 | Rgs7 | 1.03 | regulator of G protein signaling 7, mRNA (cDNA clone MGC:58271 IMAGE:6588901), complete cds. |
| BC052013 | Pcsk2 | 1.03 | proprotein convertase subtilisin/kexin type 2, mRNA (cDNA clone MGC:62349 IMAGE:5707923), complete cds. |
| BC052384 | Lingo1 | 1.03 | leucine rich repeat and Ig domain containing 1, mRNA (cDNA clone MGC:57943 IMAGE:5703857), complete cds. |
| BC054462 | Slc17a7 | 1.03 | solute carrier family 17 (sodium-dependent inorganic phosphate cotransporter), member 7, mRNA (cDNA clone MGC:62859 IMAGE:6494471), complete cds. |
| BC055854 | Prss22 | 1.03 | protease, serine, 22, mRNA (cDNA clone MGC:67911 IMAGE:4035351), complete cds. |
| BC058420 | BC058420 | 1.03 | cDNA sequence BC058420, mRNA (cDNA clone IMAGE:5687872), partial cds. |
| BC058962 | Nptxr | 1.03 | neuronal pentraxin receptor, mRNA (cDNA clone MGC:66862 IMAGE:6826075), complete cds. |
| BC062918 | Slc39a10 | 1.03 | solute carrier family 39 (zinc transporter), member 10, mRNA (cDNA clone MGC:65685 IMAGE:6833098), complete cds. |
| BC064708 | Fcrla | 1.03 | Fc receptor-like A, mRNA (cDNA clone MGC:74101 IMAGE:30301438), complete cds. |
| BC065696 | Lingo1 | 1.03 | leucine rich repeat and Ig domain containing 1, mRNA (cDNA clone MGC:69680 IMAGE:5685897), complete cds. |
| BC066111 | Car10 | 1.03 | carbonic anhydrase 10, mRNA (cDNA clone MGC:90026 IMAGE:30360743), complete cds. |
| BC066199 | Slc30a3 | 1.03 | solute carrier family 30 (zinc transporter), member 3, mRNA (cDNA clone MGC:76696 IMAGE:30476359), complete cds. |
| BC068130 | Limd2 | 1.03 | LIM domain containing 2, mRNA (cDNA clone MGC:92969 IMAGE:6413206), complete cds. |
| BC090628 | Pde1a | 1.03 | phosphodiesterase 1A, calmodulin-dependent, mRNA (cDNA clone MGC:116577 IMAGE:6842675), complete cds. |
| BC092138 | Hist1h2bh | 1.03 | histone cluster 1, H2bh, mRNA (cDNA clone MGC:106612 IMAGE:30613720), complete cds. |
| BC094450 | Mpv17l | 1.03 | Mpv17 transgene, kidney disease mutant-like, mRNA (cDNA clone MGC:102388 IMAGE:4973897), complete cds. |
| AK032947 | Meis1 | 1.02 | Myeloid ecotropic viral integration site 1, full insert sequence. |
| BC145694 | Klrc1 | 1.02 | Killer cell lectin-like receptor subfamily C, member 1, mRNA (cDNA clone MGC:175581 IMAGE:40130997), complete cds. |
| BC152835 | B3gnt6 | 1.02 | UDP-GlcNAc:betaGal beta-1,3-N-acetylglucosaminyltransferase 6 (core 3 synthase) (B3gnt6) mRNA, encodes complete protein. |
| NG_008461.1 | BSCL2 | 1.02 | Berardinelli-Seip congenital lipodystrophy 2 (seipin) |
| NM_004674.4 | ASH2L | 1.02 | ash2 (absent, small, or homeotic)-like (Drosophila) |
| NM_001037335.2 | HELZ2 | 1.02 | helicase with zinc finger 2, transcriptional coactivator |
| NM_203406.1 | MBLAC2 | 1.02 | metallo-beta-lactamase domain containing 2 |
| NM_001008220.1 | CPLX2 | 1.02 | complexin 2 |
| NG_021388.1 | ZNF280C | 1.02 | zinc finger protein 280C |
| NM_030920.4 | ANP32E | 1.02 | acidic (leucine-rich) nuclear phosphoprotein 32 family, member E |
| NC_018928.2 | NME1-NME2 | 1.02 | NME1-NME2 readthrough |
| NG_042065.1 | CALM2 | 1.02 | calmodulin 2 (phosphorylase kinase, delta) |
| NG_029728.1 | PRKACB | 1.02 | protein kinase, cAMP-dependent, catalytic, beta |
| NM_001010887.2 | ACER2 | 1.02 | alkaline ceramidase 2 |
| BC156263 | Cdk5r2 | 1.02 | cyclin-dependent kinase 5, regulatory subunit 2 (p39) (Cdk5r2) mRNA, encodes complete protein. |
| BC156784 | Gm6320 | 1.02 | predicted gene, EG622408 (EG622408) mRNA, encodes complete protein. |
| L47549 | Tnnt2 | 1.02 | cardiac troponin T isoform A1b mRNA, complete cds. |
| NM_001111030 | Acvr1c | 1.02 | activin A receptor, type IC (Acvr1c), transcript variant 1, mRNA. |
| NM_029441 | Cdyl2 | 1.02 | chromodomain protein, Y chromosome-like 2 (Cdyl2), mRNA. |
| U81453 | Myo7a | 1.02 | myosin VIIa (MyoVIIa) mRNA, complete cds. |
| XM_001474490 | Gm2707 | 1.02 | MAS-related GPR, member A4 (LOC100040314), mRNA. |
| AB194411 | Magi1 | 1.02 | MAGI1 mRNA for MAGI1a, complete cds. |
| AB294520 | N/A | 1.02 | mRNA-like noncoding RNA, partial sequence, clone: Gomafu isofom#1. |
| AK004893 | Itih4 | 1.02 | inter alpha-trypsin inhibitor, heavy chain 4, full insert sequence. |
| AK004981 | Ero1lb | 1.02 | ENDOPLASMIC RETICULUM OXIDOREDUCTIN 1-LBETA homolog [Homo sapiens], full insert sequence. |
| AK008374 | Tinag | 1.02 | tubulointerstitial nephritis antigen, full insert sequence. |
| AK019229 | 2700046G09Rik | 1.02 | hypothetical protein, full insert sequence. |
| AK030106 | C530008M17Rik | 1.02 | similar to KIAA1211 PROTEIN (FRAGMENT) [Homo sapiens], full insert sequence. |
| AK030533 | Mtss1 | 1.02 | METASTASIS SUPPRESSOR PROTEIN homolog [Homo sapiens], full insert sequence. |
| AK030684 | BC023814 | 1.02 | weakly similar to CGI-41 PROTEIN [Homo sapiens], full insert sequence. |
| AK033676 | Plekhg1 | 1.02 | pleckstrin homology domain containing, family G (with RhoGef domain) member 1, full insert sequence. |
| AK042123 | Ppp1r16b | 1.02 | protein phosphatase 1 regulatory subunit 16B (Ppp1r16b) mRNA, full insert sequence. |
| AK045995 | Rab37 | 1.02 | RAB37, member of RAS oncogene family, full insert sequence. |
| AK049264 | C330018A13Rik | 1.02 | unclassifiable, full insert sequence. |
| AK084270 | A630065K11Rik | 1.02 | unclassifiable, full insert sequence. |
| AK087218 | 9530082P21Rik | 1.02 | unclassifiable, full insert sequence. |
| AK122358 | Baiap3 | 1.02 | mKIAA0734 protein. |
| AK134563 | Evl | 1.02 | Ena-vasodilator stimulated phosphoprotein, full insert sequence. |
| AK137991 | Pdlim2 | 1.02 | PDZ and LIM domain 2, full insert sequence. |
| AK140017 | N/A | 1.02 | unclassifiable, full insert sequence. |
| AK140246 | Scfd2 | 1.02 | sec1 family domain containing 2, full insert sequence. |
| AK141492 | 2810002D19Rik | 1.02 | unclassifiable, full insert sequence. |
| AK143052 | Isl1 | 1.02 | ISL1 transcription factor, LIM/homeodomain (islet 1), full insert sequence. |
| AK147921 | Gpr137b | 1.02 | transmembrane 7 superfamily member 1, full insert sequence. |
| AK160556 | Ssbp1 | 1.02 | single-stranded DNA binding protein 1, full insert sequence. |
| AK166518 | Rgs9 | 1.02 | regulator of G-protein signaling 9, full insert sequence. |
| AK166575 | Thsd7b | 1.02 | weakly similar to Hypothetical protein GS164B05.1 in chromosome 7 (Fragment) [Homo sapiens], full insert sequence. |
| BC010980 | Rgs16 | 1.02 | regulator of G-protein signaling 16, mRNA (cDNA clone MGC:13876 IMAGE:4020813), complete cds. |
| BC013541 | Calml4 | 1.02 | calmodulin-like 4, mRNA (cDNA clone MGC:19226 IMAGE:4241711), complete cds. |
| BC017646 | Calb2 | 1.02 | calbindin 2, mRNA (cDNA clone MGC:28989 IMAGE:4527074), complete cds. |
| BC024354 | Pcbd1 | 1.02 | pterin 4 alpha carbinolamine dehydratase/dimerization cofactor of hepatocyte nuclear factor 1 alpha (TCF1) 1, mRNA (cDNA clone MGC:35787 IMAGE:5052788), complete cds. |
| BC024416 | N/A | 1.02 | predicted gene, EG668525, mRNA (cDNA clone MGC:36379 IMAGE:4988668), complete cds. |
| BC024633 | Arhgap12 | 1.02 | Rho GTPase activating protein 12, mRNA (cDNA clone IMAGE:4038635), complete cds. |
| BC024744 | Hmgcs2 | 1.02 | 3-hydroxy-3-methylglutaryl-Coenzyme A synthase 2, mRNA (cDNA clone MGC:29975 IMAGE:5123821), complete cds. |
| BC027526 | Mlf1 | 1.02 | myeloid leukemia factor 1, mRNA (cDNA clone MGC:41163 IMAGE:1495721), complete cds. |
| BC027535 | Resp18 | 1.02 | regulated endocrine-specific protein 18, mRNA (cDNA clone MGC:41217 IMAGE:3376899), complete cds. |
| BC028751 | Capn12 | 1.02 | calpain 12, mRNA (cDNA clone MGC:41077 IMAGE:1314413), complete cds. |
| BC029200 | 1700019G17Rik | 1.02 | RIKEN cDNA 1700019G17 gene, mRNA (cDNA clone MGC:35619 IMAGE:3501234), complete cds. |
| BC031768 | Os9 | 1.02 | amplified in osteosarcoma, mRNA (cDNA clone MGC:25481 IMAGE:4487829), complete cds. |
| BC048167 | Stx8 | 1.02 | syntaxin 8, mRNA (cDNA clone MGC:61287 IMAGE:6821351), complete cds. |
| BC048759 | Lemd1 | 1.02 | LEM domain containing 1, mRNA (cDNA clone IMAGE:6706955). |
| BC049108 | Rcn1 | 1.02 | reticulocalbin 1, mRNA (cDNA clone MGC:61334 IMAGE:6414128), complete cds. |
| BC052093 | Adam1a | 1.02 | a disintegrin and metallopeptidase domain 1a, mRNA (cDNA clone MGC:62530 IMAGE:5695131), complete cds. |
| BC052380 | Trove2 | 1.02 | TROVE domain family, member 2, mRNA (cDNA clone MGC:64638 IMAGE:5697399), complete cds. |
| BC066790 | Tor1aip2 | 1.02 | torsin A interacting protein 2, mRNA (cDNA clone MGC:76434 IMAGE:30099444), complete cds. |
| BC085305 | Mfsd3 | 1.02 | major facilitator superfamily domain containing 3, mRNA (cDNA clone MGC:103068 IMAGE:6399056), complete cds. |
| BC086926 | Ttr | 1.02 | transthyretin, mRNA (cDNA clone MGC:107649 IMAGE:6749152), complete cds. |
| BC098327 | Htr2c | 1.02 | 5-hydroxytryptamine (serotonin) receptor 2C, mRNA (cDNA clone IMAGE:6841927), with apparent retained intron. |
| BC116682 | Sh2d4a | 1.02 | SH2 domain containing 4A, mRNA (cDNA clone MGC:144846 IMAGE:40106612), complete cds. |
| BC119517 | Il1f8 | 1.02 | interleukin 1 family, member 8, mRNA (cDNA clone MGC:141156 IMAGE:40055701), complete cds. |
| BC125514 | Obp1a | 1.02 | odorant binding protein Ia, mRNA (cDNA clone MGC:159217 IMAGE:40130029), complete cds. |
| BC127959 | Olfr813 | 1.02 | olfactory receptor 813, mRNA (cDNA clone MGC:157500 IMAGE:40134789), complete cds. |
| BC132117 | Khdrbs2 | 1.02 | KH domain containing, RNA binding, signal transduction associated 2, mRNA (cDNA clone MGC:163748 IMAGE:40130394), complete cds. |
| BC132482 | Tmem202 | 1.02 | RIKEN cDNA 4930425N13 gene, mRNA (cDNA clone MGC:164113 IMAGE:40130759), complete cds. |
| BC132662 | Defb25 | 1.02 | defensin beta 25, mRNA (cDNA clone MGC:164293 IMAGE:40130939), complete cds. |
| BC145907 | Apod | 1.02 | apolipoprotein D, mRNA (cDNA clone MGC:175794 IMAGE:40131210), complete cds. |
| EF651798 | ENSMUSG00000079376 | 1.02 | alpha1-takusan mRNA, complete cds. |
| NM_001110496 | Tmem87a | 1.02 | transmembrane protein 87A (Tmem87a), transcript variant 2, mRNA. |
| NM_001110505 | Amy1 | 1.02 | amylase 1, salivary (Amy1), transcript variant 2, mRNA. |
| NM_001111279 | Wdfy1 | 1.02 | WD repeat and FYVE domain containing 1 (Wdfy1), transcript variant 1, mRNA. |
| NM_016956 | Hbb-b2 | 1.02 | hemoglobin, beta adult minor chain (Hbb-b2), mRNA. |
| XM_001473341 | Gm2371 | 1.02 | similar to pol protein (LOC100039687), mRNA. |
| AK005807 | 4930588J15Rik | 1.02 | unclassifiable, full insert sequence. |
| AK014343 | Tcf4 | 1.02 | transcription factor 4, full insert sequence. |
| AK016873 | Dlgap1 | 1.02 | DISKS LARGE-ASSOCIATED PROTEIN 1 (DAP-1) (GUANYLATE KINASE-ASSOCIATED PROTEIN) (SAP90/PSD-95-ASSOCIATED PROTEIN 1) (SAPAP1) (PSD-95/SAP90 BINDING PROTEIN 1) (FRAGMENT) homolog [Mus musculus], full insert sequence. |
| AK019114 | 2400009B08Rik | 1.02 | unclassifiable, full insert sequence. |
| AK019154 | 2610017I09Rik | 1.02 | hypothetical protein, full insert sequence. |
| AK028275 | Ppm1l | 1.02 | hypothetical Protein phosphatase 2C domain containing protein, full insert sequence. |
| AK052020 | Etl4 | 1.02 | KIAA1217 PROTEIN (FRAGMENT) [Homo sapiens], full insert sequence. |
| AK082292 | Lsm11 | 1.02 | hypothetical Small nuclear ribonucleoprotein (Sm protein) containing protein, full insert sequence. |
| AK082388 | Enox1 | 1.02 | HYPOTHETICAL 73.3 KDA PROTEIN homolog [Homo sapiens], full insert sequence. |
| AK086078 | Etl4 | 1.02 | uknown protein mapping to human hypothetical protein DKFZp761L0424, full insert sequence. |
| AK087448 | Ppfia2 | 1.02 | hypothetical SAM domain (Sterile alpha motif) containing protein, full insert sequence. |
| AK087950 | N/A | 1.02 | T-cell receptor beta, variable 13, full insert sequence. |
| AK088204 | Satb1 | 1.02 | special AT-rich sequence binding protein 1, full insert sequence. |
| AK088395 | Plekha2 | 1.02 | pleckstrin homology domain-containing, family A (phosphoinositide binding specific) member 2andem PH Domain containing protein 2, full insert sequence. |
| AK122458 | Nav1 | 1.02 | mKIAA1151 protein. |
| AK132676 | Osbpl1a | 1.02 | oxysterol binding protein-like 1A, full insert sequence. |
| AK143444 | Hivep2 | 1.02 | human immunodeficiency virus type I enhancer binding protein 2, full insert sequence. |
| AK146098 | Ppp1r13l | 1.02 | hypothetical SH3/Ankyrin/Proline-rich region profile containing protein, full insert sequence. |
| AK147396 | Hspg2 | 1.02 | perlecan (heparan sulfate proteoglycan 2), full insert sequence. |
| AK148135 | Jph4 | 1.02 | Junctophilin-like 1 protein, full insert sequence. |
| AK151523 | AI506816 | 1.02 | unclassifiable, full insert sequence. |
| AK153638 | Rnf43 | 1.02 | Hypothetical serine-rich region/histidine-rich region/RING finger containing protein homolog [Mus musculus], full insert sequence. |
| AK158740 | Mpped1 | 1.02 | hypothetical protein, full insert sequence. |
| AK159410 | Arpc4 | 1.02 | actin related protein 2/3 complex, subunit 4, full insert sequence. |
| AK159977 | Gpr177 | 1.02 | Putative NFkB activating protein homolog [Homo sapiens], full insert sequence. |
| AK171427 | Anpep | 1.02 | B6-derived CD11 +ve dendritic cells cDNA, RIKEN full-length enriched library, clone:F730214N21 product:alanyl (membrane) aminopeptidase, full insert sequence. |
| AK220219 | Urb1 | 1.02 | mKIAA0539 protein. |
| AK220445 | Gpd1 | 1.02 | mKIAA4010 protein. |
| AY138505 | Rgs19 | 1.02 | GAIP/RGS19, short isoform mRNA, complete cds. |
| AY756066 | Pfkfb4 | 1.02 | alt 'C230090D14' |
| AY861418 | Pcdh1 | 1.02 | alt '2010005A06Rik#AI585920' |
| BC002215 | Dkkl1 | 1.02 | dickkopf-like 1, mRNA (cDNA clone MGC:7472 IMAGE:3490482), complete cds. |
| BC003869 | Dusp6 | 1.02 | dual specificity phosphatase 6, mRNA (cDNA clone MGC:6625 IMAGE:3491528), complete cds. |
| BC010278 | Lmo4 | 1.02 | LIM domain only 4, mRNA (cDNA clone MGC:5732 IMAGE:3589532), complete cds. |
| BC011306 | H2-K1 | 1.02 | histocompatibility 2, K1, K region, mRNA (cDNA clone MGC:7052 IMAGE:3156482), complete cds. |
| BC013539 | Igh | 1.02 | immunoglobulin heavy chain complex, mRNA (cDNA clone MGC:19223 IMAGE:4241603), complete cds. |
| BC014822 | Ephb3 | 1.02 | Eph receptor B3, mRNA (cDNA clone MGC:18409 IMAGE:3673003), complete cds. |
| BC016632 | Cplx3 | 1.02 | complexin 3, mRNA (cDNA clone MGC:21420 IMAGE:4500804), complete cds. |
| BC017688 | Bcat2 | 1.02 | branched chain aminotransferase 2, mitochondrial, mRNA (cDNA clone MGC:19230 IMAGE:4242224), complete cds. |
| BC018508 | Exd2 | 1.02 | exonuclease 3''-5'' domain-like 2, mRNA (cDNA clone MGC:27781 IMAGE:3156447), complete cds. |
| BC018517 | Prss23 | 1.02 | protease, serine, 23, mRNA (cDNA clone MGC:27987 IMAGE:3597686), complete cds. |
| BC019118 | Rab6 | 1.02 | RAB6, member RAS oncogene family, mRNA (cDNA clone MGC:29434 IMAGE:3711087), complete cds. |
| BC020015 | Apln | 1.02 | apelin, mRNA (cDNA clone MGC:27819 IMAGE:3483588), complete cds. |
| BC021342 | Smad2 | 1.02 | MAD homolog 2 (Drosophila), mRNA (cDNA clone MGC:29095 IMAGE:5066237), complete cds. |
| BC023050 | Slc10a3 | 1.02 | solute carrier family 10 (sodium/bile acid cotransporter family), member 3, mRNA (cDNA clone MGC:38638 IMAGE:5355849), complete cds. |
| BC024480 | Aimp2 | 1.02 | JTV1 gene, mRNA (cDNA clone MGC:37389 IMAGE:4977193), complete cds. |
| BC025795 | Ugt2a3 | 1.02 | UDP glucuronosyltransferase 2 family, polypeptide A3, mRNA (cDNA clone MGC:35622 IMAGE:5133743), complete cds. |
| BC025888 | Sidt1 | 1.02 | SID1 transmembrane family, member 1, mRNA (cDNA clone MGC:32412 IMAGE:5039343), complete cds. |
| BC026375 | Gpnmb | 1.02 | glycoprotein (transmembrane) nmb, mRNA (cDNA clone MGC:31121 IMAGE:4164706), complete cds. |
| BC026465 | Tubd1 | 1.02 | tubulin, delta 1, mRNA (cDNA clone MGC:31477 IMAGE:4484099), complete cds. |
| BC027291 | Itpka | 1.02 | inositol 1,4,5-trisphosphate 3-kinase A, mRNA (cDNA clone MGC:28924 IMAGE:3481738), complete cds. |
| BC027805 | Ephb3 | 1.02 | Eph receptor B3, mRNA (cDNA clone IMAGE:5322161), with apparent retained intron. |
| BC028487 | Cck | 1.02 | cholecystokinin, mRNA (cDNA clone MGC:41001 IMAGE:1400830), complete cds. |
| BC028664 | Prkdc | 1.02 | protein kinase, DNA activated, catalytic polypeptide, mRNA (cDNA clone IMAGE:1345935), with apparent retained intron. |
| BC031419 | AI480653 | 1.02 | expressed sequence AI480653, mRNA (cDNA clone MGC:19367 IMAGE:2615706), complete cds. |
| BC031507 | Khdrbs3 | 1.02 | KH domain containing, RNA binding, signal transduction associated 3, mRNA (cDNA clone MGC:21726 IMAGE:4500976), complete cds. |
| BC031781 | BC031781 | 1.02 | cDNA sequence BC031781, mRNA (cDNA clone MGC:30618 IMAGE:3672316), complete cds. |
| BC034400 | Lztr1 | 1.02 | leucine-zipper-like transcriptional regulator, 1, mRNA (cDNA clone MGC:35711 IMAGE:2651456), complete cds. |
| BC037194 | Ogt | 1.02 | O-linked N-acetylglucosamine (GlcNAc) transferase (UDP-N-acetylglucosamine:polypeptide-N-acetylglucosaminyl transferase), mRNA (cDNA clone IMAGE:4500866), complete cds. |
| BC043012 | Npy | 1.02 | neuropeptide Y, mRNA (cDNA clone MGC:57879 IMAGE:5683102), complete cds. |
| BC043106 | 4933427D14Rik | 1.02 | RIKEN cDNA 4933427D14 gene, mRNA (cDNA clone MGC:58026 IMAGE:6406803), complete cds. |
| BC049737 | Ypel1 | 1.02 | yippee-like 1 (Drosophila), mRNA (cDNA clone MGC:58754 IMAGE:6771362), complete cds. |
| BC052737 | Tbr1 | 1.02 | T-box brain gene 1, mRNA (cDNA clone MGC:64688 IMAGE:6825394), complete cds. |
| BC055484 | Chd3 | 1.02 | chromodomain helicase DNA binding protein 3, mRNA (cDNA clone IMAGE:4459032), complete cds. |
| BC057087 | Dnajb5 | 1.02 | DnaJ (Hsp40) homolog, subfamily B, member 5, mRNA (cDNA clone MGC:73423 IMAGE:5695243), complete cds. |
| BC057678 | Pik3c3 | 1.02 | phosphoinositide-3-kinase, class 3, mRNA (cDNA clone MGC:68341 IMAGE:3496633), complete cds. |
| BC058084 | Unc5a | 1.02 | unc-5 homolog A (C. elegans), mRNA (cDNA clone MGC:66671 IMAGE:6813463), complete cds. |
| BC058355 | N/A | 1.02 | MAP/microtubule affinity-regulating kinase 4, mRNA (cDNA clone IMAGE:6826411), **** WARNING: chimeric clone ****. |
| BC058965 | Neurod2 | 1.02 | neurogenic differentiation 2, mRNA (cDNA clone MGC:67032 IMAGE:6817440), complete cds. |
| BC059216 | Tmem44 | 1.02 | transmembrane protein 44, mRNA (cDNA clone IMAGE:6408767). |
| BC061247 | Crhbp | 1.02 | corticotropin releasing hormone binding protein, mRNA (cDNA clone MGC:74402 IMAGE:30246702), complete cds. |
| BC062101 | E2f2 | 1.02 | E2F transcription factor 2, mRNA (cDNA clone MGC:69659 IMAGE:6848237), complete cds. |
| BC063251 | Rnf26 | 1.02 | ring finger protein 26, mRNA (cDNA clone MGC:66654 IMAGE:6413980), complete cds. |
| BC064678 | Itch | 1.02 | itchy, E3 ubiquitin protein ligase, mRNA (cDNA clone MGC:67157 IMAGE:6409535), complete cds. |
| BC066996 | Gal3st3 | 1.02 | galactose-3-O-sulfotransferase 3, mRNA (cDNA clone MGC:90145 IMAGE:5707267), complete cds. |
| BC068241 | Cts8 | 1.02 | cathepsin 8, mRNA (cDNA clone MGC:60501 IMAGE:30019120), complete cds. |
| BC068313 | Itga4 | 1.02 | integrin alpha 4, mRNA (cDNA clone MGC:76683 IMAGE:30093248), complete cds. |
| BC070452 | Pitpnm2 | 1.02 | phosphatidylinositol transfer protein, membrane-associated 2, mRNA (cDNA clone MGC:99420 IMAGE:30621943), complete cds. |
| BC087946 | Cnrip1 | 1.02 | cannabinoid receptor interacting protein 1, mRNA (cDNA clone MGC:107336 IMAGE:6591410), complete cds. |
| BC089501 | Zmat2 | 1.02 | zinc finger, matrin type 2, mRNA (cDNA clone MGC:107181 IMAGE:6594761), complete cds. |
| BC089539 | Gm5506 | 1.02 | predicted gene, EG433182, mRNA (cDNA clone MGC:107267 IMAGE:6707317), complete cds. |
| BC094461 | Jup | 1.02 | junction plakoglobin, mRNA (cDNA clone MGC:103220 IMAGE:4459882), complete cds. |
| BC096471 | Flrt2 | 1.02 | fibronectin leucine rich transmembrane protein 2, mRNA (cDNA clone MGC:106049 IMAGE:3582155), complete cds. |
| AK033676 | Plekhg1 | 1.01 | Pleckstrin homology domain containing, family G (with RhoGef domain) member 1, full insert sequence. |
| NG_016169.1 | CITED2 | 1.01 | Cbp/p300-interacting transactivator, with Glu/Asp-rich carboxy-terminal domain, 2 |
| NG_034254.1 | CCND2 | 1.01 | cyclin D2 |
| NM_012482.4 | ZNF281 | 1.01 | zinc finger protein 281 |
| NM_001164211.1 | LRCH1 | 1.01 | leucine-rich repeats and calponin homology (CH) domain containing 1 |
| NG_033082.1 | AP1S1 | 1.01 | adaptor-related protein complex 1, sigma 1 subunit |
| NM_001358.2 | DHX15 | 1.01 | DEAH (Asp-Glu-Ala-His) box helicase 15 |
| NG_032988.1 | MEOX2 | 1.01 | mesenchyme homeobox 2 |
| NM_001286150.1 | BPNT1 | 1.01 | 3'(2'), 5'-bisphosphate nucleotidase 1 |
| NG_028007.1 | MAP4K3 | 1.01 | mitogen-activated protein kinase kinase kinase kinase 3 |
| NM_174907.3 | PPP4R2 | 1.01 | protein phosphatase 4, regulatory subunit 2 |
| NG_016808.1 | CBL | 1.01 | Cbl proto-oncogene, E3 ubiquitin protein ligase |
| NM_001310.3 | CREBL2 | 1.01 | cAMP responsive element binding protein-like 2 |
| NM_152835.4 | PDIK1L | 1.01 | PDLIM1 interacting kinase 1 like |
| BC156318 | Rhou | 1.01 | ras homolog gene family, member U (Rhou) mRNA, encodes complete protein. |
| BC156809 | Sema4b | 1.01 | sema domain, immunoglobulin domain (Ig), transmembrane domain (TM) and short cytoplasmic domain, (semaphorin) 4B (Sema4b) mRNA, encodes complete protein. |
| L12367 | Cap1 | 1.01 | adenylyl cyclase-associated protein (CAP) mRNA, complete cds. |
| NM_001101588 | Cyp4f40 | 1.01 | cytochrome P450, family 4, subfamily f, polypeptide 40 (Cyp4f40), mRNA. |
| NM_172505 | A730008H23Rik | 1.01 | RIKEN cDNA A730008H23 gene (A730008H23Rik), mRNA. |
| NR_003519 | Pisd-ps2 | 1.01 | RIKEN cDNA D030013I16 gene (D030013I16Rik) on chromosome 17. |
| XM_001000857 | Tchh | 1.01 | trichohyalin (Tchh), mRNA. |
| XM_001474559 | Gm2738 | 1.01 | similar to retinitis pigmentosa GTPase regulator interacting protein 1 (LOC100040369), mRNA. |
| XM_001474997 | Wdr64 | 1.01 | WD repeat domain 64 (Wdr64), mRNA. |
| XM_001476161 | Epb4.1l4b | 1.01 | erythrocyte protein band 4.1-like 4b, transcript variant 1 (Epb4.1l4b), mRNA. |
| AF107298 | Tcf7l2 | 1.01 | HMG-box transcription factor TCF4B (Tcf4) mRNA, complete cds. |
| AF475074 | Ntng1 | 1.01 | domesticus laminet-1F (Lmnt1) mRNA, complete cds |
| AF498302 | Rian | 1.01 | BALB/c imprinted mMeg8/Irm variant 2 mRNA, partial sequence. |
| AK009258 | D16Ertd472e | 1.01 | weakly similar to EURL PROTEIN [Gallus gallus], full insert sequence. |
| AK018427 | 8430419K02Rik | 1.01 | hypothetical protein, full insert sequence. |
| AK019022 | Foxa3 | 1.01 | forkhead box A3, full insert sequence. |
| AK029544 | Ccdc81 | 1.01 | RIKEN cDNA 4921513D09 gene, full insert sequence. |
| AK034086 | Hmgxb3 | 1.01 | hypothetical HMG1/2 (high mobility group) box containing protein, full insert sequence. |
| AK034291 | Syp | 1.01 | synaptophysin, full insert sequence. |
| AK041755 | N/A | 1.01 | cadherin 1, full insert sequence. |
| AK041893 | N/A | 1.01 | hypothetical protein, full insert sequence. |
| AK045744 | Tmem181d-ps | 1.01 | unclassifiable, full insert sequence. |
| AK049692 | Sparc | 1.01 | secreted acidic cysteine rich glycoprotein, full insert sequence. |
| AK086738 | Tmem87a | 1.01 | hypothetical protein, full insert sequence. |
| AK086923 | Heph | 1.01 | hephaestin, full insert sequence. |
| AK122515 | Sema6d | 1.01 | mKIAA1479 protein. |
| AK131825 | 1810020O05Rik | 1.01 | unclassifiable, full insert sequence. |
| AK132176 | Gab1 | 1.01 | growth factor receptor bound protein 2-associated protein 1, full insert sequence. |
| AK134467 | Ntng1 | 1.01 | netrin G1, full insert sequence. |
| AK135381 | Cd82 | 1.01 | kangai 1 (suppression of tumorigenicity 6, prostate), full insert sequence. |
| AK135487 | 6820431F20Rik | 1.01 | unclassifiable, full insert sequence. |
| AK137390 | Pigq | 1.01 | phosphatidylinositol glycan, class Q, full insert sequence. |
| AK144111 | Itgb4 | 1.01 | integrin beta 4, full insert sequence. |
| AK147906 | N/A | 1.01 | inositol hexaphosphate kinase 1, full insert sequence. |
| AK148054 | Neat1 | 1.01 | unclassifiable, full insert sequence. |
| AK153563 | Prdm16 | 1.01 | PR domain containing 16, full insert sequence. |
| AK159365 | Ecel1 | 1.01 | endothelin converting enzyme-like 1, full insert sequence. |
| AK166114 | Slc9a2 | 1.01 | Sodium/hydrogen exchanger 2 (Na(+)/H(+) exchanger 2) (NHE-2) homolog [Homo sapiens], full insert sequence. |
| AK170980 | Ap2a2 | 1.01 | adaptor protein complex AP-2, alpha 2 subunit, full insert sequence. |
| AK220516 | Camk2d | 1.01 | mKIAA4163 protein. |
| AY341881 | Rps6ka5 | 1.01 | ribosomal protein S6 kinase splice variant 5 mRNA, complete cds |
| BC005679 | Sdc4 | 1.01 | syndecan 4, mRNA (cDNA clone MGC:11456 IMAGE:3154160), complete cds. |
| BC008262 | Snrpe | 1.01 | small nuclear ribonucleoprotein E, mRNA (cDNA clone MGC:6518 IMAGE:2650413), complete cds. |
| BC011116 | Plin3 | 1.01 | mannose-6-phosphate receptor binding protein 1, mRNA (cDNA clone MGC:19018 IMAGE:4023354), complete cds. |
| BC011208 | Tmem98 | 1.01 | transmembrane protein 98, mRNA (cDNA clone MGC:19304 IMAGE:4163101), complete cds. |
| BC012966 | Eed | 1.01 | embryonic ectoderm development, mRNA (cDNA clone MGC:13909 IMAGE:3991086), complete cds. |
| BC013651 | Serpina3n | 1.01 | serine (or cysteine) peptidase inhibitor, clade A, member 3N, mRNA (cDNA clone MGC:18516 IMAGE:4160649), complete cds. |
| BC015077 | Phf1 | 1.01 | cDNA clone IMAGE:4007482, containing frame-shift errors. |
| BC024534 | Pllp | 1.01 | plasma membrane proteolipid, mRNA (cDNA clone MGC:37565 IMAGE:4987882), complete cds. |
| BC027525 | Reg3b | 1.01 | pancreatitis-associated protein, mRNA (cDNA clone MGC:41159 IMAGE:3471932), complete cds. |
| BC027551 | Krtap13-1 | 1.01 | keratin associated protein 13-1, mRNA (cDNA clone MGC:41371 IMAGE:1363815), complete cds. |
| BC031854 | D14Ertd449e | 1.01 | DNA segment, Chr 14, ERATO Doi 449, expressed, mRNA (cDNA clone MGC:25850 IMAGE:4194400), complete cds. |
| BC031918 | Gpr37 | 1.01 | G protein-coupled receptor 37, mRNA (cDNA clone MGC:36067 IMAGE:5364562), complete cds. |
| BC040774 | C1ql2 | 1.01 | complement component 1, q subcomponent-like 2, mRNA (cDNA clone MGC:49730 IMAGE:4503096), complete cds. |
| BC045152 | Lect1 | 1.01 | leukocyte cell derived chemotaxin 1, mRNA (cDNA clone MGC:54653 IMAGE:6439864), complete cds. |
| BC047997 | Rnaseh2b | 1.01 | ribonuclease H2, subunit B, mRNA (cDNA clone MGC:59603 IMAGE:6510494), complete cds. |
| BC049071 | Zcchc12 | 1.01 | zinc finger, CCHC domain containing 12, mRNA (cDNA clone MGC:61184 IMAGE:5701407), complete cds. |
| BC049235 | Gpx3 | 1.01 | glutathione peroxidase 3, mRNA (cDNA clone MGC:54712 IMAGE:4221025), complete cds. |
| BC052790 | Ngdn | 1.01 | neuroguidin, EIF4E binding protein, mRNA (cDNA clone MGC:63070 IMAGE:1515292), complete cds. |
| BC055099 | Zfp474 | 1.01 | zinc finger protein 474, mRNA (cDNA clone MGC:58179 IMAGE:6704066), complete cds. |
| BC059775 | Rhog | 1.01 | ras homolog gene family, member G, mRNA (cDNA clone MGC:68130 IMAGE:5370916), complete cds. |
| BC066034 | Gpbp1l1 | 1.01 | GC-rich promoter binding protein 1-like 1, mRNA (cDNA clone MGC:90065 IMAGE:6836605), complete cds. |
| BC066095 | Nckap5 | 1.01 | RIKEN cDNA E030049G20 gene, mRNA (cDNA clone MGC:90076 IMAGE:6856184), complete cds. |
| BC087556 | Ass1 | 1.01 | Mus musculus argininosuccinate synthetase 1, mRNA (cDNA clone MGC:103151 IMAGE:6478590), complete cds. |
| BC089535 | Sfxn4 | 1.01 | sideroflexin 4, mRNA (cDNA clone MGC:107253 IMAGE:6589805), complete cds. |
| BC096605 | Chchd8 | 1.01 | coiled-coil-helix-coiled-coil-helix domain containing 8, mRNA (cDNA clone MGC:106079 IMAGE:5371531), complete cds. |
| BC098096 | Six3 | 1.01 | sine oculis-related homeobox 3 homolog (Drosophila), mRNA (cDNA clone MGC:106488 IMAGE:6402835), complete cds. |
| BC098487 | Creb5 | 1.01 | cAMP responsive element binding protein 5, mRNA (cDNA clone MGC:107455 IMAGE:30090687), complete cds. |
| BC099446 | Gkn1 | 1.01 | gastrokine 1, mRNA (cDNA clone MGC:117639 IMAGE:30521253), complete cds. |
| BC114997 | Pfas | 1.01 | phosphoribosylformylglycinamidine synthase (FGAR amidotransferase), mRNA (cDNA clone MGC:118146 IMAGE:30110327), complete cds. |
| BC117963 | Clasp2 | 1.01 | CLIP associating protein 2, mRNA (cDNA clone MGC:144153 IMAGE:40098296), complete cds. |
| BC125460 | Lpar3 | 1.01 | endothelial differentiation, lysophosphatidic acid G-protein-coupled receptor 7, mRNA (cDNA clone MGC:159163 IMAGE:40129975), complete cds. |
| BC132120 | Cbln1 | 1.01 | cerebellin 1 precursor protein, mRNA (cDNA clone MGC:163751 IMAGE:40130397), complete cds. |
| BC145918 | Defb48 | 1.01 | predicted gene, EG432867, mRNA (cDNA clone MGC:175805 IMAGE:40131221), complete cds. |
| NM_001017393 | MGC107098 | 1.01 | similar to spermiogenesis specific transcript on the Y 2 (MGC107098), mRNA. |
| NM_020279 | Ccl28 | 1.01 | chemokine (C-C motif) ligand 28 (Ccl28), mRNA. |
| XM_001471946 | Msi2 | 1.01 | similar to Musashi homolog 2 (Drosophila) (LOC100038945), mRNA. |
| XM_001473508 | Gm12407 | 1.01 | similar to EBI-1 ligand chemokine (LOC100039789), mRNA. |
| AB080741 | Car10 | 1.01 | Car10 mRNA for carbonic anhydrase-related protein X, complete cds. |
| AB093290 | 3110047P20Rik | 1.01 | mKIAA1239 protein. |
| AF321853 | Chrdl1 | 1.01 | ventroptin-alpha mRNA, complete cds. |
| AK004405 | Xrcc6bp1 | 1.01 | similar to Ku70-binding protein (Fragment) [Homo sapiens], full insert sequence. |
| AK013520 | Necab3 | 1.01 | amyloid beta (A4) precursor protein-binding, family A, member 1 binding protein, full insert sequence. |
| AK016788 | 4933412E12Rik | 1.01 | unclassifiable, full insert sequence. |
| AK029106 | Trappc9 | 1.01 | similar to KIAA1882 PROTEIN (FRAGMENT) [Homo sapiens], full insert sequence. |
| AK044080 | Cpne9 | 1.01 | COPINE-LIKE PROTEIN KIAA1599 [Homo sapiens], full insert sequence. |
| AK044487 | Ipo11 | 1.01 | RAN BINDING PROTEIN 11 [Homo sapiens], full insert sequence. |
| AK076803 | Pfkp | 1.01 | phosphofructokinase, platelet, full insert sequence. |
| AK080296 | 5530601H04Rik | 1.01 | RIKEN cDNA 5530601H04 gene, full insert sequence. |
| AK080405 | Mfap3 | 1.01 | MICROFIBRIL-ASSOCIATED GLYCOPROTEIN 3 PRECURSOR [Homo sapiens], full insert sequence. |
| AK081495 | March2 | 1.01 | hypothetical protein, full insert sequence. |
| AK082667 | Nav1 | 1.01 | hypothetical Serine-rich region/Treacher Collins syndrome protein Treacle containing protein, full insert sequence. |
| AK085945 | Lama4 | 1.01 | laminin, alpha 4, full insert sequence. |
| AK122426 | Trak1 | 1.01 | mKIAA1042 protein. |
| AK131826 | N/A | 1.01 | T-cell receptor alpha chain C region, full insert sequence. |
| AK143206 | Itpr2 | 1.01 | inositol 1,4,5-triphosphate receptor 2, full insert sequence. |
| AK144536 | Ttbk1 | 1.01 | TAU-tubulin kinase homolog (Fragment) [Mus musculus], full insert sequence. |
| AK152074 | Maf | 1.01 | avian musculoaponeurotic fibrosarcoma (v-maf) AS42 oncogene homolog, full insert sequence. |
| AK154247 | Coro2a | 1.01 | coronin, actin binding protein 2A, full insert sequence. |
| AK156629 | Fam53b | 1.01 | hypothetical protein, full insert sequence. |
| AK158820 | Fmnl1 | 1.01 | formin-like 1, full insert sequence. |
| AK161542 | Paqr7 | 1.01 | membrane progestin receptor alpha, full insert sequence. |
| AK167498 | Ctsh | 1.01 | cathepsin H, full insert sequence. |
| AK171222 | Trpv2 | 1.01 | NOD-derived CD11c +ve dendritic cells cDNA, RIKEN full-length enriched library, clone:F630311B03 product:transient receptor potential cation channel, subfamily V, member 2, full insert sequence. |
| BC004791 | 6330527O06Rik | 1.01 | RIKEN cDNA 6330527O06 gene, mRNA (cDNA clone MGC:8097 IMAGE:3588577), complete cds. |
| BC005472 | Cap1 | 1.01 | CAP, adenylate cyclase-associated protein 1 (yeast), mRNA (cDNA clone MGC:6739 IMAGE:3590890), complete cds. |
| BC019836 | Igfbp4 | 1.01 | insulin-like growth factor binding protein 4, mRNA (cDNA clone MGC:29917 IMAGE:5123738), complete cds. |
| BC020036 | Dusp18 | 1.01 | dual specificity phosphatase 18, mRNA (cDNA clone MGC:28218 IMAGE:3990751), complete cds. |
| BC021798 | Pparg | 1.01 | peroxisome proliferator activated receptor gamma, mRNA (cDNA clone MGC:18439 IMAGE:4191539), complete cds. |
| BC025097 | Ogt | 1.01 | O-linked N-acetylglucosamine (GlcNAc) transferase (UDP-N-acetylglucosamine:polypeptide-N-acetylglucosaminyl transferase), mRNA (cDNA clone IMAGE:4480930), with apparent retained intron. |
| BC027126 | 6430548M08Rik | 1.01 | RIKEN cDNA 6430548M08 gene, mRNA (cDNA clone MGC:38801 IMAGE:5359842), complete cds. |
| BC038126 | Entpd6 | 1.01 | ectonucleoside triphosphate diphosphohydrolase 6, mRNA (cDNA clone MGC:47939 IMAGE:1348160), complete cds. |
| BC047383 | Sfrs14 | 1.01 | cDNA clone IMAGE:5356975, containing frame-shift errors. |
| BC055803 | Etl4 | 1.01 | cDNA sequence BC026657, mRNA (cDNA clone IMAGE:6413543). |
| BC087831 | Neurod6 | 1.01 | neurogenic differentiation 6, mRNA (cDNA clone MGC:90713 IMAGE:6411826), complete cds. |
| BC140301 | Dgke | 1 | Diacylglycerol kinase, epsilon (Dgke) mRNA, encodes complete protein. |
| BC156341 | Hoxd8 | 1 | homeo box D8 (Hoxd8) mRNA, encodes complete protein. |
| BC156520 | Iqgap3 | 1 | IQ motif containing GTPase activating protein 3 (Iqgap3) mRNA, encodes complete protein. |
| AK040931 | Smoc1 | 1 | SPARC-RELATED PROTEIN homolog [Mus musculus], full insert sequence. |
| AK088441 | Rnf114 | 1 | zinc finger protein 313, full insert sequence. |
| AK172312 | Smoc2 | 1 | SPARC related modular calcium binding 2, full insert sequence. |
| BC130254 | Acer1 | 0.99 | N-acylsphingosine amidohydrolase (alkaline ceramidase) 3, mRNA (cDNA clone MGC:161323 IMAGE:40142524), complete cds. |
| BC132380 | Htra4 | 0.99 | HtrA serine peptidase 4, mRNA (cDNA clone MGC:164011 IMAGE:40130657), complete cds. |
| NM_001080396.2 | FAM155A | 0.99 | family with sequence similarity 155, member A |
| NG_033888.1 | DGKE | 0.99 | diacylglycerol kinase, epsilon 64kDa |
| NM_004148.3 | NINJ1 | 0.99 | ninjurin 1 |
| NM_001289413.1 | EHMT2 | 0.99 | euchromatic histone-lysine N-methyltransferase 2 |
| BC156227 | Igdcc4 | 0.99 | neighbor of Punc E11 (Nope) mRNA, encodes complete protein. |
| U56649 | Pde1a | 0.99 | cyclic nucleotide phosphodiesterase (PDE1A2) mRNA, complete cds. |
| AK004584 | N/A | 0.99 | glucocorticoid-induced leucine zipper, full insert sequence. |
| AK007524 | Fam32a | 0.99 | hypothetical protein, full insert sequence. |
| AK029333 | Iqce | 0.99 | hypothetical protein, full insert sequence. |
| AK039576 | Kif13a | 0.99 | kinesin 13A, full insert sequence. |
| AK044282 | Gucy2e | 0.99 | guanylate cyclase 2e, full insert sequence. |
| AK050963 | Ddc | 0.99 | dopa decarboxylase, full insert sequence. |
| AK051045 | Snhg1 | 0.99 | U22 snoRNA host gene (UHG) gene, complete sequence, full insert sequence. |
| AK078855 | 9030025P20Rik | 0.99 | hypothetical protein, full insert sequence. |
| AK132581 | Pam | 0.99 | peptidylglycine alpha-amidating monooxygenase, full insert sequence. |
| AK158125 | Slc38a5 | 0.99 | Amino acid transporter system N2 homolog, full insert sequence. |
| AK165582 | Prune2 | 0.99 | Weakly similar to BCL2/adenovirus E1B 19-kDa protein-interacting protein 2, full insert sequence. |
| AK170700 | 5033414D02Rik | 0.99 | similar to AD025 (Hypothetical protein FLJ14688) [Homo sapiens], full insert sequence. |
| BC002243 | 4930455C21Rik | 0.99 | RIKEN cDNA 4930455C21 gene, mRNA (cDNA clone MGC:7537 IMAGE:3492342), complete cds. |
| BC009155 | Mgst1 | 0.99 | microsomal glutathione S-transferase 1, mRNA (cDNA clone MGC:6549 IMAGE:2655738), complete cds. |
| BC027505 | Magohb | 0.99 | RIKEN cDNA 2010012C16 gene, mRNA (cDNA clone MGC:41040 IMAGE:3372083), complete cds. |
| BC030177 | Endog | 0.99 | endonuclease G, mRNA (cDNA clone MGC:29098 IMAGE:5029633), complete cds. |
| BC036984 | Nnat | 0.99 | neuronatin, mRNA (cDNA clone MGC:46898 IMAGE:4981517), complete cds. |
| BC038898 | Trappc4 | 0.99 | trafficking protein particle complex 4, mRNA (cDNA clone MGC:49061 IMAGE:5401311), complete cds. |
| BC125311 | 1700045I19Rik | 0.99 | RIKEN cDNA 1700045I19 gene, mRNA (cDNA clone MGC:159014 IMAGE:40129826), complete cds. |
| BC132025 | Cbln4 | 0.99 | cerebellin 4 precursor protein, mRNA (cDNA clone MGC:163656 IMAGE:40130302), complete cds. |
| M22959 | Prlr | 0.99 | prolactin receptor (PRLR2) mRNA, complete cds. |
| NR_003640 | 1700045I19Rik | 0.99 | RIKEN cDNA 1700045I19 gene (1700045I19Rik) on chromosome X. |
| XM_001473759 | Gm2490 | 0.99 | 4933409K07Rik protein (LOC100039909), mRNA. |
| XM_001474410 | Gucy2d | 0.99 | guanylate cyclase 2d (Gucy2d), mRNA. |
| AF084459 | Capn1 | 0.99 | calpain I large subunit (Capn1) mRNA, complete cds. |
| AF454944 | Bbx | 0.99 | alt '5530401J07Rik#5730403O13Rik' |
| AK012133 | Myef2 | 0.99 | myelin basic protein expression factor 2, repressor, full insert sequence. |
| AK029042 | Capn12 | 0.99 | calpain 12, full insert sequence. |
| AK032763 | Hrh1 | 0.99 | HISTAMINE RECEPTOR H1, full insert sequence. |
| AK043819 | Srr | 0.99 | serine racemase, full insert sequence. |
| AK045873 | Dopey1 | 0.99 | hypothetical P-loop containing nucleotide triphosphate hydrolases structure containing protein, full insert sequence. |
| AK080000 | Il15ra | 0.99 | interleukin 15 receptor, alpha chain, full insert sequence. |
| AK083315 | Ctse | 0.99 | unclassifiable, full insert sequence. |
| AK083464 | Schip1 | 0.99 | schwannomin interacting protein 1, full insert sequence. |
| AK090202 | Rexo2 | 0.99 | SMALL FRAGMENT NUCLEASE homolog [Homo sapiens], full insert sequence. |
| AK129336 | Myef2 | 0.99 | mKIAA1341 protein. |
| AK143147 | Chat | 0.99 | choline acetyltransferase, full insert sequence. |
| AK144492 | Casz1 | 0.99 | unclassifiable, full insert sequence. |
| AK145601 | Pak6 | 0.99 | p21 (CDKN1A)-activated kinase 6, full insert sequence. |
| AK148053 | Synj2 | 0.99 | synaptojanin 2, full insert sequence. |
| AK151433 | Tcf4 | 0.99 | transcription factor 4, full insert sequence. |
| AK158612 | A830036E02Rik | 0.99 | unclassifiable, full insert sequence. |
| AK171048 | Agfg2 | 0.99 | NOD-derived CD11c +ve dendritic cells cDNA, RIKEN full-length enriched library, clone:F630221A09 product:HIV-1 Rev binding protein-like, full insert sequence. |
| AK172220 | Plekhm3 | 0.99 | spleen cDNA, RIKEN full-length enriched library, clone:F830107B19 product:Hypothetical pleckstrin homology homolog [Mus musculus], full insert sequence. |
| AK220217 | Tatdn2 | 0.99 | mKIAA0218 protein. |
| AY728090 | Cacna1c | 0.99 | neuronal voltage-gated calcium channel alpha 1C subunit (Cacna1c) mRNA, complete cds. |
| BC009653 | Npepps | 0.99 | aminopeptidase puromycin sensitive, mRNA (cDNA clone MGC:5986 IMAGE:3487224), complete cds. |
| BC011111 | Ssr3 | 0.99 | signal sequence receptor, gamma, mRNA (cDNA clone MGC:18930 IMAGE:3965897), complete cds. |
| BC014294 | Cd6 | 0.99 | CD6 antigen, mRNA (cDNA clone MGC:14018 IMAGE:4187689), complete cds. |
| BC019819 | N/A | 0.99 | cDNA clone IMAGE:5123675. |
| BC021331 | Tbrg1 | 0.99 | transforming growth factor beta regulated gene 1, mRNA (cDNA clone MGC:29296 IMAGE:5003215), complete cds. |
| BC024955 | Gdpd5 | 0.99 | glycerophosphodiester phosphodiesterase domain containing 5, mRNA (cDNA clone MGC:37148 IMAGE:4953174), complete cds. |
| BC032290 | Cc2d2a | 0.99 | coiled-coil and C2 domain containing 2A, mRNA (cDNA clone MGC:40866 IMAGE:5369681), complete cds. |
| BC054061 | Galnt9 | 0.99 | UDP-N-acetyl-alpha-D-galactosamine:polypeptide N-acetylgalactosaminyltransferase 9, mRNA (cDNA clone MGC:63144 IMAGE:5056396), complete cds. |
| BC055038 | Trim47 | 0.99 | tripartite motif protein 47, mRNA (cDNA clone IMAGE:6439977), partial cds. |
| BC055768 | Doc2a | 0.99 | double C2, alpha, mRNA (cDNA clone MGC:67326 IMAGE:6413208), complete cds. |
| BC057667 | Nlk | 0.99 | nemo like kinase, mRNA (cDNA clone MGC:68238 IMAGE:4235238), complete cds. |
| BC060694 | Pvrl1 | 0.99 | poliovirus receptor-related 1, mRNA (cDNA clone MGC:66906 IMAGE:6405088), complete cds. |
| BC064468 | 4930452B06Rik | 0.99 | RIKEN cDNA 4930452B06 gene, mRNA (cDNA clone MGC:69980 IMAGE:6333742), complete cds. |
| BC082585 | Gdpd5 | 0.99 | glycerophosphodiester phosphodiesterase domain containing 5, mRNA (cDNA clone MGC:100083 IMAGE:30608279), complete cds. |
| BC132041 | Fads6 | 0.98 | Fatty acid desaturase domain family, member 6, mRNA (cDNA clone MGC:163672 IMAGE:40130318), complete cds. |
| BC148308 | Dagla | 0.98 | Diacylglycerol lipase, alpha, mRNA (cDNA clone MGC:171133 IMAGE:40061146), complete cds. |
| BC148725 | Dgke | 0.98 | Diacylglycerol kinase, epsilon (Dgke) mRNA, encodes complete protein. |
| BC152876 | Rspo2 | 0.98 | R-spondin 2 homolog (Xenopus laevis) (Rspo2) mRNA, encodes complete protein. |
| NM_001080533.2 | UNC119B | 0.98 | unc-119 homolog B (C. elegans) |
| NM_024913.4 | CPED1 | 0.98 | cadherin-like and PC-esterase domain containing 1 |
| NM_001128.5 | AP1G1 | 0.98 | adaptor-related protein complex 1, gamma 1 subunit |
| NM_016202.2 | ZNF580 | 0.98 | zinc finger protein 580 |
| NM_001320595.1 | DDX5 | 0.98 | DEAD (Asp-Glu-Ala-Asp) box helicase 5 |
| NM_001042576.1 | RRBP1 | 0.98 | ribosome binding protein 1 |
| NM_003714.2 | STC2 | 0.98 | stanniocalcin 2 |
| NM_016422.3 | RNF141 | 0.98 | ring finger protein 141 |
| NM_019080.2 | NDFIP2 | 0.98 | Nedd4 family interacting protein 2 |
| NM_207385.1 | C16orf47 | 0.98 | chromosome 16 open reading frame 47 |
| NM_001267843.1 | CLOCK | 0.98 | clock circadian regulator |
| NM_194247.3 | HNRNPA3 | 0.98 | heterogeneous nuclear ribonucleoprotein A3 |
| NG_009630.1 | LRRC8A | 0.98 | leucine rich repeat containing 8 family, member A |
| NM_001002796.3 | MCTP1 | 0.98 | multiple C2 domains, transmembrane 1 |
| NM_177966.6 | PDE12 | 0.98 | phosphodiesterase 12 |
| BC156383 | Cep63 | 0.98 | centrosomal protein 63 (Cep63) mRNA, encodes complete protein. |
| NM_001099346 | Gm11937 | 0.98 | Krtap2-4-like (LOC100041488), mRNA. |
| NM_001099632 | Rnf39 | 0.98 | ring finger protein 39 (Rnf39), mRNA. |
| NM_207673 | Olfr100 | 0.98 | olfactory receptor 100 (Olfr100), mRNA. |
| NR_002839 | Dlx6as | 0.98 | distal-less homeobox 6, antisense (Dlx6as) on chromosome 6. |
| NR_003634 | Rps4y2 | 0.98 | ribosomal protein S4, Y-linked 2 (Rps4y2) on chromosome 6. |
| S78797 | Hyou1 | 0.98 | calcium binding protein/heat shock protein homolog |
| AB037111 | Asah2 | 0.98 | LCDase mRNA for neutral ceramidase, complete cds. |
| AB104633 | Isl1 | 0.98 | isl1-beta mRNA for transcription factor islet-1 isoform beta, complete cds. |
| AK003346 | Mobkl1a | 0.98 | weakly similar to CELL CYCLE ASSOCIATED PROTEIN MOB1-1 [Trypanosoma brucei], full insert sequence. |
| AK006748 | Prdx2 | 0.98 | peroxiredoxin 2, full insert sequence. |
| AK007015 | Hrasls5 | 0.98 | 1700086E08RIK PROTEIN (FRAGMENT) homolog [Mus musculus], full insert sequence. |
| AK007260 | 1700123O12Rik | 0.98 | unclassifiable, full insert sequence. |
| AK017085 | Gm11149 | 0.98 | neural cell adhesion molecule, full insert sequence. |
| AK019290 | Erlin1 | 0.98 | unclassifiable, full insert sequence. |
| AK038614 | Zfp583 | 0.98 | hypothetical Zn-finger, C2H2 type/KRAB box containing protein, full insert sequence. |
| AK039901 | Mll2 | 0.98 | unclassifiable, full insert sequence. |
| AK040020 | Osmr | 0.98 | oncostatin receptor, full insert sequence. |
| AK048056 | Dgcr8 | 0.98 | DGCRK6 PROTEIN homolog [Homo sapiens], full insert sequence. |
| AK053800 | N/A | 0.98 | RIKEN cDNA 2310079F23 gene, full insert sequence. |
| AK075736 | Lgals8 | 0.98 | lectin, galactose binding, soluble 8, full insert sequence. |
| AK076426 | Ednrb | 0.98 | ENDOTHELIN B RECEPTOR PRECURSOR, full insert sequence. |
| AK078065 | Sfrs5 | 0.98 | splicing factor, arginine/serine-rich 5 (SRp40, HRS), full insert sequence. |
| AK078282 | Gm3435 | 0.98 | similar to CDNA FLJ11152 FIS, CLONE PLACE1006901 (FRAGMENT) [Homo sapiens], full insert sequence. |
| AK080258 | N/A | 0.98 | unclassifiable, full insert sequence. |
| AK082264 | C230030N03Rik | 0.98 | hypothetical protein, full insert sequence. |
| AK082447 | Reln | 0.98 | reelin, full insert sequence. |
| AK082488 | Gtf2ird2 | 0.98 | GTF2IRD2 homolog [Mus musculus], full insert sequence. |
| AK084609 | Neo1 | 0.98 | neogenin, full insert sequence. |
| AK132365 | Brd8 | 0.98 | bromodomain containing 8, full insert sequence. |
| AK140748 | Meis1 | 0.98 | :myeloid ecotropic viral integration site 1, full insert sequence. |
| AK149867 | Med25 | 0.98 | TCBAP0758 protein (Fragment) homolog [Homo sapiens], full insert sequence. |
| AK154567 | Ppard | 0.98 | peroxisome proliferator activator receptor delta, full insert sequence. |
| AK156001 | Polq | 0.98 | polymerase (DNA directed), theta, full insert sequence. |
| AK164641 | Srp72 | 0.98 | similar to CaM kinase II isoform [Homo sapiens], full insert sequence. |
| AK170001 | Prune2 | 0.98 | Weakly similar to BCL2/adenovirus E1B 19-kDa protein-interacting protein 2, full insert sequence. |
| BC002214 | Echdc3 | 0.98 | enoyl Coenzyme A hydratase domain containing 3, mRNA (cDNA clone MGC:7470 IMAGE:3490443), complete cds. |
| BC003339 | Gpx3 | 0.98 | glutathione peroxidase 3, mRNA (cDNA clone MGC:6076 IMAGE:3257333), complete cds. |
| BC003491 | Hdhd3 | 0.98 | haloacid dehalogenase-like hydrolase domain containing 3, mRNA (cDNA clone MGC:6931 IMAGE:2811559), complete cds. |
| BC004680 | Cxadr | 0.98 | coxsackievirus and adenovirus receptor, mRNA (cDNA clone IMAGE:3500491), complete cds. |
| BC005705 | BC005705 | 0.98 | predicted gene, ENSMUSG00000042973, mRNA (cDNA clone MGC:11898 IMAGE:3598523), complete cds. |
| BC006800 | Trib1 | 0.98 | tribbles homolog 1 (Drosophila), mRNA (cDNA clone MGC:11752 IMAGE:3152842), complete cds. |
| BC009118 | Cbr4 | 0.98 | carbonyl reductase 4, mRNA (cDNA clone MGC:6971 IMAGE:3154595), complete cds. |
| BC011148 | Gjb5 | 0.98 | gap junction protein, beta 5, mRNA (cDNA clone MGC:18579 IMAGE:4207506), complete cds. |
| BC012518 | Tjp3 | 0.98 | tight junction protein 3, mRNA (cDNA clone MGC:11960 IMAGE:3600848), complete cds. |
| BC022170 | Slc27a2 | 0.98 | solute carrier family 27 (fatty acid transporter), member 2, mRNA (cDNA clone MGC:13834 IMAGE:4159081), complete cds. |
| BC025496 | Vat1 | 0.98 | vesicle amine transport protein 1 homolog (T californica), mRNA (cDNA clone MGC:38107 IMAGE:5320239), complete cds. |
| BC027309 | Lrrc61 | 0.98 | leucine rich repeat containing 61, mRNA (cDNA clone MGC:28139 IMAGE:3981816), complete cds. |
| BC030042 | Gm5861 | 0.98 | predicted gene, EG545728, mRNA (cDNA clone IMAGE:1329483), partial cds. |
| BC031758 | Mt2 | 0.98 | metallothionein 2, mRNA (cDNA clone MGC:19383 IMAGE:2651471), complete cds. |
| BC036560 | AI987944 | 0.98 | expressed sequence AI987944, mRNA (cDNA clone IMAGE:1446471), with apparent retained intron. |
| BC038612 | Slc12a3 | 0.98 | solute carrier family 12, member 3, mRNA (cDNA clone MGC:46751 IMAGE:4237274), complete cds. |
| BC043020 | Hba-a1 | 0.98 | hemoglobin alpha, adult chain 1, mRNA (cDNA clone MGC:57888 IMAGE:5684314), complete cds. |
| BC082583 | Rdh13 | 0.98 | retinol dehydrogenase 13 (all-trans and 9-cis), mRNA (cDNA clone MGC:105205 IMAGE:30613493), complete cds. |
| BC094889 | Chrnb4 | 0.98 | cholinergic receptor, nicotinic, beta polypeptide 4, mRNA (cDNA clone IMAGE:30653355), with apparent retained intron. |
| BC125281 | Lhx8 | 0.98 | LIM homeobox protein 8, mRNA (cDNA clone MGC:158984 IMAGE:40129796), complete cds. |
| BC131925 | Myl12b | 0.98 | myosin light chain, regulatory B, mRNA (cDNA clone MGC:163556 IMAGE:40130202), complete cds. |
| M16367 | Fcgr2b | 0.98 | Fc receptor (IgG receptor (beta-1)) mRNA, complete cds. |
| NM_001100116 | 1700047I17Rik2 | 0.98 | RIKEN cDNA 1700047I17 gene 2 (1700047I17Rik2), mRNA. |
| NR_002928 | Gm1943 | 0.98 | gene model 1943, (NCBI) (Gm1943) on chromosome 8. |
| AF081568 | Tubd1 | 0.98 | delta-tubulin mRNA, complete cds. |
| AF206720 | Apbb1 | 0.98 | Fe65 mRNA, complete cds. |
| AF398884 | Elmo2 | 0.98 | ELMO2 mRNA, complete cds. |
| AK011662 | Med25 | 0.98 | hypothetical protein, full insert sequence. |
| AK021163 | Dzip1 | 0.98 | RIKEN cDNA 2810422M04 gene, full insert sequence. |
| AK028783 | Ptpn14 | 0.98 | protein tyrosine phosphatase, non-receptor type 14, full insert sequence. |
| AK032504 | 6430573F11Rik | 0.98 | unclassifiable, full insert sequence. |
| AK032654 | C230057M02Rik | 0.98 | unclassifiable, full insert sequence. |
| AK036208 | Rfx1 | 0.98 | regulatory factor (trans-acting) 1, full insert sequence. |
| AK039254 | Mapk8 | 0.98 | unclassifiable, full insert sequence. |
| AK043659 | A830009L08Rik | 0.98 | unclassifiable, full insert sequence. |
| AK047396 | Sec14l1 | 0.98 | SEC14-LIKE PROTEIN 1 homolog [Homo sapiens], full insert sequence. |
| AK049668 | Zfp33b | 0.98 | unclassifiable, full insert sequence. |
| AK084198 | Ptpn13 | 0.98 | protein tyrosine phosphatase, non-receptor type 13, full insert sequence. |
| AK138412 | N28178 | 0.98 | hypothetical protein, full insert sequence. |
| AK147689 | Asap1 | 0.98 | development and differentiation enhancing, full insert sequence. |
| AK162386 | 9330154J02Rik | 0.98 | Hypothetical proline-rich region containing protein (Fragment) homolog [Mus musculus], full insert sequence. |
| BC003766 | Nfix | 0.98 | nuclear factor I/X, mRNA (cDNA clone MGC:5944 IMAGE:3491917), complete cds. |
| BC012668 | Vamp8 | 0.98 | vesicle-associated membrane protein 8, mRNA (cDNA clone MGC:13842 IMAGE:4205509), complete cds. |
| BC026146 | Pik3r1 | 0.98 | phosphatidylinositol 3-kinase, regulatory subunit, polypeptide 1 (p85 alpha), mRNA (cDNA clone MGC:13952 IMAGE:3979333), complete cds. |
| BC034855 | St8sia5 | 0.98 | ST8 alpha-N-acetyl-neuraminide alpha-2,8-sialyltransferase 5, mRNA (cDNA clone MGC:41119 IMAGE:5146703), complete cds. |
| BC038362 | Napb | 0.98 | N-ethylmaleimide sensitive fusion protein attachment protein beta, mRNA (cDNA clone MGC:36510 IMAGE:5368175), complete cds. |
| BC044208 | H2-T9 | 0.98 | histocompatibility 2, T region locus 9, mRNA (cDNA clone MGC:49483 IMAGE:5036676), complete cds. |
| BC050107 | Hyou1 | 0.98 | hypoxia up-regulated 1, mRNA (cDNA clone MGC:60469 IMAGE:30012694), complete cds. |
| BC050781 | Btbd10 | 0.98 | BTB (POZ) domain containing 10, mRNA (cDNA clone MGC:58464 IMAGE:6591397), complete cds. |
| BC052371 | 6530418L21Rik | 0.98 | RIKEN cDNA 6530418L21 gene, mRNA (cDNA clone MGC:58959 IMAGE:6491043), complete cds. |
| BC057309 | Hjurp | 0.98 | RIKEN cDNA 6430706D22 gene, mRNA (cDNA clone MGC:66590 IMAGE:6826508), complete cds. |
| BC067001 | C1qb | 0.98 | complement component 1, q subcomponent, beta polypeptide, mRNA (cDNA clone MGC:90036 IMAGE:5715633), complete cds. |
| BC068251 | 1110021L09Rik | 0.98 | RIKEN cDNA 1110021L09 gene, mRNA (cDNA clone IMAGE:6430662), **** WARNING: chimeric clone ****. |
| AK015672 | Csmd3 | 0.97 | Hypothetical Sushi domain / SCR repeat / CCP module containing protein, full insert sequence. |
| BC148635 | H2-Q6 | 0.97 | Histocompatibility 2, Q region locus 6 (H2-Q6) mRNA, encodes complete protein. |
| BC156068 | Tcfap2e | 0.97 | Transcription factor AP-2, epsilon (Tcfap2e) mRNA, encodes complete protein. |
| NM_014232.2 | VAMP2 | 0.97 | vesicle-associated membrane protein 2 (synaptobrevin 2) |
| NM_020379.3 | MAN1C1 | 0.97 | mannosidase, alpha, class 1C, member 1 |
| NR_033361.1 | TMCC1 | 0.97 | transmembrane and coiled-coil domain family 1 |
| NG_046782.1 | HACE1 | 0.97 | HECT domain and ankyrin repeat containing E3 ubiquitin protein ligase 1 |
| NM_001031562.1 | HIAT1 | 0.97 | hippocampus abundant transcript 1 |
| NM_032177.3 | PHAX | 0.97 | phosphorylated adaptor for RNA export |
| NM_006924.4 | SRSF1 | 0.97 | serine/arginine-rich splicing factor 1 |
| NG_027815.1 | TKT | 0.97 | transketolase |
| NM_001110807 | Capn12 | 0.97 | calpain 12 (Capn12), mRNA. |
| NM_017385 | Ear7 | 0.97 | eosinophil-associated, ribonuclease A family, member 7 (Ear7), mRNA. |
| NM_177052 | Kif6 | 0.97 | kinesin family member 6 (Kif6), mRNA. |
| AK012683 | Pmm2 | 0.97 | phosphomannomutase 2, full insert sequence. |
| AK079713 | N/A | 0.97 | unclassifiable, full insert sequence. |
| AK081942 | Sncaip | 0.97 | synuclein, alpha interacting protein (synphilin), full insert sequence. |
| AK085745 | Mapk8 | 0.97 | mitogen activated protein kinase 8, full insert sequence. |
| AK153514 | Irf1 | 0.97 | interferon regulatory factor 1, full insert sequence. |
| AK158158 | Nlrp12 | 0.97 | NACHT, LRR and PYD containing protein 12, full insert sequence. |
| BC016222 | 544988 | 0.97 | cDNA clone IMAGE:4486605. |
| BC021835 | Nfatc3 | 0.97 | nuclear factor of activated T-cells, cytoplasmic, calcineurin-dependent 3, mRNA (cDNA clone IMAGE:4015209), containing frame-shift errors. |
| BC055059 | Hoxa9 | 0.97 | homeo box A9, mRNA (cDNA clone MGC:62545 IMAGE:6529502), complete cds. |
| BC055730 | Cbln1 | 0.97 | cerebellin 1 precursor protein, mRNA (cDNA clone MGC:66947 IMAGE:6404555), complete cds. |
| BC060740 | Ttyh2 | 0.97 | tweety homolog 2 (Drosophila), mRNA (cDNA clone MGC:67934 IMAGE:4158813), complete cds. |
| BC065787 | Crabp1 | 0.97 | cellular retinoic acid binding protein I, mRNA (cDNA clone MGC:73634 IMAGE:891192), complete cds. |
| BC080693 | Zfp619 | 0.97 | zinc finger protein 619, mRNA (cDNA clone MGC:91101 IMAGE:30432277), complete cds. |
| BC082788 | Col4a5 | 0.97 | collagen, type IV, alpha 5, mRNA (cDNA clone MGC:91198 IMAGE:5343488), complete cds. |
| BC120894 | Cacng5 | 0.97 | calcium channel, voltage-dependent, gamma subunit 5, mRNA (cDNA clone MGC:141423 IMAGE:40061528), complete cds. |
| BC132629 | Tmprss11f | 0.97 | transmembrane protease, serine 11f, mRNA (cDNA clone MGC:164260 IMAGE:40130906), complete cds. |
| BC132645 | Ddit4 | 0.97 | DNA-damage-inducible transcript 4, mRNA (cDNA clone MGC:164276 IMAGE:40130922), complete cds. |
| BC152885 | Hist4h4 | 0.97 | histone cluster 4, H4 (Hist4h4) mRNA, encodes complete protein. |
| AK015404 | 4930447F24Rik | 0.97 | unclassifiable, full insert sequence. |
| AK030275 | 1700020I14Rik | 0.97 | RIKEN cDNA 1700020I14 gene, full insert sequence. |
| AK047522 | Adc | 0.97 | ORNITHINE DECARBOXYLASE-LIKE PROTEIN VARIANT 1 (SIMILAR TO ORNITHINE DECARBOXYLASE 1) homolog [Homo sapiens], full insert sequence. |
| AK048454 | Dars2 | 0.97 | ASPARTYL-TRNA SYNTHETASE (EC 6.1.1.12) (ASPARTATE--TRNA LIGASE) (ASPRS) [Bacillus subtilis], full insert sequence. |
| AK053169 | Clp1 | 0.97 | hypothetical protein, full insert sequence. |
| AK136877 | N/A | 0.97 | unclassifiable, full insert sequence. |
| AK141213 | Cd6 | 0.97 | CD6 antigen, full insert sequence. |
| AK170678 | H2-D1 | 0.97 | NOD-derived CD11c +ve dendritic cells cDNA, RIKEN full-length enriched library, clone:F630113J05 product:histocompatibility 2, D region locus 1, full insert sequence. |
| AK171658 | Csrnp1 | 0.97 | activated spleen cDNA, RIKEN full-length enriched library, clone:F830002L07 product:AXIN1 up-regulated 1, full insert sequence. |
| BC004079 | Mpst | 0.97 | mercaptopyruvate sulfurtransferase, mRNA (cDNA clone MGC:8271 IMAGE:3592659), complete cds. |
| BC005443 | Fbln2 | 0.97 | fibulin 2, mRNA (cDNA clone MGC:6112 IMAGE:3490759), complete cds. |
| BC046957 | Cd22 | 0.97 | CD22 antigen, mRNA (cDNA clone IMAGE:6314073), with apparent retained intron. |
| BC050125 | Adcy1 | 0.97 | adenylate cyclase 1, mRNA (cDNA clone IMAGE:30015692). |
| BC051986 | Ier5 | 0.97 | immediate early response 5, mRNA (cDNA clone MGC:62292 IMAGE:5707524), complete cds. |
| BC060373 | BC027072 | 0.97 | cDNA sequence BC027072, mRNA (cDNA clone MGC:65563 IMAGE:6494066), complete cds. |
| BC066052 | Hdac4 | 0.97 | histone deacetylase 4, mRNA (cDNA clone MGC:91339 IMAGE:6827645), complete cds. |
| BC067048 | Tiam2 | 0.97 | T-cell lymphoma invasion and metastasis 2, mRNA (cDNA clone MGC:91320 IMAGE:30535152), complete cds. |
| BC082561 | Glt8d2 | 0.97 | glycosyltransferase 8 domain containing 2, mRNA (cDNA clone MGC:100315 IMAGE:30603848), complete cds. |
| BC092225 | Scn2b | 0.97 | sodium channel, voltage-gated, type II, beta, mRNA (cDNA clone MGC:116602 IMAGE:6839081), complete cds. |
| BC094607 | Sel1l3 | 0.97 | RIKEN cDNA 2310045A20 gene, mRNA (cDNA clone MGC:106638 IMAGE:6837048), complete cds. |
| AK031221 | Uvrag | 0.96 | Unclassifiable, full insert sequence. |
| BC148419 | Adrbk2 | 0.96 | Adrenergic receptor kinase, beta 2 (Adrbk2) mRNA, encodes complete protein. |
| NG_029614.1 | PDE7A | 0.96 | phosphodiesterase 7A |
| NM_001136157.1 | OTUD5 | 0.96 | OTU domain containing 5 |
| NM_001659.2 | ARF3 | 0.96 | ADP-ribosylation factor 3 |
| NG_029731.1 | CLCN3 | 0.96 | chloride channel, voltage-sensitive 3 |
| NM_006401.2 | ANP32B | 0.96 | acidic (leucine-rich) nuclear phosphoprotein 32 family, member B |
| BC156198 | Kcnu1 | 0.96 | potassium channel, subfamily U, member 1 (Kcnu1) mRNA, encodes complete protein. |
| DQ157177 | Pttg1 | 0.96 | PTTG3 (Pttg3) mRNA, complete cds. |
| DQ314497 | Lrfn1 | 0.96 | synaptic differentiation enhancing molecule 1 (Semo1) mRNA, complete cds. |
| AK004870 | 1300003B13Rik | 0.96 | similar to MSZF13 (FRAGMENT) [Mus musculus], full insert sequence. |
| AK012646 | Grb10 | 0.96 | growth factor receptor bound protein 10, full insert sequence. |
| AK038783 | Amigo2 | 0.96 | A230062J08 product:hypothetical Leucine-rich repeat, outliers/Leucine-rich repeat/Immunoglobulin and major histocompatibility complex domain/Immunoglobulin-like/Immunoglobulin subtype/Leucine-rich repeat, typical subtype containing protein, full insert sequence. |
| AK038859 | Gabrq | 0.96 | gamma-aminobutyric acid (GABA-A) receptor, subunit theta, full insert sequence. |
| AK049101 | H13 | 0.96 | histocompatibility 13, full insert sequence. |
| AK050809 | N/A | 0.96 | BICAUDAL D (DROSOPHILA) HOMOLOG 1 homolog [Homo sapiens], full insert sequence. |
| AK083547 | Prox1 | 0.96 | unclassifiable, full insert sequence. |
| AK083596 | Ambra1 | 0.96 | ISCHEMIA RELATED FACTOR NYW-1 homolog [Rattus norvegicus], full insert sequence. |
| AK141257 | Nubpl | 0.96 | weakly similar to Putative nucleotide-binding protein [Oryza sativa], full insert sequence. |
| AK143562 | Lbp | 0.96 | lipopolysaccharide binding protein, full insert sequence. |
| AK148670 | Sparc | 0.96 | secreted acidic cysteine rich glycoprotein, full insert sequence. |
| AK155011 | 2010106G01Rik | 0.96 | Signal peptide peptidase-like 2A (EC 3.4.99.-) (SPP-like 2A protein) (SPPL2a protein) (Intramembrane protease 3) (IMP3) (Presenilin-like protein 2), full insert sequence. |
| AK155538 | Zfp395 | 0.96 | Si-1-8-16 protein (Fragment) homolog [Homo sapiens], full insert sequence. |
| AK162249 | Nuf2 | 0.96 | cell division cycle associated 1, full insert sequence. |
| AK163167 | Stx6 | 0.96 | syntaxin 6, full insert sequence. |
| BC003305 | Lpl | 0.96 | lipoprotein lipase, mRNA (cDNA clone MGC:5666 IMAGE:3584788), complete cds. |
| BC003482 | Tspan4 | 0.96 | tetraspanin 4, mRNA (cDNA clone MGC:6308 IMAGE:2811065), complete cds. |
| BC012675 | Clk4 | 0.96 | CDC like kinase 4, mRNA (cDNA clone MGC:13871 IMAGE:3982920), complete cds. |
| BC012871 | Phactr2 | 0.96 | phosphatase and actin regulator 2, mRNA (cDNA clone IMAGE:3482264), partial cds. |
| BC018267 | Rit2 | 0.96 | Ras-like without CAAX 2, mRNA (cDNA clone MGC:25308 IMAGE:4511264), complete cds. |
| BC021376 | Zfp521 | 0.96 | zinc finger protein 521, mRNA (cDNA clone MGC:29358 IMAGE:5038671), complete cds. |
| BC021453 | Ddx3y | 0.96 | DEAD (Asp-Glu-Ala-Asp) box polypeptide 3, Y-linked, mRNA (cDNA clone MGC:29067 IMAGE:5043470), complete cds. |
| BC023365 | Gdpd2 | 0.96 | glycerophosphodiester phosphodiesterase domain containing 2, mRNA (cDNA clone IMAGE:5039062), with apparent retained intron. |
| BC025502 | Arhgap24 | 0.96 | Rho GTPase activating protein 24, mRNA (cDNA clone MGC:38175 IMAGE:5321985), complete cds. |
| BC026752 | Vta1 | 0.96 | Vps20-associated 1 homolog (S. cerevisiae), mRNA (cDNA clone MGC:25603 IMAGE:4034998), complete cds. |
| BC027226 | Lman2l | 0.96 | lectin, mannose-binding 2-like, mRNA (cDNA clone MGC:27758 IMAGE:2654285), complete cds. |
| BC034187 | BC035947 | 0.96 | cDNA sequence BC035947, mRNA (cDNA clone MGC:37308 IMAGE:4975064), complete cds. |
| BC039141 | C4b | 0.96 | complement component 4B (Childo blood group), mRNA (cDNA clone IMAGE:3660898), containing frame-shift errors. |
| BC039804 | Wdsub1 | 0.96 | cDNA clone IMAGE:1495768. |
| BC039805 | Tmem53 | 0.96 | transmembrane protein 53, mRNA (cDNA clone IMAGE:1511070). |
| BC043069 | Hnrnpab | 0.96 | heterogeneous nuclear ribonucleoprotein A/B, mRNA (cDNA clone MGC:57973 IMAGE:6402930), complete cds. |
| BC044745 | N/A | 0.96 | cDNA clone IMAGE:5149239, partial cds. |
| BC050804 | Whamm | 0.96 | WAS protein homology region 2 domain containing 1, mRNA (cDNA clone MGC:58752 IMAGE:6771347), complete cds. |
| BC050816 | Lrrc27 | 0.96 | leucine rich repeat containing 27, mRNA (cDNA clone MGC:58860 IMAGE:6774506), complete cds. |
| BC050889 | Zic1 | 0.96 | zinc finger protein of the cerebellum 1, mRNA (cDNA clone MGC:63273 IMAGE:6418777), complete cds. |
| BC051924 | Gstm7 | 0.96 | glutathione S-transferase, mu 7, mRNA (cDNA clone MGC:62165 IMAGE:5692787), complete cds. |
| BC054763 | Magel2 | 0.96 | melanoma antigen, family L, 2, mRNA (cDNA clone IMAGE:6414320), complete cds. |
| BC057853 | Abca4 | 0.96 | ATP-binding cassette, sub-family A (ABC1), member 4, mRNA (cDNA clone MGC:62660 IMAGE:6492139), complete cds. |
| BC058595 | Gjd2 | 0.96 | gap junction protein, delta 2, mRNA (cDNA clone MGC:68336 IMAGE:5364942), complete cds. |
| BC060600 | Pla2g4e | 0.96 | phospholipase A2, group IVE, mRNA (cDNA clone IMAGE:5705456). |
| BC064060 | Gjb4 | 0.96 | gap junction protein, beta 4, mRNA (cDNA clone MGC:73616 IMAGE:1498230), complete cds. |
| BC065089 | Slc5a7 | 0.96 | solute carrier family 5 (choline transporter), member 7, mRNA (cDNA clone MGC:86023 IMAGE:6851099), complete cds. |
| BC094319 | Eomes | 0.96 | eomesodermin homolog (Xenopus laevis), mRNA (cDNA clone MGC:106424 IMAGE:5721456), complete cds. |
| BC094322 | Srrm1 | 0.96 | serine/arginine repetitive matrix 1, mRNA (cDNA clone MGC:106668 IMAGE:6401250), complete cds. |
| BC108402 | Rbms3 | 0.96 | RNA binding motif, single stranded interacting protein, mRNA (cDNA clone MGC:118437 IMAGE:6516420), complete cds. |
| U79523 | Pam | 0.96 | peptidylglycine alpha-amidating monooxygenase (PAM) mRNA, complete cds. |
| XM_001474794 | Gm12349 | 0.96 | hypothetical protein LOC100040491 (LOC100040491), mRNA. |
| AF169191 | Numb | 0.96 | 71 kDa Numb protein isoform mRNA, complete cds. |
| AF220039 | Trim9 | 0.96 | tripartite motif protein TRIM9 (Trim9) mRNA, partial cds. |
| AK021127 | C030037D09Rik | 0.96 | unclassifiable, full insert sequence. |
| AK028498 | Phf6 | 0.96 | hypothetical PHD-finger/Cytochrome c family heme-binding site containing protein, full insert sequence. |
| AK037867 | 6030458C11Rik | 0.96 | hypothetical protein, full insert sequence. |
| AK047175 | Kit | 0.96 | unclassifiable, full insert sequence. |
| AK081235 | Unc80 | 0.96 | CG18437 PROTEIN [Drosophila melanogaster], full insert sequence. |
| AK163141 | Ptprn | 0.96 | protein tyrosine phosphatase, receptor type, N, full insert sequence. |
| AK163512 | Synj2 | 0.96 | synaptojanin 2, full insert sequence. |
| AK166436 | Kcnma1 | 0.96 | potassium large conductance calcium-activated channel, subfamily M, alpha member 1, full insert sequence. |
| AY968047 | Dclk1 | 0.96 | CLICK-I mRNA, complete cds. |
| BC002051 | Khdrbs1 | 0.96 | KH domain containing, RNA binding, signal transduction associated 1, mRNA (cDNA clone MGC:6063 IMAGE:3490667), complete cds. |
| BC006771 | Kpna1 | 0.96 | karyopherin (importin) alpha 1, mRNA (cDNA clone MGC:6726 IMAGE:3588534), complete cds. |
| BC006863 | Faah | 0.96 | fatty acid amide hydrolase, mRNA (cDNA clone MGC:11634 IMAGE:3595191), complete cds. |
| BC010968 | Insl5 | 0.96 | insulin-like 5, mRNA (cDNA clone MGC:13722 IMAGE:4217383), complete cds. |
| BC031988 | Zfp87 | 0.96 | zinc finger protein 87, mRNA (cDNA clone MGC:25780 IMAGE:4017149), complete cds. |
| BC049180 | Vps26b | 0.96 | vacuolar protein sorting 26 homolog B (yeast), mRNA (cDNA clone MGC:54784 IMAGE:6314347), complete cds. |
| BC051917 | Mmp17 | 0.96 | matrix metallopeptidase 17, mRNA (cDNA clone MGC:62154 IMAGE:5716458), complete cds. |
| BC052526 | Acot8 | 0.96 | acyl-CoA thioesterase 8, mRNA (cDNA clone IMAGE:3472350). |
| BC055342 | Fam185a | 0.96 | expressed sequence AI847670, mRNA (cDNA clone MGC:65666 IMAGE:6810783), complete cds. |
| BC060243 | Tmem25 | 0.96 | transmembrane protein 25, mRNA (cDNA clone MGC:66738 IMAGE:5696102), complete cds. |
| BC062968 | Btbd3 | 0.96 | BTB (POZ) domain containing 3, mRNA (cDNA clone MGC:76394 IMAGE:6408511), complete cds. |
| BC080288 | Scarf1 | 0.96 | scavenger receptor class F, member 1, mRNA (cDNA clone MGC:90792 IMAGE:6411836), complete cds. |
| AK014823 | Rbm4b | 0.95 | RNA-BINDING PROTEIN LARK homolog, full insert sequence. |
| AK031163 | Col16a1 | 0.95 | Procollagen, type XVI, alpha 1, full insert sequence. |
| NM_152441.2 | FBXL14 | 0.95 | F-box and leucine-rich repeat protein 14 |
| NM_001135700.1 | YWHAZ | 0.95 | tyrosine 3-monooxygenase/tryptophan 5-monooxygenase activation protein, zeta polypeptide |
| NG_032112.1 | TPK1 | 0.95 | thiamin pyrophosphokinase 1 |
| NG_012846.1 | MATR3 | 0.95 | matrin 3 |
| NM_181746.3 | CERS2 | 0.95 | ceramide synthase 2 |
| DQ463750 | Dst | 0.95 | BPAG1 isoform 3 mRNA, partial cds. |
| NM_026834 | Krtap4-6 | 0.95 | RIKEN cDNA 1110054P19 gene (1110054P19Rik), mRNA. |
| AK006973 | 1700081N11Rik | 0.95 | unclassifiable, full insert sequence. |
| AK054190 | Prune2 | 0.95 | weakly similar to BCL2/ADENOVIRUS E1B 19-KDA PROTEIN-INTERACTING PROTEIN 2 [Homo sapiens], full insert sequence. |
| AK083200 | 1500004A13Rik | 0.95 | unclassifiable, full insert sequence. |
| AK159332 | Tgfbrap1 | 0.95 | Hypothetical prenyl group binding site, full insert sequence. |
| BC006937 | Ociad1 | 0.95 | OCIA domain containing 1, mRNA (cDNA clone MGC:6810 IMAGE:2648369), complete cds. |
| BC012697 | Ucp2 | 0.95 | uncoupling protein 2 (mitochondrial, proton carrier), mRNA (cDNA clone MGC:13955 IMAGE:4205625), complete cds. |
| BC023300 | Zmynd8 | 0.95 | protein kinase C binding protein 1, mRNA (cDNA clone IMAGE:4194817), complete cds. |
| BC025898 | Polr3k | 0.95 | polymerase (RNA) III (DNA directed) polypeptide K, mRNA (cDNA clone MGC:30983 IMAGE:5253879), complete cds. |
| BC034166 | Rasd1 | 0.95 | RAS, dexamethasone-induced 1, mRNA (cDNA clone MGC:36188 IMAGE:4989312), complete cds. |
| BC036126 | Gpc3 | 0.95 | glypican 3, mRNA (cDNA clone MGC:35964 IMAGE:4973409), complete cds. |
| BC039156 | Hcn3 | 0.95 | hyperpolarization-activated, cyclic nucleotide-gated K+ 3, mRNA (cDNA clone MGC:25272 IMAGE:4511289), complete cds. |
| BC039569 | Accs | 0.95 | RIKEN cDNA 2610203E10 gene, mRNA (cDNA clone MGC:48140 IMAGE:1449080), complete cds. |
| BC044055 | Gal | 0.95 | galanin, mRNA (cDNA clone MGC:54666 IMAGE:6476459), complete cds. |
| BC054764 | Ppp1r16b | 0.95 | protein phosphatase 1, regulatory (inhibitor) subunit 16B, mRNA (cDNA clone MGC:64754 IMAGE:6414610), complete cds. |
| BC055403 | Zbtb11 | 0.95 | zinc finger and BTB domain containing 11, mRNA (cDNA clone IMAGE:6485438). |
| BC058238 | Mobkl2a | 0.95 | MOB1, Mps One Binder kinase activator-like 2A (yeast), mRNA (cDNA clone MGC:65537 IMAGE:6307543), complete cds. |
| BC058615 | Igf2 | 0.95 | insulin-like growth factor 2, mRNA (cDNA clone MGC:64763 IMAGE:5686066), complete cds. |
| BC058626 | 2310022B05Rik | 0.95 | RIKEN cDNA 2310022B05 gene, mRNA (cDNA clone MGC:73467 IMAGE:6401577), complete cds. |
| BC109368 | Msi2 | 0.95 | Musashi homolog 2 (Drosophila), mRNA (cDNA clone MGC:118040 IMAGE:5249862), complete cds. |
| BC110468 | Olfr1370 | 0.95 | olfactory receptor 1370, mRNA (cDNA clone MGC:123746 IMAGE:40043199), complete cds. |
| BC113756 | Mela | 0.95 | melanoma antigen, mRNA (cDNA clone MGC:130515 IMAGE:40047695), complete cds. |
| BC116905 | Npvf | 0.95 | neuropeptide VF precursor, mRNA (cDNA clone MGC:151282 IMAGE:40126224), complete cds. |
| BC116911 | Prok2 | 0.95 | prokineticin 2, mRNA (cDNA clone MGC:151288 IMAGE:40126230), complete cds. |
| EF651819 | Gm8050 | 0.95 | alpha22-takusan mRNA, complete cds. |
| EF651833 | LOC100038847 | 0.95 | alpha36-takusan mRNA, complete cds. |
| EF651841 | Gm3642 | 0.95 | alpha44-takusan mRNA, complete cds. |
| NM_001085417 | Zfp467 | 0.95 | zinc finger protein 467 (Zfp467), transcript variant 4, mRNA. |
| NM_001100454 | Wfikkn1 | 0.95 | WAP, FS, Ig, KU, and NTR-containing protein 1 (Wfikkn1), mRNA. |
| NM_001102446 | Alas2 | 0.95 | aminolevulinic acid synthase 2, erythroid (Alas2), transcript variant 2, mRNA. |
| AF053955 | Runx2 | 0.95 | Cbfa1/Osf2 transcription factor isoform-2 mRNA, partial sequence. |
| AF079108 | Gfra2 | 0.95 | glial cell line derived neurotrophic factor family receptor alpha 2c (Gfra2) mRNA, complete cds. |
| AJ278430 | Acox3 | 0.95 | pristanoyl-CoA oxidase (Acox3 gene). |
| AK010587 | 2410024N18Rik | 0.95 | DM417G6.2.2 (NOVEL PROTEIN (ISOFORM 2)) homolog |
| AK012787 | Yaf2 | 0.95 | YY1 associated factor 2, full insert sequence. |
| AK029800 | Nol4 | 0.95 | NOLP PROTEIN (HRIHFB2255 PROTEIN) [Homo sapiens], full insert sequence. |
| AK031757 | Sept9 | 0.95 | septin 9, full insert sequence. |
| AK045624 | 5330416C01Rik | 0.95 | unclassifiable, full insert sequence. |
| AK075657 | Myo5c | 0.95 | MYOSIN 5C homolog [Mus musculus], full insert sequence. |
| AK083260 | Kidins220 | 0.95 | KIDINS220 homolog [Rattus norvegicus], full insert sequence. |
| AK090150 | N/A | 0.95 | unclassifiable, full insert sequence. |
| AK129364 | Pitpnm2 | 0.95 | mKIAA1457 protein. |
| AK142919 | Synj2 | 0.95 | synaptojanin 2, full insert sequence. |
| AK170381 | Galnt9 | 0.95 | UDP-N-acetyl-alpha-D-galactosamine:polypeptide N-acetylgalactosaminyltransferase 9, full insert sequence. |
| BC005550 | Lum | 0.95 | lumican, mRNA (cDNA clone MGC:5828 IMAGE:3585672), complete cds. |
| BC016235 | Hkdc1 | 0.95 | hexokinase domain containing 1, mRNA (cDNA clone MGC:28816 IMAGE:4504302), complete cds. |
| BC039571 | C2cd4b | 0.95 | RIKEN cDNA 3300001A09 gene, mRNA (cDNA clone MGC:48200 IMAGE:1515262), complete cds. |
| BC050909 | Zfp238 | 0.95 | zinc finger protein 238, clone IMAGE:6417039, mRNA, partial cds. |
| BC052011 | Foxc1 | 0.95 | forkhead box C1, mRNA (cDNA clone MGC:62340 IMAGE:5720391), complete cds. |
| BC052317 | Rtn4r | 0.95 | reticulon 4 receptor, mRNA (cDNA clone MGC:54506 IMAGE:6397765), complete cds. |
| BC068307 | Loxl4 | 0.95 | lysyl oxidase-like 4, mRNA (cDNA clone MGC:76593 IMAGE:30059745), complete cds. |
| BC079863 | Srd5a1 | 0.95 | steroid 5 alpha-reductase 1, mRNA (cDNA clone MGC:100251 IMAGE:6807475), complete cds. |
| AK010245 | 2310081O03Rik | 0.94 | Unclassifiable, full insert sequence. |
| BC130262 | Gatad2b | 0.94 | MuGATA zinc finger domain containing 2B, mRNA (cDNA clone MGC:161344 IMAGE:40142858), complete cds. |
| BC152841 | Hspa1b | 0.94 | Heat shock protein 1B (Hspa1b) mRNA, encodes complete protein. |
| NM_024015.4 | HOXB4 | 0.94 | homeobox B4 |
| NM_001010875.3 | SLC25A30 | 0.94 | solute carrier family 25, member 30 |
| NG_017066.1 | SLC29A3 | 0.94 | solute carrier family 29 (equilibrative nucleoside transporter), member 3 |
| NG_008915.1 | HSPD1 | 0.94 | heat shock 60kDa protein 1 (chaperonin) |
| NG_023407.1 | SLC25A22 | 0.94 | solute carrier family 25 (mitochondrial carrier: glutamate), member 22 |
| NG_050652.1 | NET1 | 0.94 | neuroepithelial cell transforming 1 |
| NM_001044305.2 | SMAP1 | 0.94 | small ArfGAP 1 |
| NG_012071.1 | GCLC | 0.94 | glutamate-cysteine ligase, catalytic subunit |
| BC156500 | Col6a1 | 0.94 | collagen, type VI, alpha 1 (Col6a1) mRNA, encodes complete protein. |
| DQ284430 | 1700003O11Rik | 0.94 | spermatocyte-specific marker protein mRNA, complete cds. |
| XM_001474920 | Gm9352 | 0.94 | similar to Retinoblastoma binding protein 6 (LOC668776), mRNA. |
| AK006011 | Speer4c | 0.94 | 4933431D05RIK PROTEIN (FRAGMENT) homolog [Mus musculus], full insert sequence. |
| AK028940 | Papola | 0.94 | POLY(A) POLYMERASE ALPHA (EC 2.7.7.19) (PAP) (POLYNUCLEOTIDE ADENYLYLTRANSFERASE ALPHA) homolog [Bos taurus], full insert sequence. |
| AK043731 | Rsrc1 | 0.94 | BM-011 homolog [Homo sapiens], full insert sequence. |
| BC002074 | Ass1 | 0.94 | argininosuccinate synthetase 1, mRNA (cDNA clone MGC:6218 IMAGE:3491910), complete cds. |
| BC039220 | Hnf4a | 0.94 | hepatic nuclear factor 4, alpha, mRNA (cDNA clone MGC:31368 IMAGE:4238842), complete cds. |
| BC080781 | Suclg2 | 0.94 | succinate-Coenzyme A ligase, GDP-forming, beta subunit, mRNA (cDNA clone MGC:91183 IMAGE:30470296), complete cds. |
| BC106124 | Sfi1 | 0.94 | Sfi1 homolog, spindle assembly associated (yeast), mRNA (cDNA clone IMAGE:3675856), complete cds. |
| BC107277 | Arpm1 | 0.94 | actin related protein M1, mRNA (cDNA clone MGC:130225 IMAGE:40052700), complete cds. |
| BC110376 | Cbx3 | 0.94 | chromobox homolog 3 (Drosophila HP1 gamma), mRNA (cDNA clone MGC:107015 IMAGE:3467208), complete cds. |
| BC115833 | Adcy7 | 0.94 | adenylate cyclase 7, mRNA (cDNA clone MGC:141539 IMAGE:40086640), complete cds. |
| BC116901 | Speer4f | 0.94 | spermatogenesis associated glutamate (E)-rich protein 4f, mRNA (cDNA clone MGC:151278 IMAGE:40126220), complete cds. |
| BC116957 | Mc4r | 0.94 | melanocortin 4 receptor, mRNA (cDNA clone MGC:151334 IMAGE:40126276), complete cds. |
| BC116986 | Htr7 | 0.94 | 5-hydroxytryptamine (serotonin) receptor 7, mRNA (cDNA clone MGC:151363 IMAGE:40126305), complete cds. |
| BC132221 | Tmem90a | 0.94 | transmembrane protein 90a, mRNA (cDNA clone MGC:163852 IMAGE:40130498), complete cds. |
| BC132263 | Isl1 | 0.94 | ISL1 transcription factor, LIM/homeodomain, mRNA (cDNA clone MGC:163894 IMAGE:40130540), complete cds. |
| BC152781 | Gm15386 | 0.94 | predicted gene, OTTMUSG00000020946 (OTTMUSG00000020946) mRNA, encodes complete protein. |
| BC152867 | 1110032A03Rik | 0.94 | encodes complete protein. |
| BC152869 | Hba-a2 | 0.94 | hemoglobin alpha, adult chain 2 (Hba-a2) mRNA, encodes complete protein. |
| EF651820 | Gm3264 | 0.94 | alpha23-takusan mRNA, complete cds. |
| EF651840 | Gm3642 | 0.94 | alpha43-takusan mRNA, complete cds. |
| NM_001085513 | Fam131c | 0.94 | gene model 693, (NCBI) (Gm693), mRNA. |
| NM_001085529 | Slc2a7 | 0.94 | novel member of the solute carrier family 2 (facilitated glucose transporter) Slc2 family (LOC435818), mRNA. |
| NM_001101483 | Tmem22 | 0.94 | transmembrane protein 22 (Tmem22), mRNA. |
| NM_001102468 | Calml4 | 0.94 | calmodulin-like 4 (Calml4), transcript variant 2, mRNA. |
| X06454 | C4a | 0.94 | sex-limited protein Slp(w7) alpha-gamma chain. |
| X66903 | Fblim1 | 0.94 | En-2/lacZ junction mRNA (Gt10). |
| XM_001473755 | Gm2488 | 0.94 | Ubtf protein (LOC100039907), mRNA. |
| XM_001474391 | Gm2775 | 0.94 | MGC58416 protein (LOC100040445), mRNA. |
| AB359229 | H2-T22 | 0.94 | H2-T22 mRNA for MHC classIb T22, complete cds. |
| AF041861 | Synj2 | 0.94 | synaptojanin 2 isoform zeta mRNA, partial cds. |
| AF115848 | Unc13b | 0.94 | renal munc13 mRNA, complete cds. |
| AF461114 | Fmr1 | 0.94 | alt 'FMRP#Fmr-1' |
| AF535869 | Rgs19 | 0.94 | GAIP/RGS19 mRNA, complete cds |
| AK009836 | 2310046A06Rik | 0.94 | hypothetical protein, full insert sequence. |
| AK014556 | Eps8l1 | 0.94 | similar to CDNA FLJ20258 FIS, CLONE COLF7250 [Homo sapiens], full insert sequence. |
| AK014832 | Dscaml1 | 0.94 | hypothetical protein, full insert sequence. |
| AK016516 | Efcab6 | 0.94 | hypothetical EF-hand containing protein, full insert sequence. |
| AK017116 | Pisd-ps3 | 0.94 | DJ858B16.2 (PHOSPHATIDYLSERINE DECARBOXYLASE (PSSC, EC 4.1.1.65)) [Homo sapiens], full insert sequence. |
| AK017302 | Psrc1 | 0.94 | differential display and activated by p53, full insert sequence. |
| AK017394 | Gas7 | 0.94 | growth arrest specific 7, full insert sequence. |
| AK028475 | Sept11 | 0.94 | SEPTIN6 TYPE II [Homo sapiens], full insert sequence. |
| AK028679 | Asph | 0.94 | aspartate-beta-hydroxylase, full insert sequence. |
| AK029166 | Txndc11 | 0.94 | hypothetical Thioredoxin containing protein, full insert sequence. |
| AK029371 | Ablim1 | 0.94 | ACTIN-BINDING DOUBLE ZINC FINGER PROTEIN (FRAGMENT) [Mus musculus], full insert sequence. |
| AK029781 | 1700007E06Rik | 0.94 | unclassifiable, full insert sequence. |
| AK032070 | Camkk2 | 0.94 | CA+/CALMODULIN-DEPENDENT PROTEIN KINASE KINASE BETA (CAM-KINASE KINASE BETA) homolog [Rattus norvegicus], full insert sequence. |
| AK032256 | Shroom2 | 0.94 | APICAL-LIKE PROTEIN (APXL PROTEIN) [Homo sapiens], full insert sequence. |
| AK032417 | Arpp21 | 0.94 | protein phosphatase 1, regulatory (inhibitor) subunit 1C, full insert sequence. |
| AK038020 | C77080 | 0.94 | hypothetical Proline-rich region profile/Serine-rich region profile containing protein, full insert sequence. |
| AK038097 | Elmo2 | 0.94 | engulfment and cell motility 2, ced-12 homolog (C. elegans), full insert sequence. |
| AK045675 | Tmem44 | 0.94 | hypothetical protein, full insert sequence. |
| AK047094 | Tnks2 | 0.94 | TANKYRASE-RELATED PROTEIN (FRAGMENT) homolog [Homo sapiens], full insert sequence. |
| AK050586 | C920006O11Rik | 0.94 | unclassifiable, full insert sequence. |
| AK052936 | Fhl2 | 0.94 | SKELETAL MUSCLE LIM-PROTEIN 3 (SLIM 3) (LIM-DOMAIN PROTEIN DRAL) (FOUR AND A HALF LIM DOMAINS PROTEIN 2) (FHL-2), full insert sequence. |
| AK054522 | Nr1d2 | 0.94 | ORPHAN NUCLEAR RECEPTOR NR1D2 (REV-ERB-BETA) (EAR4) homolog [Rattus norvegicus], full insert sequence. |
| AK078032 | BC024139 | 0.94 | hypothetical Growth-Arrest-Specific Protein 2 Domain/Bacterial regulatory protein, LysR family containing protein, full insert sequence. |
| AK078368 | 6530418L21Rik | 0.94 | hypothetical (Trans)glycosidases structure containing protein, full insert sequence. |
| AK080626 | 4732418C07Rik | 0.94 | hypothetical EF-hand/Protein splicing (intein) containing protein, full insert sequence. |
| AK090038 | Rpl7l1 | 0.94 | hypothetical protein, full insert sequence. |
| AK131128 | A430107D22Rik | 0.94 | mFLJ00087 protein. |
| AK135776 | Pdzrn3 | 0.94 | semaF cytoplasmic domain associated protein 3, full insert sequence. |
| AK144183 | Ccdc80 | 0.94 | URB precursor, full insert sequence. |
| AK147518 | Kalrn | 0.94 | Kalirin-7c isoform homolog [Rattus norvegicus], full insert sequence. |
| AK147657 | Camk1d | 0.94 | calcium/calmodulin-dependent protein kinase ID, full insert sequence. |
| AK149445 | Chrd | 0.94 | chordin, full insert sequence. |
| AK154975 | Tbc1d5 | 0.94 | TBC1 domain family, member 5, full insert sequence. |
| AK155221 | Agfg2 | 0.94 | HIV-1 Rev binding protein-like, full insert sequence. |
| AK157542 | Ankrd13c | 0.94 | hypothetical Ankyrin containing protein, full insert sequence. |
| AK158851 | Psd | 0.94 | Exchange factor for ARF6 homolog [Rattus norvegicus], full insert sequence. |
| AK162965 | 2610507I01Rik | 0.94 | unclassifiable, full insert sequence. |
| AK166840 | Dusp14 | 0.94 | dual specificity phosphatase 14, full insert sequence. |
| AK170117 | Rgp1 | 0.94 | hypothetical protein, full insert sequence. |
| AK170368 | Lilrb4 | 0.94 | glycoprotein 49 B, full insert sequence. |
| AK173319 | Rnf169 | 0.94 | mKIAA1991 protein. |
| AL358753 | Lingo1 | 0.94 | EUROIMAGE 425482. |
| AY968048 | Dclk1 | 0.94 | CLICK-I beta mRNA, complete cds. |
| BC003488 | Lmo4 | 0.94 | LIM domain only 4, mRNA (cDNA clone MGC:6561 IMAGE:2811636), complete cds. |
| BC004013 | Tmem49 | 0.94 | transmembrane protein 49, mRNA (cDNA clone MGC:7590 IMAGE:3493738), complete cds. |
| BC004797 | 0610040J01Rik | 0.94 | RIKEN cDNA 0610040J01 gene, mRNA (cDNA clone MGC:8120 IMAGE:3589105), complete cds. |
| BC005583 | Darc | 0.94 | Duffy blood group, chemokine receptor, mRNA (cDNA clone MGC:11454 IMAGE:3968984), complete cds. |
| BC013223 | Stard8 | 0.94 | START domain containing 8, mRNA (cDNA clone IMAGE:4023042). |
| BC018315 | Igh-6 | 0.94 | immunoglobulin heavy chain 6 (heavy chain of IgM), mRNA (cDNA clone MGC:18788 IMAGE:4189350), complete cds. |
| BC022917 | Ric8 | 0.94 | resistance to inhibitors of cholinesterase 8 homolog (C. elegans), mRNA (cDNA clone MGC:27540 IMAGE:4460521), complete cds. |
| BC023331 | N/A | 0.94 | clone IMAGE:4500938, mRNA. |
| BC025858 | 2210021J22Rik | 0.94 | RIKEN cDNA 2210021J22 gene, mRNA (cDNA clone MGC:32213 IMAGE:5007877), complete cds. |
| BC026428 | Gdpd5 | 0.94 | glycerophosphodiester phosphodiesterase domain containing 5, mRNA (cDNA clone MGC:31332 IMAGE:4224574), complete cds. |
| BC026939 | Fam132a | 0.94 | C1q domain containing 2, mRNA (cDNA clone MGC:25716 IMAGE:3966551), complete cds. |
| BC030471 | Rtn4rl1 | 0.94 | reticulon 4 receptor-like 1, mRNA (cDNA clone MGC:40829 IMAGE:5368560), complete cds. |
| BC031417 | Fntb | 0.94 | farnesyltransferase, CAAX box, beta, mRNA (cDNA clone MGC:6966 IMAGE:3154478), complete cds. |
| BC031442 | Crybb3 | 0.94 | crystallin, beta B3, mRNA (cDNA clone MGC:25487 IMAGE:4501690), complete cds. |
| BC033533 | Phf23 | 0.94 | PHD finger protein 23, mRNA (cDNA clone MGC:25514 IMAGE:2655211), complete cds. |
| BC035531 | Nrn1 | 0.94 | neuritin 1, mRNA (cDNA clone MGC:40786 IMAGE:5367281), complete cds. |
| BC040363 | Smarcc2 | 0.94 | SWI/SNF related, matrix associated, actin dependent regulator of chromatin, subfamily c, member 2, mRNA (cDNA clone IMAGE:4239046). |
| BC052185 | Tecpr1 | 0.94 | RIKEN cDNA 2210010N04 gene, mRNA (cDNA clone IMAGE:6393526), containing frame-shift errors. |
| BC055741 | Lhx2 | 0.94 | LIM homeobox protein 2, mRNA (cDNA clone MGC:67038 IMAGE:6413339), complete cds. |
| BC056484 | Sprn | 0.94 | shadow of prion protein, mRNA (cDNA clone MGC:67455 IMAGE:5696462), complete cds. |
| BC057098 | Zfp341 | 0.94 | zinc finger protein 341, mRNA (cDNA clone MGC:73471 IMAGE:5715767), complete cds. |
| BC058652 | Nlk | 0.94 | nemo like kinase, mRNA (cDNA clone MGC:76386 IMAGE:6406851), complete cds. |
| BC059087 | Myl1 | 0.94 | myosin, light polypeptide 1, mRNA (cDNA clone MGC:70067 IMAGE:30139109), complete cds. |
| BC066861 | Akt3 | 0.94 | thymoma viral proto-oncogene 3, mRNA (cDNA clone MGC:76721 IMAGE:30089997), complete cds. |
| BC085083 | Hmgcr | 0.94 | 3-hydroxy-3-methylglutaryl-Coenzyme A reductase, mRNA (cDNA clone MGC:103269 IMAGE:5320486), complete cds. |
| BC089035 | Thrb | 0.94 | thyroid hormone receptor beta, mRNA (cDNA clone MGC:115768 IMAGE:30695885), complete cds. |
| BC098373 | Anks1b | 0.94 | ankyrin repeat and sterile alpha motif domain containing 1B, mRNA (cDNA clone IMAGE:30621789), complete cds. |
| BC099479 | Lgals1 | 0.94 | lectin, galactose binding, soluble 1, mRNA (cDNA clone MGC:117697 IMAGE:30841237), complete cds. |
| AK030642 | Dner | 0.93 | Delta/notch-like EGF-related receptor, full insert sequence. |
| AK032092 | Sh3bgrl2 | 0.93 | SH3BGRL2-like protein, full insert sequence. |
| BC141529 | Otop2 | 0.93 | Otopetrin 2 (Otop2) mRNA, encodes complete protein. |
| BC146434 | Olfr282 | 0.93 | Olfactory receptor 282 (Olfr282) mRNA, encodes complete protein. |
| BC156087 | Gabbr2 | 0.93 | Gamma-aminobutyric acid (GABA) B receptor 2 (Gabbr2) mRNA, encodes complete protein. |
| NM_002643.3 | PIGF | 0.93 | phosphatidylinositol glycan anchor biosynthesis, class F |
| NG_029522.1 | TACR1 | 0.93 | tachykinin receptor 1 |
| NM_001038707.1 | CDC42SE1 | 0.93 | CDC42 small effector 1 |
| NG_027724.1 | MIPOL1 | 0.93 | mirror-image polydactyly 1 |
| NM_002631.3 | PGD | 0.93 | phosphogluconate dehydrogenase |
| NM_012340.4 | NFATC2 | 0.93 | nuclear factor of activated T-cells, cytoplasmic, calcineurin-dependent 2 |
| NM_005409.4 | CXCL11 | 0.93 | chemokine (C-X-C motif) ligand 11 |
| NM_001204890.1 | ISY1-RAB43 | 0.93 | ISY1-RAB43 readthrough |
| NM_021813.3 | BACH2 | 0.93 | BTB and CNC homology 1, basic leucine zipper transcription factor 2 |
| NM_001100814.2 | TMEM55B | 0.93 | transmembrane protein 55B |
| NG_033243.2 | FUBP1 | 0.93 | far upstream element (FUSE) binding protein 1 |
| NM_024640.3 | YRDC | 0.93 | yrdC N(6)-threonylcarbamoyltransferase domain containing |
| NM_028410.1 | PRKRIR | 0.93 | protein-kinase, interferon-inducible double stranded RNA dependent inhibitor, repressor of (P58 repressor) |
| NM_001286968.1 | JUND | 0.93 | jun D proto-oncogene |
| NG_008204.1 | OTX2 | 0.93 | orthodenticle homeobox 2 |
| NM_003581.4 | NCK2 | 0.93 | NCK adaptor protein 2 |
| NM_001098237 | Zbtb3 | 0.93 | zinc finger and BTB domain containing 3 (Zbtb3), transcript variant 1, mRNA. |
| NM_001110780 | Syn1 | 0.93 | synapsin I (Syn1), transcript variant b, mRNA. |
| S62756 | Thrb | 0.93 | hormone receptor beta subunit |
| XM_001471588 | Nanog | 0.93 | hypothetical protein LOC100038891 (LOC100038891), mRNA. |
| XM_001472292 | Gm6804 | 0.93 | hypothetical LOC627881 (LOC627881), mRNA. |
| XM_001474127 | Pisd-ps2 | 0.93 | hypothetical protein LOC100040128 (LOC100040128), mRNA. |
| XM_001474703 | Gm7262 | 0.93 | predicted gene, EG639116 (EG639116), mRNA. |
| AB255881 | Abca8a | 0.93 | abca8a mRNA for ABC transporter A subfamily member, A8a, complete cds. |
| AK002300 | Alad | 0.93 | aminolevulinate, delta-, dehydratase, full insert sequence. |
| AK014857 | 4921509O09Rik | 0.93 | unclassifiable, full insert sequence. |
| AK028305 | Zic4 | 0.93 | zinc finger protein of the cerebellum 4, full insert sequence. |
| AK031951 | Ece2 | 0.93 | ENDOTHELIN CONVERTING ENZYME-2 homolog [Mus musculus], full insert sequence. |
| AK039097 | Rpgrip1 | 0.93 | retinitis pigmentosa GTPase regulator interacting protein 1, full insert sequence. |
| AK039945 | Txndc16 | 0.93 | hypothetical Thioredoxin-like structure containing protein, full insert sequence. |
| AK047790 | Kdm5d | 0.93 | selected mouse cDNA on the Y, full insert sequence. |
| AK078163 | Snip1 | 0.93 | :hypothetical Forkhead-associated (FHA) domain containing protein; SIMILAR TO C.ELEGANS PROTEIN CE08529) (FRAGMENT) homolog [Homo sapiens], full insert sequence. |
| AK079651 | Arhgap6 | 0.93 | rho GTPase activating protein 6, full insert sequence. |
| AK082072 | LOC553095 | 0.93 | unclassifiable, full insert sequence. |
| AK085526 | 1110021J02Rik | 0.93 | clone:D630037N19 product:hypothetical protein, full insert sequence. |
| AK151257 | Ammecr1l | 0.93 | weakly similar to AMME syndrome candidate gene 1 protein [Homo sapiens], full insert sequence. |
| AK159322 | Hmgb3 | 0.93 | high mobility group box 3, full insert sequence. |
| AK161957 | Usp15 | 0.93 | ubiquitin specific protease 15, full insert sequence. |
| AK167690 | Ap1s2 | 0.93 | adaptor-related protein complex 1, sigma 2 subunit, full insert sequence. |
| BC002172 | Gdpd3 | 0.93 | glycerophosphodiester phosphodiesterase domain containing 3, mRNA (cDNA clone MGC:7338 IMAGE:3486726), complete cds. |
| BC004638 | Sparc | 0.93 | secreted acidic cysteine rich glycoprotein, mRNA (cDNA clone MGC:6232 IMAGE:3586402), complete cds. |
| BC025131 | Mfap5 | 0.93 | microfibrillar associated protein 5, mRNA (cDNA clone MGC:35969 IMAGE:3982519), complete cds. |
| BC026021 | Opn1sw | 0.93 | opsin 1 (cone pigments), short-wave-sensitive (color blindness, tritan), mRNA (cDNA clone MGC:28865 IMAGE:4511808), complete cds. |
| BC026696 | Srrm2 | 0.93 | serine/arginine repetitive matrix 2, mRNA (cDNA clone IMAGE:4922676). |
| BC030939 | Wfdc12 | 0.93 | WAP four-disulfide core domain 12, mRNA (cDNA clone MGC:32134 IMAGE:4923469), complete cds. |
| BC043024 | Nts | 0.93 | neurotensin, mRNA (cDNA clone MGC:57895 IMAGE:5691298), complete cds. |
| BC050213 | Fads6 | 0.93 | fatty acid desaturase domain family, member 6, mRNA (cDNA clone IMAGE:30015265). |
| BC050821 | Pmaip1 | 0.93 | phorbol-12-myristate-13-acetate-induced protein 1, mRNA (cDNA clone MGC:59280 IMAGE:6517820), complete cds. |
| BC051919 | Esm1 | 0.93 | endothelial cell-specific molecule 1, mRNA (cDNA clone MGC:62159 IMAGE:6403364), complete cds. |
| BC076631 | Ncapg2 | 0.93 | non-SMC condensin II complex, subunit G2, mRNA (cDNA clone MGC:96880 IMAGE:30637859), complete cds. |
| BC093494 | ENSMUSG00000068790 | 0.93 | Mus musculus predicted gene, ENSMUSG00000068790, mRNA (cDNA clone MGC:102321 IMAGE:2812218), complete cds. |
| BC096416 | N/A | 0.93 | cDNA clone IMAGE:3369166. |
| BC109178 | Rgs10 | 0.93 | regulator of G-protein signalling 10, mRNA (cDNA clone MGC:129414 IMAGE:40049527), complete cds. |
| BC113184 | Gpr165 | 0.93 | G protein-coupled receptor 165, mRNA (cDNA clone MGC:132943 IMAGE:40061761), complete cds. |
| BC113759 | Gjc2 | 0.93 | gap junction protein, chi 2, mRNA (cDNA clone MGC:130506 IMAGE:40046961), complete cds. |
| BC120574 | Olfr397 | 0.93 | olfactory receptor 397, mRNA (cDNA clone MGC:155811 IMAGE:40129497), complete cds. |
| BC127994 | Olfr1170 | 0.93 | olfactory receptor 1170, mRNA (cDNA clone MGC:157568 IMAGE:40135743), complete cds. |
| BC132178 | Olfr113 | 0.93 | olfactory receptor 113, mRNA (cDNA clone MGC:163809 IMAGE:40130455), complete cds. |
| BC146344 | Olfr493 | 0.93 | olfactory receptor 493 (Olfr493) mRNA, encodes complete protein. |
| NM_001085530 | Gm13298 | 0.93 | hypothetical LOC545611 (LOC545611), mRNA. |
| XM_001473614 | Gm2378 | 0.93 | pORF2 (LOC100039703), mRNA. |
| XM_001473893 | Hmcn2 | 0.93 | hypothetical LOC670182 (LOC670182), mRNA. |
| AB013097 | Rnf112 | 0.93 | brain finger protein, complete cds. |
| AF075435 | Trp63 | 0.93 | TA*p63 beta mRNA, complete cds. |
| AK009188 | 2310006M14Rik | 0.93 | hypothetical protein, full insert sequence. |
| AK012717 | Arhgap12 | 0.93 | hypothetical Src homology 3 (SH3) domain profile/Repeat in HS1/Cortactin/WW/rsp5/WWP domain profile/WW / rsp5 / WWP domain/Src homology 3 (SH3) domain containing protein, full insert sequence. |
| AK020369 | Shisa4 | 0.93 | hypothetical protein, full insert sequence. |
| AK032194 | N/A | 0.93 | unclassifiable, full insert sequence. |
| AK032424 | Dclk1 | 0.93 | double cortin and calcium/calmodulin-dependent protein kinase-like 1, full insert sequence. |
| AK033077 | Dlgap2 | 0.93 | DISKS LARGE-ASSOCIATED PROTEIN 1 (DAP-1) (GUANYLATE KINASE-ASSOCIATED PROTEIN) (SAP90/PSD-95-ASSOCIATED PROTEIN 1) (SAPAP1) (PSD-95/SAP90 BINDING PROTEIN 1) (FRAGMENT) [Mus musculus], full insert sequence. |
| AK033525 | N/A | 0.93 | telomerase associated protein 1, full insert sequence. |
| AK035922 | Ncoa1 | 0.93 | nuclear receptor coactivator 1, full insert sequence. |
| AK046727 | 9130024F11Rik | 0.93 | unclassifiable, full insert sequence. |
| AK047931 | Zfp826 | 0.93 | zinc finger protein ZNF126 (fragment) [Homo sapiens], full insert sequence. |
| AK049874 | 0610007P08Rik | 0.93 | PUTATIVE REPAIR AND RECOMBINATION HELICASE RAD26L (FRAGMENT) homolog [Mus musculus], full insert sequence. |
| AK049897 | Hook1 | 0.93 | DJ782L23.1 (HOOK1) (FRAGMENT) homolog [Homo sapiens], full insert sequence. |
| AK050333 | Pik3r4 | 0.93 | ADAPTOR PROTEIN homolog [Homo sapiens], full insert sequence. |
| AK079821 | Ptk2 | 0.93 | PTK2 protein tyrosine kinase 2, full insert sequence. |
| AK083197 | 9330132A10Rik | 0.93 | hypothetical protein, full insert sequence. |
| AK088538 | Mex3b | 0.93 | unclassifiable, full insert sequence. |
| AK133401 | Ypel1 | 0.93 | yippee-like 1 (Drosophila), full insert sequence. |
| AK140849 | Cux2 | 0.93 | cut-like 2 (Drosophila), full insert sequence. |
| AK142396 | Acvr1c | 0.93 | activin A receptor, type IC, full insert sequence. |
| AK144100 | N/A | 0.93 | deltex 4 homolog (Drosophila), full insert sequence. |
| AK144963 | 6030446N20Rik | 0.93 | hypothetical Peptidase S8 and S53, subtilisin, kexin, sedolisin containing protein, full insert sequence. |
| AK147384 | Dagla | 0.93 | neural stem cell-derived dendrite regulator, full insert sequence. |
| AK147647 | Tbc1d24 | 0.93 | Hypothetical RabGAP/TBC domain containing protein, full insert sequence. |
| AK155956 | St6galnac4 | 0.93 | sialyltransferase 7 ((alpha-N-acetylneuraminyl 2,3-betagalactosyl-1,3)-N-acetyl galactosaminide alpha-2,6-sialyltransferase) D, full insert sequence. |
| AK158826 | Thrb | 0.93 | thyroid hormone receptor beta, full insert sequence. |
| AK161601 | Cryba4 | 0.93 | crystallin, beta A4, full insert sequence. |
| AK162220 | Rapgef5 | 0.93 | Rap guanine nucleotide exchange factor (GEF) 5, full insert sequence. |
| AK171529 | Galnt9 | 0.93 | B6-derived CD11 +ve dendritic cells cDNA, RIKEN full-length enriched library, clone:F730227C10 product:UDP-N-acetyl-alpha-D-galactosamine:polypeptide N-acetylgalactosaminyltransferase 9, full insert sequence. |
| AK173143 | Birc6 | 0.93 | mKIAA1289 protein. |
| AK220418 | Tmem132b | 0.93 | mKIAA1786 protein. |
| AK220501 | Slc4a10 | 0.93 | mKIAA4136 protein. |
| AY257970 | Itsn1 | 0.93 | intersectin 1 isoform 7 (Itsn1) mRNA, partial cds |
| BC003309 | Tmem79 | 0.93 | transmembrane protein 79, mRNA (cDNA clone MGC:6976 IMAGE:3154669), complete cds. |
| BC003886 | Gmpr2 | 0.93 | guanosine monophosphate reductase 2, mRNA (cDNA clone MGC:6736 IMAGE:3590411), complete cds. |
| BC005622 | Exosc9 | 0.93 | exosome component 9, mRNA (cDNA clone MGC:11686 IMAGE:3711930), complete cds. |
| BC006806 | Lenep | 0.93 | lens epithelial protein, mRNA (cDNA clone MGC:11754 IMAGE:3152957), complete cds. |
| BC010245 | Kank2 | 0.93 | ankyrin repeat domain 25, mRNA (cDNA clone MGC:12143 IMAGE:3710765), complete cds. |
| BC010840 | Lasp1 | 0.93 | LIM and SH3 protein 1, mRNA (cDNA clone MGC:5975 IMAGE:3490532), complete cds. |
| BC012898 | 2010002N04Rik | 0.93 | RIKEN cDNA 2010002N04 gene, mRNA (cDNA clone MGC:19279 IMAGE:4013357), complete cds. |
| BC014736 | Chgb | 0.93 | chromogranin B, mRNA (cDNA clone MGC:25417 IMAGE:4511248), complete cds. |
| BC015292 | N/A | 0.93 | cDNA clone MGC:19136 IMAGE:4216659, complete cds. |
| BC016217 | Dgkz | 0.93 | diacylglycerol kinase zeta, mRNA (cDNA clone IMAGE:4483006), partial cds. |
| BC017134 | Eltd1 | 0.93 | EGF, latrophilin seven transmembrane domain containing 1, mRNA (cDNA clone MGC:27649 IMAGE:4511572), complete cds. |
| BC018307 | Cyp46a1 | 0.93 | cytochrome P450, family 46, subfamily a, polypeptide 1, mRNA (cDNA clone MGC:18311 IMAGE:4195579), complete cds. |
| BC019785 | Hyou1 | 0.93 | hypoxia up-regulated 1, mRNA (cDNA clone MGC:30561 IMAGE:5151731), complete cds. |
| BC020004 | Gprc5b | 0.93 | G protein-coupled receptor, family C, group 5, member B, mRNA (cDNA clone MGC:25522 IMAGE:3499841), complete cds. |
| BC020991 | Lipg | 0.93 | lipase, endothelial, mRNA (cDNA clone MGC:13719 IMAGE:3981856), complete cds. |
| BC022913 | Slc48a1 | 0.93 | RIKEN cDNA 4930570C03 gene, mRNA (cDNA clone MGC:28670 IMAGE:4237364), complete cds. |
| BC023237 | Edem1 | 0.93 | ER degradation enhancer, mannosidase alpha-like 1, mRNA (cDNA clone MGC:25513 IMAGE:2654113), complete cds. |
| BC026054 | Nptx2 | 0.93 | neuronal pentraxin 2, mRNA (cDNA clone MGC:13847 IMAGE:4038070), complete cds. |
| BC027224 | Pde4a | 0.93 | phosphodiesterase 4A, cAMP specific, mRNA (cDNA clone IMAGE:4167351), partial cds. |
| BC027404 | Trmt1 | 0.93 | TRM1 tRNA methyltransferase 1 homolog (S. cerevisiae), mRNA (cDNA clone MGC:37065 IMAGE:4950980), complete cds. |
| BC027746 | Plcl2 | 0.93 | phospholipase C-like 2, mRNA (cDNA clone MGC:29392 IMAGE:5065782), complete cds. |
| BC028249 | Igh | 0.93 | immunoglobulin heavy chain complex, mRNA (cDNA clone MGC:40652 IMAGE:3487241), complete cds. |
| BC028540 | Igk | 0.93 | cDNA clone MGC:41421 IMAGE:3371732, complete cds. |
| BC031533 | 8430408G22Rik | 0.93 | RIKEN cDNA 8430408G22 gene, mRNA (cDNA clone MGC:6835 IMAGE:2649431), complete cds. |
| BC031924 | Ephb6 | 0.93 | Eph receptor B6, mRNA (cDNA clone MGC:25301 IMAGE:4911959), complete cds. |
| BC034768 | Tmem14a | 0.93 | transmembrane protein 14A, mRNA (cDNA clone MGC:36280 IMAGE:4039224), complete cds. |
| BC037002 | Adra1b | 0.93 | adrenergic receptor, alpha 1b, mRNA (cDNA clone IMAGE:5097685), partial cds. |
| BC046784 | Tspan5 | 0.93 | tetraspanin 5, mRNA (cDNA clone MGC:61325 IMAGE:6416107), complete cds. |
| BC048431 | Gng13 | 0.93 | guanine nucleotide binding protein 13, gamma, mRNA (cDNA clone MGC:58102 IMAGE:6601662), complete cds. |
| BC049086 | Ccnd2 | 0.93 | cyclin D2, mRNA (cDNA clone MGC:61305 IMAGE:5716186), complete cds. |
| BC050040 | Phka2 | 0.93 | phosphorylase kinase alpha 2, mRNA (cDNA clone MGC:60895 IMAGE:6431743), complete cds. |
| BC050752 | Cap2 | 0.93 | CAP, adenylate cyclase-associated protein, 2 (yeast), mRNA (cDNA clone MGC:58170 IMAGE:6533812), complete cds. |
| BC052687 | Ccdc32 | 0.93 | coiled-coil domain containing 32, mRNA (cDNA clone MGC:60849 IMAGE:30033673), complete cds. |
| BC052880 | Slc39a10 | 0.93 | solute carrier family 39 (zinc transporter), member 10, mRNA (cDNA clone MGC:60701 IMAGE:30045566), complete cds. |
| BC053089 | Kcnf1 | 0.93 | potassium voltage-gated channel, subfamily F, member 1, mRNA (cDNA clone MGC:62472 IMAGE:5709192), complete cds. |
| BC053527 | Inhba | 0.93 | inhibin beta-A, mRNA (cDNA clone MGC:58937 IMAGE:6528822), complete cds. |
| BC055718 | Fezf2 | 0.93 | Fez family zinc finger 2, mRNA (cDNA clone MGC:66830 IMAGE:6415359), complete cds. |
| BC056232 | Rrp12 | 0.93 | ribosomal RNA processing 12 homolog (S. cerevisiae), mRNA (cDNA clone MGC:60855 IMAGE:30060906), complete cds. |
| BC057588 | Sema3a | 0.93 | sema domain, immunoglobulin domain (Ig), short basic domain, secreted, (semaphorin) 3A, mRNA (cDNA clone MGC:67120 IMAGE:6416990), complete cds. |
| BC058099 | Ap2a2 | 0.93 | adaptor protein complex AP-2, alpha 2 subunit, mRNA (cDNA clone MGC:67130 IMAGE:6825406), complete cds. |
| BC058616 | Ccdc3 | 0.93 | coiled-coil domain containing 3, mRNA (cDNA clone MGC:57889 IMAGE:5686139), complete cds. |
| BC059070 | Trim37 | 0.93 | tripartite motif protein 37, mRNA (cDNA clone MGC:69911 IMAGE:6825999), complete cds. |
| BC060113 | Sh3rf1 | 0.93 | SH3 domain containing ring finger 1, mRNA (cDNA clone MGC:63290 IMAGE:6405370), complete cds. |
| BC062125 | 6430706D22Rik | 0.93 | RIKEN cDNA 6430706D22 gene, mRNA (cDNA clone MGC:69925 IMAGE:6826662), complete cds. |
| BC062245 | Ooep | 0.93 | oocyte expressed protein homolog (dog), mRNA (cDNA clone MGC:73547 IMAGE:3470921), complete cds. |
| BC066013 | Spred2 | 0.93 | sprouty-related, EVH1 domain containing 2, mRNA (cDNA clone MGC:90041 IMAGE:5706237), complete cds. |
| BC066997 | 3110035E14Rik | 0.93 | RIKEN cDNA 3110035E14 gene, mRNA (cDNA clone MGC:91354 IMAGE:5703513), complete cds. |
| BC075656 | Fbxo27 | 0.93 | F-box protein 27, mRNA (cDNA clone MGC:96367 IMAGE:30540609), complete cds. |
| BC078629 | S100a8 | 0.93 | S100 calcium binding protein A8 (calgranulin A), mRNA (cDNA clone MGC:73590 IMAGE:864657), complete cds. |
| BC085145 | Dyrk2 | 0.93 | dual-specificity tyrosine-(Y)-phosphorylation regulated kinase 2, mRNA (cDNA clone MGC:109676 IMAGE:6808145), complete cds. |
| BC087929 | Gm5169 | 0.93 | Xmr protein, mRNA (cDNA clone MGC:107293 IMAGE:6742946), complete cds. |
| BC089525 | 2310007B03Rik | 0.93 | RIKEN cDNA 2310007B03 gene, mRNA (cDNA clone MGC:107226 IMAGE:30315016), complete cds. |
| BC091738 | Gm8760 | 0.93 | immunoglobulin kappa chain, constant region, mRNA (cDNA clone MGC:102611 IMAGE:4219669), complete cds. |
| BC093479 | Nptx1 | 0.93 | neuronal pentraxin 1, mRNA (cDNA clone MGC:118731 IMAGE:30947054), complete cds. |
| BC094665 | Pip5k1c | 0.93 | phosphatidylinositol-4-phosphate 5-kinase, type 1 gamma, mRNA (cDNA clone MGC:102155 IMAGE:30356181), complete cds. |
| BC096543 | B930095G15Rik | 0.93 | RIKEN cDNA B930095G15 gene, mRNA (cDNA clone MGC:106552 IMAGE:6825849), complete cds. |
| BC096591 | Nup188 | 0.93 | nucleoporin 188, mRNA (cDNA clone MGC:106061 IMAGE:4916413), complete cds. |
| BC096667 | Igh-6 | 0.93 | immunoglobulin heavy chain 6 (heavy chain of IgM), mRNA (cDNA clone MGC:107036 IMAGE:1515459), complete cds. |
| BC098199 | Nptxr | 0.93 | neuronal pentraxin receptor, mRNA (cDNA clone MGC:106462 IMAGE:5705707), complete cds. |
| BC130244 | Olfr341 | 0.92 | Olfactory receptor 341, mRNA (cDNA clone MGC:161303 IMAGE:40142305), complete cds. |
| BC148569 | Ipcef1 | 0.92 | RIKEN cDNA A130090K04 gene (A130090K04Rik) mRNA, encodes complete protein. |
| NG_029727.1 | PDGFA | 0.92 | platelet-derived growth factor alpha polypeptide |
| XM_420763.5 | GPR125 | 0.92 | G protein-coupled receptor 125 |
| NM_001079533.1 | CPEB1 | 0.92 | cytoplasmic polyadenylation element binding protein 1 |
| NM_007030.2 | TPPP | 0.92 | tubulin polymerization promoting protein |
| NG_029675.1 | FOSB | 0.92 | FBJ murine osteosarcoma viral oncogene homolog B |
| NM_198467.2 | RSBN1L | 0.92 | round spermatid basic protein 1-like |
| NM_006016.4 | CD164 | 0.92 | CD164 molecule, sialomucin |
| NM_001025595.2 | ARFIP1 | 0.92 | ADP-ribosylation factor interacting protein 1 |
| NM_003953.5 | MPZL1 | 0.92 | myelin protein zero-like 1 |
| XM_001471840 | Hjurp | 0.92 | hypothetical protein LOC100038822 (LOC100038822), mRNA. |
| XM_001474669 | 1110021L09Rik | 0.92 | RIKEN cDNA 1110021L09 gene (1110021L09Rik), mRNA. |
| AB201455 | Prkcd | 0.92 | Prkcd mRNA for protein kinase C, delta VI, complete cds. |
| AF453427 | Nudt6 | 0.92 | anti-sense basic fibroblast growth factor A (Asfgf2a) mRNA, complete cds. |
| AK012594 | N/A | 0.92 | hypothetical protein, full insert sequence. |
| AK019929 | Mtap7d2 | 0.92 | hypothetical protein, full insert sequence. |
| AK020895 | Gnas | 0.92 | unclassifiable, full insert sequence. |
| AK028023 | Hnrnpk | 0.92 | heterogeneous nuclear ribonucleoprotein K, full insert sequence. |
| AK028047 | Os9 | 0.92 | similar to PROTEIN OS-9 PRECURSOR [Homo sapiens], full insert sequence. |
| AK031460 | A330035P11Rik | 0.92 | mitochondria located 1 homolog (human), full insert sequence. |
| AK032081 | Pmpca | 0.92 | MITOCHONDRIAL PROCESSING PEPTIDASE ALPHA SUBUNIT, MITOCHONDRIAL PRECURSOR (EC 3.4.24.64) (ALPHA-MPP) (P-55) homolog [Rattus norvegicus], full insert sequence. |
| AK038720 | Tcf7l2 | 0.92 | transcription factor 7-like 2, T-cell specific, HMG-box, full insert sequence. |
| AK039035 | Gnas | 0.92 | GNAS (guanine nucleotide binding protein, alpha stimulating) complex locus, full insert sequence. |
| AK040354 | Epb4.1l5 | 0.92 | SIMILAR TO EHM2 GENE homolog [Mus musculus], full insert sequence. |
| AK043183 | Tnrc6b | 0.92 | hypothetical Glutamine-rich region containing protein, full insert sequence. |
| AK048362 | Zfp414 | 0.92 | hypothetical Zinc finger, C2H2 type containing protein, full insert sequence. |
| AK052968 | Slc35d1 | 0.92 | UDP-GLUCURONIC ACID/UDP-N-ACETYLGALACTOSAMINE TRANSPORTER (UDP- GLCA/UDP-GALNAC TRANSPORTER) homolog [Homo sapiens], full insert sequence. |
| AK085039 | Aida | 0.92 | clone:D430028H19 product:hypothetical protein, full insert sequence. |
| AK132829 | N/A | 0.92 | unclassifiable, full insert sequence. |
| AK140957 | AI316807 | 0.92 | Similar to fasciculation and elongation protein zeta 2 (zygin II) homolog [Homo sapiens], full insert sequence. |
| AK146133 | Pcgf5 | 0.92 | hypothetical Zn-finger, RING/Zinc finger RING-type profile containing protein, full insert sequence. |
| AK152518 | Zc3h12a | 0.92 | DJ423B22.1 (Novel protein similar to KIAA0323, KIAA0615 and C.elegans C30F12.1) (Fragment) homolog [Homo sapiens], full insert sequence. |
| AK163401 | Fcgr3 | 0.92 | Fc receptor, IgG, low affinity III, full insert sequence. |
| AK169248 | Ctdspl2 | 0.92 | hypothetical protein, full insert sequence. |
| AK169793 | Centb1 | 0.92 | centaurin, beta 1, full insert sequence. |
| BC001990 | Unc119 | 0.92 | unc-119 homolog (C. elegans), mRNA (cDNA clone MGC:5721 IMAGE:3484396), complete cds. |
| BC003733 | Tspan6 | 0.92 | tetraspanin 6, mRNA (cDNA clone MGC:5801 IMAGE:3590862), complete cds. |
| BC011139 | Gck | 0.92 | glucokinase, mRNA (cDNA clone MGC:18414 IMAGE:4195943), complete cds. |
| BC016212 | Lpo | 0.92 | lactoperoxidase, mRNA (cDNA clone MGC:27708 IMAGE:4924899), complete cds. |
| BC020026 | Nuf2 | 0.92 | NUF2, NDC80 kinetochore complex component, homolog (S. cerevisiae), mRNA (cDNA clone MGC:27942 IMAGE:3587655), complete cds. |
| BC025067 | Snhg8 | 0.92 | cDNA clone IMAGE:4036503, with apparent retained intron. |
| BC025432 | F2rl1 | 0.92 | coagulation factor II (thrombin) receptor-like 1, mRNA (cDNA clone MGC:29183 IMAGE:5006769), complete cds. |
| BC026553 | Ednrb | 0.92 | endothelin receptor type B, mRNA (cDNA clone MGC:36102 IMAGE:4971909), complete cds. |
| BC027104 | Otx2 | 0.92 | orthodenticle homolog 2 (Drosophila), mRNA (cDNA clone MGC:38809 IMAGE:5359966), complete cds. |
| BC030381 | Rfesd | 0.92 | Rieske (Fe-S) domain containing, mRNA (cDNA clone MGC:40940 IMAGE:5375753), complete cds. |
| BC036347 | Clcn5 | 0.92 | chloride channel 5, mRNA (cDNA clone MGC:35965 IMAGE:3989812), complete cds. |
| BC043656 | Eif2s3y | 0.92 | eukaryotic translation initiation factor 2, subunit 3, structural gene Y-linked, mRNA (cDNA clone MGC:49070 IMAGE:5368023), complete cds. |
| BC043680 | Agxt2l1 | 0.92 | alanine-glyoxylate aminotransferase 2-like 1, mRNA (cDNA clone MGC:49277 IMAGE:5101568), complete cds. |
| BC048474 | Dbil5 | 0.92 | diazepam binding inhibitor-like 5, mRNA (cDNA clone MGC:58228 IMAGE:6773240), complete cds. |
| BC048631 | Tcerg1l | 0.92 | transcription elongation regulator 1-like, mRNA (cDNA clone IMAGE:6744153), partial cds. |
| BC050786 | Mdh1b | 0.92 | malate dehydrogenase 1B, NAD (soluble), mRNA (cDNA clone MGC:58554 IMAGE:6703279), complete cds. |
| BC053402 | Elk3 | 0.92 | ELK3, member of ETS oncogene family, mRNA (cDNA clone MGC:60539 IMAGE:30060743), complete cds. |
| BC055400 | Ahi1 | 0.92 | Abelson helper integration site, mRNA (cDNA clone MGC:62786 IMAGE:6467369), complete cds. |
| BC057657 | Nudt12 | 0.92 | nudix (nucleoside diphosphate linked moiety X)-type motif 12, mRNA (cDNA clone MGC:68086 IMAGE:2646355), complete cds. |
| BC080297 | N/A | 0.92 | RIKEN cDNA B930046C15 gene, mRNA (cDNA clone IMAGE:30363602). |
| BC080300 | Dnajc1 | 0.92 | DnaJ (Hsp40) homolog, subfamily C, member 1, mRNA (cDNA clone MGC:90686 IMAGE:30536910), complete cds. |
| BC081450 | Rpl41 | 0.92 | cDNA clone IMAGE:6815275. |
| BC117030 | Oxt | 0.92 | oxytocin, mRNA (cDNA clone MGC:151407 IMAGE:40126349), complete cds. |
| BC120488 | AU021034 | 0.92 | expressed sequence AU021034, mRNA (cDNA clone MGC:155725 IMAGE:40129411), complete cds. |
| BC120768 | Gm4876 | 0.92 | predicted gene, EG232599, mRNA (cDNA clone MGC:156005 IMAGE:40129691), complete cds. |
| BC127960 | Rph3al | 0.92 | rabphilin 3A-like (without C2 domains), mRNA (cDNA clone MGC:157502 IMAGE:40134813), complete cds. |
| BC129973 | N/A | 0.92 | cDNA clone IMAGE:40092570. |
| BC132647 | Serpina3h | 0.92 | serine (or cysteine) peptidase inhibitor, clade A, member 3H, mRNA (cDNA clone MGC:164278 IMAGE:40130924), complete cds. |
| NM_001081299 | Cdh18 | 0.92 | cadherin 18 (Cdh18), mRNA. |
| NM_207162 | LOC382133 | 0.92 | similar to RIKEN cDNA 1700029H17 (LOC382133), mRNA. |
| U63133 | Mela | 0.92 | C-type ecotropic endogenous retrovirus, complete mRNA sequence. |
| AF139059 | Neu2 | 0.92 | sialidase mRNA, complete sequence. |
| AK010756 | Sgsm1 | 0.92 | DJ930L11.1 (SIMILAR TO KIAA0397) (FRAGMENT) homolog |
| AK013054 | Dkk3 | 0.92 | dickkopf homolog 3 (Xenopus laevis), full insert sequence. |
| AK015096 | N/A | 0.92 | telomerase associated protein 1, full insert sequence. |
| AK015907 | Kcnj9 | 0.92 | potassium inwardly-rectifying channel, subfamily J, member 9, full insert sequence. |
| AK016918 | Nbas | 0.92 | RIKEN cDNA 4933425L03 gene, full insert sequence. |
| AK017848 | 5730559C18Rik | 0.92 | hypothetical protein, full insert sequence. |
| AK033147 | 1010001N08Rik | 0.92 | unclassifiable, full insert sequence. |
| AK035040 | Lsm14b | 0.92 | BA11M20.3.1 (NOVEL PROTEIN SIMILAR TO PLEURODELES WALTLII RAP55 PROTEIN, ISOFORM 1) [Homo sapiens], full insert sequence. |
| AK035262 | Ank3 | 0.92 | ankyrin 3, epithelial, full insert sequence. |
| AK038450 | Kcnv1 | 0.92 | POTASSIUM CHANNEL KV8.1 homolog [Mesocricetus auratus], full insert sequence. |
| AK042641 | Syt15 | 0.92 | synaptotagmin 15, full insert sequence. |
| AK045359 | Rapgefl1 | 0.92 | B230105J10 product:inferred: Link guanine nucleotide exchange factor II {Homo sapiens}, full insert sequence. |
| AK048637 | Antxr1 | 0.92 | ANTRAX TOXIN RECEPTOR PRECURSOR (TUMOR ENDOTHELIAL MARKER 8) homolog [Mus musculus], full insert sequence. |
| AK050641 | Osbpl1a | 0.92 | oxysterol binding protein-like 1A, full insert sequence. |
| AK052858 | Mylk3 | 0.92 | myosin light chain kinase, full insert sequence. |
| AK082779 | Atrx | 0.92 | X-linked nuclear protein, full insert sequence. |
| AK088909 | Trip10 | 0.92 | SALT-TOLERANT PROTEIN homolog [Rattus norvegicus], full insert sequence. |
| AK133342 | Znrf3 | 0.92 | hypothetical Zn-finger, RING/Zinc finger RING-type profile/Serine-rich region profile containing protein, full insert sequence. |
| AK137067 | Gpam | 0.92 | glycerol-3-phosphate acyltransferase, mitochondrial, full insert sequence. |
| AK141081 | Nphs1 | 0.92 | nephrosis 1 homolog, nephrin (human), full insert sequence. |
| AK145286 | Cep68 | 0.92 | hypothetical protein, full insert sequence. |
| AK147419 | Hivep2 | 0.92 | human immunodeficiency virus type I enhancer binding protein 2, full insert sequence. |
| AK148317 | Ulk1 | 0.92 | Unc-51 like kinase 1 (C. elegans), full insert sequence. |
| AK150563 | Cdc42 | 0.92 | cell division cycle 42 homolog (S. cerevisiae), full insert sequence. |
| AK157053 | Kcnab2 | 0.92 | potassium voltage-gated channel, shaker-related subfamily, beta member 2, full insert sequence. |
| AK165375 | Fam78a | 0.92 | hypothetical protein, full insert sequence. |
| AK167050 | Rbm28 | 0.92 | hypothetical RNA-binding region RNP-1 (RNA recognition motif) containing protein, full insert sequence. |
| AY587571 | Espn | 0.92 | alt 'je' |
| BC002093 | Crip2 | 0.92 | cysteine rich protein 2, mRNA (cDNA clone MGC:6346 IMAGE:3489018), complete cds. |
| BC005481 | Emilin1 | 0.92 | elastin microfibril interfacer 1, mRNA (cDNA clone MGC:7278 IMAGE:3485057), complete cds. |
| BC006643 | N/A | 0.92 | cDNA clone MGC:6582 IMAGE:3483797, complete cds. |
| BC010215 | Plekha2 | 0.92 | pleckstrin homology domain-containing, family A (phosphoinositide binding specific) member 2, mRNA (cDNA clone MGC:7728 IMAGE:3498236), complete cds. |
| BC011511 | Rassf3 | 0.92 | Ras association (RalGDS/AF-6) domain family 3, mRNA (cDNA clone MGC:19436 IMAGE:3495833), complete cds. |
| BC016075 | Rmnd5b | 0.92 | required for meiotic nuclear division 5 homolog B (S. cerevisiae), mRNA (cDNA clone MGC:27569 IMAGE:4485143), complete cds. |
| BC020019 | Mtmr6 | 0.92 | myotubularin related protein 6, mRNA (cDNA clone MGC:27983 IMAGE:3596732), complete cds. |
| BC021460 | Lsm1 | 0.92 | LSM1 homolog, U6 small nuclear RNA associated (S. cerevisiae), mRNA (cDNA clone MGC:29255 IMAGE:5054997), complete cds. |
| BC021751 | H2-T10 | 0.92 | histocompatibility 2, T region locus 10, mRNA (cDNA clone MGC:25390 IMAGE:4165944), complete cds. |
| BC024132 | Sc5d | 0.92 | sterol-C5-desaturase (fungal ERG3, delta-5-desaturase) homolog (S. cerevisae), mRNA (cDNA clone MGC:37898 IMAGE:5101986), complete cds. |
| BC024790 | Rusc2 | 0.92 | RUN and SH3 domain containing 2, mRNA (cDNA clone IMAGE:5357662), partial cds. |
| BC025832 | Notum | 0.92 | notum pectinacetylesterase homolog (Drosophila), mRNA (cDNA clone MGC:37936 IMAGE:5125723), complete cds. |
| BC026127 | Csnk1e | 0.92 | casein kinase 1, epsilon, mRNA (cDNA clone MGC:13740 IMAGE:4010696), complete cds. |
| BC026390 | Sfi1 | 0.92 | Sfi1 homolog, spindle assembly associated (yeast), mRNA (cDNA clone IMAGE:4191452), partial cds. |
| BC026817 | Aacs | 0.92 | acetoacetyl-CoA synthetase, mRNA (cDNA clone MGC:28651 IMAGE:4235112), complete cds. |
| BC029669 | Mgst3 | 0.92 | microsomal glutathione S-transferase 3, mRNA (cDNA clone MGC:35738 IMAGE:5372427), complete cds. |
| BC031436 | Slc47a1 | 0.92 | solute carrier family 47, member 1, mRNA (cDNA clone MGC:28678 IMAGE:4238749), complete cds. |
| BC034862 | Bdnf | 0.92 | brain derived neurotrophic factor, mRNA (cDNA clone MGC:41191 IMAGE:1397218), complete cds. |
| BC037643 | Txnrd1 | 0.92 | thioredoxin reductase 1, mRNA (cDNA clone MGC:46868 IMAGE:5059831), complete cds. |
| BC038256 | Mafb | 0.92 | v-maf musculoaponeurotic fibrosarcoma oncogene family, protein B (avian), mRNA (cDNA clone MGC:47160 IMAGE:5097650), complete cds. |
| BC047138 | Ltk | 0.92 | leukocyte tyrosine kinase, mRNA (cDNA clone MGC:54747 IMAGE:6389834), complete cds. |
| BC052731 | Kcnj11 | 0.92 | potassium inwardly rectifying channel, subfamily J, member 11, mRNA (cDNA clone MGC:64626 IMAGE:6816299), complete cds. |
| BC053752 | Sept10 | 0.92 | septin 10, mRNA (cDNA clone MGC:59570 IMAGE:6506619), complete cds. |
| BC054833 | D10Ertd610e | 0.92 | DNA segment, Chr 10, ERATO Doi 610, expressed, mRNA (cDNA clone MGC:62781 IMAGE:6439728), complete cds. |
| BC057652 | Moxd1 | 0.92 | monooxygenase, DBH-like 1, mRNA (cDNA clone MGC:67985 IMAGE:4017199), complete cds. |
| BC060157 | Pak1 | 0.92 | p21 (CDKN1A)-activated kinase 1, mRNA (cDNA clone IMAGE:6810707). |
| BC062158 | Mybpc1 | 0.92 | RIKEN cDNA 8030451F13 gene, mRNA (cDNA clone MGC:70062 IMAGE:30134842), complete cds. |
| BC064041 | Homer1 | 0.92 | homer homolog 1 (Drosophila), mRNA (cDNA clone MGC:73537 IMAGE:1398649), complete cds. |
| BC066062 | Bmp1 | 0.92 | bone morphogenetic protein 1, mRNA (cDNA clone MGC:86027 IMAGE:6849066), complete cds. |
| BC070399 | Zfp366 | 0.92 | zinc finger protein 366, mRNA (cDNA clone MGC:99415 IMAGE:5694313), complete cds. |
| BC075657 | Stra6 | 0.92 | stimulated by retinoic acid gene 6, mRNA (cDNA clone MGC:96382 IMAGE:30605532), complete cds. |
| BC085184 | Fbxw7 | 0.92 | F-box and WD-40 domain protein 7, archipelago homolog (Drosophila), mRNA (cDNA clone MGC:109692 IMAGE:30664813), complete cds. |
| BC094384 | Pgbd5 | 0.92 | piggyBac transposable element derived 5, mRNA (cDNA clone MGC:106643 IMAGE:30606749), complete cds. |
| AK009502 | Tia1 | 0.91 | Cytotoxic granule-associated RNA binding protein 1 |
| AK011205 | Rbms1 | 0.91 | Unclassifiable, full insert sequence. |
| BC129872 | Atp8a1 | 0.91 | ATPase, aminophospholipid transporter (APLT), class I, type 8A, member 1, mRNA (cDNA clone MGC:150131 IMAGE:40110214), complete cds. |
| BC131919 | A730008H23Rik | 0.91 | RIKEN cDNA A730008H23 gene, mRNA (cDNA clone MGC:163550 IMAGE:40130196), complete cds. |
| BC146355 | Tmem195 | 0.91 | Transmembrane protein 195 (Tmem195) mRNA, encodes complete protein. |
| NM_005381.2 | NCL | 0.91 | nucleolin |
| NM_020839.3 | WDR48 | 0.91 | WD repeat domain 48 |
| NG_016281.1 | SEC23B | 0.91 | Sec23 homolog B (S. cerevisiae) |
| NM_198490.2 | RAB43 | 0.91 | RAB43, member RAS oncogene family |
| NM_022474.3 | MPP5 | 0.91 | membrane protein, palmitoylated 5 (MAGUK p55 subfamily member 5) |
| NM_003714.2 | ZMAT3 | 0.91 | zinc finger, matrin-type 3 |
| NM_003246.3 | THBS1 | 0.91 | thrombospondin 1 |
| NM_002841.3 | PTPRG | 0.91 | protein tyrosine phosphatase, receptor type, G |
| NG_012742.2 | KALRN | 0.91 | kalirin, RhoGEF kinase |
| NG_008270.1 | SEC63 | 0.91 | SEC63 homolog (S. cerevisiae) |
| NC_018913.2 | POTEI | 0.91 | POTE ankyrin domain family, member I |
| NM_015017.4 | USP33 | 0.91 | ubiquitin specific peptidase 33 |
| NG_007484.2 | CDK4 | 0.91 | cyclin-dependent kinase 4 |
| NM_014905.4 | GLS | 0.91 | glutaminase |
| NG_033910.1 | VPS45 | 0.91 | vacuolar protein sorting 45 homolog (S. cerevisiae) |
| NG_012196.1 | FN1 | 0.91 | fibronectin 1 |
| NM_003100.3 | SNX2 | 0.91 | sorting nexin 2 |
| NM_152641.2 | ARID2 | 0.91 | AT rich interactive domain 2 (ARID, RFX-like) |
| NG_032912.1 | FLI1 | 0.91 | Fli-1 proto-oncogene, ETS transcription factor |
| BC156236 | Amigo3 | 0.91 | adhesion molecule with Ig like domain 3 (Amigo3) mRNA, encodes complete protein. |
| DQ065607 | Nkain2 | 0.91 | T-cell lymphoma breakpoint-associated target 1 long isoform (Tcba1) mRNA, complete cds. |
| NM_001081345 | Chd2 | 0.91 | chromodomain helicase DNA binding protein 2 (Chd2), mRNA. |
| NR_003518 | Pisd-ps3 | 0.91 | RIKEN cDNA 4933439C20 gene (4933439C20Rik) on chromosome Y. |
| XM_001004778 | EG668525 | 0.91 | predicted gene, EG668525 (EG668525), mRNA. |
| XM_001474442 | Gm9639 | 0.91 | similar to A030009A09Rik protein (LOC675294), mRNA. |
| XM_001474780 | Mbnl1 | 0.91 | hypothetical protein LOC100040485 (LOC100040485), mRNA. |
| AK002541 | 1190007F08Rik | 0.91 | unclassifiable, full insert sequence. |
| AK016837 | Wdr37 | 0.91 | unclassifiable, full insert sequence. |
| AK019339 | 2900016B01Rik | 0.91 | unclassifiable, full insert sequence. |
| AK020674 | Itgav | 0.91 | integrin alpha V, full insert sequence. |
| AK027957 | 1110035M17Rik | 0.91 | unclassifiable, full insert sequence. |
| AK031649 | Hs6st2 | 0.91 | heparan sulfate 6-O-sulfotransferase 2, full insert sequence. |
| AK032260 | Cacng5 | 0.91 | voltage-dependent calcium channel gamma-5 subunit, full insert sequence. |
| AK039170 | Gorab | 0.91 | hypothetical protein, full insert sequence. |
| AK040441 | Fcgbp | 0.91 | similar to IGG FC BINDING PROTEIN (FRAGMENT) [Homo sapiens], full insert sequence. |
| AK040597 | Scml4 | 0.91 | sex comb on midleg-like 4 (Drosophila), full insert sequence. |
| AK047603 | Sufu | 0.91 | suppressor of fused homolog (Drosophila), full insert sequence. |
| AK048446 | Ikbkg | 0.91 | unclassifiable, full insert sequence. |
| AK050000 | C530008M17Rik | 0.91 | similar to HYPOTHETICAL 67.3 KDA PROTEIN (FRAGMENT) [Homo sapiens], full insert sequence. |
| AK052631 | Dnahc1 | 0.91 | hypothetical Dynein heavy chain containing protein, full insert sequence. |
| AK052773 | D630048o14rik | 0.91 | unclassifiable, full insert sequence. |
| AK053225 | E030046B03Rik | 0.91 | unclassifiable, full insert sequence. |
| AK053578 | Rassf4 | 0.91 | similar to AD037 [Homo sapiens], full insert sequence. |
| AK078264 | Fam70a | 0.91 | HYPOTHETICAL 38.5 KDA PROTEIN homolog [Macaca fascicularis], full insert sequence. |
| AK082321 | AW551984 | 0.91 | similar to LOSS OF HETEROZYGOSITY, 11, CHROMOSOMAL REGION 2, GENE A [Homo sapiens], full insert sequence. |
| AK132661 | Bbs7 | 0.91 | Bardet-Biedl syndrome 7, full insert sequence. |
| AK133186 | N/A | 0.91 | hypothetical protein, full insert sequence. |
| AK133611 | Naalad2 | 0.91 | N-acetylated alpha-linked acidic dipeptidase 2, full insert sequence. |
| AK134741 | Ptger1 | 0.91 | Protein kinase C-like 1 (EC 2.7.1.-) (Protein-kinase C-related kinase 1) (Protein kinase C-like PKN) (Serine-threonine protein kinase N) (Fragment), full insert sequence. |
| AK136042 | B4galt4 | 0.91 | UDP-Gal:betaGlcNAc beta 1,4-galactosyltransferase, polypeptide 4, full insert sequence. |
| AK137440 | St18 | 0.91 | suppression of tumorigenicity 18, full insert sequence. |
| AK146386 | N/A | 0.91 | RIKEN full-length enriched library, clone:I730068L11 product:unclassifiable, full insert sequence. |
| AK146701 | Espn | 0.91 | espin, full insert sequence. |
| AK149350 | Dhodh | 0.91 | dihydroorotate dehydrogenase, full insert sequence. |
| AK155769 | Lamb3 | 0.91 | laminin, beta 3, full insert sequence. |
| AK158392 | Oas3 | 0.91 | 2'-5' oligoadenylate synthetase 3, full insert sequence. |
| AK161361 | 1700071M16Rik | 0.91 | unclassifiable, full insert sequence. |
| AK163438 | Nxph1 | 0.91 | neurexophilin 1, full insert sequence. |
| BC003297 | Aldh9a1 | 0.91 | aldehyde dehydrogenase 9, subfamily A1, mRNA (cDNA clone MGC:6698 IMAGE:3583976), complete cds. |
| BC003431 | Spint2 | 0.91 | serine protease inhibitor, Kunitz type 2, mRNA (cDNA clone MGC:6479 IMAGE:2646512), complete cds. |
| BC003708 | Ptges3 | 0.91 | prostaglandin E synthase 3 (cytosolic), mRNA (cDNA clone MGC:5681 IMAGE:3489418), complete cds. |
| BC003872 | Tspan15 | 0.91 | tetraspanin 15, mRNA (cDNA clone MGC:6632 IMAGE:3492667), complete cds. |
| BC004688 | Nxn | 0.91 | nucleoredoxin, mRNA (cDNA clone MGC:7861 IMAGE:3501314), complete cds. |
| BC005695 | Adi1 | 0.91 | acireductone dioxygenase 1, mRNA (cDNA clone MGC:11855 IMAGE:3597652), complete cds. |
| BC006895 | Ergic2 | 0.91 | ERGIC and golgi 2, mRNA (cDNA clone MGC:6874 IMAGE:2651322), complete cds. |
| BC006956 | Tmc6 | 0.91 | transmembrane channel-like gene family 6, mRNA (cDNA clone IMAGE:3707726), complete cds. |
| BC009002 | Rnd3 | 0.91 | Rho family GTPase 3, mRNA (cDNA clone MGC:6381 IMAGE:3500586), complete cds. |
| BC016576 | Isoc1 | 0.91 | isochorismatase domain containing 1, mRNA (cDNA clone MGC:27719 IMAGE:2609919), complete cds. |
| BC025172 | Mb | 0.91 | myoglobin, mRNA (cDNA clone MGC:36715 IMAGE:3979003), complete cds. |
| BC026774 | Nkap | 0.91 | NFKB activating protein, mRNA (cDNA clone MGC:25785 IMAGE:4019392), complete cds. |
| BC027099 | Ubxn10 | 0.91 | UBX domain containing 3, mRNA (cDNA clone MGC:32424 IMAGE:5041000), complete cds. |
| BC030351 | 2900062L11Rik | 0.91 | RIKEN cDNA 2900062L11 gene, mRNA (cDNA clone MGC:40746 IMAGE:5365902), complete cds. |
| BC031374 | Plekhn1 | 0.91 | pleckstrin homology domain containing, family N member 1, mRNA (cDNA clone IMAGE:5358510), with apparent retained intron. |
| BC034089 | Hap1 | 0.91 | huntingtin-associated protein 1, mRNA (cDNA clone MGC:31449 IMAGE:4481735), complete cds. |
| BC034120 | Vit | 0.91 | vitrin, mRNA (cDNA clone MGC:31645 IMAGE:4527027), complete cds. |
| BC038937 | 9230105E10Rik | 0.91 | RIKEN cDNA 9230105E10 gene, mRNA (cDNA clone MGC:46806 IMAGE:2648191), complete cds. |
| BC048491 | Npm3 | 0.91 | nucleoplasmin 3, mRNA (cDNA clone MGC:58307 IMAGE:6704326), complete cds. |
| BC048680 | Ropn1l | 0.91 | ropporin 1-like, mRNA (cDNA clone MGC:58858 IMAGE:6774441), complete cds. |
| BC050127 | Inpp5f | 0.91 | inositol polyphosphate-5-phosphatase F, mRNA (cDNA clone IMAGE:30021371), partial cds. |
| BC050799 | Spata18 | 0.91 | spermatogenesis associated 18, mRNA (cDNA clone IMAGE:6743872), partial cds. |
| BC053493 | Trh | 0.91 | thyrotropin releasing hormone, mRNA (cDNA clone MGC:60687 IMAGE:30010395), complete cds. |
| BC055780 | B630019K06Rik | 0.91 | RIKEN cDNA B630019K06 gene, mRNA (cDNA clone MGC:67565 IMAGE:6403722), complete cds. |
| BC060996 | 1700001C02Rik | 0.91 | RIKEN cDNA 1700001C02 gene, mRNA (cDNA clone MGC:74094 IMAGE:6743526), complete cds. |
| BC061017 | 1700009P17Rik | 0.91 | RIKEN cDNA 1700009P17 gene, mRNA (cDNA clone MGC:74123 IMAGE:6774925), complete cds. |
| BC092288 | Nr2c2ap | 0.91 | RIKEN cDNA 2310073E15 gene, mRNA (cDNA clone MGC:103332 IMAGE:5003230), complete cds. |
| BC094371 | N/A | 0.91 | cDNA clone IMAGE:6849194, with apparent retained intron. |
| BC094614 | Pfn4 | 0.91 | profilin family, member 4, mRNA (cDNA clone IMAGE:6852241), with apparent retained intron. |
| BC095964 | Bcl2 | 0.91 | B-cell leukemia/lymphoma 2, mRNA (cDNA clone MGC:106289 IMAGE:6306635), complete cds. |
| BC117923 | Gprasp2 | 0.91 | G protein-coupled receptor associated sorting protein 2, mRNA (cDNA clone MGC:144083 IMAGE:40097504), complete cds. |
| BC119578 | C130060K24Rik | 0.91 | RIKEN cDNA C130060K24 gene, mRNA (cDNA clone MGC:141328 IMAGE:40058179), complete cds. |
| BC120498 | Slc18a3 | 0.91 | solute carrier family 18 (vesicular monoamine), member 3, mRNA (cDNA clone MGC:155735 IMAGE:40129421), complete cds. |
| BC120504 | Chrm4 | 0.91 | cholinergic receptor, muscarinic 4, mRNA (cDNA clone MGC:155741 IMAGE:40129427), complete cds. |
| BC120512 | Paqr6 | 0.91 | progestin and adipoQ receptor family member VI, mRNA (cDNA clone MGC:155749 IMAGE:40129435), complete cds. |
| BC120892 | Srgap1 | 0.91 | SLIT-ROBO Rho GTPase activating protein 1, mRNA (cDNA clone MGC:141420 IMAGE:40061454), complete cds. |
| BC131649 | Gli1 | 0.91 | GLI-Kruppel family member GLI1, mRNA (cDNA clone IMAGE:40110424), complete cds. |
| BC146307 | Slc6a11 | 0.91 | solute carrier family 6 (neurotransmitter transporter, GABA), member 11 (Slc6a11) mRNA, encodes complete protein. |
| BC148239 | Tas2r143 | 0.91 | taste receptor, type 2, member 143, mRNA (cDNA clone MGC:165170 IMAGE:40050382), complete cds. |
| BC152544 | Sncaip | 0.91 | cDNA clone IMAGE:40141761. |
| BC152724 | Ddc | 0.91 | dopa decarboxylase (Ddc) mRNA, encodes complete protein. |
| EF651818 | ENSMUSG00000072735 | 0.91 | alpha21-takusan mRNA, complete cds. |
| NM_001085543 | Gm14347 | 0.91 | predicted gene, OTTMUSG00000016407 (OTTMUSG00000016407), mRNA. |
| NR_001583 | Speer9-ps1 | 0.91 | spermatogenesis associated glutamate (E)-rich protein 9, pseudogene 1 (Speer9-ps1) on chromosome 7. |
| XM_001473569 | C730027H18Rik | 0.91 | RIKEN cDNA C730027H18 gene (C730027H18Rik), mRNA. |
| XM_001473776 | Gm14702 | 0.91 | hypothetical LOC671950 (LOC671950), mRNA. |
| XM_001473858 | Gm15411 | 0.91 | hypothetical protein LOC100039964 (LOC100039964), mRNA. |
| XM_001474814 | Fuz | 0.91 | fuzzy homolog (Drosophila) (Fuz), mRNA. |
| AB011499 | Ptk2 | 0.91 | focal adhesion kinase spliced variant p110FAK, complete cds. |
| AF188008 | Plec1 | 0.91 | plectin isoform plec 1,2alpha (Plec1) mRNA, partial cds. |
| AF199608 | Fgf13 | 0.91 | fibroblast growth factor homologous factor 2 isoform 1P+1Y'+1V (FHF-2) mRNA, partial cds. |
| AF281634 | Zfp422 | 0.91 | Kruppel-type zinc finger protein KROX-25 mRNA, complete cds. |
| AF287732 | Kcnip3 | 0.91 | alt '4933407H12Rik#AI413860#Csen#DREAM#KChIP3#R74849' |
| AF459018 | C1rb | 0.91 | complement component C1RB (C1rb) mRNA, complete cds. |
| AK008258 | Otub2 | 0.91 | RIKEN cDNA 4930586I02 gene, full insert sequence. |
| AK009214 | 2610203C22Rik | 0.91 | unclassifiable, full insert sequence. |
| AK011047 | Herc5 | 0.91 | hypothetical Regulator of chromosome condensation (RCC1) containing protein, full insert sequence. |
| AK012841 | 2810029C07Rik | 0.91 | unclassifiable, full insert sequence. |
| AK015684 | Lrrc57 | 0.91 | hypothetical Leucine-rich repeat/Leucine-rich repeat, typical subtype containing protein, full insert sequence. |
| AK030214 | Tubd1 | 0.91 | tubulin, delta 1, full insert sequence. |
| AK032568 | Galnt9 | 0.91 | UDP-GALNAC: POLYPEPTIDE N-ACETYLGALACTOSAMINYLTRANSFERASE homolog [Homo sapiens], full insert sequence. |
| AK032961 | Ccnf | 0.91 | cyclin F, full insert sequence. |
| AK032980 | Cdca4 | 0.91 | HEMATOPOIETIC PROGENITOR PROTEIN, full insert sequence. |
| AK035657 | AI314180 | 0.91 | KIAA0368 PROTEIN (FRAGMENT) homolog [Homo sapiens], full insert sequence. |
| AK036509 | Igfn1 | 0.91 | hypothetical Fibronectin type III domain/Fibronectin type III repeat containing protein, full insert sequence. |
| AK038528 | N28178 | 0.91 | hypothetical EF-hand structure containing protein, full insert sequence. |
| AK038650 | Jmjd4 | 0.91 | unnamed protein product {Homo sapiens}, full insert sequence. |
| AK038986 | Rnf14 | 0.91 | ring finger protein 14, full insert sequence. |
| AK042240 | Itpr1 | 0.91 | hypothetical protein, full insert sequence. |
| AK043581 | N/A | 0.91 | unclassifiable, full insert sequence. |
| AK049880 | Fam19a1 | 0.91 | unclassifiable, full insert sequence. |
| AK050613 | Arpp21 | 0.91 | protein phosphatase 1, regulatory (inhibitor) subunit 1C, full insert sequence. |
| AK077603 | Mef2c | 0.91 | myocyte enhancer factor 2C, full insert sequence. |
| AK079080 | Pak7 | 0.91 | p21 (CDKN1A)-activated kinase 7, full insert sequence. |
| AK089260 | Nlrx1 | 0.91 | hypothetical P-loop containing nucleotide triphosphate hydrolases structure containing protein, full insert sequence. |
| AK090085 | Rasgef1b | 0.91 | hypothetical Guanine-nucleotide dissociation stimulators CDC25 family containing protein, full insert sequence. |
| AK131707 | BC016423 | 0.91 | Serine/threonine-specific protein phosphatase and bis(5-nucleosyl)-tetraphosphatase containing protein, full insert sequence. |
| AK133759 | Clcn4-2 | 0.91 | chloride channel 4-2, full insert sequence. |
| AK135845 | C86187 | 0.91 | hypothetical protein, full insert sequence. |
| AK136474 | Upp1 | 0.91 | uridine phosphorylase 1, full insert sequence. |
| AK136939 | Palmd | 0.91 | palmdelphin, full insert sequence. |
| AK137265 | D1Ertd622e | 0.91 | hypothetical gene supported by AF038182, BC009203 (DNA segment, Chr 1, ERATO Doi 622, expressed), full insert sequence. |
| AK141157 | Zeb2 | 0.91 | zinc finger homeobox 1b, full insert sequence. |
| AK143111 | Herc4 | 0.91 | hect domain and RLD 3, full insert sequence. |
| AK144338 | Ncoa6 | 0.91 | nuclear receptor coactivator 6, full insert sequence. |
| AK149514 | Pde4c | 0.91 | phosphodiesterase 4C, cAMP specific, full insert sequence. |
| AK150309 | Ppm1h | 0.91 | Hypothetical protein phosphatase 2C domain containing protein homolog [Mus musculus], full insert sequence. |
| AK155969 | Nfkbid | 0.91 | IkappaBNS, full insert sequence. |
| AK157951 | Col27a1 | 0.91 | procollagen, type XXVII, alpha 1, full insert sequence. |
| AK161752 | Cacna1a | 0.91 | calcium channel, voltage-dependent, P/Q type, alpha 1A subunit, full insert sequence. |
| AK162596 | Hisppd2a | 0.91 | KIAA0377-like protein homolog [Mus musculus], full insert sequence. |
| AK163851 | Col13a1 | 0.91 | procollagen, type XIII, alpha 1, full insert sequence. |
| AK166215 | Slc12a7 | 0.91 | solute carrier family 12, member 7, full insert sequence. |
| AK172428 | Ubxn1 | 0.91 | hypothetical protein, full insert sequence. |
| AY861419 | Pcdh7 | 0.91 | alt '-' |
| BC002049 | Tgfb1i1 | 0.91 | transforming growth factor beta 1 induced transcript 1, mRNA (cDNA clone MGC:6057 IMAGE:3489459), complete cds. |
| BC003316 | Klf10 | 0.91 | Kruppel-like factor 10, mRNA (cDNA clone MGC:7000 IMAGE:3155247), complete cds. |
| BC004637 | C1ra | 0.91 | complement component 1, r subcomponent, mRNA (cDNA clone MGC:6404 IMAGE:3586055), complete cds. |
| BC004794 | Pgap2 | 0.91 | FGF receptor activating protein 1, mRNA (cDNA clone MGC:8108 IMAGE:3588752), complete cds. |
| BC004809 | Pdlim1 | 0.91 | PDZ and LIM domain 1 (elfin), mRNA (cDNA clone MGC:5634 IMAGE:3588132), complete cds. |
| BC016434 | Mafb | 0.91 | v-maf musculoaponeurotic fibrosarcoma oncogene family, protein B (avian), mRNA (cDNA clone MGC:25341 IMAGE:4485468), complete cds. |
| BC018385 | Sh3gl2 | 0.91 | SH3-domain GRB2-like 2, mRNA (cDNA clone MGC:25314 IMAGE:4503038), complete cds. |
| BC021788 | Rbpms2 | 0.91 | RNA binding protein with multiple splicing 2, mRNA (cDNA clone MGC:28665 IMAGE:4236661), complete cds. |
| BC023020 | Actr1b | 0.91 | ARP1 actin-related protein 1 homolog B (yeast), mRNA (cDNA clone MGC:36526 IMAGE:5375425), complete cds. |
| BC024472 | Crtac1 | 0.91 | cartilage acidic protein 1, mRNA (cDNA clone MGC:37356 IMAGE:4976509), complete cds. |
| BC024864 | Stac2 | 0.91 | SH3 and cysteine rich domain 2, mRNA (cDNA clone MGC:38869 IMAGE:5361431), complete cds. |
| BC026941 | 1810065E05Rik | 0.91 | RIKEN cDNA 1810065E05 gene, mRNA (cDNA clone MGC:25794 IMAGE:4024443), complete cds. |
| BC031745 | Camk2a | 0.91 | calcium/calmodulin-dependent protein kinase II alpha, mRNA (cDNA clone MGC:25415 IMAGE:4506005), complete cds. |
| BC044878 | Rinl | 0.91 | RIKEN cDNA 5830482F20 gene, mRNA (cDNA clone IMAGE:3589197), complete cds. |
| BC047201 | Mapk1ip1l | 0.91 | RIKEN cDNA C130032J12 gene, mRNA (cDNA clone MGC:56754 IMAGE:6396409), complete cds. |
| BC049186 | Enc1 | 0.91 | ectodermal-neural cortex 1, mRNA (cDNA clone MGC:54719 IMAGE:6485551), complete cds. |
| BC058678 | Trim37 | 0.91 | tripartite motif protein 37, mRNA (cDNA clone MGC:76361 IMAGE:6834513), complete cds. |
| BC064068 | Hlx | 0.91 | H2.0-like homeobox, mRNA (cDNA clone MGC:73651 IMAGE:1380899), complete cds. |
| BC085276 | Melk | 0.91 | maternal embryonic leucine zipper kinase, mRNA (cDNA clone MGC:102507 IMAGE:30443236), complete cds. |
| BC094276 | Kcne1 | 0.91 | potassium voltage-gated channel, Isk-related subfamily, member 1, mRNA (cDNA clone MGC:106148 IMAGE:4189679), complete cds. |
| BC099699 | Tesk1 | 0.91 | testis specific protein kinase 1, mRNA (cDNA clone MGC:106523 IMAGE:6821934), complete cds. |
| AJ413953 | Arfrp1 | 0.9 | ARF-related protein 1 (Arfrp1 gene). |
| AK015921 | Sv2c | 0.9 | Weakly similar to SYNAPTIC VESICLE PROTEIN 2C, full insert sequence. |
| NG_033949.1 | KMT2E | 0.9 | lysine (K)-specific methyltransferase 2E |
| BC003897 | Arl6ip5 | 0.9 | ADP-ribosylation factor-like 6 interacting protein 5, mRNA (cDNA clone MGC:6760 IMAGE:3600236), complete cds. |
| BC050865 | Pknox2 | 0.9 | Pbx/knotted 1 homeobox 2, mRNA (cDNA clone MGC:59500 IMAGE:6332968), complete cds. |
| AK014857 | 4921509O09Rik | 0.89 | Unclassifiable, full insert sequence. |
| AK032260 | Cacng5 | 0.89 | Voltage-dependent calcium channel gamma-5 subunit, full insert sequence. |
| AK034133 | Slc29a3 | 0.84 | EQUILIBRATIVE NUCLEOSIDE TRANSPORTER 3, full insert sequence. |
| AK014042 | Sirt2 | 0.83 | Sirtuin 2 (silent mating type information regulation 2, homolog) 2 (S. cerevisiae), full insert sequence. |
| AK013623 | Xlr4c | 0.8 | X-LINKED LYMPHOCYTE REGULATED GENE 4 homolog, full insert sequence. |
| AK030943 | Edem1 | 0.8 | EDEM PROTEIN homolog, full insert sequence. |
| AK030726 | Galnt13 | 0.79 | Similar to POLYPEPTIDE N-ACETYLGALACTOSAMINYLTRANSFERASE (EC 2.4.1.41) (PROTEIN- UDP ACETYLGALACTOSAMINYLTRANSFERASE) (UDP-GALNAC:POLYPEPTIDE, N-ACETYLGALACTOSAMINYLTRANSFERASE) (GALNAC-T1) [Bos taurus], full insert sequence. |
| AK002300 | Alad | 0.77 | aminolevulinate, delta-, dehydratase |
| AK015826 | Galntl6 | 0.76 | Hypothetical Ricin B-like lectin structure containing protein, full insert sequence. |
| AK009857 | Smpx | 0.74 | Small muscle protein, X-linked, full insert sequence. |
| AK015218 | Chd1 | 0.69 | Chromodomain helicase DNA binding protein 1, full insert sequence. |
| AK013805 | Pmm1 | 0.68 | Phosphomannomutase 1, full insert sequence. |
| AK002830 | Irf7 | 0.66 | Interferon regulatory factor 7, full insert sequence. |
| AK033281 | Cep250 | 0.66 | SIMILAR TO CENTROSOMAL PROTEIN 2 homolog, full insert sequence. |
| AJ243657 | Eda | 0.63 | Ectodysplasin, isoform Ta-A2 (Tabby gene). |
| AK031337 | Fads2 | 0.63 | Fatty acid desaturase 2, full insert sequence. |
| BC145708 | B630005N14Rik | 0.62 | RIKEN cDNA B630005N14 gene, mRNA (cDNA clone MGC:175595 IMAGE:40131011), complete cds. |
| AJ130943 | Slc7a7 | 0.61 | Glycoprotein-associated amino acid transporter y+LAT1b. |
| AK030632 | Zfp14 | 0.61 | Zinc finger protein 14, full insert sequence. |
| AF167552 | Tnfrsf19 | 0.6 | TAJ-alpha long mRNA, complete cds. |
| AK034753 | Ptprm | 0.59 | Protein tyrosine phosphatase, receptor type, M, full insert sequence. |
| BC131644 | Mkl2 | 0.58 | MKL/myocardin-like 2, mRNA (cDNA clone MGC:150100 IMAGE:40110019), complete cds. |
| AK007524 | Fam32a | 0.57 | Hypothetical protein, full insert sequence. |
| AF536772 | Robo4 | 0.56 | Roundabout-like protein ROBO4 mRNA, complete cds. |
| BC140373 | Kif17 | 0.56 | Kinesin family member 17 (Kif17) mRNA, encodes complete protein. |
| AF479773 | Usf1 | 0.55 | UPstream transcription factor 1 short form mRNA, complete cds. |
| AK014813 | Esyt2 | 0.55 | CHR2 SYNAPTOTAGMIN (FRAGMENT) homolog, full insert sequence. |
| AK034254 | Fam161b | 0.55 | Similar to CDNA FLJ31697 FIS, CLONE NT2RI2005851, WEAKLY SIMILAR TO PLECTIN [Homo sapiens], full insert sequence. |
| BC130237 | V1rd15 | 0.55 | Vomeronasal 1 receptor, D15, mRNA (cDNA clone MGC:161285 IMAGE:40142092), complete cds. |
| AF401983 | Zim2 | 0.53 | Imprinted zinc-finger 2 gene, partial sequence. |
| BC127981 | Olfr641 | 0.53 | Olfactory receptor 641, mRNA (cDNA clone MGC:157539 IMAGE:40135228), complete cds. |
| AK033562 | St3gal6 | 0.52 | Sialyltransferase 10 (alpha-2,3-sialyltransferase VI), full insert sequence. |
| AF475074 | Ntng1 | 0.2 | Domesticus laminet-1F (Lmnt1) mRNA, complete cds |
| BC053751 | Nsun6 | 0.096 | NOL1/NOP2/Sun domain family 6, mRNA (cDNA clone MGC:59523 IMAGE:6335896), complete cds. |
| BC034161 | Sh3bgrl | 0.092 | SH3-binding domain glutamic acid-rich protein like, mRNA (cDNA clone MGC:35895 IMAGE:4954986), complete cds. |

Enrichment in AGO-miRNPs after miR-1 transfection, n=3161

The relative enrichment for G6PD in the miRNPs following miR-1 transfection is >50-fold, which held consistently across all experiments. The vast majority of mRNAs were not enriched in the miRNPs following miR-1 transfection.
